# *BRAF* mutation-specific promoter methylation of *FOX* genes in colorectal cancer

**DOI:** 10.1186/1868-7083-5-2

**Published:** 2013-01-16

**Authors:** Eddy H van Roon, Arnoud Boot, Ashwin A Dihal, Robert F Ernst, Tom van Wezel, Hans Morreau, Judith M Boer

**Affiliations:** 1 Center for Human and Clinical Genetics, Leiden University Medical Center, Leiden, The Netherlands; 2 Department of Pathology, Leiden University Medical Center, Leiden, The Netherlands; 3 Department of Pediatric Oncology, Erasmus MC - Sophia Children’s Hospital, Rotterdam, The Netherlands; 4 Netherlands Bioinformatics Center, Nijmegen, the Netherlands

**Keywords:** *BRAF* mutation, Colorectal cancer, DNA methylation, Histone pre-marking

## Abstract

**Background:**

Cancer-specific hypermethylation of (promoter) CpG islands is common during the tumorigenesis of colon cancer. Although associations between certain genetic aberrations, such as *BRAF* mutation and microsatellite instability, and the CpG island methylator phenotype (CIMP), have been found, the mechanisms by which these associations are established are still unclear. We studied genome-wide DNA methylation differences between colorectal tumors carrying a *BRAF* mutation and *BRAF* wildtype tumors.

**Results:**

Using differential methylation hybridization on oligonucleotide microarrays representing 32,171 CpG-rich regions, we identified 1,770 regions with differential methylation between colorectal tumor and paired normal colon. Next, we compared the tumor/normal methylation ratios between different groups of patients. Related to CIMP, we identified 749 differentially methylated regions, of which 86% had a higher tumor/normal methylation ratio in the CIMP-positive group. We identified 758 regions with a *BRAF* mutation-specific methylation change, of which 96% had a higher tumor/normal methylation ratio in the *BRAF* mutant group. Among the genes affected by *BRAF* mutation-specific methylation changes, we found enrichment of several cancer-related pathways, including the PI3 kinase and Wnt signaling pathways. To focus on genes that are silenced in a tumor-specific rather than a lineage-specific manner, we used information on the epigenetic silencing mark H3K27me^3^ in embryonic stem (ES) cells. Among the genes showing *BRAF* mutation-specific promoter methylation but no H3K27me^3^ mark in ES cells were forkhead box (*FOX*) transcription factors associated with the PI3 kinase pathway, as well as *MLH1* and *SMO*. Repression of *FOXD3* gene expression in tumors could be related to its promoter hypermethylation.

**Conclusions:**

We identified new *BRAF* mutation-specific methylation changes in colorectal cancer. Epigenetic downregulation of these targets may contribute to mutationally active *BRAF*-driven tumorigenesis, explaining its association with aberrant DNA methylation.

## Background

The CpG island methylator phenotype (CIMP) was introduced in 1999 by Toyota *et al*. to describe a subset of colorectal tumors with high levels of cancer-specific methylation [1]. Subsequent studies regarding CIMP in colon cancer described a strong association between this epigenetic phenotype, *BRAF* mutations, and microsatellite instability (MSI) [2-8]. As sporadic MSI colon cancer is caused by promoter methylation of a mismatch repair gene (*MLH1*, *MSH2*, or *MSH6*) the association between MSI and the high levels of DNA methylation in CIMP is considered a causative one [9,10]. However, the association between activating *BRAF* mutations and CIMP remains unclear.

The field of epigenetic research has progressed from a candidate-gene to a genome-wide approach, which not only provides a plethora of new candidate targets of cancer-specific DNA methylation but also a better understanding of transcription regulation by DNA methylation [11]. Using such genome-wide DNA methylation approaches could help to identify new targets of *BRAF* mutation-specific promoter methylation. Hinoue *et al*. [2] examined the CIMP- and *BRAF* mutation-specific methylation status of 1,505 CpG sites, located at 807 genes, in 235 primary colorectal tumors and discovered specific methylation of genes mediating various signaling pathways involved in colon cancer tumorigenesis. In this study, we screened 32,171 CpG sites located at 10,537 genes in a selected cohort of 19 patients with right-sided colon cancer to obtain additional insight into the association between *BRAF* mutations and DNA methylation in colon cancer tumorigenesis. Recent studies have described a gradual increase in CIMP and *BRAF* mutation prevalence from the rectum to the ascending colon [12,13]. To avoid tumor location as a factor that could possibly influence the levels of methylation, we specifically studied tumors originating from the ascending colon and cecum. The frequency of *BRAF* mutations in the CIMP-positive patients was comparable to those previously described in larger cohort studies [2,8,14,15].

Recent publications have reported a possible pre-marking of cancer-specific hypermethylated genes by the inactivation mark histone H3 lysine 27 trimethylation (H3K27me^3^) and binding of the polycomb group member SUZ12 in both ES cells and differentiated normal colon mucosal tissue [16-18]. These studies led to the suggestion that colon cancer cells utilize a pre-existing repression program to target loci for cancer-specific promoter methylation [16,18-20]. However, the presence of such repressive histone modifications at promoters during differentiation from ES to normal colon epithelium suggests that the associated genes are at a transcriptional silent state prior to tumor formation, reducing the relevance of the DNA methylation of pre-marked genes to tumorigenesis. In an attempt to identify biologically relevant *BRAF* mutation-specific promoter methylation, we excluded loci with H3K27me^3^ pre-marking in ES cells from the functional pathway analyses. By both extending the number of screened loci and filtering out pre-marked genes, we identified new targets of *BRAF* mutation-specific methylation that could either create a favorable setting for the acquisition of *BRAF* mutations or function as an addition to up-regulation of the RAS-RAF-MEK pathway.

## Results

### Colon cancer-specific CpG island methylation

We identified 1,770 CpG-rich regions with significant methylation differences between tumor and paired normal colon. Of these, 1,234 differentially methylated regions were associated with 816 genes, of which 531 were localized to gene promoters (Additional file 1). As expected, CpG islands were mostly found hypermethylated in tumors (78.8%) [11].

We compared our results with those of Irizarry *et al*. [11], who described 2,707 cancer-specific differentially methylated regions based on the comparison of 13 colorectal cancer tumor-normal pairs. Of the described differentially methylated regions, 1,203 overlapped with our CpG island array regions, of which 282 (23%) were also differentially methylated between tumor and normal in our analysis. This overlap is reasonable, considering the different, modest-sized patient groups, and different experimental approaches.

### CIMP-specific methylation

Next, we compared the tumor/normal methylation ratios between different groups of patients. Between CIMP-positive (*n* = 11) and CIMP-negative (*n* = 8) patients, 749 CpG-rich regions showed methylation changes, of which 85.6% had a higher tumor/normal methylation ratio in the CIMP-positive group. Of these differentially methylated regions, 589 were associated with 508 genes, of which 244 were localized to gene promoters (Additional file 2). In 8 out of 11 CIMP-positive tumors, promoter methylation of *MLH1*, the cause of microsatellite instability in sporadic colon cancer, was observed, which was consistent with methylation-specific PCR (Additional file 3). We conclude that the hypermethylation in specific genomic regions used to define CIMP [6] is associated with methylation changes throughout the genome.

### *BRAF* mutation-specific methylation

Activating *BRAF* mutations have been associated with high levels of CpG island methylation and MSI in colon cancer [2-8]. To investigate this association, we compared the tumor/normal methylation ratio profiles of *BRAF* wildtypes (*n* = 11) with those containing the *BRAF*^*V600E*^ mutation (*n* = 8). We identified 758 regions with a *BRAF* mutation-specific methylation change, of which 96.3% had a higher tumor/normal methylation ratio in the *BRAF* mutant group. Out of these 758 regions, 579 were associated with 479 genes, of which 229 were localized to gene promoters (Additional file 4).

**Figure 1:**
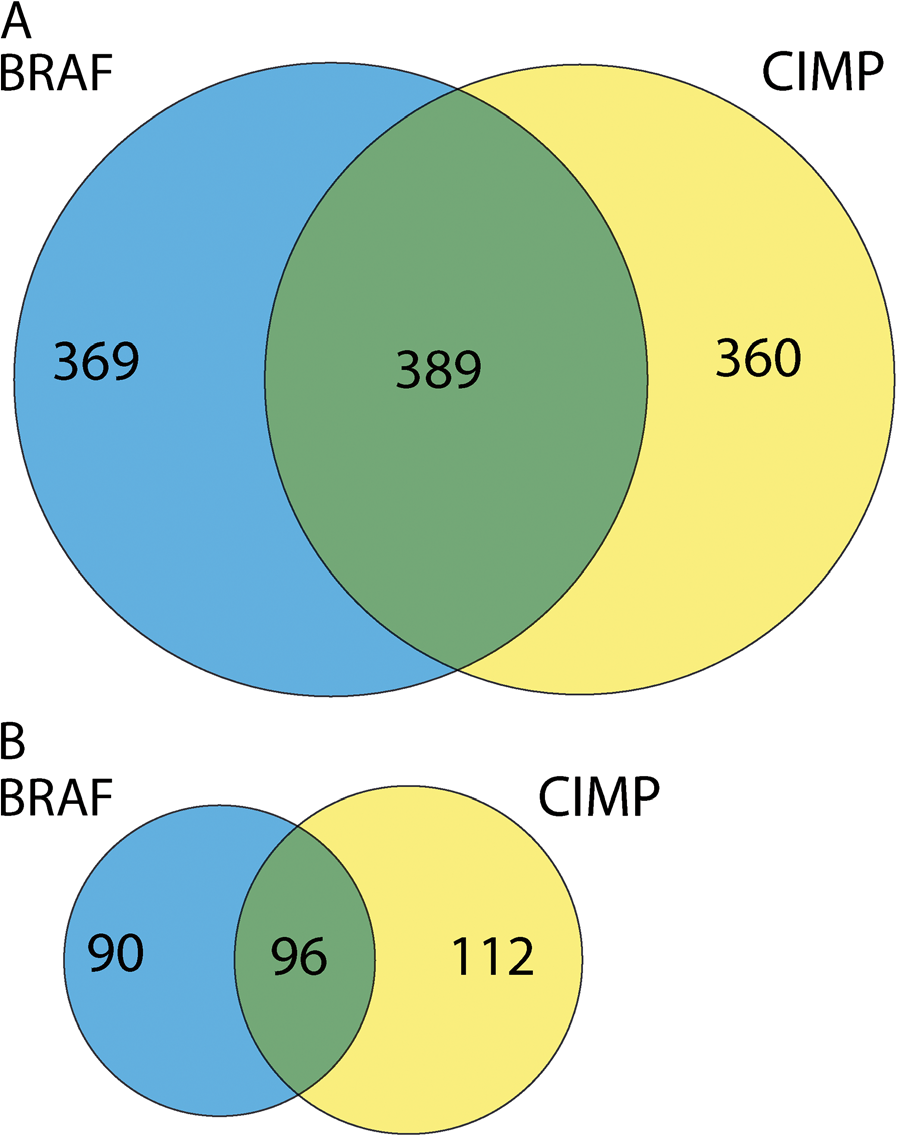
**Proportional Venn diagrams showing the overlap between *BRAF *mutation- (blue) and CIMP-specific (yellow) methylation changes (A) for all CpG-rich regions and (B) for promoter regions filtered for H3K27me**^**3 **^**binding in embryonic stem cells.**

Since *BRAF* mutations and CIMP co-occurred in eight samples, as expected from other studies [8,14,15], there was a high level of overlap between CIMP- and *BRAF* mutation-specific methylation changes (Figure 1A). Comparable levels of overlap were found, focusing on promoter regions only (data not shown).

### Regions with methylation changes are enriched for inactive chromatin marks

Regions binding the polycomb repressor complex 2 (PRC2) component SUZ12 in ES cells were found to be enriched among the loci differentially methylated between colon cancer and normal colon (Table 1). The histone mark H3K27me^3^ is mediated by the PRC2 complex [21], and the two marks have been reported to be highly correlated [17]. Enrichment of ES cell H3K27me^3^ binding regions among the regions with colon cancer-associated methylation changes was, therefore, expected and was indeed observed. Similarly, regions with CIMP- and *BRAF* mutation-associated differential methylation changes were also highly enriched for regions binding SUZ12 and H3K27me^3^ in ES cells (Table 1). Additionally, sites binding CTCF and the active chromatin mark H3K4me^3^ were underrepresented among the differentially methylated regions. Interestingly, although all colon cancer-, CIMP-, and *BRAF* mutation-specific differentially methylated regions are underrepresented for H3K4me^3^, this depletion is most evident for *BRAF* mutation-specific regions.

**Table 1:** Overlap between regions with methylation changes in colorectal cancer with chromatin marks in ES cells

	**Total**	**SUZ12 *P***^a^	**H3K27me**^**3 **^***P***	**CTCF *P***	**H3K4me**^**3 **^***P***
**Colon tumor regions**	1,770	540 (30.5%) 0	561 (31.7%) 0	379 (21.4%) 0	711 (40.2%) 0.000,012
**Remaining regions**	30,401	3,447 (11.3%)	3,336 (11%)	10,137 (33.3%)	13,835 (45.5%)
**CIMP-specific regions**	749	160 (21.4%) 0	169 (22.6%) 0	183 (24.4%) 0.000,001	300 (40.1%) 0.004,6
**Remaining regions**	31,422	3,827 (12.2%)	3,728 (11.9%)	10,333 (32.9%)	14,246 (45.3%)
***BRAF*-specific regions**	758	171 (22.6%) 0	180 (23.7%) 0	195 (25.7%) 0.000,035	169 (22.3%) 0
**Remaining regions**	31,413	3,816 (12.1%)	3,717 (11.8%)	10,321 (32.8%)	14,377 (45.8%)

^a^*P* values are those for the *χ*-squared test.

After exclusion of regions with H3K27me^3^ pre-marking in ES cells, the overlap between CIMP- and *BRAF* mutation-specific methylation changes for all loci (not shown) and promoters (Figure 1B) remained highly significant. Despite this high level of overlap, approximately 50% of *BRAF* mutation-specific methylation changes showed no overlap with CIMP. In our functional analysis, we focused on all promoter regions with *BRAF* mutation-specific methylation changes, regardless of overlap with CIMP.

### *BRAF* mutation-associated methylation pathway analysis

To identify biological pathways affected by *BRAF* mutation-associated gene methylation, we used 186 promoter regions that did not bind H3K27me^3^ in ES cells representing 125 genes after exclusion of duplicates and annotation by Panther 6.0. We found five significantly enriched pathways (*P* < 0.01) containing 13 unique genes (Table 2).

**Table 2:** **Pathways enriched for *BRAF *mutation-associated promoter methylation after exclusion of ES cell H3K27me**^**3 **^**binding promoter regions**

***BRAF *mutation-specific promoters (125)**	**Associated genes**	**Expected hits**^a^	**Hits**	***P***^**b**^
Hedgehog signaling pathway	*SMO*; *GSK3A*^c^; *CREBBP*^c^	0.16	3	0.000561
PI3 kinase pathway	*FOXB1*; *FOXB2*; *FOXD3*; *CCND1*; *GSK3A*^c^	0.72	5	0.000849
Insulin/IGF pathway-protein kinase B signaling cascade	*FOXB1*; *FOXB2*; *FOXD3*; *GSK3A*^c^	0.56	4	0.00252
Wnt signaling pathway	*NKD2*^c^; *GNG4*; *CCND1*; *GSK3A*^c^; *CREBBP*^c^; *AXIN1*; *LEF1*	1.99	7	0.00405
Transcription regulation by bZIP transcription factor	*MTERF*; *CREBBP*^c^*TAF7*	0.33	3	0.00471

^a^Expected hits: expected number of hits by chance in the reference gene list; ^b^*P* values of the binomial test between the *BRAF* mutation-specific gene list and the reference gene list; ^c^ targets with low tumor/normal log2 ratios in the *BRAF* wildtype group, suggesting that additional mechanisms, such as genomic loss, may play a role.

**Figure 2:**
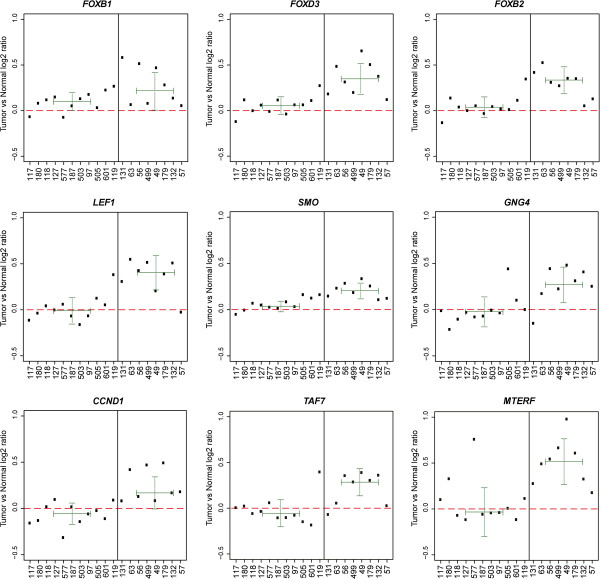
**Scatter plots for nine unique genes with *BRAF *mutation-specific promoter methylation causing pathway enrichment.** The *y* axis represents the tumor/normal log2 ratio for the median probe per region. Sample IDs are given below the *x* axis with *BRAF* wildtypes on the left of the black line and *BRAF* mutants on the right. Median log2 ratios and standard deviations (dotted lines) for the *BRAF* wildtype and *BRAF* mutant groups are given in dark green.

With seven genes, the Wnt pathway contained the most *BRAF* mutation-specific methylation changes (Table 2). However, the tumor/normal log2 ratios (Figure 2) of *AXIN1*, *CREBBP*, *GSK3A*, and *NKD2* in the *BRAF* wildtype samples were low (−0.26 median, 0.12 standard deviation) compared with those in the *BRAF* mutated samples (−0.02 median, 0.12 standard deviation). While this could indicate tumor hypomethylation in *BRAF* wildtype samples compared with normal and *BRAF* mutated samples, the high level of chromosomal instability among *BRAF* wildtype samples suggests that copy-number loss is the most plausible explanation. To filter for this phenomenon, we excluded regions with a log2 ratio below one standard deviation of the median log2 ratio of all *BRAF* mutation-specific regions in the *BRAF* wildtype group. A significant increase in the *BRAF* mutant log2 ratios, compared with those of the *BRAF* wildtypes, indicates *BRAF* mutation-specific hypermethylation in these colon cancer samples (Figure 2). After filtering out copy-number alterations, nine of the pathway-associated genes remained (*SMO*, *FOXB1*, *FOXB2*, *FOXD3*, *CCND1*, *GNG4*, *LEF1*, *MTERF*, *TAF7*) and the PI3 kinase pathway was the only statistically significant enriched (*P* = 0.005) pathway. Interestingly, besides promoter methylation of PI3 kinase pathway-associated forkhead box (*FOX*) genes, we identified promoter methylation of three other *FOX* transcription factors: *FOXA1*, *FOXC1*, and *FOXF1*. However, these promoters were bound by H3K27me^3^ and were excluded from our pathway analysis.

### Validation of promoter methylation of *FOX* genes

**Figure 3:**
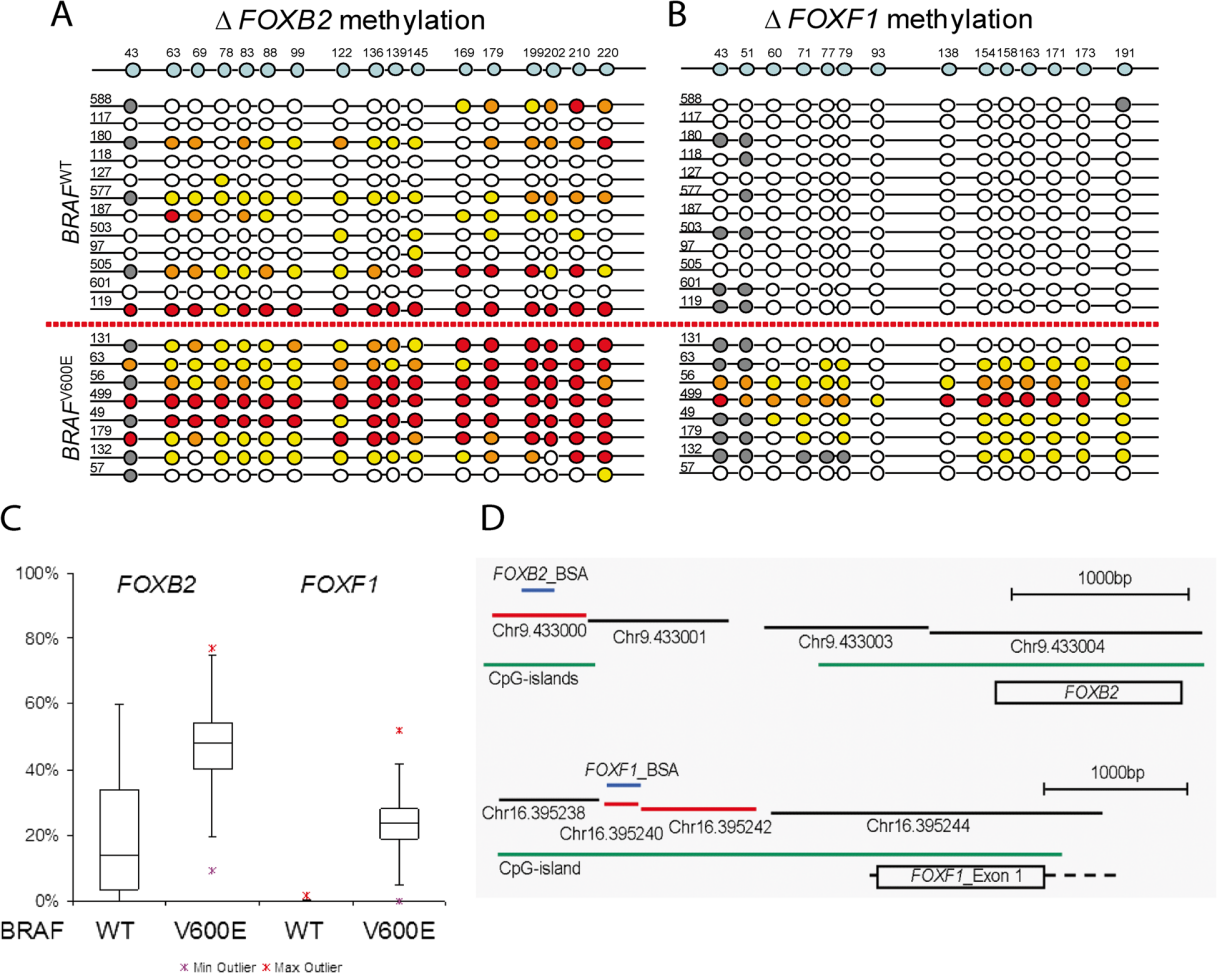
**Tumor hypermethylation of *FOXB2 *and *FOXF1. ***(**A**, **B**) Bisulfite sequence results for 12 *BRAF* wildtype tumors and 8 *BRAF* mutant tumors, separated by the horizontal red line. Schematic display of the BSA region with CpG locations therein shown on top (blue). Sample IDs are given on the 5' end of the BSA region. Each circle depicts a CpG. Color code: gray = ND, white = 0% to 20%, yellow = 20% to 40%, orange = 40% to 60%, red > 60% methylation. (**C**) Boxplot showing average methylation percentage over all detected CpGs in *BRAF* wildtype and *BRAF*^*V600E*^ tumors for *FOXB2* (left) and *FOXF1* (right). The box indicates the 25 and 75 percentiles, with the median shown in bold. Upper and lower outliers are shown as red and purple asterisks, respectively. (**D**) Schematic display of *FOXB2* (top) and *FOXF1* (bottom) loci depicting the location of the BSA regions in relation to MseI fragments detected on the Agilent array (significant fragments marked red, not significant as black). CpG islands are shown in green, and the BSA region is shown in blue.

*FOX* gene promoter hypermethylation in patient samples was validated by bisulfite sequencing analysis (BSA). For *FOXB2* and *FOXF1*, which were found to be hypermethylated in *BRAF* mutated tumors compared with *BRAF* wildtypes, DNA methylation was validated using BSA. Bisulfite sequencing analysis was also attempted for *FOXD3* but was unsuccessful, possibly as a result of the high guanine-cytosine (GC) content [22]. For both promoters, hypermethylation in *BRAF* mutant tumors was confirmed (Figure 3A and B). Methylation levels in normal tissue were below 8% and subtracted from the methylation levels in the corresponding tumors. The average methylation per sample for *BRAF* mutated tumors was significantly higher than that in *BRAF* wildtype tumors (Figure 3C, *FOXB2 P* = 0.0075, *FOXF1 P* = 0.001).

### DNA methylation and gene expression

**Figure 4:**
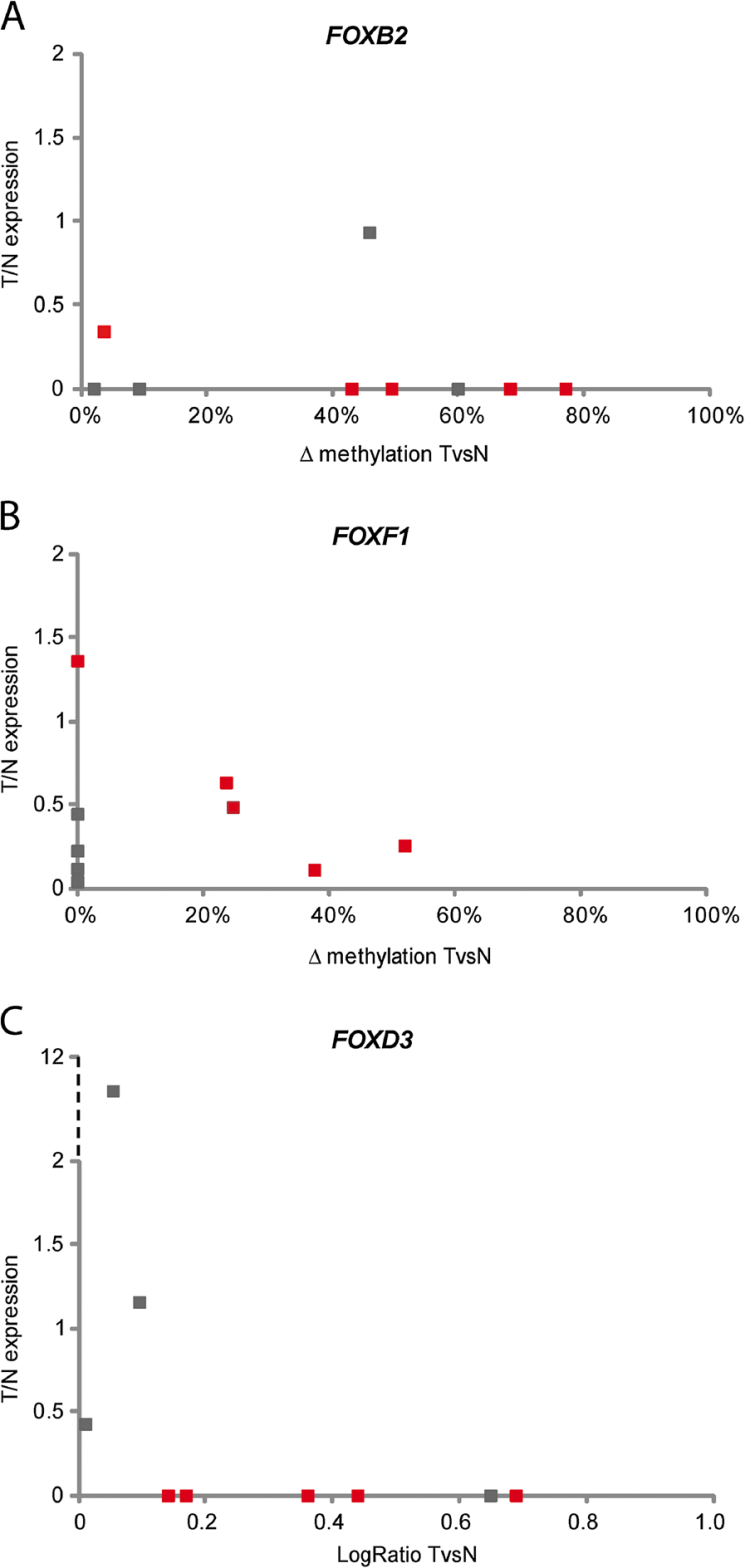
**Relation between methylation and expression.** Tumor/normal expression ratios (*y* axis) were plotted against the average DNA methylation measured by bisulfite sequence analysis for *FOXB2* (**A**) and *FOXF1* (**B**), and the methylation log2 ratio measured by CpG array for *FOXD3* (**C**). *BRAF* mutant samples are shown in red, wildtype samples in black.

For a subset of nine tumor-normal pairs, the expression of *FOXB2*, *FOXD3*, and *FOXF1* was determined using real-time reverse-transcription PCR (RT-qPCR). Only two tumors (both *BRAF* wildtype) had detectable levels of *FOXB2* expression, and an additional three samples showed no detectable *FOXB2* expression in either tumor or normal tissue. *FOXD3* expression was detected in all but one of the normal tissue samples, and in three *BRAF* wildtype tumor samples. A decrease in *FOXF1* expression was observed in all tumors except for sample 57. Next, we compared the tumor/normal expression ratios with the methylation measured by BSA (*FOXB2* and *FOXF1*) or array (*FOXD3*) (Figure 4). *FOXB2* showed loss of expression in tumors, independent of methylation status. Expression of *FOXF1* was repressed in all tumors. In *BRAF* mutated tumors there appeared to be a negative correlation between DNA methylation and expression. The tumors with *FOXD3* hypermethylation showed no detectable *FOXD3* expression, suggesting methylation-related silencing.

## Discussion

In this study, we extended the number of screened CpG loci compared with previous studies performed in context of *BRAF* mutations to identify new *BRAF* mutation-specific methylation changes in colorectal cancer. We validated hypermethylation of forkhead box transcription factors *FOXB2* and *FOXF1* in *BRAF*^*V600E*^ tumors. Additionally, repression of *FOXD3* gene expression in tumors could be related to promoter hypermethylation.

The association between DNA methylation and activating *BRAF* mutations in colon cancer has been identified in several studies [2-8]. Here, we attempted to identify additional targets of *BRAF* mutation-specific DNA methylation that could provide a favorable context, either to obtain a *BRAF* mutation or to attain the full potential of RAS-RAF-MEK-induced proliferation provided by this activating mutation. Identified targets of promoter methylation showing pre-marking by H3K27me^3^ in ES cells were excluded, to filter out methylation changes with minimal expected effects on transcription and thereby tumorigenesis [17]. We showed high levels of overlap between CIMP- and *BRAF* mutation-specific methylation changes, which remained after filtering out pre-marked loci. Although Rada-Iglesias *et al*. [17] showed a higher pre-marking of colon cancer-specific DNA methylation by H3K27me^3^ binding in normal colon epithelium compared with ES cells, we were restricted to using ES cell data, owing to the incompatibility between data formats in our analyses. Interestingly, the promoter region of *MLH1*, found methylated in both a CIMP- and *BRAF* mutation-specific manner, was not filtered out. Therefore, *MLH1* promoter methylation, the cause of sporadic MSI colon cancer, is not established through utilization of a pre-existing repressive program in ES cells.

The study by Hinoue *et al*. [2] described *BRAF* mutation-specific DNA methylation of 60 genes in a comparison of 1,505 CpG sites between 33 *BRAF* mutated tumors and 202 *BRAF* wildtype tumors. The identification of promoter methylation of the mediator of *BRAF*^*V600E*^-induced senescence, *IGFBP7*, led them to suggest that this epigenetic silencing provides a favorable context for the acquisition of *BRAF* mutations [2,23]. Despite differences in experimental techniques and coverage, 10 genes overlapped with our set of *BRAF* mutation-specific methylated regions, including the RAS-RAF hyperactivation-associated *BMP3*, receptor kinases *EPHA3* and *FLT3* as well as the hedgehog signaling protein *SMO*. However, no overlap was found for the mediator of RAS-RAF oncogene-induced senescence, *IGFBP7*, despite coverage of the *IGFBP7* promoter CpG island with two fragments in our assay. Lack of overlap between these studies may be a consequence of different experimental methods as well as of different patient cohorts. Additionally, *BMP3* and *EPHA3* were pre-marked by H3K27me^3^ in our analysis suggesting minimal impact on gene expression and tumorigenesis.

We initially identified enrichment of five cancer-associated pathways by *BRAF* mutation-specific promoter methylation of nine unique genes. Our analysis took into account copy-number changes and filtered for this, as this could improve the reproducibility of differential methylation hybridization (DMH) based assays [24,25]. After exclusion of these loci, the PI3 kinase pathway was the only pathway enriched in our analysis. Among the four genes enriched in this pathway were the *FOX* transcription factors *FOXD3*, *FOXB1*, and *FOXB2*. A recent study described FOXD3 as a TP53 and CDKN1A/p21^cip1^-dependent negative cell cycle regulator, which is suppressed by activated BRAF in melanoma cells [26]. Downregulation of *FOXD3* levels by promoter methylation in colon cancer might provide a favorable setting for either acquisition of a *BRAF* mutation or proliferation by RAS-RAF-MEK over-activation, similar to *IGFBP7*[2].

Interestingly, the *FOXO* transcription factors have also been described as mediators of CDKN2A/p21^cip1^-dependent BRAF-induced senescence, indicating that multiple *FOX* genes are involved in this process [27]. We identified additional *FOX* genes with *BRAF* mutation-specific promoter methylation that were excluded from the pathway analysis as they were pre-marked by H3K27me^3^ in ES cells: *FOXA1*, *FOXC1*, and *FOXF1*. However, the promoters of these genes were also pre-marked with H3K4me^3^ indicating possible tissue-specific expression. All three are targets of inactivation in breast cancer and both *FOXC1* and *FOXF1* are subjected to promoter methylation [28-30]. Most intriguing is the description of FOXF1 as an inducer of G1-S and S-G2 cell cycle arrest, indicating a possible role in oncogene-induced senescence in breast cancer [29]. Our finding that *FOXF1* was downregulated in the *BRAF* mutated cohort suggests that this gene might also play a role in oncogene-induced senescence in colon cancer. However, additional research is required to determine the role of these *FOX* genes in colon cancer-associated oncogene-induced senescence and the impact of their promoter methylation on this mechanism. Finally, research into the sequence of such events is required to provide a better insight in the association between activating *BRAF* mutations and DNA methylation in colon cancer.

## Conclusions

In this study, we identified *BRAF* mutation-specific hypermethylation of CpG regions by DMH on high-density oligonucleotide microarrays. We found enrichment of several cancer-related pathways, including the PI3 kinase and Wnt signaling pathways. We validated differential methylation of forkhead box (*FOX*) transcription factors and found methylation-dependent silencing of *FOXD3*. By both extending the number of screened loci and filtering out genes pre-marked by H3K27me3, we identified new targets of *BRAF* mutation-specific methylation that could provide a favorable context to either obtain a *BRAF* mutation or to attain the full potential of this activating mutation.

## Availability of supporting data

The data set supporting the results of this article is available in the Gene Expression Omnibus (GEO) repository under accession number GSE39334.

## Materials and methods

### Patient material

From anonymized tumor and normal fresh-frozen colon mucosa samples obtained from patients who underwent surgery between 2002 and 2005 at the Leiden University Medical Center (Leiden, The Netherlands) or at the Rijnland Hospital (Leiderdorp, The Netherlands), a cohort containing a high number of CIMP-positive patients was selected. Age, sex, histology, microsatellite instability, and *BRAF*^*V600E*^ status for the 19 patients used for the array profiling are listed in Additional file 3. Prior to DNA isolation, frozen sections were micro-dissected to minimize the presence of normal epithelium and stromal cells. To correct for age-dependent methylation, we used normal mucosa, distant from the tumor, from the same individuals. The patients’ DNA was isolated by phenol and chloroform extraction and ethanol precipitation from 10 to 20 sections of 30 μm. This process yielded 10 to 50 μg of DNA. This study was approved by the Medical Ethics committee of the LUMC (protocol P01-019). Cases were analyzed following the medical ethical guidelines described in the Code Proper Secondary Use of Human Tissue established by the Dutch Federation of Medical Sciences.

### *BRAF* mutation analysis

*BRAF*^*V600E*^ mutations were detected using flanking primers that have been previously described [31]. The products of the PCR were purified with the QIAquick PCR Purification kit (#28106, Qiagen). Sequencing was performed at the Leiden Genome Technology Center (LGTC, Leiden, The Netherlands) using an ABI 3730 xl (Applied Biosystems). Mutational analysis was performed using Mutation Surveyor (SoftGenetics LLC). Results are summarized in Additional file 3.

### Array hybridization

Differential methylation hybridization was performed according to Yan *et al*. [25] DNA (500 ng) was digested with MseI, ligated to linkers, and sequentially digested with two methylation-sensitive restriction enzymes (HpaII #R0171 and BstUI #R0518, New England Biolabs). Digested linker-ligated DNA was used as a template for PCR amplification (20 cycles) and coupled to fluorescent dyes. Cy5- or Cy3-labeled amplicons, representing methylated DNA fragments derived from tumor and normal samples, were co-hybridized to the Agilent 244 k human CpG island microarrays (#G4492A, Agilent Technologies) in a dye-swap setup. Detection was done on a G2565BA scanner (Agilent Technologies) and feature extraction using Feature Extraction Software version 9.5.3.1 (Agilent Technologies).

### Array data analysis

Non-background corrected data were preprocessed by within-array LOESS normalization followed by between-array aquantile normalization using limma v3.2.1 [32] in R2.10.0 [33]. Data were corrected for gene-specific dye bias using R package dyebias v1.4.0 [34]. Raw data and preprocessed log2 ratios (tumor versus normal) per probe are available via the Gene Expression Omnibus (GEO) under accession number GSE39334. Probes mapping to the same MseI fragment were expected to show similar hybridization patterns and not to be independent. Therefore, we mapped probes to the human genome (UCSC assembly March 2006) cut *in silico* with MseI. Fragments of 150 to 3,000 bp mapping at least one complete probe and containing at least one BstUI or HpaII restriction site (*n* = 32,171) were selected. In total, 195,625 of the 244,000 array probes (80.2%) mapped to such informative fragments, mostly with 1 or 2 probes per fragment, up to 33. For statistical analysis and visualization, the median log ratio per fragment was used to represent the fragment. Methylation differences between tumor and normal samples and tumor subgroups were analyzed using a linear model in limma v3.2.1 [32]. The obtained *P* values were corrected for multiple testing [35] and fragments with a false discovery rate ≤0.01 were selected as significantly differentially methylated regions.

### *MLH1* and CIMP marker methylation

DNA samples (500 ng) were bisulfite converted using the EZ DNA methylation Gold kit (#D5006 Zymo Research). For validation of methylation changes, we performed a methylation-specific PCR on the *MLH1* promoter using primers previously described (Additional file 3) [36]. Methylation of previously described CIMP markers: MINT1, MINT2, MINT12, MINT31, *PRDM2*/*RIZ1*, and *TIMP3* were determined by methylation-specific PCR, while MINT27 and *LRP2*/megalin methylation were determined by Combined Bisulfite Restriction Analysis [6,37]. Using the criteria suggested by Shen *et al*. [6,37] including these methylation markers and mutation status of *BRAF*, *KRAS*, and *TP53*, tumors were determined to be CIMP-positive when two or more CIMP1 markers (*BRAF* mutation, methylated *MLH1*, *TIMP3*, MINT1, *PRDM2*/*RIZ1*), or three or more CIMP2 markers (*KRAS* mutation, methylated MINT27, MINT2, MINT31, *LRP2*/megalin) were present. Tumors were called CIMP-negative when two or more CIMP-negative markers (*TP53* mutation, unmethylated MINT27, MINT2, MINT31, MINT1) were present. This CIMP marker set was previously validated with the CIMP loci proposed by Weisenberger *et al*. [8,38]. Amplifications were carried out in a DNA Engine Dyad Peltier Thermal Cycler (Bio-Rad) using AmpliTaq Gold PCR buffer and enzyme (#4317742 Invitrogen). Amplified bands were visualized on a 2% agarose gel.

### Bisulfite sequencing analysis

Bisulfite conversion was performed on 200 ng of DNA using the EZ DNA methylation Gold kit and eluted in 15 μl Milli-Q purified water. The PCR amplification was performed using primers designed using MethPrimer (Additional file 5) [39]. The PCR reaction mixture contained 1× iQ SYBR green supermix (#170-8884, Bio-Rad), 1 μl of the bisulfite-converted DNA and 5 nmol of forward and reverse primer. The PCR products were purified using the MinElute® 96 UF PCR Purification kit (#28051, Qiagen) and sent for sequencing at Macrogen (Macrogen, Europe). Sequence alignment and quantification of methylation was performed using ESME (Epigenomics Inc.) [40]. Statistical significance of hypermethylation in *BRAF*^*V600E*^ tumors was determined using a one-sided Mann–Whitney test.

### Gene expression analysis

Gene expression of *FOXB2*, *FOXD3*, and *FOXF1* was determined in nine pairs of tumor and normal tissue of which RNA was available. cDNA was synthesized using 1 to 2 μg RNA, 50 ng oligo-dT, 1.6 μg random primer, 1 mM dNTPs, 5U AMV-RT transcriptase, and 10 U RNasin (#M5108 and #N2615, Promega). Gene expression was determined using TaqMan Gene Expression Assays (Applied Biosystems). Gene expression was normalized with *CPSF6* and *HNRNP*[41], which were amplified using 0.8 pmol forward and reverse primer in a 1× iQ SYBR green supermix (#170-8884, Bio-Rad). For *FOXB1*, *FOXB2*, and *FOXD3*, 2 μl of 125× diluted cDNA was amplified in a mix containing 1× iQ supermix (#170-8862, Bio-Rad) and 1× TaqMan assay (#Hs00247213_s1, #Hs02386300_s1, Hs00255287_s1, Hs00230962_m1, Applied Biosystems). All qPCRs were performed in duplicate.

### Exploratory data analysis

Differentially methylated regions were compared with publicly available data containing chromosomal regions identified in chromatin immunoprecipitation using antibodies against H3K27me^3^, H3K4me^3^, CTCF, and SUZ12 in ES cells followed by high-throughput sequencing [42-44]. By using the sqldf R package (version 0.3-5), we determined overlap of at least 20 bp between CpG regions represented on the array and these regions. Enrichment of chromatin domains among the differentially methylated regions was calculated by χ-squared test. Functional annotation clustering was performed in Panther 6.0 [45]. Filtering of the differential methylation datasets by H3K27me^3^ in ES cells using the dataset from Zhao *et al*. [44] was performed in R using the sqldf package.

## Abbreviations

BSA: Bisulfite sequencing analysis; CIMP: CpG island methylator phenotype; DMH: Differential methylation hybridization; ES cells: Embryonic stem cells; FOX: Forkhead box; H3K27me^3^: Histone H3 lysine 27 trimethylation inactivation mark; MSI: Microsatellite instability; PCR: Polymerase chain reaction; RT-qPCR: Real-time reverse-transcription PCR.

## Competing interests

The authors declare that they have no competing interests.

## Authors’ contributions

EHJvR carried out the methylation profiling study and data analysis, and drafted part of the manuscript. AB carried out the validation experiments and drafted part of the manuscript. AAD and RFE participated in data analysis. TvW participated in coordination, and interpretation of the data. HM conceived of the study and participated in its design. JMB participated in study design, and carried out array data analysis and critical revision of the manuscript. All authors read and approved the final manuscript.

## Supplementary Material

Additional file 1 Regions with tumor-specific methylation changes (UCSC assembly: March 2006, NCBI36/hg18). Click here for file

Additional file 2 Regions with CIMP-specific methylation changes (UCSC assembly: March 2006, NCBI36/hg18). Click here for file

Additional file 3 Sample information. Click here for file

Additional file 4 **Regions with *BRAF *mutation-specific methylation changes (UCSC assembly: March 2006, NCBI36/hg18).** Click here for file

Additional file 5 Primers used in this study. Click here for file
