# Supplementary material for: BRAF mutation-specific promoter methylation of FOX genes in colorectal cancer
Source: Clin Epigenetics. 2013 Jan 16;5(1):2. doi: 10.1186/1868-7083-5-2 (PMC3599401; doi:10.1186/1868-7083-5-2)
Supplement: Additional file 1 — Regions with tumor-specific methylation changes (UCSC assembly: March 2006, NCBI36/hg18). [file 1868-7083-5-2-S1.pdf]

Gene Name	Description	UniGene	EntrezGene	Chrom	Start	End	Length	BstUI sites	HpaII sites	Probes	logFC	adj.P.val	H3K27me3 bound
FLJ44006-BCL2L11	DIVERGENT_PROMOTER		NA	2	111592583	111593558	976	13	9	11	0.166172285	0.003269553	yes
ALKBH1	DIVERGENT_PROMOTER	Hs.94542	8846	14	77243572	77244382	811	4	8	6	-0.065586576	0.002273691	no
COL4A2	DIVERGENT_PROMOTER	Hs.508716	1284	13	109757069	109758722	1654	22	22	19	0.08736973	0.003850983	no
EVX2-HOXD13	DIVERGENT_PROMOTER		NA	2	176658363	176658723	361	4	1	3	0.360700288	6.85E-06	no
EVX2-HOXD13	DIVERGENT_PROMOTER		NA	2	176664734	176665031	298	1	3	3	0.280404464	0.000572648	no
EVX2-HOXD13	DIVERGENT_PROMOTER		NA	2	176657563	176657987	425	2	3	2	0.161440016	0.005928752	no
GAPDHS	DIVERGENT_PROMOTER	Hs.248017	26330	19	40715999	40716876	878	5	3	8	0.13035318	0.004507238	no
HSPA1A	DIVERGENT_PROMOTER	Hs.520028	3303	6	31890965	31893632	2668	9	16	4	0.185705382	0.002692155	no
NUP62CL	DIVERGENT_PROMOTER	Hs.163629	54830	X	106335655	106336325	671	6	8	5	-0.103470027	0.001626604	no
PSMD13	DIVERGENT_PROMOTER	Hs.134688	5719	11	226644	227403	760	4	14	6	-0.066119052	0.009137417	no
TIGD7-ZNF75A	DIVERGENT_PROMOTER		NA	16	3294682	3295370	689	5	6	3	0.220431188	0.000518806	no
XPNPEP3	DIVERGENT_PROMOTER	Hs.529163	63929	22	39582772	39583493	722	6	5	5	-0.071902972	0.007239439	no
ZIK1	DIVERGENT_PROMOTER	Hs.454830	284307	19	62787068	62788591	1524	4	2	6	0.199986229	1.13E-05	no
AATF	PROMOTER	Hs.195740	26574	17	32374596	32375343	748	3	4	5	0.100885224	0.005355829	yes
ABCC9	PROMOTER	Hs.446050	10060	12	21985681	21986126	446	1	3	3	0.251683141	0.003166562	yes
ACCN1	PROMOTER	Hs.368417	40	17	29508097	29509450	1354	2	5	4	0.114137407	0.002899612	yes
ADAMTS5	PROMOTER	Hs.58324	11096	21	27261368	27261564	197	1	1	1	0.469359838	0.000531451	yes
ADCYAP1	PROMOTER	Hs.531719	116	18	894424	894713	290	2	2	2	0.250012442	0.003016494	yes
ADRA1A	PROMOTER	Hs.654357	148	8	26779409	26779884	476	0	5	4	0.122699408	0.003475427	yes
ALOX12	PROMOTER	Hs.654431	239	17	6839212	6840063	852	2	7	8	0.214038014	6.81E-05	yes
ALX4	PROMOTER	Hs.436055	60529	11	44288848	44289037	190	2	0	1	0.207680451	0.004730551	yes
BDNF	PROMOTER	Hs.502182	627	11	27699715	27700174	460	0	2	2	0.18103538	0.001558127	yes
BDNF	PROMOTER	Hs.502182	627	11	27700616	27701448	833	5	5	6	0.263313577	0.003780535	yes
BHLHB5	PROMOTER	Hs.388788	27319	8	65653629	65654063	435	0	3	2	0.281969944	0.005185371	yes
BRUNOL4	PROMOTER	Hs.435976	56853	18	33401442	33401693	252	2	1	2	0.160723867	0.001101399	yes
C14orf39	PROMOTER	Hs.335754	317761	14	60021902	60022793	892	8	5	9	0.122516214	0.000241071	yes
C20orf103	PROMOTER	Hs.22920	24141	20	9434659	9435475	817	3	5	5	0.176073669	0.000645655	yes
CBLN4	PROMOTER	Hs.126141	140689	20	54013579	54014129	551	10	4	5	0.347913085	6.85E-06	yes
CBLN4	PROMOTER	Hs.126141	140689	20	54012850	54013578	729	7	4	6	0.256452944	0.001033655	yes
CDH4	PROMOTER	Hs.473231	1002	20	59259950	59260612	663	6	4	3	0.36238725	0.003593592	yes
CGB7	PROMOTER	Hs.681647	94027	19	54251073	54251226	154	2	0	1	0.108574393	0.009802959	yes
CIDEA	PROMOTER	Hs.249129	1149	18	12243999	12244330	332	5	2	3	0.538161292	1.35E-05	yes
CIDEA	PROMOTER	Hs.249129	1149	18	12244331	12245122	792	12	5	9	0.203692934	0.001617123	yes
COL12A1	PROMOTER	Hs.101302	1303	6	75973024	75973179	156	4	1	1	0.30692884	0.000467016	yes
COL12A1	PROMOTER	Hs.101302	1303	6	75972516	75972803	288	1	2	2	0.454805488	0.002371094	yes
CYP26A1	PROMOTER	Hs.150595	1592	10	94817946	94819100	1155	10	6	12	0.187750657	0.000732582	yes
CYP26C1	PROMOTER	Hs.369993	340665	10	94809439	94810450	1012	5	6	7	0.11310868	0.001229742	yes
DBX1	PROMOTER	Hs.558604	120237	11	20141512	20142124	613	2	6	6	0.109373583	0.009719989	yes
DBX2	PROMOTER	Hs.302764	440097	12	43731177	43731817	641	4	2	6	0.213228342	0.000475147	yes
DLX4	PROMOTER	Hs.591167	1748	17	45397357	45398423	1067	5	7	9	0.105675329	0.001902772	yes
DMRT1	PROMOTER	Hs.98586	1761	9	831205	833027	1823	11	23	16	0.066134828	0.007078089	yes
DMRT2	PROMOTER	Hs.59506	10655	9	1035005	1036115	1111	6	9	6	0.345127143	0.000179092	yes
DMRT3	PROMOTER	Hs.189174	58524	9	961538	961910	373	6	2	3	0.434651979	0.000163975	yes
DMRT3	PROMOTER	Hs.189174	58524	9	960455	961468	1014	8	6	6	0.112406827	0.002029968	yes
DMRT3	PROMOTER	Hs.189174	58524	9	962604	963858	1255	7	7	8	0.067413938	0.003525426	yes
DMRT3	PROMOTER	Hs.189174	58524	9	959793	960443	651	4	4	5	0.138957441	0.003754112	yes
DMRT3	PROMOTER	Hs.189174	58524	9	965963	966676	714	6	5	5	0.118858297	0.003957164	yes
EBF2	PROMOTER	Hs.491292	64641	8	25964811	25965597	787	6	0	4	0.293440994	0.002348991	yes
EBF2	PROMOTER	Hs.491292	64641	8	25961458	25961791	334	4	3	3	0.113375548	0.003516843	yes
EBF2	PROMOTER	Hs.491292	64641	8	25961010	25961457	448	4	1	4	0.324054181	0.006001521	yes
EBF3	PROMOTER	Hs.699395	253738	10	131657800	131658301	502	3	4	4	0.129337095	0.001246631	yes
EFCBP1	PROMOTER	Hs.560892	64168	8	91872667	91873544	878	8	6	7	0.238051686	0.000372492	yes
EN1	PROMOTER	Hs.271977	2019	2	119330550	119331405	856	2	5	8	0.179719349	7.87E-05	yes
EN1	PROMOTER	Hs.271977	2019	2	119331416	119332085	670	6	8	7	0.322036499	0.000168981	yes
EN1	PROMOTER	Hs.271977	2019	2	119323809	119323960	152	2	0	1	0.246691152	0.000215845	yes
EOMES	PROMOTER	Hs.591663	8320	3	27740317	27740564	248	1	2	2	0.47812104	5.10E-06	yes
EOMES	PROMOTER	Hs.591663	8320	3	27746374	27747220	847	4	2	5	0.218002701	0.000819303	yes
EOMES	PROMOTER	Hs.591663	8320	3	27740565	27741240	676	6	0	2	0.225400041	0.008178457	yes
ERICH1	PROMOTER	Hs.655310	157697	8	679088	679363	276	2	5	2	0.099318247	0.004380752	yes
ERICH1	PROMOTER	Hs.655310	157697	8	679364	679640	277	1	3	2	0.130288324	0.00938857	yes
FAM123A	PROMOTER	Hs.528335	219287	13	24643568	24644840	1273	8	7	7	0.182942902	0.003019475	yes
FEZF2	PROMOTER	Hs.241523	55079	3	62334225	62334656	432	6	3	4	0.390864089	0.000237189	yes
FEZF2	PROMOTER	Hs.241523	55079	3	62333962	62334224	263	3	2	1	0.546106009	0.002191639	yes
FGF9	PROMOTER	Hs.111	2254	13	21141014	21141328	315	0	1	1	0.265494533	0.001229742	yes
FLI1	PROMOTER	Hs.504281	2313	11	128068668	128069169	502	2	2	3	0.168043612	0.001045681	yes
FLI1	PROMOTER	Hs.504281	2313	11	128067857	128068529	673	8	11	6	0.08919443	0.003352734	yes
FLJ46831	PROMOTER		NA	10	129424252	129425408	1157	9	12	11	0.114777937	0.000566055	yes
FLRT2	PROMOTER	Hs.533710	23768	14	85066158	85066751	594	4	2	6	0.169821559	0.003016494	yes
FLT3	PROMOTER	Hs.507590	2322	13	27572738	27573180	443	4	4	4	0.20874013	0.003163171	yes
FOXA1	PROMOTER	Hs.163484	3169	14	37137269	37137912	644	6	5	6	0.196883326	0.001355437	yes
FOXC1	PROMOTER	Hs.348883	2296	6	1553532	1553683	152	0	2	1	0.139032017	0.008080187	yes
FOXC2	PROMOTER	Hs.436448	2303	16	85156512	85157261	750	8	5	6	0.211613077	3.56E-05	yes
FOXF2	PROMOTER	Hs.484423	2295	6	1329787	1330721	935	3	6	5	0.167528883	0.003510606	yes
FUSSEL18	PROMOTER		NA	18	43031520	43032101	582	3	6	6	0.226428193	0.002837492	yes
GAS2	PROMOTER		NA	11	22645148	22645365	218	1	1	1	0.548480902	0.000165128	yes
GDNF	PROMOTER	Hs.248114	2668	5	37874056	37874658	603	4	3	5	0.408957032	4.08E-05	yes
GDNF	PROMOTER	Hs.248114	2668	5	37872231	37872600	370	4	3	2	0.36420114	0.000181946	yes
GDNF	PROMOTER	Hs.248114	2668	5	37874659	37875401	743	1	8	8	0.157299105	0.001240127	yes
GHSR	PROMOTER	Hs.248115	2693	3	173650435	173650650	216	0	1	2	0.371900418	0.000102079	yes
GJA9	PROMOTER	Hs.632402	81025	15	32833309	32834091	783	2	7	5	0.205143681	3.59E-06	yes
GJA9	PROMOTER	Hs.632402	81025	15	32834146	32834514	369	6	4	3	0.178164667	2.13E-05	yes
GRID1	PROMOTER	Hs.530653	2894	10	88117365	88117638	274	1	1	1	0.12073618	0.003385783	yes
GRM7	PROMOTER	Hs.606393	2917	3	6877772	6878645	874	10	11	8	0.291314582	0.000505078	yes
GSC	PROMOTER	Hs.440438	145258	14	94308861	94309566	706	3	4	6	0.211210692	0.001510128	yes
HMX2	PROMOTER	Hs.444756	3167	10	124895449	124895824	376	4	2	3	0.538986867	1.15E-05	yes
HMX2	PROMOTER	Hs.444756	3167	10	124891220	124892258	1039	6	7	3	0.286072798	4.08E-05	yes
HMX2	PROMOTER	Hs.444756	3167	10	124892259	124893350	1092	4	8	5	0.147130649	0.001250274	yes
HMX2	PROMOTER	Hs.444756	3167	10	124896127	124896433	307	1	1	1	0.330823314	0.003952252	yes
HOXC11	PROMOTER	Hs.127562	3227	12	52646481	52647136	656	1	4	6	0.2070643	4.77E-05	yes

HOXC11	PROMOTER	Hs.127562	3227	12	52652742	52653297	556	1	3	2	0.294227519	0.000217943	yes
HOXC11	PROMOTER	Hs.127562	3227	12	52646113	52646448	336	0	4	1	0.393609471	0.000285683	yes
HOXC11	PROMOTER	Hs.127562	3227	12	52645223	52646112	890	1	4	2	0.312470157	0.003571467	yes
HOXC12	PROMOTER	Hs.381267	3228	12	52631728	52631920	193	0	1	1	0.729821788	3.36E-05	yes
HOXC9	PROMOTER	Hs.658823	3225	12	52679650	52679855	206	2	0	2	0.320512871	0.004496872	yes
HS3ST2	PROMOTER	Hs.115830	9956	16	22731628	22732955	1328	12	8	10	0.131838779	5.42E-05	yes
HS3ST2	PROMOTER	Hs.115830	9956	16	22732956	22734005	1050	15	17	9	0.097595053	0.000148213	yes
hsa-mir-124a-2	PROMOTER		NA	8	65452502	65453612	1111	8	6	11	0.163564301	0.000122213	yes
hsa-mir-129-2	PROMOTER		NA	11	43558871	43559803	933	8	7	8	0.073302772	0.004672111	yes
hsa-mir-9-3	PROMOTER		NA	15	87711502	87713185	1684	9	16	19	0.044887618	0.009520923	yes
INSM1	PROMOTER	Hs.89584	3642	20	20293917	20294324	408	3	3	3	0.376724887	7.66E-05	yes
IRX1	PROMOTER	Hs.424156	79192	5	3643426	3643914	489	3	2	4	0.17647296	3.37E-05	yes
IRX1	PROMOTER	Hs.424156	79192	5	3644733	3645075	343	5	2	2	0.279953828	0.000692507	yes
IRX1	PROMOTER	Hs.424156	79192	5	3645292	3646037	746	3	7	6	0.079461249	0.001784107	yes
IRX1	PROMOTER	Hs.424156	79192	5	3644102	3644732	631	3	4	7	0.178892106	0.004159485	yes
IRX3	PROMOTER	Hs.499205	79191	16	52879869	52880117	249	1	1	2	0.891881846	6.17E-08	yes
IRX3	PROMOTER	Hs.499205	79191	16	52880602	52881169	568	7	5	5	0.238700365	0.000124696	yes
IRX3	PROMOTER	Hs.499205	79191	16	52880184	52880588	405	2	3	4	0.214308917	0.000187609	yes
IRX3	PROMOTER	Hs.499205	79191	16	52881544	52882349	806	8	7	7	0.163736571	0.001043223	yes
IRX3	PROMOTER	Hs.499205	79191	16	52881170	52881466	297	0	1	1	0.186707223	0.008545802	yes
IRX4	PROMOTER	Hs.196927	50805	5	1936549	1936815	267	2	4	2	0.518832372	1.51E-06	yes
IRX4	PROMOTER	Hs.196927	50805	5	1935963	1936324	362	4	7	3	0.409422545	3.84E-06	yes
IRX4	PROMOTER	Hs.196927	50805	5	1936816	1937434	619	2	11	7	0.079180067	0.003352024	yes
ISLR2	PROMOTER	Hs.254775	57611	15	72209856	72210394	539	5	1	3	0.418557962	0.001950518	yes
ISLR2	PROMOTER	Hs.254775	57611	15	72208754	72209497	744	5	6	7	0.090864863	0.005167708	yes
ITGA8	PROMOTER	Hs.171311	8516	10	15801268	15802349	1082	10	12	10	0.119013237	0.002425972	yes
KCNH2	PROMOTER	Hs.647099	3757	7	150306882	150308825	1944	2	10	7	-0.070295855	0.008804782	yes
LBX1	PROMOTER	Hs.37128	10660	10	102985980	102986278	299	3	4	3	0.399158495	0.000251282	yes
LBXCOR1	PROMOTER	Hs.451224	390598	15	65900334	65900788	455	3	5	3	0.401740048	0.003754112	yes
LHX1	PROMOTER	Hs.443727	3975	17	32368397	32369050	654	7	2	7	0.183562517	0.000762701	yes
LOC643923	PROMOTER	Hs.647220	643923	11	106967578	106967989	412	2	4	5	0.279360652	7.00E-05	yes
LOC644168	PROMOTER		NA	10	50274743	50274896	154	1	1	1	0.460490392	0.000154518	yes
LOC644168	PROMOTER		NA	10	50274976	50275228	253	0	1	2	0.223710403	0.00274541	yes
MAFB	PROMOTER	Hs.702085	9935	20	38752718	38752968	251	7	4	2	0.224282061	5.07E-05	yes
MAFB	PROMOTER	Hs.702085	9935	20	38753132	38753609	478	2	2	3	0.233630593	0.000493318	yes
MAFB	PROMOTER	Hs.702085	9935	20	38752179	38752717	539	5	7	6	0.059447011	0.002969754	yes
MAGI2	PROMOTER	Hs.654788	9863	7	78921634	78921918	285	1	1	2	0.155261768	0.001366159	yes
MGC39900	PROMOTER	Hs.675540	286527	X	103103499	103104231	733	3	6	7	0.062277119	0.004751215	yes
MKX	PROMOTER	Hs.128193	283078	10	28074434	28074945	512	9	9	5	0.309649824	0.001144224	yes
MKX	PROMOTER	Hs.128193	283078	10	28074946	28075782	837	5	9	3	0.296658418	0.007767557	yes
MPPED2	PROMOTER	Hs.289795	744	11	30562409	30563005	597	2	5	4	0.179475902	0.006420096	yes
NGB	PROMOTER	Hs.274363	58157	14	76807330	76807844	515	2	1	2	0.424607227	4.76E-06	yes
NKX2-2	PROMOTER	Hs.516922	4821	20	21442865	21443015	151	1	0	1	0.453432729	0.001213111	yes
NKX2-2	PROMOTER	Hs.516922	4821	20	21443206	21443424	219	1	4	2	0.243305776	0.002147667	yes
NKX2-2	PROMOTER	Hs.516922	4821	20	21443470	21444094	625	4	5	7	0.076833646	0.008195482	yes
NKX2-2	PROMOTER	Hs.516922	4821	20	21444224	21444876	653	5	4	7	0.115039657	0.009633158	yes
NKX2-3	PROMOTER	Hs.243272	159296	10	101280335	101280811	477	0	1	3	0.230366217	0.00074159	yes
NKX2-8	PROMOTER	Hs.234763	26257	14	36121994	36123171	1178	5	9	11	0.137131195	0.009133785	yes
NMBR	PROMOTER	Hs.654478	4829	6	142451732	142452122	391	1	2	2	0.106200878	0.001377412	yes
NPBWR1	PROMOTER	Hs.248117	2831	8	54014193	54014806	614	6	3	5	0.196030924	0.000152543	yes
NPR3	PROMOTER	Hs.237028	4883	5	32747157	32747438	282	2	2	3	0.217367573	0.003984654	yes
NR2E1	PROMOTER	Hs.157688	7101	6	108593040	108593807	768	7	4	7	0.316843642	2.46E-05	yes
NR2E1	PROMOTER	Hs.157688	7101	6	108593824	108594459	636	3	4	6	0.33710776	0.000475019	yes
NRG3	PROMOTER	Hs.125119	10718	10	83623687	83623998	312	0	1	3	0.193141346	0.001094436	yes
OLIG2	PROMOTER	Hs.176977	10215	21	33318899	33319220	322	2	1	3	0.268179093	0.000540154	yes
OLIG2	PROMOTER	Hs.176977	10215	21	33318267	33318498	232	1	0	3	0.354820268	0.004792654	yes
OLIG3	PROMOTER	Hs.195398	167826	6	137859692	137860262	571	1	5	5	0.163911981	0.001819565	yes
ONECUT1	PROMOTER	Hs.658573	3175	15	50870384	50870564	181	0	1	2	0.780390034	7.86E-08	yes
ONECUT1	PROMOTER	Hs.658573	3175	15	50874398	50874819	422	2	3	4	0.228774267	0.00014805	yes
OTP	PROMOTER	Hs.202247	23440	5	76970321	76971099	779	5	6	9	0.15258139	2.73E-05	yes
OTX2	PROMOTER	Hs.288655	5015	14	56348799	56349011	213	0	1	1	0.410068982	6.85E-06	yes
OTX2	PROMOTER	Hs.288655	5015	14	56344770	56345011	242	3	2	2	0.272168757	0.001718281	yes
OTX2	PROMOTER	Hs.288655	5015	14	56345012	56345890	879	9	7	8	0.112084944	0.001920368	yes
PAX1	PROMOTER	Hs.349082	5075	20	21631084	21632447	1364	8	8	8	0.133447844	0.000152595	yes
PAX2	PROMOTER	Hs.155644	5076	10	102487540	102488033	494	1	3	2	0.534819027	0.000379072	yes
PAX2	PROMOTER	Hs.155644	5076	10	102486611	102487539	929	1	5	5	0.165187106	0.001895711	yes
PAX6	PROMOTER	Hs.591993	5080	11	31797663	31798044	382	0	1	3	0.348943272	1.05E-05	yes
PAX6	PROMOTER	Hs.591993	5080	11	31791919	31792134	216	1	1	2	-0.094072241	0.006253405	yes
PAX9	PROMOTER	Hs.132576	5083	14	36196496	36197382	887	5	5	9	0.134606878	0.000453355	yes
PAX9	PROMOTER	Hs.132576	5083	14	36196266	36196495	230	2	1	1	0.326347909	0.008994031	yes
PCDHGC4	PROMOTER		NA	5	140844663	140847537	2875	6	7	4	0.131176878	0.001578765	yes
PCSK2	PROMOTER	Hs.315186	5126	20	17154635	17155100	466	2	6	5	0.109471748	0.00130735	yes
PENK	PROMOTER	Hs.339831	5179	8	57520661	57521760	1100	15	13	12	0.279213723	0.000225088	yes
PHOX2A	PROMOTER	Hs.697462	401	11	71632895	71633214	320	4	3	4	0.105256199	0.008080187	yes
POU4F1	PROMOTER	Hs.654522	5457	13	78079732	78080327	596	2	7	4	0.415175433	2.20E-06	yes
POU4F1	PROMOTER	Hs.654522	5457	13	78079083	78079731	649	1	5	5	0.107925391	0.006109377	yes
POU4F1	PROMOTER	Hs.654522	5457	13	78081800	78082006	207	0	2	2	0.157234009	0.007980063	yes
RASGRF1	PROMOTER	Hs.591111	5923	15	77168737	77170415	1679	18	11	17	0.12045155	0.000425022	yes
RAX	PROMOTER	Hs.278957	30062	18	55091826	55092840	1015	5	7	10	0.13233253	9.21E-07	yes
SALL3	PROMOTER	Hs.669133	27164	18	74838450	74838700	251	1	2	1	0.323172304	0.000245173	yes
SALL3	PROMOTER	Hs.669133	27164	18	74837981	74838449	469	2	5	4	0.223933186	0.002938515	yes
SCN4B	PROMOTER	Hs.65239	6330	11	117529253	117529999	747	2	4	2	0.167841646	0.001949183	yes
SFMBT2	PROMOTER	Hs.407983	57713	10	7493998	7494641	644	3	3	7	0.430465026	1.57E-06	yes
SFMBT2	PROMOTER	Hs.407983	57713	10	7493645	7493997	353	1	5	3	0.453254949	0.000139609	yes
SIM1	PROMOTER	Hs.520293	6492	6	101018662	101018945	284	2	1	2	0.701544106	3.73E-06	yes
SIM1	PROMOTER	Hs.520293	6492	6	101023875	101024099	225	1	1	1	0.257715805	1.37E-05	yes
SIM1	PROMOTER	Hs.520293	6492	6	101022125	101022981	857	2	5	5	0.230426738	0.000713433	yes
SIM1	PROMOTER	Hs.520293	6492	6	101019518	101020097	580	8	8	6	0.098462348	0.004143249	yes
SIM1	PROMOTER	Hs.520293	6492	6	101024100	101024392	293	2	3	3	0.210352384	0.005361988	yes
SIM2	PROMOTER	Hs.146186	6493	21	36986757	36987552	796	9	4	6	0.271254574	4.15E-06	yes

SIM2	PROMOTER	Hs.146186	6493	21	36989964	36991371	1408	10	9	14	0.126926581	0.000460616	yes
SIM2	PROMOTER	Hs.146186	6493	21	36991372	36992504	1133	8	9	11	0.115196183	0.000732448	yes
SIX2	PROMOTER	Hs.101937	10736	2	45091004	45091309	306	0	7	3	0.196452228	0.005653974	yes
SIX6	PROMOTER	Hs.194756	4990	14	60044976	60046570	1595	10	10	12	0.080729308	0.00407876	yes
SLC6A5	PROMOTER	Hs.136557	9152	11	20574947	20575937	991	3	8	10	0.092653243	0.004049806	yes
SLC8A3	PROMOTER	Hs.337696	6547	14	69725456	69725874	419	7	4	3	0.277348759	0.000377139	yes
SLFN13	PROMOTER	Hs.462833	146857	17	30799341	30800832	1492	9	11	9	0.072728915	0.002013809	yes
SNAP91	PROMOTER	Hs.368046	9892	6	84475684	84476053	370	3	4	4	0.234640854	2.88E-05	yes
SOX1	PROMOTER	Hs.202526	6656	13	111763154	111763633	480	3	4	4	0.301782293	8.05E-06	yes
SOX1	PROMOTER	Hs.202526	6656	13	111763942	111764342	401	1	4	4	0.475183582	0.000267564	yes
SOX1	PROMOTER	Hs.202526	6656	13	111763634	111763941	308	1	3	3	0.142716053	0.003400042	yes
SOX1	PROMOTER	Hs.202526	6656	13	111760495	111760868	374	0	1	2	0.256373851	0.00445448	yes
SOX17	PROMOTER	Hs.98367	64321	8	55529731	55530085	355	0	4	4	0.661112277	9.12E-08	yes
SOX17	PROMOTER	Hs.98367	64321	8	55529223	55529730	508	6	5	5	0.308431087	0.000180777	yes
SOX17	PROMOTER	Hs.98367	64321	8	55530086	55530933	848	4	2	1	0.253518406	0.000599954	yes
SOX17	PROMOTER	Hs.98367	64321	8	55528496	55529222	727	5	7	6	0.181681548	0.002785327	yes
SOX7	PROMOTER		NA	8	10625902	10626588	687	4	8	6	0.2163259	0.000102966	yes
SOX7	PROMOTER		NA	8	10627901	10628543	643	11	7	6	0.243790814	0.005832848	yes
SPAG6	PROMOTER	Hs.655170	9576	10	22673979	22675583	1605	13	11	11	0.104351077	8.73E-05	yes
SPAG6	PROMOTER	Hs.655170	9576	10	22664502	22666148	1647	21	13	15	0.095829654	0.001169085	yes
SYCP2	PROMOTER	Hs.202676	10388	20	57942765	57942981	217	2	0	2	0.299706826	0.007216985	yes
SYT10	PROMOTER	Hs.118703	341359	12	33483002	33484463	1462	19	15	15	0.061283229	0.008009588	yes
TBX5	PROMOTER	Hs.381715	6910	12	113336362	113337196	835	2	8	4	0.142649014	9.03E-05	yes
TFAP2A	PROMOTER	Hs.519880	7020	6	10530145	10530906	762	3	2	6	0.116280662	0.000423693	yes
TITF1	PROMOTER		NA	14	36059857	36060200	344	4	3	3	0.412115294	0.003163171	yes
TITF1	PROMOTER		NA	14	36062561	36063788	1228	5	4	7	0.143480572	0.006836416	yes
TLX3	PROMOTER	Hs.249125	30012	5	170668670	170669045	376	3	2	4	0.337904539	7.81E-05	yes
TSPAN11	PROMOTER	Hs.505141	441631	12	30969964	30970660	697	2	3	3	0.18343136	0.000219858	yes
VAX1	PROMOTER	Hs.681703	11023	10	118889083	118889770	688	4	5	6	0.115060166	0.000805227	yes
VAX1	PROMOTER	Hs.681703	11023	10	118889771	118890344	574	3	6	6	0.087987958	0.009386372	yes
VGCNL1	PROMOTER		NA	13	100866948	100867193	246	1	2	2	0.380702716	6.39E-05	yes
VSX1	PROMOTER	Hs.274264	30813	20	25011537	25012111	575	5	3	4	0.262543966	4.79E-05	yes
VSX1	PROMOTER	Hs.274264	30813	20	25012112	25012406	295	1	1	2	0.439927921	0.000122213	yes
VSX1	PROMOTER	Hs.274264	30813	20	25012407	25012754	348	3	2	3	0.25969084	0.001183679	yes
VSX1	PROMOTER	Hs.274264	30813	20	25012792	25013559	768	5	6	7	0.218178821	0.002883096	yes
VSX1	PROMOTER	Hs.274264	30813	20	25009205	25011185	1981	15	16	14	0.067698645	0.003239258	yes
WIF1	PROMOTER	Hs.284122	11197	12	63801000	63802061	1062	11	7	10	0.159342136	0.000879791	yes
ZIC5	PROMOTER	Hs.508570	85416	13	99421973	99422946	974	8	2	5	0.182896565	0.000159091	yes
ZNF503	PROMOTER	Hs.195710	84858	10	76835789	76836616	828	3	5	8	-0.103423246	0.008543428	yes
ABCC9	PROMOTER	Hs.446050	10060	12	21985364	21985680	317	5	3	3	0.138073393	0.004533538	no
ABHD9	PROMOTER	Hs.156457	79852	19	15204759	15205958	1200	3	7	6	0.126742606	0.000203468	no
ADAM12	PROMOTER	Hs.655388	8038	10	128066884	128067650	767	8	8	8	0.11546506	0.009861949	no
ADCY1	PROMOTER	Hs.192215	107	7	45579737	45580191	455	2	2	4	0.217872795	0.000267517	no
ADHFE1	PROMOTER	Hs.268869	137872	8	67507040	67507595	556	6	3	6	0.664139718	1.38E-08	no
ADRA1A	PROMOTER	Hs.654357	148	8	26779885	26780077	193	1	1	1	0.368025046	0.004197537	no
AKAP12	PROMOTER	Hs.371240	9590	6	151602399	151602676	278	1	3	2	0.638377447	9.44E-08	no
ALS2CR11	PROMOTER	Hs.335788	151254	2	202191969	202192506	538	2	2	3	0.201236418	0.000530942	no
ANPEP	PROMOTER	Hs.1239	290	15	88158019	88158537	519	2	2	2	0.225725758	0.006705347	no
AP3B2	PROMOTER	Hs.199593	8120	15	81175827	81176190	364	1	1	3	0.134422386	0.005087241	no
ARRDC2	PROMOTER	Hs.515249	27106	19	17979661	17981259	1599	18	12	10	0.073738618	0.007647519	no
ATP8B2	PROMOTER	Hs.435700	57198	1	152564453	152565820	1368	4	13	5	0.084749451	0.009628482	no
AURKC	PROMOTER	Hs.98338	6795	19	62433578	62435710	2133	6	6	6	-0.09058194	0.006142807	no
AVP	PROMOTER	Hs.89648	551	20	3021118	3021338	221	0	1	2	0.319802388	0.000315973	no
BAPX1	PROMOTER		NA	4	13157531	13158765	1235	17	16	10	0.083474013	0.004827202	no
BARHL1	PROMOTER	Hs.283809	56751	9	134447623	134448528	906	7	5	8	0.109771074	0.000884044	no
BARHL2	PROMOTER	Hs.451956	343472	1	90962974	90963317	344	2	1	3	0.271380019	3.10E-06	no
BARHL2	PROMOTER	Hs.451956	343472	1	90963768	90964279	512	2	3	5	0.35262133	7.41E-05	no
BARHL2	PROMOTER	Hs.451956	343472	1	90954912	90955447	536	3	2	4	0.278210831	0.000147781	no
BARHL2	PROMOTER	Hs.451956	343472	1	90961690	90961947	258	2	1	2	0.182755469	0.00544958	no
BARX1	PROMOTER	Hs.164960	56033	9	95761255	95761562	308	6	3	3	0.116182283	0.009223622	no
BBC3	PROMOTER	Hs.467020	27113	19	52434188	52434530	343	0	2	1	0.423817912	0.001592171	no
BIK	PROMOTER	Hs.475055	638	22	41836281	41837189	909	8	15	8	-0.065286729	0.006937792	no
BOLA3	PROMOTER	Hs.61472	388962	2	74227920	74229313	1394	13	12	8	0.047073966	0.009584342	no
BOLL	PROMOTER	Hs.169797	66037	2	198357914	198359486	1573	5	7	17	0.091282266	0.000464253	no
BRDT	PROMOTER	Hs.482520	676	1	92187315	92188202	888	2	5	4	-0.100400427	0.004557699	no
BSND	PROMOTER	Hs.151291	7809	1	55235119	55235665	547	4	5	4	0.102122898	0.004410423	no
C12orf42	PROMOTER	Hs.534649	374470	12	102413525	102414324	800	6	7	5	0.103461443	0.00035953	no
C13orf21	PROMOTER	Hs.377972	387923	13	43845503	43846608	1106	13	20	10	0.06862858	0.002010938	no
C17orf46	PROMOTER	Hs.412719	124783	17	40694552	40695829	1278	6	5	10	0.096778127	0.007223385	no
C19orf28	PROMOTER	Hs.656901	126321	19	3509122	3509531	410	2	1	2	-0.116564653	0.000535216	no
C1orf2	PROMOTER	Hs.348308	10712	1	153492174	153492423	250	1	2	2	-0.104688901	0.00417388	no
C1orf77	PROMOTER	Hs.611057	26097	1	151871751	151872909	1159	2	3	2	-0.12574992	0.000215343	no
C1QL1	PROMOTER	Hs.134012	10882	17	40402901	40403536	636	7	8	6	0.075282798	0.001248997	no
C6orf159	PROMOTER	Hs.149454	134701	6	84619600	84620326	727	14	8	8	0.147504499	0.008804782	no
C6orf173	PROMOTER	Hs.486401	387103	6	126702584	126702927	344	0	2	2	-0.142478258	0.006170301	no
C6orf65	PROMOTER	Hs.582993	221336	6	56926609	56926896	288	1	1	2	0.584813511	0.000157489	no
CABIN1	PROMOTER	Hs.517478	23523	22	22737235	22738393	1159	18	10	5	-0.089064389	0.001732497	no
CBX7	PROMOTER	Hs.356416	23492	22	37877691	37878873	1183	19	12	7	-0.067386421	0.005840067	no
CCDC13	PROMOTER		NA	3	42789439	42789907	469	7	6	3	0.129707633	0.009033014	no
CCDC8	PROMOTER	Hs.97876	83987	19	51608106	51609011	906	6	6	6	0.192491132	6.33E-05	no
CCK	PROMOTER	Hs.458426	885	3	42281828	42282638	811	4	5	7	0.131400813	2.85E-05	no
CD8A	PROMOTER	Hs.85258	925	2	86870715	86872498	1784	9	10	11	0.062585635	0.004556543	no
CDH3	PROMOTER	Hs.461074	1001	16	67234124	67234485	362	5	4	3	0.280935456	4.71E-06	no
CDH3	PROMOTER	Hs.461074	1001	16	67233861	67234088	228	2	3	1	0.360619711	6.85E-06	no
CDO1	PROMOTER	Hs.442378	1036	5	115180281	115180493	213	4	3	2	0.1397517	0.006001521	no
CDS1	PROMOTER	Hs.654899	1040	4	85722411	85722794	384	2	1	3	-0.129525807	0.002617589	no
CHGB	PROMOTER	Hs.516874	1114	20	5839677	5840125	449	0	1	4	0.153800469	0.00856983	no
CHRM2	PROMOTER	Hs.535891	1129	7	136204344	136206042	1699	10	10	18	0.089465663	0.004513148	no
CLGN	PROMOTER	Hs.86368	1047	4	141567349	141568604	1256	9	12	12	0.098237213	0.001949183	no
CNTNAP5	PROMOTER	Hs.660653	129684	2	124498381	124498830	450	3	3	2	0.13665625	0.006911973	no

COL14A1	PROMOTER	Hs.409662	7373	8	121205998	121206633	636	5	3	4	0.168432463	0.000418005	no
CPEB1	PROMOTER	Hs.547988	64506	15	81114077	81114697	621	2	6	7	0.293951928	0.000455933	no
CREBBP	PROMOTER	Hs.459759	1387	16	3871516	3871865	350	4	2	2	-0.306401231	0.00074159	no
CRHR2	PROMOTER	Hs.546246	1395	7	30687925	30689245	1321	16	11	12	0.076385291	0.002348991	no
CWF19L2	PROMOTER	Hs.212140	143884	11	106833536	106833802	267	0	1	2	-0.128891404	0.000464253	no
CXCL14	PROMOTER	Hs.483444	9547	5	134942646	134943258	613	2	4	3	0.188677436	0.006135148	no
CYP11A1	PROMOTER	Hs.303980	1583	15	72452385	72454292	1908	5	13	6	-0.056410737	0.008855037	no
DGKI	PROMOTER	Hs.242947	9162	7	137182817	137183008	192	0	1	1	0.225934408	0.001767134	no
DIP	PROMOTER	Hs.475150	23151	22	45399870	45402364	2495	5	9	9	-0.063866749	0.005230602	no
DLGAP2	PROMOTER	Hs.113287	9228	8	1427908	1429171	1264	4	5	10	-0.168202582	4.65E-05	no
DLGAP2	PROMOTER	Hs.113287	9228	8	1430581	1431418	838	0	7	2	-0.148766887	0.000219858	no
DLX1	PROMOTER	Hs.407015	1745	2	172653838	172654671	834	4	6	5	0.3080652	2.92E-06	no
DLX2	PROMOTER	Hs.419	1746	2	172680440	172681586	1147	6	6	5	0.202593358	2.51E-05	no
DNAH11	PROMOTER	Hs.655326	8701	7	21548932	21549845	914	11	8	9	0.129292752	0.008027732	no
DOK5	PROMOTER	Hs.656582	55816	20	52525098	52525906	809	7	6	7	0.108510728	0.00563761	no
DPP6	PROMOTER	Hs.490684	1804	7	153214903	153215077	175	4	1	2	0.448454945	5.76E-06	no
DPP6	PROMOTER	Hs.490684	1804	7	153213899	153214902	1004	10	5	7	0.135394571	0.001289933	no
DUXA	PROMOTER	Hs.585857	503835	19	62375570	62375789	220	1	4	1	0.11050984	0.004755817	no
DZIP1	PROMOTER	Hs.656580	22873	13	95094109	95095202	1094	10	13	13	0.103122764	0.00249054	no
EBF3	PROMOTER	Hs.699395	253738	10	131658666	131658866	201	2	0	1	0.246843324	0.005756017	no
EDNRB	PROMOTER	Hs.82002	1910	13	77391135	77391408	274	3	2	2	0.774779389	3.01E-06	no
EDNRB	PROMOTER	Hs.82002	1910	13	77390121	77391109	989	6	1	7	0.192791856	4.08E-05	no
EFEMP1	PROMOTER	Hs.76224	2202	2	56004046	56004851	806	9	5	7	0.152927936	0.001906164	no
EGFL6	PROMOTER	Hs.12844	25975	X	13497447	13498111	665	7	4	8	0.146733746	0.001205461	no
EID3	PROMOTER	Hs.659857	493861	12	103221423	103222038	616	7	9	5	0.357815408	2.62E-05	no
EMID2	PROMOTER	Hs.654854	136227	7	100792510	100792792	283	2	1	1	0.259421566	0.000829303	no
EN2	PROMOTER	Hs.134989	2020	7	154941879	154942134	256	0	1	3	0.109356255	0.004513148	no
EPHA5	PROMOTER	Hs.654492	2044	4	66217530	66218270	741	2	6	6	0.292311985	2.01E-05	no
EVX1	PROMOTER	Hs.369879	2128	7	27245737	27246049	313	2	8	3	0.163762287	0.002249551	no
EVX1	PROMOTER	Hs.369879	2128	7	27245425	27245736	312	3	4	3	0.161493481	0.008554205	no
EXOSC3	PROMOTER	Hs.591076	51010	9	37776687	37777680	994	10	5	7	0.14532108	0.007214219	no
FAM107A	PROMOTER	Hs.506357	11170	3	58547300	58548120	821	7	7	7	0.116622229	1.88E-05	no
FAT4	PROMOTER	Hs.702217	79633	4	126456942	126457744	803	5	4	8	0.174020626	0.000102966	no
FBXL7	PROMOTER	Hs.433057	23194	5	15552960	15553171	212	3	1	2	0.548423785	9.21E-07	no
FBXO39	PROMOTER	Hs.368364	162517	17	6619597	6620729	1133	5	8	8	0.17265874	0.000122213	no
FEZF1	PROMOTER	Hs.553970	389549	7	121737367	121737769	403	1	5	4	0.21271951	0.003352734	no
FGD5	PROMOTER	Hs.412406	152273	3	14826896	14827954	1059	19	23	7	0.063538578	0.004419038	no
FGF12	PROMOTER	Hs.584758	2257	3	193608348	193609308	961	2	8	9	0.162949685	0.0016054	no
FLJ21963	PROMOTER		NA	12	79995617	79996747	1131	3	11	8	0.180239377	0.005830762	no
FOXB1	PROMOTER	Hs.160375	27023	15	58079052	58079511	460	0	4	5	0.290552924	0.002953251	no
FOXD2	PROMOTER	Hs.166188	2306	1	47671836	47672469	634	5	7	6	0.264956042	0.001248591	no
FOXD3	PROMOTER	Hs.546573	27022	1	63557571	63558304	734	6	8	8	0.225830397	0.000132961	no
FOXD3	PROMOTER	Hs.546573	27022	1	63556634	63556828	195	0	2	1	0.440730949	0.000635057	no
FOXD3	PROMOTER	Hs.546573	27022	1	63559725	63559992	268	0	2	2	0.194518711	0.000994151	no
FOXD3	PROMOTER	Hs.546573	27022	1	63559266	63559697	432	2	2	2	0.762957783	0.001146248	no
FOXE1	PROMOTER	Hs.159234	2304	9	99653633	99654163	531	3	6	5	0.105210131	0.002837492	no
FOXE1	PROMOTER	Hs.159234	2304	9	99654535	99655302	768	1	8	3	0.113188728	0.007687259	no
FOXE1	PROMOTER	Hs.159234	2304	9	99654164	99654534	371	3	5	4	0.113170171	0.009566788	no
FTMT	PROMOTER	Hs.105324	94033	5	121215241	121216358	1118	5	12	9	-0.213732372	5.63E-06	no
GAD2	PROMOTER	Hs.231829	2572	10	26544791	26545498	708	7	5	8	0.12669857	0.004176075	no
GALNT14	PROMOTER	Hs.468058	79623	2	31214164	31215387	1224	9	11	12	0.06158854	0.009011239	no
GALNT17	PROMOTER	Hs.386236	442117	4	172969947	172970552	606	2	1	4	0.23232017	3.49E-05	no
GFRA1	PROMOTER	Hs.591913	2674	10	118023582	118024376	795	5	6	8	0.091192707	0.009972334	no
GIOT-1	PROMOTER		NA	19	41849712	41849982	271	4	2	2	0.175307221	0.00315852	no
GLB1L3	PROMOTER		NA	11	133650504	133650942	439	0	3	2	0.461228952	4.20E-05	no
GLDC	PROMOTER	Hs.584238	2731	9	6635729	6636075	347	0	4	4	0.153105883	0.000139609	no
GLIS1	PROMOTER	Hs.306691	148979	1	53975885	53976475	591	7	8	6	0.122458966	0.00094569	no
GLRA3	PROMOTER	Hs.413099	8001	4	175987114	175987457	344	0	1	1	0.43399799	0.000451474	no
GMFB	PROMOTER	Hs.151413	2764	14	54025442	54025689	248	2	2	2	-0.137617202	0.008866601	no
GNAS	PROMOTER	Hs.125898	2778	20	56859521	56860446	926	2	6	4	0.172815509	0.000495131	no
GPR103	PROMOTER	Hs.368977	84109	4	122521240	122521916	677	4	4	6	0.160224834	0.000472069	no
GPR45	PROMOTER	Hs.590903	11250	2	105219531	105220332	802	2	3	8	0.124484872	0.004442128	no
GRWD1	PROMOTER	Hs.400625	83743	19	53639406	53640250	845	2	3	1	0.253101228	0.000945784	no
HAND2	PROMOTER	Hs.388245	9464	4	174688964	174689658	695	4	4	7	0.214779452	0.000994998	no
HAND2	PROMOTER	Hs.388245	9464	4	174688218	174688789	572	1	4	4	0.27485995	0.008403394	no
HEBP1	PROMOTER	Hs.642618	50865	12	13045711	13046651	941	4	7	3	0.125601032	0.00605735	no
HELT	PROMOTER	Hs.531242	391723	4	186176506	186176785	280	3	2	4	0.195003388	0.004081511	no
HELT	PROMOTER	Hs.531242	391723	4	186173711	186174457	747	2	5	3	0.158381812	0.008324263	no
HEY2	PROMOTER	Hs.144287	23493	6	126110342	126110840	499	6	3	5	0.189999257	0.008311563	no
HKR1	PROMOTER	Hs.657027	284459	19	42517216	42517416	201	1	1	2	0.269166514	0.001987278	no
HLXB9	PROMOTER		NA	7	156505550	156505856	307	3	3	4	0.166898636	0.002170194	no
HMGCLL1	PROMOTER	Hs.147054	54511	6	55551549	55552146	598	6	2	6	0.181866027	0.003989755	no
HOP	PROMOTER		NA	4	57217323	57217566	244	2	3	3	0.273072711	0.002016173	no
HOXA5	PROMOTER	Hs.655218	3202	7	27151145	27152339	1195	5	6	10	0.096407247	0.008087394	no
HOXA6	PROMOTER	Hs.679517	3203	7	27153315	27154143	829	5	10	8	0.140335672	0.007745501	no
HOXD10	PROMOTER	Hs.123070	3236	2	176689334	176690127	794	5	5	3	0.207433648	0.000112116	no
HOXD12	PROMOTER	Hs.450028	3238	2	176672377	176673963	1587	8	13	17	0.143829624	0.007289441	no
HOXD8	PROMOTER	Hs.301963	3234	2	176701073	176702151	1079	6	8	10	0.094994633	0.00721521	no
HRB	PROMOTER	Hs.591619	3267	2	228044952	228045500	549	9	12	4	-0.101652248	0.00074159	no
HS3ST3A1	PROMOTER	Hs.462270	9955	17	13445418	13446783	1366	9	13	12	0.079351103	0.000317208	no
hsa-mir-124a-1	PROMOTER		NA	8	9799580	9800226	647	2	2	2	0.299954182	7.92E-05	no
hsa-mir-124a-1	PROMOTER		NA	8	9800227	9800529	303	0	2	2	0.230666191	0.000149061	no
hsa-mir-124a-1	PROMOTER		NA	8	9798355	9798745	391	1	1	4	0.179527658	0.003525426	no
hsa-mir-124a-3	PROMOTER		NA	20	61279864	61280333	470	8	5	4	0.286716108	0.000313267	no
hsa-mir-335	PROMOTER		NA	7	129920006	129920207	202	1	1	1	0.274710446	0.004599964	no
hsa-mir-432	PROMOTER		NA	14	100419180	100421928	2749	2	7	4	-0.107214139	0.000775948	no
hsa-mir-7-2	PROMOTER		NA	15	86948180	86948889	710	2	5	3	0.153464509	0.006069141	no
HTR1B	PROMOTER	Hs.123016	3351	6	78229029	78231263	2235	11	14	19	0.087806867	0.001191897	no
HTR2C	PROMOTER	Hs.149037	3358	X	113724628	113725831	1204	12	7	12	0.082602209	0.005423377	no
IFNA8	PROMOTER	Hs.73890	3445	9	21392648	21393618	971	4	2	5	0.233960983	0.001023821	no

IGF2BP1	PROMOTER	Hs.144936	10642	17	44429039	44429240	202	1	4	2	0.372740451	0.009223622	no
IGF2BP3	PROMOTER	Hs.700696	10643	7	23480763	23481016	254	0	1	2	0.17290228	0.003802288	no
IRX3	PROMOTER	Hs.499205	79191	16	52882350	52882949	600	2	9	4	0.20456197	0.000499436	no
IRX3	PROMOTER	Hs.499205	79191	16	52882950	52883178	229	2	2	2	0.277154835	0.001162154	no
KCNK12	PROMOTER	Hs.591586	56660	2	47651948	47653493	1546	8	10	8	0.086465372	0.002025198	no
KCTD8	PROMOTER	Hs.479644	386617	4	44144227	44145795	1569	18	23	14	0.068285578	0.003802288	no
KDR	PROMOTER	Hs.479756	3791	4	55686804	55687067	264	3	2	2	0.376747799	1.73E-05	no
KHDRBS2	PROMOTER	Hs.519794	202559	6	63054066	63054358	293	3	0	3	0.406241747	0.000765753	no
KIAA0644	PROMOTER	Hs.21572	9865	7	28964918	28965070	153	1	3	1	0.601744226	0.001488697	no
KIAA1729	PROMOTER	Hs.455089	85460	4	10071805	10072461	657	11	2	6	0.160337746	1.48E-05	no
KIAA1729	PROMOTER	Hs.455089	85460	4	10072470	10072935	466	3	1	3	0.308945018	0.001080395	no
KIAA1853	PROMOTER	Hs.112577	84530	12	117902597	117903266	670	0	4	3	0.474247831	0.002173948	no
KRT7	PROMOTER	Hs.411501	3855	12	50912273	50913550	1278	8	8	6	0.187367152	2.07E-05	no
LBH	PROMOTER	Hs.567598	81606	2	30306811	30307335	525	3	2	4	0.11239463	0.006205044	no
LHX2	PROMOTER	Hs.696425	9355	9	125812796	125813417	622	1	7	4	0.101776333	0.004550122	no
LHX5	PROMOTER	Hs.302029	64211	12	112401405	112402252	848	4	4	7	0.141847986	0.005256117	no
LHX5	PROMOTER	Hs.302029	64211	12	112397982	112398204	223	3	2	2	0.175669007	0.00730947	no
LHX5	PROMOTER	Hs.302029	64211	12	112398291	112398998	708	2	7	6	0.073474682	0.008080187	no
LIN28B	PROMOTER	Hs.23616	389421	6	105507478	105508425	948	4	2	6	0.180317581	0.001316092	no
LOC149134	PROMOTER	Hs.417972	149134	1	245018691	245019332	642	4	8	6	0.113274839	0.000453355	no
LOC283677	PROMOTER	Hs.700903	283677	15	71521969	71523232	1264	2	3	5	-0.112224301	0.001378993	no
LOC286187	PROMOTER	Hs.632064	286187	8	68103182	68103842	661	4	7	5	0.589739453	0.00237766	no
LOC340602	PROMOTER	Hs.97053	340602	X	51166208	51168230	2023	6	13	15	-0.079579667	0.009033014	no
LOC399706	PROMOTER		NA	10	1194909	1196146	1238	6	4	4	-0.170355137	2.27E-05	no
LOC751071	PROMOTER	Hs.640726	751071	11	62188692	62190239	1548	10	16	13	-0.079649398	0.000992694	no
LRAT	PROMOTER	Hs.658427	9227	4	155883235	155883453	219	2	3	1	0.158235256	0.000453457	no
LRAT	PROMOTER	Hs.658427	9227	4	155884372	155884859	488	5	8	6	0.309927044	0.002564307	no
LRRC4	PROMOTER	Hs.655003	64101	7	127459491	127460125	635	10	14	3	0.10219478	0.000963819	no
LYPD1	PROMOTER	Hs.694844	116372	2	133144875	133145523	649	1	8	6	0.291362199	0.005601251	no
MAP9	PROMOTER	Hs.61271	79884	4	156517079	156517892	814	5	7	6	0.142674002	0.003063901	no
MEF2D	PROMOTER	Hs.314327	4209	1	154741063	154741738	676	1	4	2	0.128295572	0.003064348	no
MEST	PROMOTER	Hs.270978	4232	7	129918628	129918876	249	0	1	3	0.194008068	0.00547624	no
MGC42090	PROMOTER		NA	7	19779132	19780201	1070	4	3	5	0.202331846	0.004343151	no
MMP16	PROMOTER	Hs.546267	4325	8	89409209	89409853	645	2	3	5	0.186792645	0.003827697	no
MSX1	PROMOTER	Hs.424414	4487	4	4910508	4910868	361	1	7	2	0.395901987	2.59E-07	no
MTERF	PROMOTER	Hs.532216	7978	7	91347860	91348424	565	4	3	5	0.346123476	0.00487057	no
MTMR15	PROMOTER	Hs.584863	22909	15	28982988	28983270	283	1	1	2	-0.100390095	0.003388515	no
MTMR7	PROMOTER	Hs.625674	9108	8	17315448	17315931	484	2	2	1	0.717446426	7.65E-07	no
NEFM	PROMOTER	Hs.458657	4741	8	24826707	24828565	1859	16	10	22	0.106344216	0.000455933	no
NETO1	PROMOTER	Hs.465407	81832	18	68684786	68685601	816	8	9	9	0.272601553	8.06E-05	no
NETO1	PROMOTER	Hs.465407	81832	18	68686673	68686944	272	6	0	2	0.340815533	0.000536952	no
NKPD1	PROMOTER	Hs.299256	284353	19	50347037	50349017	1981	30	14	21	0.061027326	0.005566775	no
NKX2-5	PROMOTER	Hs.54473	1482	5	172603234	172603489	256	1	1	1	0.267842084	0.000828566	no
NKX2-5	PROMOTER	Hs.54473	1482	5	172603490	172604450	961	7	13	9	0.09024222	0.001316819	no
NMNAT2	PROMOTER	Hs.497123	23057	1	181654184	181654508	325	0	1	2	0.248511147	0.005964081	no
NMUR1	PROMOTER	Hs.471619	10316	2	232102767	232103703	937	9	10	8	0.077582076	0.005185371	no
NOL10	PROMOTER	Hs.222494	79954	2	10746872	10748626	1755	6	14	8	-0.052143431	0.00333572	no
NPAS3	PROMOTER	Hs.659456	64067	14	32472213	32472634	422	4	2	4	0.38750189	1.69E-05	no
NPAS3	PROMOTER	Hs.659456	64067	14	32471645	32472212	568	1	6	4	0.15621722	0.004149361	no
NR0B1	PROMOTER	Hs.268490	190	X	30236240	30237598	1359	13	9	13	0.084920023	0.002968164	no
NR3C1	PROMOTER	Hs.122926	2908	5	142764704	142765514	811	6	3	6	0.206989521	0.000163975	no
NTNG2	PROMOTER	Hs.163642	84628	9	134029730	134029920	191	1	2	1	0.241975254	0.001400734	no
NUDT11	PROMOTER	Hs.200016	55190	X	51256428	51256926	499	10	1	4	0.116412966	0.004257499	no
OCA2	PROMOTER	Hs.654411	4948	15	26026121	26026814	694	6	9	7	0.231870357	0.000179092	no
OPCML	PROMOTER	Hs.4817	4978	11	132319158	132319639	482	7	2	4	0.167071308	0.000102586	no
OSAP	PROMOTER		NA	4	140420420	140421107	688	6	6	5	0.164562708	0.001393321	no
OTX1	PROMOTER	Hs.445340	5013	2	63128953	63129211	259	2	3	2	0.258421591	0.000514422	no
OTX1	PROMOTER	Hs.445340	5013	2	63129212	63129430	219	2	7	2	0.247072923	0.001126561	no
PAFAH1B3	PROMOTER	Hs.466831	5050	19	47502112	47504062	1951	9	9	4	-0.084844931	0.000766238	no
PAK1IP1	PROMOTER	Hs.310231	55003	6	10803063	10803420	358	1	4	4	-0.063609366	0.008415117	no
PALM2	PROMOTER		NA	9	111442331	111443074	744	12	6	6	0.148783572	0.004469824	no
PAQR9	PROMOTER	Hs.408385	344838	3	144165683	144165989	307	2	1	2	0.457631669	2.52E-05	no
PAQR9	PROMOTER	Hs.408385	344838	3	144165058	144165423	366	9	3	2	0.166234516	0.00388373	no
PCDH10	PROMOTER	Hs.192859	57575	4	134288581	134289155	575	5	4	5	0.77547213	4.06E-08	no
PCDH10	PROMOTER	Hs.192859	57575	4	134289508	134290167	660	2	3	6	0.089555238	0.001008505	no
PCDH10	PROMOTER	Hs.192859	57575	4	134289156	134289382	227	2	0	1	0.312109685	0.00187407	no
PCDH11Y	PROMOTER	Hs.661308	83259	Y	4927873	4928285	413	2	2	4	0.421304999	0.002073576	no
PCDH17	PROMOTER	Hs.106511	27253	13	57101980	57102214	235	2	3	2	0.351107378	0.00130169	no
PCDH19	PROMOTER	Hs.4993	57526	X	99553722	99553961	240	2	2	2	0.252581871	0.004274999	no
PCDH9	PROMOTER	Hs.654709	5101	13	66703204	66703772	569	4	3	3	0.111672472	0.000533244	no
PCDHGA7	PROMOTER		NA	5	140742288	140742967	680	2	3	4	0.110390038	0.007836742	no
PCDHGB5	PROMOTER		NA	5	140757581	140758255	675	3	4	1	0.229260443	0.002517854	no
PDE4B	PROMOTER	Hs.198072	5142	1	66031157	66032257	1101	12	9	4	0.088422237	0.003683748	no
PDGFRA	PROMOTER	Hs.74615	5156	4	54787961	54788161	201	0	2	2	0.273217544	0.004600354	no
PDPN	PROMOTER	Hs.468675	10630	1	13783051	13783838	788	4	4	4	0.287095821	0.000241071	no
PLP2	PROMOTER	Hs.77422	5355	X	48914896	48915950	1055	9	11	11	-0.068067663	0.001907247	no
PPP2R2B	PROMOTER	Hs.655213	5521	5	146238541	146238920	380	4	1	4	0.482386221	0.000165128	no
PRDM14	PROMOTER	Hs.287532	63978	8	71146214	71146670	457	3	5	4	0.176646076	0.000556029	no
PRMT8	PROMOTER	Hs.504530	56341	12	3470320	3470987	668	6	13	6	0.084503069	0.002509268	no
PRRX1	PROMOTER	Hs.702224	5396	1	168896971	168897532	562	3	3	5	0.143154109	0.004076698	no
PSKH2	PROMOTER	Hs.680136	85481	8	87150411	87151351	941	2	3	5	0.095273473	0.006252607	no
PTGFR	PROMOTER	Hs.654365	5737	1	78729104	78729568	465	7	2	4	0.162561284	0.000147887	no
PTPLAD2	PROMOTER	Hs.136247	401494	9	21021102	21021895	794	6	3	4	0.148172502	2.59E-05	no
PTPRZ1	PROMOTER	Hs.489824	5803	7	121300038	121300689	652	6	8	4	0.0951437	0.00632206	no
RACGAP1	PROMOTER	Hs.505469	29127	12	48712806	48714130	1325	9	2	4	0.152799913	0.008493556	no
RELN	PROMOTER	Hs.655654	5649	7	103416502	103417370	869	11	6	6	0.110703258	0.001318683	no
RERG	PROMOTER	Hs.199487	85004	12	15265180	15265760	581	5	3	7	0.181924171	0.002348991	no
RHBDD1	PROMOTER	Hs.471514	84236	2	227408316	227408931	616	9	5	6	0.149597761	0.005375639	no
RIC3	PROMOTER	Hs.655660	79608	11	8146682	8147607	926	6	7	6	0.240916584	0.001582896	no
ROBO2	PROMOTER	Hs.13305	6092	3	77171324	77172099	776	8	11	6	0.08068858	0.008754223	no

RPS26	PROMOTER	Hs.447562	6231	12	54721499	54722579	1081	4	7	8	-0.073468302	0.000689882	no
RPS6KA2	PROMOTER	Hs.655277	6196	6	167195652	167196097	446	1	6	4	0.148409483	0.009137303	no
RSPO4	PROMOTER	Hs.444980	343637	20	930580	931023	444	5	11	4	0.073654648	0.002790858	no
SALL1	PROMOTER	Hs.135787	6299	16	49745024	49745436	413	3	5	4	0.245298515	3.00E-06	no
SALL1	PROMOTER	Hs.135787	6299	16	49745587	49746750	1164	6	5	7	0.125526295	0.004755144	no
SALL1	PROMOTER	Hs.135787	6299	16	49747087	49747651	565	5	6	5	0.139902171	0.005672152	no
SALL1	PROMOTER	Hs.135787	6299	16	49743425	49744679	1255	11	20	14	0.083543782	0.00780831	no
SEC31B	PROMOTER	Hs.18889	25956	10	102269744	102270026	283	0	2	1	0.273486283	0.002157771	no
SFMBT2	PROMOTER	Hs.407983	57713	10	7493146	7493537	392	4	1	4	0.435854789	5.96E-06	no
SFRP2	PROMOTER	Hs.481022	6423	4	154929895	154930494	600	6	3	4	0.232354383	0.000138357	no
SH3GL3	PROMOTER	Hs.270055	6457	15	81906756	81908098	1343	16	19	11	0.06775125	0.003786294	no
SHC3	PROMOTER	Hs.656806	53358	9	90983368	90983861	494	1	2	2	0.405485019	0.000295506	no
SHOX	PROMOTER	Hs.105932	6473	Y	500542	501217	676	8	3	8	0.134829717	0.000505078	no
SHOX	PROMOTER	Hs.105932	6473	Y	501218	501517	300	2	1	2	0.263748465	0.002770604	no
SIX3	PROMOTER	Hs.658847	6496	2	45013368	45014087	720	3	2	4	0.627310815	5.82E-07	no
SIX3	PROMOTER	Hs.658847	6496	2	45017754	45018153	400	1	1	2	0.311467165	1.52E-05	no
SLC25A14	PROMOTER	Hs.194686	9016	X	129300603	129301320	718	4	3	3	-0.084341347	0.003608335	no
SLC27A6	PROMOTER	Hs.49765	28965	5	128328543	128329509	967	8	5	9	0.112577507	0.000157489	no
SLC45A1	PROMOTER	Hs.463036	50651	1	8300510	8300690	181	5	4	1	-0.154961918	0.005102524	no
SLC6A16	PROMOTER	Hs.130949	28968	19	54519466	54520608	1143	3	2	5	-0.137025201	0.000518806	no
SLC6A19	PROMOTER	Hs.585128	340024	5	1254590	1255921	1332	2	14	6	-0.08501348	0.000226594	no
SLIT2	PROMOTER	Hs.699467	9353	4	19863583	19865419	1837	13	12	19	0.1201434	0.00139181	no
SLIT2	PROMOTER	Hs.699467	9353	4	19862706	19863582	877	5	13	10	0.104439681	0.009410686	no
SLITRK5	PROMOTER	Hs.591208	26050	13	87121345	87122527	1183	10	12	12	0.161398217	0.000287097	no
SMAD6	PROMOTER	Hs.153863	4091	15	64781224	64781553	330	5	4	2	-0.085292876	0.007541239	no
SNAP25	PROMOTER	Hs.167317	6616	20	10146379	10147276	898	1	3	7	0.11191869	0.003176233	no
SOHLH2	PROMOTER	Hs.124519	54937	13	35686286	35686796	511	2	5	6	-0.076229306	0.009223622	no
SPAG17	PROMOTER	Hs.528821	200162	1	118529198	118529903	706	1	1	4	0.226978197	0.008545802	no
ST8SIA5	PROMOTER	Hs.465025	29906	18	42591126	42592350	1225	8	9	8	0.158123733	0.005928752	no
STOX2	PROMOTER	Hs.21958	56977	4	185063324	185063884	561	5	7	5	0.265057432	0.002532493	no
SYT6	PROMOTER	Hs.370963	148281	1	114498310	114498811	502	1	0	4	0.182561833	0.004906733	no
T	PROMOTER	Hs.389457	6862	6	166503176	166503433	258	1	0	2	0.161198115	0.000677992	no
T	PROMOTER	Hs.389457	6862	6	166500731	166502429	1699	22	21	17	0.062268402	0.006597096	no
T	PROMOTER	Hs.389457	6862	6	166502430	166503175	746	3	8	8	0.073655643	0.006772794	no
TBX15	PROMOTER	Hs.146196	6913	1	119337057	119338108	1052	4	8	5	0.101382717	0.007178796	no
TBX20	PROMOTER	Hs.404167	57057	7	35263766	35265008	1243	15	9	12	0.101657224	4.95E-05	no
TBX20	PROMOTER	Hs.404167	57057	7	35259349	35260260	912	7	4	9	0.128365708	0.000811671	no
TBX20	PROMOTER	Hs.404167	57057	7	35263480	35263753	274	0	3	3	0.256515202	0.001028072	no
TBX3	PROMOTER	Hs.129895	6926	12	113609422	113609653	232	0	3	2	0.306747432	0.003702399	no
TFAP2C	PROMOTER	Hs.473152	7022	20	54633593	54633902	310	1	2	3	0.564786235	4.08E-05	no
TFAP2C	PROMOTER	Hs.473152	7022	20	54633903	54634427	525	0	7	5	0.164220297	0.005351228	no
TFPI2	PROMOTER	Hs.438231	7980	7	93357262	93358480	1219	6	8	11	0.080821384	0.001907247	no
THRAP5	PROMOTER		NA	19	841449	844392	2944	11	12	7	-0.064864116	0.009177566	no
TKTL2	PROMOTER	Hs.303923	84076	4	164613506	164614627	1122	2	4	8	-0.130106101	0.000439046	no
TMEM108	PROMOTER	Hs.191616	66000	3	134239564	134240336	773	8	8	7	0.097921972	0.001250274	no
TMEM155	PROMOTER	Hs.27524	132332	4	122905084	122906076	993	12	9	8	0.066006113	0.002209432	no
TMEM22	PROMOTER	Hs.655019	80723	3	138020124	138020314	191	0	1	1	0.250717039	0.003034798	no
TMEM46	PROMOTER	Hs.433791	387914	13	25522566	25523535	970	11	9	11	0.181204736	0.000489647	no
TMEM74	PROMOTER	Hs.99439	157753	8	109868580	109869187	608	1	8	6	0.091488336	0.006090289	no
TMUB2	PROMOTER	Hs.181391	79089	17	39619779	39620048	270	2	4	2	-0.129414559	0.003690434	no
TRIM67	PROMOTER	Hs.655089	440730	1	229362917	229363527	611	2	3	4	0.083319297	0.008749775	no
TRIM71	PROMOTER	Hs.567678	131405	3	32833099	32833780	682	17	6	5	0.29107632	0.001358584	no
TRIM71	PROMOTER	Hs.567678	131405	3	32834107	32834365	259	2	3	2	0.140982525	0.001376998	no
TRPC4	PROMOTER	Hs.262960	7223	13	37341660	37342039	380	1	3	4	0.201175695	6.60E-05	no
TSPYL6	PROMOTER	Hs.620508	388951	2	54335971	54337036	1066	5	6	6	-0.127943525	0.00388373	no
TXNDC10	PROMOTER	Hs.440534	54495	18	64533491	64533699	209	2	2	2	-0.138369491	0.009589259	no
UNC84A	PROMOTER	Hs.438072	23353	7	819319	820021	703	0	5	5	-0.098936599	0.006796562	no
UNCX4.1	PROMOTER		NA	7	1236666	1236837	172	3	1	1	0.530630433	2.23E-08	no
UNCX4.1	PROMOTER		NA	7	1237014	1237233	220	4	2	2	0.118175581	0.001396548	no
USP44	PROMOTER	Hs.646421	84101	12	94466034	94467117	1084	18	19	13	0.105602943	0.000514422	no
VSTM2	PROMOTER		NA	7	54577122	54577473	352	3	4	3	0.173002484	0.000154518	no
WNT2	PROMOTER	Hs.567356	7472	7	116750282	116751076	795	7	5	7	0.401068001	0.007149715	no
ZNF134	PROMOTER	Hs.469694	7693	19	62817189	62818000	812	5	5	6	0.118250971	0.000536421	no
ZNF135	PROMOTER	Hs.85863	7694	19	63261745	63262894	1150	9	13	9	0.118149354	0.001718281	no
ZNF177	PROMOTER	Hs.172979	7730	19	9334506	9335034	529	1	8	5	0.112665601	0.006218678	no
ZNF229	PROMOTER		NA	19	49644037	49644846	810	3	2	6	0.130357596	2.35E-05	no
ZNF300	PROMOTER	Hs.134885	91975	5	150264528	150264838	311	1	2	2	0.32237735	0.003957164	no
ZNF304	PROMOTER	Hs.287374	57343	19	62554195	62555239	1045	5	8	10	0.204646582	0.000113388	no
ZNF331	PROMOTER	Hs.185674	55422	19	58715092	58716158	1067	5	5	7	0.192574915	0.00139841	no
ZNF454	PROMOTER	Hs.259441	285676	5	178300053	178301712	1660	6	8	16	0.081839984	0.004618698	no
ZNF471	PROMOTER	Hs.230188	57573	19	61710613	61711840	1228	11	5	9	0.143190509	0.000406821	no
ZNF528	PROMOTER	Hs.662043	84436	19	57592678	57592991	314	2	6	2	0.109412359	0.008397197	no
ZNF545	PROMOTER	Hs.558734	284406	19	41601606	41601799	194	2	1	2	0.367218681	1.47E-05	no
ZNF560	PROMOTER	Hs.631613	147741	19	9469934	9470477	544	4	1	6	0.704591602	7.65E-07	no
ZNF582	PROMOTER	Hs.244391	147948	19	61596321	61597205	885	7	13	8	0.094241935	0.002348991	no
ZNF597	PROMOTER	Hs.88630	146434	16	3433372	3433610	239	2	1	2	0.091606989	0.006637521	no
ZNF671	PROMOTER	Hs.180402	79891	19	62930272	62931359	1088	5	6	7	0.187906834	0.000776567	no
ZNF680	PROMOTER	Hs.520886	340252	7	63664809	63667646	2838	3	6	3	0.147762778	0.00565699	no
ZNF701	PROMOTER		NA	19	57764868	57765418	551	3	2	2	0.138084818	0.004197537	no
ZNF790	PROMOTER	Hs.282067	388536	19	42021240	42021801	562	1	2	2	0.271706696	0.000992064	no
ZNF793	PROMOTER	Hs.568010	390927	19	42689533	42689892	360	2	2	4	0.676318407	5.67E-06	no
ZSWIM2	PROMOTER	Hs.375054	151112	2	187421724	187422335	612	0	3	3	0.198167826	0.00016776	no
ADAMTS5	INSIDE	Hs.58324	11096	21	27259363	27260649	1287	16	11	10	0.106585781	0.007486039	yes
ADAMTSL3	INSIDE	Hs.459162	57188	15	82113854	82114661	808	10	8	8	0.252220361	8.23E-05	yes
ADCY8	INSIDE	Hs.591859	114	8	132122466	132123014	549	4	2	5	0.405854288	7.81E-05	yes
ADCY8	INSIDE	Hs.591859	114	8	132121332	132122465	1134	5	11	12	0.10368965	0.003064348	yes
ADCYAP1	INSIDE	Hs.531719	116	18	898533	899602	1070	8	5	11	0.176299248	0.000155225	yes
ADCYAP1	INSIDE	Hs.531719	116	18	896252	896767	516	3	5	5	0.337986998	0.000245173	yes
ADCYAP1	INSIDE	Hs.531719	116	18	896931	898286	1356	13	10	14	0.081174613	0.003533153	yes
ADRB3	INSIDE	Hs.2549	155	8	37941850	37943347	1498	10	19	15	0.109327446	0.000342446	yes

ADRB3	INSIDE	Hs.2549	155	8	37941619	37941849	231	0	2	2	0.189583728	0.001064249	yes
ALDH1A2	INSIDE	Hs.699620	8854	15	56144814	56145034	221	0	2	2	0.312915672	6.26E-05	yes
ALDH1A2	INSIDE	Hs.699620	8854	15	56144411	56144813	403	1	6	3	0.11084626	0.003228543	yes
ALOX12	INSIDE	Hs.654431	239	17	6840064	6840628	565	9	10	6	0.148290818	0.000596883	yes
ALX4	INSIDE	Hs.436055	60529	11	44287506	44287926	421	2	2	3	0.500520673	6.35E-05	yes
ALX4	INSIDE	Hs.436055	60529	11	44281401	44281820	420	2	3	4	0.346612914	0.00045363	yes
ALX4	INSIDE	Hs.436055	60529	11	44287927	44288540	614	4	6	4	0.17935295	0.004049806	yes
ALX4	INSIDE	Hs.436055	60529	11	44282532	44282959	428	4	6	4	0.198795159	0.005269189	yes
ARID3C	INSIDE	Hs.534549	138715	9	34613428	34614398	971	3	6	7	0.101583201	0.007735076	yes
B4GALNT1	INSIDE	Hs.591019	2583	12	56311538	56312447	910	6	8	5	0.217639939	2.13E-05	yes
B4GALNT1	INSIDE	Hs.591019	2583	12	56307743	56308507	765	5	5	7	0.262786857	0.001343836	yes
BDNF	INSIDE	Hs.502182	627	11	27698535	27698720	186	0	1	1	0.1518033	0.008596481	yes
BRUNOL4	INSIDE	Hs.435976	56853	18	33399495	33399984	490	3	2	2	0.577532206	3.74E-05	yes
BRUNOL4	INSIDE	Hs.435976	56853	18	33358513	33358666	154	3	2	1	0.2454927	0.006218678	yes
BTNL9	INSIDE	Hs.546502	153579	5	180418394	180419811	1418	13	10	11	0.078820367	0.003064912	yes
BVES	INSIDE	Hs.221660	11149	6	105690708	105690939	232	1	1	1	0.266411211	0.000635057	yes
C20orf103	INSIDE	Hs.22920	24141	20	9444850	9445352	503	0	1	1	0.344568252	6.03E-05	yes
C20orf103	INSIDE	Hs.22920	24141	20	9443922	9444849	928	7	6	5	0.138272526	0.000922029	yes
CCDC105	INSIDE	Hs.375985	126402	19	14981629	14983077	1449	11	2	6	0.094055047	0.001077316	yes
CDH13	INSIDE	Hs.654386	1012	16	81217780	81218495	716	5	4	4	0.160621962	0.000372492	yes
CDKN2A	INSIDE	Hs.512599	1029	9	21979342	21979929	588	2	1	3	0.320175455	0.001144224	yes
CDKN2A	INSIDE	Hs.512599	1029	9	21979930	21980266	337	0	2	3	0.406966348	0.004003749	yes
CHX10	INSIDE		NA	14	73777686	73778432	747	1	8	3	0.189102632	5.12E-05	yes
CLDN11	INSIDE	Hs.31595	5010	3	171620070	171620691	622	5	6	6	0.181429245	1.02E-05	yes
COCH	INSIDE	Hs.21016	1690	14	30414211	30414601	391	1	2	3	0.376543178	0.000963469	yes
COL12A1	INSIDE	Hs.101302	1303	6	75971207	75971994	788	5	5	8	0.196225213	0.003016494	yes
COMP	INSIDE	Hs.1584	1311	19	18759059	18759477	419	0	4	4	0.326758598	0.0038864	yes
CPEB1	INSIDE	Hs.547988	64506	15	81111950	81113123	1174	7	7	13	0.118458675	0.001404546	yes
CSMD1	INSIDE	Hs.571466	64478	8	4836829	4837242	414	3	1	4	0.090967944	0.005564927	yes
CYP26A1	INSIDE	Hs.150595	1592	10	94824499	94825309	811	9	4	7	0.315773999	8.05E-07	yes
CYP26C1	INSIDE	Hs.369993	340665	10	94815533	94816521	989	6	7	6	0.141194105	0.001075804	yes
DGKG	INSIDE	Hs.592771	1608	3	187561014	187561935	922	4	3	6	0.338559032	4.89E-05	yes
DMRT1	INSIDE	Hs.98586	1761	9	833155	833405	251	3	1	2	0.246928608	0.000149308	yes
DMRT3	INSIDE	Hs.189174	58524	9	967792	968178	387	0	3	1	0.231463347	0.001297954	yes
DOK6	INSIDE	Hs.569915	220164	18	65220829	65221349	521	0	1	4	0.173536451	0.001729288	yes
DPP10	INSIDE	Hs.591555	57628	2	115636417	115636673	257	0	2	3	0.36085266	0.000102647	yes
DPP10	INSIDE	Hs.591555	57628	2	115636681	115637069	389	1	3	5	0.261582305	0.002617589	yes
DUOXA1	INSIDE	Hs.356664	90527	15	43208294	43208598	305	0	2	2	0.292789538	0.000194318	yes
EBF2	INSIDE	Hs.491292	64641	8	25955237	25955851	615	2	3	4	0.35312168	2.51E-05	yes
EBF2	INSIDE	Hs.491292	64641	8	25957163	25957512	350	0	1	3	0.320926502	0.000886358	yes
EBF2	INSIDE	Hs.491292	64641	8	25956448	25956860	413	1	4	4	0.323904436	0.001075804	yes
EGFLAM	INSIDE	Hs.20103	133584	5	38294621	38294823	203	1	3	2	0.109472795	0.006274289	yes
EPHA7	INSIDE	Hs.73962	2045	6	94183070	94184551	1482	13	7	14	0.11388534	0.000192481	yes
EPS8L1	INSIDE	Hs.438862	54869	19	60288902	60290937	2036	7	21	12	0.069294818	0.005359441	yes
EYA4	INSIDE	Hs.661127	2070	6	133604512	133605111	600	8	10	6	0.428059132	1.34E-05	yes
EYA4	INSIDE	Hs.661127	2070	6	133605174	133605603	430	1	1	4	0.115794304	0.009177566	yes
FAM19A2	INSIDE	Hs.269745	338811	12	60871820	60872732	913	3	2	8	0.402218684	7.97E-07	yes
FAM19A2	INSIDE	Hs.269745	338811	12	60870913	60871819	907	7	9	9	0.174713779	1.55E-06	yes
FAM19A4	INSIDE	Hs.187873	151647	3	69063399	69063678	280	1	1	3	0.294001816	0.000190673	yes
FAM47A	INSIDE	Hs.143268	158724	X	34058384	34060603	2220	3	18	4	-0.122429482	0.009972334	yes
FBN1	INSIDE	Hs.591133	2200	15	46723936	46724098	163	0	1	1	0.31901763	0.000166165	yes
FEZF2	INSIDE	Hs.241523	55079	3	62331454	62332375	922	4	7	8	0.12762508	0.00042654	yes
FEZF2	INSIDE	Hs.241523	55079	3	62330882	62331453	572	4	3	5	0.18104776	0.000640791	yes
FEZF2	INSIDE	Hs.241523	55079	3	62332376	62333678	1303	6	12	11	0.053197659	0.00963242	yes
FLI1	INSIDE	Hs.504281	2313	11	128069170	128069377	208	1	2	2	0.376202117	5.10E-06	yes
FLJ45557	INSIDE	Hs.613882	642938	10	128882813	128883791	979	4	7	3	0.222245346	5.10E-06	yes
FLJ45983	INSIDE	Hs.669736	399717	10	8134298	8134613	316	4	1	2	0.344520022	5.63E-06	yes
FLRT2	INSIDE	Hs.533710	23768	14	85067121	85068078	958	9	4	9	0.267139919	0.000118085	yes
FOXF2	INSIDE	Hs.484423	2295	6	1338671	1338963	293	3	4	4	0.255777045	0.000672389	yes
FRAT1	INSIDE	Hs.126057	10023	10	99070249	99070534	286	3	2	3	0.142240329	0.006192178	yes
GATA2	INSIDE	Hs.367725	2624	3	129692480	129692768	289	1	2	2	0.303434858	0.006615669	yes
GATA3	INSIDE	Hs.524134	2625	10	8137442	8137776	335	5	5	2	0.095701755	0.001295165	yes
GATA4	INSIDE	Hs.243987	2626	8	11604571	11605037	467	3	1	1	0.743198521	7.09E-05	yes
GATA4	INSIDE	Hs.243987	2626	8	11600187	11600754	568	0	2	2	0.186381661	0.001555083	yes
GATA4	INSIDE	Hs.243987	2626	8	11602465	11603061	597	2	7	4	0.165318551	0.008107674	yes
GCM2	INSIDE	Hs.227098	9247	6	10989239	10990102	864	3	3	2	0.354951092	1.44E-06	yes
GDF6	INSIDE	Hs.492277	392255	8	97240965	97241564	600	2	6	3	0.211967587	3.06E-05	yes
GDF6	INSIDE	Hs.492277	392255	8	97240522	97240964	443	0	1	1	0.380058439	0.001027128	yes
GDF6	INSIDE	Hs.492277	392255	8	97226964	97227622	659	4	8	3	0.130802104	0.005197691	yes
GDNF	INSIDE	Hs.248114	2668	5	37870101	37870657	557	5	2	2	0.248217991	0.000289066	yes
GFRA4	INSIDE	Hs.302025	64096	20	3587916	3589619	1704	7	11	6	0.091274782	0.000853273	yes
GHR	INSIDE	Hs.125180	2690	5	42460197	42460529	333	6	1	3	0.255158861	0.000305929	yes
GRASP	INSIDE	Hs.407202	160622	12	50687523	50688042	520	5	4	6	0.171133513	0.000746031	yes
GRIK1	INSIDE	Hs.695938	2897	21	30233703	30234180	478	4	4	4	0.283892439	0.000107728	yes
GRIK1	INSIDE	Hs.695938	2897	21	30232913	30233702	790	5	7	6	0.124013849	0.001652578	yes
GRM7	INSIDE	Hs.606393	2917	3	6879128	6879905	778	3	8	6	0.134528225	0.008766997	yes
GSC	INSIDE	Hs.440438	145258	14	94304351	94304729	379	6	2	4	0.422760225	0.003263528	yes
HAND1	INSIDE	Hs.152531	9421	5	153836887	153837430	544	2	1	2	0.218327832	0.000514422	yes
HAS1	INSIDE	Hs.57697	3036	19	56913884	56915029	1146	14	6	9	0.068746428	0.006965205	yes
HMX2	INSIDE	Hs.444756	3167	10	124898903	124899154	252	0	1	2	0.298572672	9.29E-05	yes
HOXB13	INSIDE	Hs.66731	10481	17	44159152	44159827	676	3	2	3	0.250563945	0.000321682	yes
HOXC13	INSIDE	Hs.118608	3229	12	52624847	52625401	555	4	3	5	0.330811579	2.21E-05	yes
HOXC13	INSIDE	Hs.118608	3229	12	52618934	52620175	1242	6	24	11	0.06248096	0.009566788	yes
HOXC4	INSIDE	Hs.549040	3221	12	52733972	52734774	803	2	5	4	0.220299767	0.001121888	yes
HTR7	INSIDE	Hs.73739	3363	10	92606747	92607395	649	5	8	5	0.14250744	0.001680001	yes
ICAM4	INSIDE	Hs.699174	3386	19	10259248	10260190	943	4	6	5	0.179095759	0.000181446	yes
INTS10	INSIDE	Hs.512627	55174	8	19719448	19719857	410	1	3	1	-0.219117687	4.28E-05	yes
IRX1	INSIDE	Hs.424156	79192	5	3653505	3653764	260	0	3	3	0.270099442	0.000199686	yes
IRX1	INSIDE	Hs.424156	79192	5	3651777	3653504	1728	10	14	13	0.094078865	0.000887404	yes
IRX1	INSIDE	Hs.424156	79192	5	3653765	3654155	391	4	6	5	0.140948304	0.000948342	yes

IRX5	INSIDE	Hs.435730	10265	16	53523333	53524589	1257	6	10	14	0.088819552	0.000838672	yes
ISL1	INSIDE	Hs.505	3670	5	50718406	50719378	973	1	3	4	0.185289778	0.004841373	yes
ISLR2	INSIDE	Hs.254775	57611	15	72214356	72214886	531	3	1	5	0.214785986	0.002587308	yes
ITGAM	INSIDE	Hs.172631	3684	16	31249373	31251248	1876	6	7	7	0.061160963	0.006048911	yes
KCNA1	INSIDE	Hs.416139	3736	12	4890035	4890473	439	0	3	5	0.446873263	6.02E-05	yes
KCNA4	INSIDE	Hs.592002	3739	11	29993960	29994365	406	0	3	1	0.289395321	0.001126561	yes
KCNAB1	INSIDE	Hs.654519	7881	3	157491664	157492083	420	3	3	4	0.38295297	1.85E-06	yes
KCNC2	INSIDE	Hs.591041	3747	12	73889256	73890259	1004	3	6	4	0.084952163	0.004751215	yes
KCNK13	INSIDE	Hs.510191	56659	14	89598014	89598904	891	9	17	8	0.067724913	0.006297951	yes
KCNK9	INSIDE	Hs.493037	51305	8	140782765	140784034	1270	4	6	4	0.121819739	0.002268245	yes
KCNQ5	INSIDE	Hs.675919	56479	6	73389560	73389809	250	3	1	3	0.491208789	0.000183817	yes
KIAA1772	INSIDE	Hs.149020	80000	18	17077178	17077413	236	1	3	2	0.146583721	0.006189014	yes
LBX1	INSIDE	Hs.37128	10660	10	102977666	102978273	608	2	4	6	0.274611885	0.002479556	yes
LBX1	INSIDE	Hs.37128	10660	10	102976747	102977665	919	7	15	8	0.062455877	0.003836074	yes
LBXCOR1	INSIDE	Hs.451224	390598	15	65908923	65909487	565	10	6	5	0.226538167	0.000105163	yes
LBXCOR1	INSIDE	Hs.451224	390598	15	65909488	65909782	295	1	2	2	0.496400684	0.000266728	yes
LBXCOR1	INSIDE	Hs.451224	390598	15	65905147	65906032	886	7	3	6	0.176228181	0.000712418	yes
LRRK1	INSIDE	Hs.407918	79705	15	99331058	99331943	886	14	8	8	0.229248267	1.14E-06	yes
LRRK1	INSIDE	Hs.407918	79705	15	99330289	99331057	769	3	3	4	0.139216529	0.003628608	yes
MAB21L1	INSIDE	Hs.584776	4081	13	34947753	34948527	775	3	8	6	0.483987147	0.003989755	yes
MAPT	INSIDE	Hs.101174	4137	17	41330670	41331018	349	1	1	2	0.345784868	0.000120498	yes
MAPT	INSIDE	Hs.101174	4137	17	41330059	41330605	547	6	4	6	0.121391431	0.004293643	yes
MDGA1	INSIDE	Hs.437993	266727	6	37772466	37773033	568	6	10	6	0.097104763	0.006388664	yes
MKX	INSIDE	Hs.128193	283078	10	28073989	28074433	445	5	7	4	0.460525704	8.76E-06	yes
MKX	INSIDE	Hs.128193	283078	10	28070263	28070967	705	4	5	9	0.176412483	0.000765753	yes
MKX	INSIDE	Hs.128193	283078	10	28073072	28073988	917	17	4	8	0.183364404	0.005057507	yes
MKX	INSIDE	Hs.128193	283078	10	28071089	28071309	221	3	2	2	0.191179816	0.005423377	yes
MSC	INSIDE	Hs.442619	9242	8	72916996	72917413	418	4	2	4	0.444699383	1.88E-06	yes
MSC	INSIDE	Hs.442619	9242	8	72918099	72919606	1508	11	10	12	0.082733437	0.000829303	yes
MYOD1	INSIDE	Hs.181768	4654	11	17697733	17699701	1969	19	16	20	0.059581899	0.003546003	yes
NDUFA4L2	INSIDE	Hs.221447	56901	12	55916140	55916710	571	2	7	5	0.338860541	2.72E-05	yes
NEUROG1	INSIDE	Hs.248149	4762	5	134898452	134899576	1125	9	13	12	0.119710306	0.003353614	yes
NFIX	INSIDE	Hs.257970	4784	19	12985996	12986182	187	0	3	2	0.31279511	0.000244637	yes
NFIX	INSIDE	Hs.257970	4784	19	12985554	12985719	166	1	1	1	-0.142128959	0.005028016	yes
NKX2-2	INSIDE	Hs.516922	4821	20	21440239	21441418	1180	10	10	12	0.200073927	4.88E-07	yes
NKX2-3	INSIDE	Hs.243272	159296	10	101284049	101284449	401	1	1	1	0.258796213	0.002813217	yes
NMBR	INSIDE	Hs.654478	4829	6	142450900	142451566	667	3	6	4	0.116722634	0.00714668	yes
NPR3	INSIDE	Hs.237028	4883	5	32747521	32748937	1417	10	9	12	0.103651144	0.00011623	yes
NR2E1	INSIDE	Hs.157688	7101	6	108595227	108595793	567	2	3	4	0.272728587	1.33E-05	yes
NRN1	INSIDE	Hs.103291	51299	6	5947873	5948643	771	1	1	5	0.184020678	4.08E-05	yes
NRN1	INSIDE	Hs.103291	51299	6	5946414	5947872	1459	3	5	4	0.220451745	0.000105729	yes
NRN1	INSIDE	Hs.103291	51299	6	5948644	5949342	699	5	11	6	0.214107255	0.002872303	yes
NRN1	INSIDE	Hs.103291	51299	6	5949855	5950215	361	2	1	3	0.163583162	0.00489149	yes
ONECUT2	INSIDE	Hs.194725	9480	18	53259471	53259954	484	7	2	5	0.471371361	3.17E-06	yes
ONECUT2	INSIDE	Hs.194725	9480	18	53257408	53257949	542	1	5	5	0.336694771	0.000588215	yes
OSR2	INSIDE	Hs.253247	116039	8	100029782	100031446	1665	4	13	10	0.115832377	0.006252607	yes
OTP	INSIDE	Hs.202247	23440	5	76969191	76969435	245	0	2	2	0.448346494	0.000144372	yes
PAX1	INSIDE	Hs.349082	5075	20	21642186	21642422	237	0	1	1	0.431375123	0.002587308	yes
PAX2	INSIDE	Hs.155644	5076	10	102577860	102578544	685	0	10	4	-0.078319128	0.001852936	yes
PAX2	INSIDE	Hs.155644	5076	10	102498073	102498611	539	3	2	5	0.169525175	0.002473883	yes
PAX2	INSIDE	Hs.155644	5076	10	102498933	102499949	1017	3	7	8	0.099240667	0.004513148	yes
PAX6	INSIDE	Hs.591993	5080	11	31783094	31783581	488	7	8	3	0.470766382	2.53E-06	yes
PAX6	INSIDE	Hs.591993	5080	11	31776550	31776841	292	1	3	2	0.44912521	1.83E-05	yes
PAX6	INSIDE	Hs.591993	5080	11	31784304	31784608	305	5	4	2	0.337270884	0.000104849	yes
PAX6	INSIDE	Hs.591993	5080	11	31782303	31783093	791	3	8	6	0.265509486	0.003984654	yes
PAX9	INSIDE	Hs.132576	5083	14	36205916	36206219	304	3	2	3	0.439140422	2.53E-05	yes
PAX9	INSIDE	Hs.132576	5083	14	36205075	36205915	841	6	5	8	0.1025625	0.009033014	yes
PCDH17	INSIDE	Hs.106511	27253	13	57103610	57105229	1620	10	10	7	0.270496208	9.16E-06	yes
PCDH17	INSIDE	Hs.106511	27253	13	57105230	57106300	1071	6	6	13	0.310486862	0.000729955	yes
PCDHGA11	INSIDE		NA	5	140780643	140781291	649	5	3	5	0.306921803	0.000180557	yes
PDX1	INSIDE	Hs.32938	3651	13	27396085	27397263	1179	9	10	10	0.157956751	6.79E-05	yes
PHOX2A	INSIDE	Hs.697462	401	11	71629737	71630930	1194	4	7	5	0.138603669	7.87E-05	yes
PITX1	INSIDE	Hs.84136	5307	5	134391349	134391677	329	2	3	2	0.267817039	0.000252642	yes
PITX1	INSIDE	Hs.84136	5307	5	134391678	134393165	1488	18	19	13	0.070651445	0.003628608	yes
POU4F3	INSIDE	Hs.553499	5459	5	145699247	145700121	875	5	4	7	0.143391629	0.008324263	yes
PRMT8	INSIDE	Hs.504530	56341	12	3472431	3473057	627	2	2	7	0.139614757	6.24E-05	yes
PRMT8	INSIDE	Hs.504530	56341	12	3473058	3473874	817	3	3	4	0.174103983	0.001527555	yes
RSPO2	INSIDE	Hs.444834	340419	8	109163318	109164294	977	4	10	7	0.127938392	0.002684495	yes
RSPO3	INSIDE	Hs.135254	84870	6	127483001	127483557	557	3	2	4	0.248905293	1.66E-05	yes
RSPO3	INSIDE	Hs.135254	84870	6	127481981	127482938	958	11	9	10	0.220680543	0.00012849	yes
SFMBT2	INSIDE	Hs.407983	57713	10	7489645	7490455	811	8	8	8	0.105747508	0.00333572	yes
SFMBT2	INSIDE	Hs.407983	57713	10	7490456	7491156	701	7	7	7	0.092449532	0.00988749	yes
SIM1	INSIDE	Hs.520293	6492	6	101003771	101004725	955	2	6	7	0.156087142	0.009566788	yes
SIM2	INSIDE	Hs.146186	6493	21	36998528	36999724	1197	9	12	13	0.169024813	0.000109347	yes
SIX1	INSIDE	Hs.633506	6495	14	60183924	60184331	408	1	1	4	0.179713106	0.000825139	yes
SIX1	INSIDE	Hs.633506	6495	14	60184546	60185143	598	8	4	7	0.223589288	0.006379162	yes
SIX6	INSIDE	Hs.194756	4990	14	60047432	60048097	666	3	4	6	0.222401016	0.000871779	yes
SIX6	INSIDE	Hs.194756	4990	14	60046844	60047431	588	5	2	5	0.177116407	0.00395116	yes
SLC12A5	INSIDE	Hs.21413	57468	20	44119362	44120195	834	14	13	8	0.090311886	0.001907247	yes
SLC13A3	INSIDE	Hs.655498	64849	20	44713017	44713530	514	4	7	4	0.237322063	0.002939822	yes
SLC32A1	INSIDE	Hs.179080	140679	20	36789889	36790803	915	10	5	10	0.213509483	2.04E-05	yes
SLC32A1	INSIDE	Hs.179080	140679	20	36788952	36789361	410	0	1	4	0.538915793	2.70E-05	yes
SLC32A1	INSIDE	Hs.179080	140679	20	36789431	36789888	458	4	3	5	0.231906036	0.000489647	yes
SLC8A3	INSIDE	Hs.337696	6547	14	69724729	69725455	727	11	11	6	0.076748687	0.001440433	yes
SLITRK1	INSIDE	Hs.415478	114798	13	83351877	83352089	213	0	1	2	0.433178314	0.000819303	yes
SLITRK1	INSIDE	Hs.415478	114798	13	83351660	83351876	217	1	2	2	0.403177783	0.003403447	yes
SORCS3	INSIDE	Hs.671950	22986	10	106392367	106393036	670	3	6	6	0.121008199	4.08E-05	yes
SOX17	INSIDE	Hs.98367	64321	8	55533691	55534040	350	2	7	4	0.381895956	2.71E-08	yes
ST8SIA4	INSIDE	Hs.308628	7903	5	100264656	100265360	705	0	4	3	0.308959942	0.005181622	yes
ST8SIA5	INSIDE	Hs.465025	29906	18	42590055	42590342	288	3	4	3	0.176518686	0.000122914	yes

TBX18	INSIDE	Hs.251830	9096	6	85529648	85529984	337	1	4	3	0.59920427	0.000180777	yes
TBX2	INSIDE	Hs.705451	6909	17	56833274	56833487	214	0	1	2	0.27263982	0.000493318	yes
TBX2	INSIDE	Hs.705451	6909	17	56832907	56833273	367	3	3	2	0.416184322	0.001396548	yes
TCF8	INSIDE		NA	10	31649546	31649933	388	0	3	3	0.1253477	0.001539314	yes
TCF8	INSIDE		NA	10	31649152	31649545	394	3	7	4	0.386255467	0.006059859	yes
TFAP2A	INSIDE	Hs.519880	7020	6	10517844	10518835	992	3	5	8	0.326804862	0.000157489	yes
TFAP2A	INSIDE	Hs.519880	7020	6	10519235	10519456	222	2	1	2	0.183529793	0.001122315	yes
TFAP2B	INSIDE	Hs.33102	7021	6	50895623	50896315	693	6	4	5	0.274410831	0.001516025	yes
TFAP2D	INSIDE	Hs.434107	83741	6	50799946	50800517	572	4	3	6	0.183227839	2.99E-05	yes
TLX3	INSIDE	Hs.249125	30012	5	170669046	170670085	1040	10	16	10	0.139087623	0.002701215	yes
TNFRSF19	INSIDE	Hs.149168	55504	13	23051594	23052415	822	9	10	6	0.162147177	0.003411149	yes
UNQ9433	INSIDE	Hs.527111	389658	8	53639991	53640356	366	2	6	3	0.181243241	0.000883902	yes
VAX1	INSIDE	Hs.681703	11023	10	118882137	118882656	520	2	3	5	0.406958175	5.20E-06	yes
WT1	INSIDE	Hs.591980	7490	11	32411131	32412282	1152	8	3	10	0.244420424	4.76E-06	yes
ZIC4	INSIDE	Hs.415766	84107	3	148592274	148593016	743	2	7	8	0.267348233	0.000496453	yes
ZIC4	INSIDE	Hs.415766	84107	3	148590867	148592088	1222	3	4	11	0.069173808	0.006472347	yes
ABLIM2	INSIDE	Hs.233404	84448	4	8148957	8150943	1987	0	7	4	-0.105330023	0.007656599	no
ACCN3	INSIDE	Hs.647113	9311	7	150378759	150379488	730	7	6	7	0.139274342	0.003034798	no
ACTA1	INSIDE	Hs.1288	58	1	227635279	227636223	945	4	7	11	0.160723506	0.001920368	no
ACTN2	INSIDE	Hs.498178	88	1	234916395	234917434	1040	13	7	6	0.097042061	0.002709808	no
ADAMTS10	INSIDE	Hs.657508	81794	19	8563421	8564077	657	3	7	5	0.273824236	0.000872246	no
ADAMTS10	INSIDE	Hs.657508	81794	19	8562639	8563204	566	2	4	6	0.177990387	0.002396764	no
ADAMTS10	INSIDE	Hs.657508	81794	19	8566304	8568158	1855	2	6	4	-0.077342943	0.00251563	no
ADAMTS16	INSIDE	Hs.661915	170690	5	5356082	5357040	959	2	3	5	-0.229501466	5.58E-06	no
ADAMTS2	INSIDE	Hs.591725	9509	5	178480521	178483206	2686	13	20	9	-0.081758921	0.009084842	no
ADD2	INSIDE	Hs.188528	119	2	70848610	70848803	194	2	0	2	0.13726866	0.002348991	no
ADD2	INSIDE	Hs.188528	119	2	70785520	70787340	1821	1	4	4	-0.145378856	0.00319017	no
ADD2	INSIDE	Hs.188528	119	2	70847774	70848609	836	7	12	9	0.075871485	0.005351228	no
AKR1B1	INSIDE	Hs.521212	231	7	133793369	133793696	328	0	1	2	0.168006914	0.000183817	no
AKR1B1	INSIDE	Hs.521212	231	7	133793697	133794416	720	9	8	6	0.104415723	0.001046447	no
ALOX5	INSIDE	Hs.89499	240	10	45258345	45259784	1440	5	17	15	-0.120672623	0.004751215	no
ALX3	INSIDE	Hs.669953	257	1	110412730	110413276	547	6	6	5	0.2236397	1.19E-06	no
ANKRD15	INSIDE	Hs.306764	23189	9	697005	697461	457	2	11	3	0.128427517	0.005151278	no
ARHGEF10	INSIDE	Hs.98594	9639	8	1784738	1786276	1539	8	9	3	-0.145538497	0.00347605	no
ARHGEF4	INSIDE	Hs.469935	50649	2	131436455	131437264	810	0	2	1	0.510332112	1.01E-07	no
ARID3A	INSIDE	Hs.501296	1820	19	883736	884280	545	2	5	2	-0.194729334	0.000189944	no
ARSI	INSIDE	Hs.591252	340075	5	149657134	149658765	1632	3	9	9	-0.110839274	0.006577191	no
ASMTL	INSIDE	Hs.533514	8623	Y	1510824	1512147	1324	2	8	3	-0.083429299	0.009489032	no
ATCAY	INSIDE	Hs.418055	85300	19	3858477	3859012	536	1	6	5	-0.195330251	0.004960091	no
ATP8A2	INSIDE	Hs.444957	51761	13	24844383	24845256	874	15	6	4	0.143016201	0.001308401	no
BAPX1	INSIDE		NA	4	13152424	13153040	617	4	6	4	0.283427392	0.002425543	no
BARHL1	INSIDE	Hs.283809	56751	9	134451623	134453582	1960	9	15	12	0.093698041	0.006448439	no
BARHL2	INSIDE	Hs.451956	343472	1	90954470	90954907	438	0	1	3	0.250747575	0.000496453	no
BCL11A	INSIDE	Hs.370549	53335	2	60631309	60631696	388	5	7	4	-0.089494706	0.009124803	no
BCL11B	INSIDE	Hs.699440	64919	14	98782381	98783387	1007	7	6	9	0.119461202	0.000126537	no
BNIP3	INSIDE	Hs.144873	664	10	133644247	133644647	401	1	3	3	0.249661381	0.00274541	no
BRSK1	INSIDE	Hs.182081	84446	19	60507316	60509055	1740	2	13	6	-0.076773686	0.000962275	no
BRSK1	INSIDE	Hs.182081	84446	19	60504602	60506227	1626	5	9	4	-0.115799877	0.002490498	no
BRUNOL4	INSIDE	Hs.435976	56853	18	33170755	33171872	1118	0	8	2	-0.112393935	0.002630662	no
C10orf90	INSIDE	Hs.587663	118611	10	128182856	128183899	1044	2	3	5	-0.108648124	0.005197691	no
C14orf115	INSIDE	Hs.578167	55237	14	73893003	73894440	1438	0	9	5	-0.118713657	0.006583847	no
C15orf26	INSIDE	Hs.130979	161502	15	79213504	79214216	713	2	3	2	0.400769586	0.001096619	no
C1orf159	INSIDE	Hs.235095	54991	1	1035612	1036430	819	1	2	5	-0.164130602	0.001816431	no
C1orf164	INSIDE	Hs.456557	55182	1	44655615	44656793	1179	15	7	12	0.091271562	0.001368137	no
C1QC	INSIDE	Hs.467753	714	1	22845919	22847092	1174	2	6	6	-0.165945883	0.000960294	no
C2orf32	INSIDE		NA	2	68399880	68400390	511	8	6	4	0.204261865	4.35E-07	no
C2orf32	INSIDE		NA	2	68399620	68399879	260	1	1	1	0.315184879	0.001551343	no
C3orf15	INSIDE	Hs.341906	89876	3	120904331	120905214	884	5	3	6	0.155602824	0.002883096	no
C8orf42	INSIDE	Hs.289293	157695	8	484143	484402	260	6	5	3	0.198481326	0.007980063	no
CACNA1B	INSIDE	Hs.495522	774	9	139896586	139897833	1248	0	2	5	-0.27423962	4.80E-07	no
CACNA1C	INSIDE	Hs.118262	775	12	2669931	2670719	789	5	1	5	0.127463963	0.005256117	no
CACNA1E	INSIDE	Hs.437444	777	1	180033984	180034988	1005	2	2	7	-0.155040859	0.002837492	no
CACNA1G	INSIDE	Hs.591169	8913	17	46003261	46004558	1298	0	7	2	-0.107300211	0.001418014	no
CAMK2B	INSIDE	Hs.351887	816	7	44315999	44316275	277	6	3	2	0.222902576	0.000301306	no
CAMK2B	INSIDE	Hs.351887	816	7	44315474	44315998	525	4	2	4	0.239442386	0.001343455	no
CAMK2B	INSIDE	Hs.351887	816	7	44316276	44316627	352	1	1	1	0.174614097	0.003043804	no
CAMTA1	INSIDE	Hs.397705	23261	1	7645049	7646660	1612	1	7	8	-0.141626484	0.001819565	no
CAMTA1	INSIDE	Hs.397705	23261	1	7044880	7045625	746	2	5	4	-0.117017864	0.003180663	no
CASP6	INSIDE	Hs.654616	839	4	110842893	110843629	737	2	4	2	0.24198276	0.001435892	no
CBS	INSIDE	Hs.533013	875	21	43353431	43355850	2420	4	3	5	-0.100885696	0.006450637	no
CCKBR	INSIDE	Hs.203	887	11	6247294	6249677	2384	8	11	12	-0.084331963	0.004621045	no
CCL28	INSIDE	Hs.656904	56477	5	43432537	43432911	375	4	1	2	0.359321992	0.003756626	no
CD163L1	INSIDE	Hs.631727	283316	12	7483471	7484059	589	4	2	2	0.349550787	0.00030786	no
CDH22	INSIDE	Hs.472861	64405	20	44263099	44263780	682	2	2	2	0.142068186	0.006370553	no
CDH4	INSIDE	Hs.473231	1002	20	59805355	59807463	2109	4	13	4	-0.09798662	0.000725924	no
CDH4	INSIDE	Hs.473231	1002	20	59888572	59890241	1670	1	4	7	-0.098285709	0.002969123	no
CDH8	INSIDE	Hs.368322	1006	16	60626281	60626742	462	1	4	2	0.378434065	0.00055215	no
CDO1	INSIDE	Hs.442378	1036	5	115179445	115180280	836	8	6	10	0.170809781	4.88E-05	no
CDO1	INSIDE	Hs.442378	1036	5	115179148	115179385	238	1	2	2	0.307948054	9.44E-05	no
CHRM2	INSIDE	Hs.535891	1129	7	136206043	136206786	744	5	4	7	0.08701419	0.000952371	no
CHRNB2	INSIDE	Hs.2306	1141	1	152809800	152811658	1859	4	8	8	-0.115866724	0.000154518	no
CHST1	INSIDE	Hs.104576	8534	11	45628375	45629314	940	7	10	7	-0.09230428	0.003754112	no
CITED1	INSIDE	Hs.40403	4435	X	71440923	71441838	916	3	3	4	0.095672249	0.009761007	no
COL14A1	INSIDE	Hs.409662	7373	8	121206634	121207130	497	4	1	4	0.533074431	2.07E-05	no
COL4A2	INSIDE	Hs.508716	1284	13	109758900	109759172	273	1	3	2	0.239721699	0.005351228	no
COL5A1	INSIDE	Hs.210283	1289	9	136799479	136800312	834	0	4	1	-0.226951797	0.000372492	no
CPLX1	INSIDE	Hs.478930	10815	4	801445	802865	1421	2	5	5	-0.144992952	0.002538893	no
CREB3L1	INSIDE	Hs.405961	90993	11	46273495	46273871	377	3	8	4	0.171305778	0.000514422	no
CREB3L1	INSIDE	Hs.405961	90993	11	46273926	46274101	176	1	2	1	0.250763992	0.000960679	no
CREB3L1	INSIDE	Hs.405961	90993	11	46272787	46273494	708	1	5	1	0.260752526	0.003019475	no

CTBP2	INSIDE	Hs.501345	1488	10	126701754	126702342	589	2	3	4	0.188913062	0.00074159	no
CTDP1	INSIDE	Hs.465490	9150	18	75579898	75581610	1713	2	3	4	-0.154313781	0.003355313	no
CXorf40A	INSIDE	Hs.654738	91966	X	148429752	148431099	1348	7	10	8	-0.068916884	0.008554205	no
CYP1B1	INSIDE	Hs.154654	1545	2	38154686	38156344	1659	19	12	18	0.087608648	0.000222251	no
CYP1B1	INSIDE	Hs.154654	1545	2	38156345	38156558	214	1	3	2	0.180014653	0.005468178	no
CYP26B1	INSIDE	Hs.91546	56603	2	72226954	72227204	251	3	2	2	0.153094895	0.000237189	no
DAB1	INSIDE	Hs.477370	1600	1	58487777	58487985	209	2	2	1	0.334271652	0.006490823	no
DDI1	INSIDE	Hs.591941	414301	11	103412423	103413497	1075	1	5	8	-0.102069915	0.003352734	no
DIP	INSIDE	Hs.475150	23151	22	45432009	45434443	2435	5	6	4	-0.076752095	0.007450458	no
DIRAS3	INSIDE	Hs.194695	9077	1	68284866	68285830	965	3	3	7	-0.119273178	0.007050257	no
DKK2	INSIDE	Hs.211869	27123	4	108175672	108176576	905	4	3	7	0.160659447	0.002268245	no
DLGAP2	INSIDE	Hs.113287	9228	8	1603625	1604596	972	3	2	6	-0.125955074	0.006041245	no
DLGAP3	INSIDE	Hs.436393	58512	1	35106213	35107393	1181	4	5	3	-0.144489653	0.000666064	no
DLX5	INSIDE	Hs.99348	1749	7	96489289	96489641	353	2	1	4	0.609380367	1.14E-06	no
DLX5	INSIDE	Hs.99348	1749	7	96491009	96492104	1096	4	7	8	0.171730262	0.002768331	no
DMRTA1	INSIDE	Hs.371976	63951	9	22436822	22437832	1011	9	10	10	0.085750699	0.001568229	no
DNAH2	INSIDE	Hs.367649	146754	17	7630117	7631483	1367	4	6	6	-0.117351166	0.004771909	no
DNAJC6	INSIDE	Hs.647643	9829	1	65503952	65504262	311	3	5	3	0.18060214	3.27E-05	no
DOK5	INSIDE	Hs.656582	55816	20	52526331	52526731	401	0	2	2	0.5803196	4.86E-05	no
DOK5	INSIDE	Hs.656582	55816	20	52525907	52526330	424	4	8	5	0.220899061	0.003220319	no
DPP6	INSIDE	Hs.490684	1804	7	153215429	153215630	202	1	0	2	0.193318355	0.002725184	no
EBF3	INSIDE	Hs.699395	253738	10	131527586	131529127	1542	3	3	6	-0.122875835	0.000350219	no
ECAT1	INSIDE		NA	6	74128844	74130342	1499	4	15	5	-0.161610121	5.13E-07	no
EDG1	INSIDE	Hs.154210	1901	1	101474991	101475322	332	5	2	3	0.362053633	0.000573055	no
EDIL3	INSIDE	Hs.482730	10085	5	83715180	83716089	910	5	9	9	0.083776371	0.007178796	no
EFHD1	INSIDE	Hs.516769	80303	2	233207402	233207758	357	4	4	3	0.458657801	1.28E-05	no
EFHD1	INSIDE	Hs.516769	80303	2	233206475	233207382	908	10	12	10	0.076010256	0.005356	no
ELAVL2	INSIDE	Hs.166109	1993	9	23810620	23811016	397	8	4	4	0.358357322	9.84E-07	no
ELAVL2	INSIDE	Hs.166109	1993	9	23812343	23812659	317	1	2	3	0.577425641	6.85E-06	no
EMX1	INSIDE	Hs.516090	2016	2	72999972	73000472	501	0	1	5	0.206986074	0.000872246	no
EMX1	INSIDE	Hs.516090	2016	2	73000510	73001250	741	6	9	8	0.147985477	0.001002635	no
EPHA2	INSIDE	Hs.171596	1969	1	16347297	16348620	1324	0	5	4	-0.140347912	0.000779966	no
EPS8L1	INSIDE	Hs.438862	54869	19	60285194	60285968	775	6	6	9	0.119706172	0.000851189	no
ESRRB	INSIDE	Hs.435845	2103	14	76027230	76028571	1342	1	3	4	-0.109038399	0.006905209	no
EVX1	INSIDE	Hs.369879	2128	7	27250317	27251641	1325	4	5	6	0.12822805	0.004038109	no
EVX2	INSIDE	Hs.452341	344191	2	176656342	176656761	420	2	3	4	0.261286978	1.62E-06	no
EVX2	INSIDE	Hs.452341	344191	2	176655807	176656341	535	3	4	5	0.317941262	2.54E-05	no
F7	INSIDE	Hs.36989	2155	13	112818804	112821405	2602	9	9	9	-0.063859915	0.002121418	no
FAM101B	INSIDE	Hs.345588	359845	17	292783	293720	938	3	4	8	-0.125655211	0.001805579	no
FAM43A	INSIDE	Hs.435080	131583	3	195889441	195890545	1105	5	7	12	0.154545741	1.40E-06	no
FAT4	INSIDE	Hs.702217	79633	4	126457745	126458410	666	8	3	7	0.266945569	1.34E-05	no
FBXL7	INSIDE	Hs.433057	23194	5	15980972	15981843	872	3	5	7	-0.106153117	0.006370553	no
FBXO17	INSIDE	Hs.531770	115290	19	44157555	44157881	327	1	1	2	0.207054968	0.005258851	no
FGF12	INSIDE	Hs.584758	2257	3	193714744	193714964	221	4	1	1	0.169596145	0.002790858	no
FGF6	INSIDE	Hs.166015	2251	12	4424059	4425028	970	1	4	4	-0.176393772	0.000413409	no
FHOD3	INSIDE	Hs.436636	80206	18	32594424	32595317	894	3	4	6	-0.135478143	0.003641277	no
FLJ42258	INSIDE		NA	11	69177902	69178942	1041	5	8	7	-0.098931768	0.003819289	no
FLT1	INSIDE	Hs.654360	2321	13	27965778	27966070	293	0	1	2	0.298020942	4.73E-06	no
FNBP1	INSIDE	Hs.189409	23048	9	131843862	131844333	472	0	4	3	0.127324573	0.007049256	no
FNDC1	INSIDE	Hs.520525	84624	6	159572889	159574259	1371	4	9	12	-0.086742393	0.009819661	no
FOXB1	INSIDE	Hs.160375	27023	15	58084790	58085242	453	4	3	5	0.267274372	0.004485229	no
FOXB1	INSIDE	Hs.160375	27023	15	58084131	58084789	659	4	7	6	0.241390107	0.005026029	no
FOXD4L4	INSIDE	Hs.534644	349334	9	69666954	69668217	1264	6	8	1	0.316727799	0.00899833	no
FOXE1	INSIDE	Hs.159234	2304	9	99655303	99657553	2251	29	27	21	0.063409502	0.00416252	no
FOXP4	INSIDE	Hs.131436	116113	6	41669203	41669468	266	0	2	2	0.22389843	0.001278804	no
GABRB3	INSIDE	Hs.302352	2562	15	24424684	24426060	1377	3	5	8	-0.08228402	0.006109377	no
GAD1	INSIDE	Hs.420036	2571	2	171384668	171384958	291	2	1	2	0.778534948	5.76E-06	no
GAD1	INSIDE	Hs.420036	2571	2	171384959	171385825	867	1	5	3	0.382669811	7.45E-05	no
GAD1	INSIDE	Hs.420036	2571	2	171387929	171388443	515	4	4	4	0.173845557	0.000160529	no
GAD1	INSIDE	Hs.420036	2571	2	171386727	171387748	1022	7	5	12	0.108300676	0.000439046	no
GAD1	INSIDE	Hs.420036	2571	2	171387749	171387913	165	0	1	1	0.369535762	0.00046151	no
GALNT14	INSIDE	Hs.468058	79623	2	31213267	31214163	897	5	9	5	0.108576208	0.007685519	no
GALR2	INSIDE	Hs.666366	8811	17	71583855	71585183	1329	18	9	13	0.121300678	7.02E-05	no
GBX2	INSIDE	Hs.184945	2637	2	236739143	236739947	805	3	4	8	0.186853783	0.004350111	no
GDF7	INSIDE	Hs.447688	151449	2	20733265	20733756	492	5	3	4	0.349806817	6.81E-05	no
GIMAP1	INSIDE	Hs.647087	170575	7	150048039	150048979	941	5	12	8	-0.077178034	0.002257179	no
GLI2	INSIDE	Hs.111867	2736	2	121458300	121459587	1288	0	6	5	-0.079385749	0.007370246	no
GLI3	INSIDE	Hs.21509	2737	7	41970398	41971895	1498	3	9	4	-0.097405083	0.001328096	no
GLRA3	INSIDE	Hs.413099	8001	4	175986448	175986972	525	0	2	2	0.205280632	6.06E-05	no
GMEB2	INSIDE	Hs.473286	26205	20	61693654	61693943	290	1	1	3	0.218754868	0.007188058	no
GNG7	INSIDE	Hs.515544	2788	19	2539447	2539943	497	1	2	2	-0.134932127	0.000829303	no
GP5	INSIDE	Hs.73734	2814	3	195599862	195600519	658	8	2	5	0.151671639	0.003248399	no
GPR103	INSIDE	Hs.368977	84109	4	122520874	122521239	366	3	4	3	0.433898209	4.12E-05	no
GRIA2	INSIDE	Hs.32763	2891	4	158363334	158363747	414	4	2	2	0.251216485	0.000746793	no
GRIA2	INSIDE	Hs.32763	2891	4	158361964	158363143	1180	6	7	4	0.071011434	0.008282141	no
GRID2	INSIDE	Hs.480281	2895	4	93445289	93445993	705	8	6	7	0.206336635	0.003352734	no
GRIK2	INSIDE	Hs.98262	2898	6	101956628	101957003	376	3	1	2	0.27290252	1.29E-05	no
GRIK2	INSIDE	Hs.98262	2898	6	101956153	101956627	475	1	1	2	0.114545078	0.000859643	no
GRIK5	INSIDE	Hs.367799	2901	19	47201076	47202193	1118	1	3	4	-0.201288498	1.13E-05	no
GRM1	INSIDE	Hs.32945	2911	6	146797349	146797780	432	1	1	3	0.670201918	2.63E-05	no
GRM4	INSIDE	Hs.654847	2914	6	34110402	34112528	2127	1	7	8	-0.089803369	0.001146248	no
GRM6	INSIDE	Hs.248131	2916	5	178345011	178346685	1675	1	7	6	-0.138557931	0.000232548	no
GSH2	INSIDE		NA	4	54661583	54662079	497	7	1	3	0.221139291	0.002448761	no
GSH2	INSIDE		NA	4	54662131	54662284	154	2	0	1	0.214508971	0.002969754	no
GSH2	INSIDE		NA	4	54662285	54663062	778	3	5	6	0.14352091	0.005758777	no
GSTM3	INSIDE	Hs.2006	2947	1	110083813	110084167	355	2	4	3	0.499909137	0.000223389	no
GUCY2D	INSIDE	Hs.592109	3000	17	7850034	7851886	1853	4	10	6	-0.085526213	0.000222251	no
H1FNT	INSIDE	Hs.155833	341567	12	47009030	47010485	1456	5	9	9	-0.084909072	0.004025904	no
HAND2	INSIDE	Hs.388245	9464	4	174687674	174687977	304	3	1	3	0.334737333	2.97E-07	no
HCRTR2	INSIDE	Hs.151624	3062	6	55146932	55147429	498	1	7	4	0.16290017	0.009819661	no

HECW1	INSIDE	Hs.164453	23072	7	43450153	43451761	1609	8	5	10	-0.088718229	0.006965205	no
HFE2	INSIDE	Hs.632436	148738	1	144125899	144127482	1584	2	10	6	0.092110282	0.00445448	no
HIST1H2BO	INSIDE	Hs.673856	8348	6	27969084	27969846	763	0	5	2	-0.107485705	0.006617669	no
HIST1H4C	INSIDE	Hs.46423	8364	6	26212112	26212330	219	2	3	1	-0.201525231	0.007214219	no
HIST1H4F	INSIDE	Hs.247816	8361	6	26348608	26348808	201	2	0	1	0.31965963	0.005200711	no
HLX1	INSIDE		NA	1	219123784	219124422	639	2	3	4	0.158805125	0.002746584	no
HOXA3	INSIDE	Hs.659337	3200	7	27112288	27113142	855	5	8	6	0.216425468	0.000189944	no
HOXD10	INSIDE	Hs.123070	3236	2	176690128	176690928	801	2	7	6	0.153958513	0.001679556	no
HS3ST2	INSIDE	Hs.115830	9956	16	22833613	22834161	549	0	2	3	-0.310664462	0.000935156	no
HTR1B	INSIDE	Hs.123016	3351	6	78228711	78229028	318	1	1	1	0.759498184	0.000134923	no
IGF2BP1	INSIDE	Hs.144936	10642	17	44467655	44468368	714	1	7	2	-0.200219277	0.000129518	no
IGF2BP1	INSIDE	Hs.144936	10642	17	44468397	44468975	579	1	2	2	-0.179762142	0.000279306	no
IGSF21	INSIDE	Hs.212511	84966	1	18563674	18565296	1623	0	3	4	-0.152791364	0.000520851	no
INPP5D	INSIDE	Hs.262886	3635	2	233633299	233634048	750	1	4	4	-0.211568502	0.002770604	no
ITGB1BP3	INSIDE	Hs.135458	27231	19	3892430	3893411	982	4	6	4	-0.114467873	0.001441839	no
JPH3	INSIDE	Hs.592068	57338	16	86270678	86273505	2828	17	29	3	-0.208580351	0.007532109	no
KALRN	INSIDE	Hs.8004	8997	3	125469897	125470866	970	1	5	5	-0.137591537	0.000955293	no
KATNAL2	INSIDE	Hs.404137	83473	18	42780750	42781032	283	1	2	3	0.739544769	0.001179834	no
KCND3	INSIDE	Hs.666367	3752	1	112325519	112327059	1541	4	11	11	-0.12866935	0.000166165	no
KCNG2	INSIDE	Hs.247905	26251	18	75743592	75744482	891	17	13	7	-0.133794748	0.009250043	no
KCNG4	INSIDE	Hs.335877	93107	16	82813016	82814602	1587	1	6	5	-0.143156186	4.71E-06	no
KCNG4	INSIDE	Hs.335877	93107	16	82827743	82828763	1021	3	6	7	-0.080038173	0.006699831	no
KCNH2	INSIDE	Hs.647099	3757	7	150278713	150280024	1312	6	1	10	-0.121695836	0.002990016	no
KCNJ9	INSIDE	Hs.66726	3765	1	158320327	158321641	1315	16	2	12	0.060248058	0.00544958	no
KCNK17	INSIDE	Hs.162282	89822	6	39379205	39380080	876	3	4	5	-0.174107856	1.63E-05	no
KCNN1	INSIDE	Hs.158173	3780	19	17938206	17938808	603	0	4	1	0.216047149	0.004031297	no
KCNN1	INSIDE	Hs.158173	3780	19	17944997	17946341	1345	0	15	6	-0.097926916	0.004886366	no
KHDRBS2	INSIDE	Hs.519794	202559	6	63053525	63054065	541	8	3	3	0.782682147	4.88E-07	no
KIAA1024	INSIDE	Hs.301654	23251	15	77512399	77512757	359	3	1	3	0.412036138	1.60E-06	no
KIAA1026	INSIDE		NA	1	15000095	15001840	1746	2	6	5	-0.127425666	0.005190575	no
KIAA1409	INSIDE	Hs.126561	57578	14	92966136	92966711	576	2	7	4	0.126307426	0.001620105	no
KIAA1529	INSIDE	Hs.435629	57653	9	99109771	99109935	165	2	1	1	0.364743304	0.000753552	no
KIAA1683	INSIDE	Hs.313471	80726	19	18229151	18230008	858	5	8	6	-0.14849381	0.002863583	no
KIAA1688	INSIDE	Hs.148988	80728	8	145725576	145725840	265	0	2	1	0.207008224	0.001000272	no
KIAA1804	INSIDE	Hs.547779	84451	1	231564052	231564546	495	1	2	3	0.167667789	0.009250043	no
KIAA2022	INSIDE	Hs.124128	340533	X	74060551	74060904	354	6	2	3	0.239141941	0.000966177	no
KIF5C	INSIDE	Hs.435557	3800	2	149350149	149350735	587	6	1	3	0.181501464	0.003580796	no
KLF11	INSIDE	Hs.12229	8462	2	10101772	10102212	441	4	4	5	-0.158306085	0.003634636	no
KLF6	INSIDE	Hs.4055	1316	10	3813896	3814546	651	0	4	3	-0.285990175	8.68E-05	no
KNDC1	INSIDE	Hs.530685	85442	10	134868137	134869161	1025	3	7	6	-0.09900734	0.000864185	no
KNDC1	INSIDE	Hs.530685	85442	10	134866254	134867149	896	1	7	5	-0.107090297	0.00315852	no
KRT72	INSIDE	Hs.662013	140807	12	51280989	51281584	686	4	5	5	0.169888991	0.003700594	no
LBX2	INSIDE	Hs.554975	85474	2	74580131	74581028	898	4	11	5	0.180773602	0.000344852	no
LEPR	INSIDE	Hs.705413	3953	1	65763498	65764585	1088	12	9	11	0.072954673	0.000218338	no
LHPP	INSIDE	Hs.527748	64077	10	126270944	126272600	1657	3	7	4	-0.135878134	6.95E-05	no
LHX2	INSIDE	Hs.696425	9355	9	125817026	125817732	707	4	4	7	0.140947945	0.002209432	no
LHX2	INSIDE	Hs.696425	9355	9	125818717	125818885	169	1	1	1	0.167692106	0.003635224	no
LHX2	INSIDE	Hs.696425	9355	9	125817733	125818195	463	1	4	5	0.193028279	0.007290906	no
LHX2	INSIDE	Hs.696425	9355	9	125818886	125819237	352	1	2	3	0.23142512	0.008049269	no
LHX8	INSIDE	Hs.403934	431707	1	75373161	75373560	400	2	4	4	0.343308485	0.001028072	no
LHX8	INSIDE	Hs.403934	431707	1	75368451	75369567	1117	4	6	7	0.119031429	0.004675829	no
LHX8	INSIDE	Hs.403934	431707	1	75373683	75373955	273	2	1	2	0.165674837	0.008474019	no
LHX9	INSIDE	Hs.442578	56956	1	196156919	196157697	779	3	3	6	0.235682084	0.006450908	no
LIFR	INSIDE	Hs.133421	3977	5	38593163	38593346	184	1	1	1	0.464533099	0.000267564	no
LMX1A	INSIDE	Hs.667312	4009	1	163590004	163590555	552	0	5	5	0.406418279	3.37E-05	no
LMX1B	INSIDE	Hs.133709	4010	9	128427128	128427399	272	2	2	2	0.373959335	0.001709081	no
LOC161247	INSIDE	Hs.128060	161247	14	23671117	23672302	1186	1	4	5	-0.097202568	0.002813217	no
LOC197322	INSIDE		NA	16	87713463	87715413	1951	1	10	6	-0.106580582	0.005230602	no
LOC389257	INSIDE	Hs.683662	389257	5	245791	248617	2827	1	11	5	-0.129076746	0.000604506	no
LOC389936	INSIDE	Hs.532673	389936	10	9488799	9490507	1709	0	2	2	-0.216356806	0.002750732	no
LOC441177	INSIDE	Hs.701661	441177	6	166322221	166322668	448	2	3	4	0.237265864	3.39E-05	no
LOC51057	INSIDE	Hs.414952	51057	2	63668463	63668798	336	0	2	1	-0.116616126	0.001170048	no
LPHN2	INSIDE	Hs.24212	23266	1	82040612	82040846	235	1	3	1	0.557798994	0.001472564	no
LRP1	INSIDE	Hs.162757	4035	12	55865218	55866900	1683	2	8	6	-0.085232648	0.001998775	no
LRRTM1	INSIDE	Hs.591580	347730	2	80383828	80384109	282	2	2	3	0.582245936	1.34E-07	no
LRRTM1	INSIDE	Hs.591580	347730	2	80382765	80383827	1063	3	5	7	0.285126888	1.76E-07	no
LRRTM1	INSIDE	Hs.591580	347730	2	80384192	80384764	573	1	3	2	0.124879902	0.005386197	no
MAEA	INSIDE	Hs.139896	10296	4	1310008	1311251	1244	3	7	4	-0.176697459	0.009177566	no
MAL	INSIDE	Hs.80395	4118	2	95055153	95055765	613	8	2	5	0.241245939	0.000403305	no
MAP1D	INSIDE	Hs.298250	254042	2	172653207	172653417	211	4	2	1	0.387409931	0.000200879	no
MCF2L2	INSIDE	Hs.584868	23101	3	184627867	184628348	482	2	2	4	0.139668945	0.007188058	no
MDGA1	INSIDE	Hs.437993	266727	6	37733380	37734403	1024	1	4	4	-0.162712402	6.02E-05	no
MGC2752	INSIDE	Hs.541177	65996	19	63784332	63784988	657	4	2	6	0.2000449	0.001404546	no
MLX	INSIDE	Hs.383019	6945	17	37973004	37973218	215	3	3	2	-0.08538977	0.005230602	no
MOCS3	INSIDE	Hs.159410	27304	20	49009353	49009951	599	1	1	2	0.175192397	0.001747745	no
MOS	INSIDE	Hs.533432	4342	8	57188007	57188576	570	5	2	6	0.20422424	0.000163975	no
MPPED1	INSIDE	Hs.592198	758	22	42160622	42162355	1734	2	6	5	-0.110269857	0.009061734	no
MRC2	INSIDE	Hs.7835	9902	17	58097606	58098774	1169	3	7	7	-0.138497653	0.001102898	no
MRGPRF	INSIDE	Hs.118513	219928	11	68528888	68530501	1614	3	15	12	-0.089101532	0.001593919	no
MST1R	INSIDE	Hs.517973	4486	3	49913929	49916147	2219	6	9	9	-0.096051696	0.000322022	no
MTL5	INSIDE	Hs.145932	9633	11	68273899	68274638	740	6	5	6	0.158150173	0.00182399	no
MTNR1B	INSIDE	Hs.569039	4544	11	92342900	92343214	315	3	2	2	0.148835378	0.006697629	no
MTNR1B	INSIDE	Hs.569039	4544	11	92342387	92342899	513	6	6	6	0.092027036	0.008049269	no
MYH7B	INSIDE	Hs.414122	57644	20	33048510	33049142	633	6	1	5	0.247930185	0.007176343	no
MYO5A	INSIDE	Hs.21213	4644	15	50607557	50607859	303	1	3	2	0.484423363	0.003034798	no
MYOM2	INSIDE	Hs.443683	9172	8	2024997	2025487	491	2	2	5	-0.289703932	0.001080395	no
MYOM2	INSIDE	Hs.443683	9172	8	1991213	1992080	868	2	2	3	-0.076379551	0.005170204	no
MYT1L	INSIDE	Hs.434418	23040	2	1874653	1875966	1314	11	5	4	-0.063184265	0.008999135	no
NAP1L3	INSIDE	Hs.21365	4675	X	92814941	92815298	358	2	3	1	0.735232961	0.00498312	no
NARS2	INSIDE	Hs.503389	79731	11	77962800	77963048	249	1	0	1	-0.223488823	0.00315852	no

NCAPH	INSIDE	Hs.308045	23397	2	96365265	96365637	373	3	6	4	0.07600259	0.008222686	no
NFATC1	INSIDE	Hs.534074	4772	18	75302877	75305568	2692	5	16	16	-0.067654207	0.002016513	no
NFATC1	INSIDE	Hs.534074	4772	18	75386891	75387680	790	1	3	5	-0.099219784	0.008830576	no
NFIL3	INSIDE	Hs.79334	4783	9	93223133	93223937	805	11	10	7	0.1437944	0.000163975	no
NLGN4X	INSIDE	Hs.21107	57502	X	6155368	6155883	516	3	4	3	0.458916242	3.65E-06	no
NLGN4Y	INSIDE	Hs.439199	22829	Y	15450776	15452116	1341	2	5	6	-0.195061451	0.000193077	no
NLRP4	INSIDE	Hs.631533	147945	19	61060810	61061701	892	0	5	5	-0.288814331	3.19E-06	no
NMNAT2	INSIDE	Hs.497123	23057	1	181653501	181654183	683	2	5	6	0.244066293	0.00187407	no
NO145	INSIDE		NA	6	10995067	10995707	641	4	4	6	0.426832451	1.66E-06	no
NODAL	INSIDE	Hs.370414	4838	10	71869679	71870523	845	7	6	6	0.11688916	0.000285807	no
NOVA1	INSIDE	Hs.31588	4857	14	26136078	26136590	513	4	5	4	0.079949916	0.005758777	no
NR4A2	INSIDE	Hs.563344	4929	2	156894410	156894673	264	0	3	3	0.321895002	0.003400297	no
NR5A2	INSIDE	Hs.33446	2494	1	198271506	198272303	798	2	7	1	0.739962738	5.75E-08	no
NR5A2	INSIDE	Hs.33446	2494	1	198270992	198271505	514	4	1	5	0.391170484	0.000225734	no
NR5A2	INSIDE	Hs.33446	2494	1	198278322	198278692	371	4	6	3	-0.115914247	0.005929889	no
NRP2	INSIDE	Hs.471200	8828	2	206259443	206259885	443	1	4	2	0.554217138	6.51E-06	no
NRXN1	INSIDE	Hs.637685	9378	2	50427973	50428721	749	4	5	4	0.223803696	0.000212728	no
NTNG1	INSIDE	Hs.657434	22854	1	107485076	107485290	215	0	1	1	0.24242939	0.003690434	no
NTNG2	INSIDE	Hs.163642	84628	9	134062619	134064589	1971	6	10	7	-0.082579714	0.003285978	no
NXPH1	INSIDE	Hs.487564	30010	7	8448709	8449217	509	4	4	5	0.598710138	3.55E-06	no
NXPH1	INSIDE	Hs.487564	30010	7	8449861	8450153	293	0	1	2	0.725020607	1.94E-05	no
NXPH1	INSIDE	Hs.487564	30010	7	8448309	8448708	400	4	4	4	0.362175072	2.26E-05	no
NXPH1	INSIDE	Hs.487564	30010	7	8449492	8449860	369	1	1	3	0.430957376	0.000267517	no
NXPH1	INSIDE	Hs.487564	30010	7	8440656	8442272	1617	10	10	12	0.089831199	0.001993587	no
OCA2	INSIDE	Hs.654411	4948	15	25921273	25923550	2278	13	6	8	-0.064698423	0.004181737	no
ODZ3	INSIDE	Hs.130438	55714	4	183606154	183606310	157	1	2	1	0.239400935	0.002282969	no
OLIG1	INSIDE	Hs.56663	116448	21	33365415	33365966	552	3	5	7	0.195498008	0.000258186	no
OLIG1	INSIDE	Hs.56663	116448	21	33366218	33366414	197	1	2	2	0.142747939	0.002348991	no
OSR1	INSIDE	Hs.123933	130497	2	19420163	19420374	212	0	4	2	0.713837788	0.000398305	no
OTOF	INSIDE	Hs.91608	9381	2	26579869	26580513	645	3	6	5	-0.094854535	0.008520992	no
OTX1	INSIDE	Hs.445340	5013	2	63134439	63135016	578	5	2	6	0.535529564	0.000556161	no
PABPC5	INSIDE	Hs.246590	140886	X	90576238	90576800	563	7	4	5	0.093679627	0.002813217	no
PALM2	INSIDE		NA	9	111443075	111443347	273	1	1	2	0.52610654	8.10E-06	no
PAX5	INSIDE	Hs.654464	5079	9	36992352	36993066	715	4	4	7	0.242179882	3.52E-06	no
PAX5	INSIDE	Hs.654464	5079	9	37015371	37015632	262	1	2	2	0.392983573	4.16E-05	no
PAX7	INSIDE	Hs.113253	5081	1	18831735	18832186	452	4	5	5	0.128586786	0.009620502	no
PCBP3	INSIDE	Hs.474049	54039	21	46157635	46159773	2139	2	10	8	-0.074061669	0.008545802	no
PCDH10	INSIDE	Hs.192859	57575	4	134291090	134291971	882	7	5	8	0.392296002	9.21E-07	no
PCDH10	INSIDE	Hs.192859	57575	4	134291972	134293255	1284	10	11	12	0.129585898	0.000455933	no
PCDH11Y	INSIDE	Hs.661308	83259	Y	4930831	4931217	387	3	3	3	0.307452017	0.001732497	no
PCDH11Y	INSIDE	Hs.661308	83259	Y	4930527	4930830	304	3	0	1	0.430607906	0.002273691	no
PCDH11Y	INSIDE	Hs.661308	83259	Y	4928286	4928768	483	2	3	4	0.189240615	0.008686991	no
PCDH7	INSIDE	Hs.479439	5099	4	30333351	30333962	612	1	2	6	0.21253948	0.001409542	no
PCDHA12	INSIDE	Hs.608548	56137	5	140235201	140235618	418	3	2	1	0.369169493	0.001042732	no
PCDHA4	INSIDE		NA	5	140167334	140167741	408	1	2	1	0.324462146	0.006841222	no
PCDHGA11	INSIDE		NA	5	140782808	140783463	656	3	1	1	0.264169225	0.00216999	no
PCDHGA12	INSIDE		NA	5	140790888	140791603	716	6	4	9	0.318990943	0.000105163	no
PCDHGA12	INSIDE		NA	5	140790472	140790820	349	4	5	1	0.45179548	0.003595278	no
PCDHGA5	INSIDE		NA	5	140724507	140725256	750	3	5	4	0.231689469	0.003516843	no
PDE3A	INSIDE	Hs.591150	5139	12	20595715	20597296	1582	4	16	8	-0.104849213	0.00644499	no
PDE6B	INSIDE	Hs.59872	5158	4	636712	637563	852	1	2	4	-0.149700949	0.00228026	no
PDGFRA	INSIDE	Hs.74615	5156	4	54793299	54793821	523	5	1	2	0.18887406	6.68E-06	no
PDGFRA	INSIDE	Hs.74615	5156	4	54792733	54793298	566	3	5	5	0.210763929	0.000200879	no
PEMT	INSIDE	Hs.287717	10400	17	17405341	17406294	954	1	8	5	-0.232039786	0.002076902	no
PGBD5	INSIDE	Hs.520463	79605	1	228559111	228560125	1015	0	3	5	-0.170985696	0.000150413	no
PHF21B	INSIDE	Hs.254097	112885	22	43690628	43692659	2032	2	8	5	-0.104954588	0.003615224	no
PITX2	INSIDE	Hs.643588	5308	4	111774274	111774469	196	0	1	1	0.229700159	0.0014541	no
PKIA	INSIDE	Hs.433700	5569	8	79591316	79591543	228	1	0	1	0.371109992	0.000492759	no
PLD5	INSIDE	Hs.672452	200150	1	240753208	240753585	378	3	1	1	0.209794772	0.001290602	no
PLD5	INSIDE	Hs.672452	200150	1	240753586	240753841	256	1	2	2	0.178429791	0.005885081	no
POU6F2	INSIDE	Hs.137106	11281	7	39420336	39420779	444	3	2	4	0.392032616	0.009456006	no
PPARA	INSIDE	Hs.103110	5465	22	44988687	44990302	1616	8	15	9	-0.1273223	0.009802959	no
PPM1E	INSIDE	Hs.245044	22843	17	54188259	54188823	565	5	9	2	0.153860225	0.008945777	no
PPP2R3A	INSIDE	Hs.518155	5523	3	137167754	137168168	415	5	5	4	-0.06776699	0.005042292	no
PPP2R3B	INSIDE	Hs.124942	28227	Y	251358	252717	1360	2	9	13	-0.090380767	0.000577878	no
PPP2R3B	INSIDE	Hs.124942	28227	Y	253399	253763	365	0	1	1	0.365541982	0.002449109	no
PRDM12	INSIDE	Hs.495311	59335	9	132530969	132531484	516	2	8	5	0.11407476	0.001269471	no
PRDM14	INSIDE	Hs.287532	63978	8	71145691	71146065	375	2	1	4	0.441093362	1.96E-06	no
PRDM14	INSIDE	Hs.287532	63978	8	71144829	71145576	748	8	8	7	0.078098337	0.005653974	no
PRDM15	INSIDE	Hs.473893	63977	21	42114182	42117153	2972	5	12	4	-0.091684821	0.001240127	no
PRDM16	INSIDE	Hs.99500	63976	1	3153690	3154493	804	8	7	9	0.088625158	0.008311563	no
PREX1	INSIDE	Hs.153310	57580	20	46876885	46877294	410	3	3	2	0.357698191	9.32E-06	no
PTGER3	INSIDE	Hs.445000	5733	1	71284620	71285088	469	1	1	3	0.394566695	0.008080187	no
PTGFR	INSIDE	Hs.654365	5737	1	78729569	78730427	859	1	4	6	0.222766183	0.000904099	no
PTPRG	INSIDE	Hs.654488	5793	3	61525364	61525974	611	4	1	3	0.127245519	0.004771909	no
PTPRS	INSIDE	Hs.700666	5802	19	5201255	5202099	845	1	4	4	-0.087266156	0.00782224	no
PTPRZ1	INSIDE	Hs.489824	5803	7	121300690	121301293	604	5	2	6	0.29485443	0.001146248	no
PXDN	INSIDE	Hs.332197	7837	2	1725343	1726044	702	3	4	3	0.347134034	0.006033374	no
PXDN	INSIDE	Hs.332197	7837	2	1630982	1632820	1839	8	11	14	-0.080953846	0.008489394	no
RASD2	INSIDE	Hs.474711	23551	22	34277363	34278973	1611	2	5	5	-0.165978677	0.000776567	no
RBM5	INSIDE		NA	3	50102073	50102324	252	2	1	2	-0.074320941	0.009804716	no
RCVRN	INSIDE	Hs.80539	5957	17	9747762	9749450	1689	1	5	4	-0.152119084	0.000142604	no
RESP18	INSIDE	Hs.290551	389075	2	219904565	219905378	814	3	2	6	0.169913704	0.005026029	no
RGAG4	INSIDE	Hs.512180	340526	X	71267800	71267985	186	2	2	1	0.256533854	0.00135549	no
RGMA	INSIDE	Hs.271277	56963	15	91417731	91418316	586	3	4	7	0.160627109	0.00094569	no
RIMBP2	INSIDE	Hs.657441	23504	12	129519230	129519952	723	2	3	3	-0.129902456	0.007243095	no
RNF123	INSIDE	Hs.553723	63891	3	49701708	49702429	722	7	11	1	0.138672006	0.008362452	no
RNF180	INSIDE	Hs.657843	285671	5	63497739	63497936	198	1	1	1	0.289085663	0.000561545	no
ROBO2	INSIDE	Hs.13305	6092	3	77229822	77230734	913	4	5	3	-0.161132299	0.005356	no
ROR2	INSIDE	Hs.98255	4920	9	93525110	93527383	2274	1	13	14	-0.084738228	0.006167922	no

RORB	INSIDE	Hs.494178	6096	9	76302525	76303863	1339	12	15	14	0.140494463	0.004376648	no
RYR3	INSIDE	Hs.369250	6263	15	31391163	31391575	413	2	2	2	0.154100885	0.003756626	no
S100B	INSIDE	Hs.422181	6285	21	46842008	46843276	1269	5	11	3	-0.23551023	7.48E-05	no
SALL3	INSIDE	Hs.669133	27164	18	74841935	74842151	217	3	2	2	0.447558972	8.45E-06	no
SDC2	INSIDE	Hs.1501	6383	8	97575316	97576243	928	10	6	10	0.472178139	8.51E-07	no
SDC2	INSIDE	Hs.1501	6383	8	97576244	97576977	734	4	4	6	0.200746906	0.00407876	no
SEMA6B	INSIDE	Hs.465642	10501	19	4506747	4508584	1838	3	8	3	-0.106521553	0.003064348	no
SFMBT2	INSIDE	Hs.407983	57713	10	7491759	7492044	286	0	1	3	0.350934218	0.002376458	no
SFRP1	INSIDE	Hs.695991	6422	8	41284953	41285369	417	4	5	4	0.20247304	2.12E-05	no
SHOX	INSIDE	Hs.105932	6473	Y	514545	515030	486	5	3	5	0.322116066	0.001586191	no
SLC12A8	INSIDE	Hs.658514	84561	3	126342268	126343857	1590	8	7	5	0.098803723	0.000732131	no
SLC17A6	INSIDE	Hs.242821	57084	11	22319425	22320161	737	3	1	7	0.233448729	0.000406821	no
SLC1A2	INSIDE	Hs.502338	6506	11	35396888	35397191	304	3	4	3	0.1719439	0.000905427	no
SLC26A11	INSIDE	Hs.4866	284129	17	75809282	75809550	269	1	1	3	0.158322482	0.005131821	no
SLC2A14	INSIDE		NA	12	7915941	7916817	877	3	4	2	0.147070387	0.000814836	no
SLC35F1	INSIDE	Hs.654841	222553	6	118347876	118348228	353	3	4	3	0.130484894	0.005561118	no
SLC35F3	INSIDE	Hs.158748	148641	1	232107994	232108549	556	2	3	2	0.280945998	0.005230602	no
SLC45A1	INSIDE	Hs.463036	50651	1	8317195	8319161	1967	2	2	6	-0.07781069	0.004485229	no
SLC6A1	INSIDE	Hs.443874	6529	3	11033506	11034319	814	0	3	6	-0.188050966	0.000267517	no
SLC6A17	INSIDE	Hs.128382	388662	1	110495541	110495819	279	1	0	2	0.188521039	0.003166562	no
SLC6A19	INSIDE	Hs.585128	340024	5	1268423	1270277	1855	4	6	6	-0.100148776	0.008457977	no
SLIT1	INSIDE	Hs.632082	6585	10	98792431	98793635	1205	2	6	6	-0.14566906	0.000128276	no
SLIT2	INSIDE	Hs.699467	9353	4	19865420	19866025	606	8	8	5	0.190799714	0.001435892	no
SLITRK5	INSIDE	Hs.591208	26050	13	87124565	87124727	163	1	1	2	0.463363591	0.000113947	no
SLITRK5	INSIDE	Hs.591208	26050	13	87127774	87128447	674	4	8	1	0.496478646	0.004251726	no
SLITRK5	INSIDE	Hs.591208	26050	13	87127462	87127640	179	1	2	1	0.14818479	0.008260682	no
SMPDL3B	INSIDE	Hs.123659	27293	1	28157342	28158217	876	3	2	5	-0.107567738	0.005146608	no
SORCS2	INSIDE	Hs.479099	57537	4	7717095	7717954	860	1	2	2	-0.220390708	0.000505078	no
SORCS2	INSIDE	Hs.479099	57537	4	7643566	7644283	718	2	3	1	-0.136985496	0.002482421	no
SORCS2	INSIDE	Hs.479099	57537	4	7698377	7699041	665	0	6	3	-0.236104757	0.004841373	no
SORCS2	INSIDE	Hs.479099	57537	4	7562683	7563082	400	1	4	3	-0.17744165	0.009033014	no
SOST	INSIDE	Hs.349204	50964	17	39187905	39188925	1021	13	11	7	0.170907094	2.39E-05	no
SOX11	INSIDE	Hs.432638	6664	2	5753767	5755239	1473	18	21	14	0.070465838	0.009489032	no
SOX7	INSIDE		NA	8	10624274	10624581	308	0	2	1	0.306325839	0.000578795	no
SPATS2	INSIDE	Hs.654826	65244	12	48069217	48069620	404	2	2	2	0.114644055	0.005677113	no
SPERT	INSIDE	Hs.186363	220082	13	45185287	45186657	1371	7	14	12	-0.07640024	0.003765585	no
SPG20	INSIDE	Hs.440414	23111	13	35817799	35818106	308	3	0	3	0.916403347	0.001593919	no
SPN	INSIDE	Hs.632188	6693	16	29582407	29583750	1344	1	7	5	-0.104265539	0.000462774	no
SPOCK2	INSIDE	Hs.523009	9806	10	73518139	73518384	246	4	1	2	0.376588175	0.007295447	no
SRP14	INSIDE	Hs.533732	6727	15	38118096	38118639	544	3	6	5	0.081193194	0.004513148	no
SST	INSIDE	Hs.12409	6750	3	188870109	188870902	794	4	2	4	0.112090418	0.009223419	no
ST8SIA3	INSIDE	Hs.23172	51046	18	53171432	53171682	251	2	2	2	0.394053455	0.000122185	no
ST8SIA3	INSIDE	Hs.23172	51046	18	53172189	53172564	376	3	5	4	0.253386234	0.001968318	no
ST8SIA3	INSIDE	Hs.23172	51046	18	53171683	53172188	506	3	6	3	0.119048078	0.006794201	no
ST8SIA5	INSIDE	Hs.465025	29906	18	42513416	42514656	1241	9	6	8	-0.174495698	0.00015376	no
STRA8	INSIDE	Hs.592279	346673	7	134581344	134582583	1240	4	8	8	-0.066874313	0.004614781	no
STRA8	INSIDE	Hs.592279	346673	7	134568599	134569846	1248	6	5	5	-0.082939977	0.006637521	no
SUSD2	INSIDE	Hs.131819	56241	22	22912736	22914524	1789	3	7	5	-0.086680882	0.001539001	no
SYTL1	INSIDE	Hs.469175	84958	1	27549744	27550147	404	6	2	3	0.163440945	0.004496872	no
T	INSIDE	Hs.389457	6862	6	166499760	166500730	971	6	5	8	0.097263671	0.000505078	no
TAC1	INSIDE	Hs.2563	6863	7	97199238	97199777	540	7	3	5	0.240847082	0.008435296	no
TBC1D16	INSIDE	Hs.369819	125058	17	75531273	75531892	620	2	4	4	0.177125165	0.003219863	no
TBCD	INSIDE	Hs.464391	6904	17	78365855	78366384	530	2	0	4	0.267629228	0.002700696	no
TBR1	INSIDE	Hs.705400	10716	2	161981125	161982369	1245	1	8	7	0.158039187	0.002249551	no
TBX15	INSIDE	Hs.146196	6913	1	119333489	119333818	330	1	4	2	0.638498539	2.10E-07	no
TBX15	INSIDE	Hs.146196	6913	1	119328196	119329121	926	7	6	7	0.216628527	0.00034643	no
TBX15	INSIDE	Hs.146196	6913	1	119331035	119332650	1616	11	11	11	0.068011826	0.008037516	no
TBX5	INSIDE	Hs.381715	6910	12	113322394	113322864	471	1	4	2	0.185739827	0.002564307	no
TCERG1L	INSIDE	Hs.126575	256536	10	132947966	132948676	711	2	4	5	-0.179495163	0.002804784	no
TCF7L1	INSIDE	Hs.516297	83439	2	85215281	85215620	340	0	2	3	0.54029911	4.88E-07	no
TCF7L1	INSIDE	Hs.516297	83439	2	85214985	85215280	296	4	4	2	0.243776844	0.000238319	no
TCF7L1	INSIDE	Hs.516297	83439	2	85215792	85216196	405	3	1	4	0.236068023	0.000815906	no
Tenr	INSIDE		NA	4	123519297	123520399	1103	6	5	6	-0.143919307	0.002536946	no
TEPP	INSIDE	Hs.132976	374739	16	56576737	56577617	881	4	2	9	0.131016554	0.008044182	no
TERT	INSIDE	Hs.492203	7015	5	1305662	1307326	1665	0	9	5	-0.109164148	0.00158878	no
TFAP2E	INSIDE	Hs.567844	339488	1	35814856	35816175	1320	10	7	13	0.127663634	0.004881534	no
TGM6	INSIDE	Hs.452039	343641	20	2331674	2332641	968	2	5	5	-0.145913033	0.006490823	no
TMEFF1	INSIDE	Hs.657066	8577	9	102276338	102276765	428	4	0	2	0.290009755	0.002059292	no
TMEM16E	INSIDE	Hs.154329	203859	11	22171599	22172149	551	4	4	4	0.164317623	0.002504837	no
TMEM26	INSIDE	Hs.623955	219623	10	62881980	62882823	844	7	1	4	0.148463686	0.005086176	no
TMTC2	INSIDE	Hs.577775	160335	12	81605718	81605871	154	1	2	1	-0.142610455	0.009386372	no
TNFRSF1B	INSIDE	Hs.256278	7133	1	12173149	12174652	1504	3	6	4	-0.107593433	0.000647004	no
TRIM9	INSIDE	Hs.654750	114088	14	50629283	50630414	1132	8	5	6	0.086296229	0.002490498	no
TRPM8	INSIDE	Hs.366053	79054	2	234511905	234512678	774	5	4	4	0.264948626	5.14E-05	no
TUBA8	INSIDE	Hs.137400	51807	22	16973854	16974427	574	3	4	4	0.333907797	6.74E-05	no
UHRF1	INSIDE	Hs.108106	29128	19	4901255	4902938	1684	3	8	2	-0.09023612	0.003621045	no
VMO1	INSIDE	Hs.122561	284013	17	4635337	4636476	1140	6	3	10	0.229965672	0.000863755	no
VSTM2	INSIDE		NA	7	54579717	54580156	440	4	7	4	0.089136034	0.008196453	no
VSX1	INSIDE	Hs.274264	30813	20	25006174	25006857	684	1	2	6	0.129013624	0.002231784	no
VWC2	INSIDE	Hs.677488	375567	7	49785083	49785379	297	1	3	3	0.456334855	1.31E-05	no
WNT2	INSIDE	Hs.567356	7472	7	116749613	116750281	669	3	0	4	0.426488905	0.002732715	no
WNT3A	INSIDE	Hs.336930	89780	1	226262476	226263715	1240	11	5	8	0.092380745	0.0092925	no
WTIP	INSIDE	Hs.585010	126374	19	39680309	39681348	1040	3	4	4	-0.146888226	0.001881228	no
WWTR1	INSIDE	Hs.699296	25937	3	150857624	150857818	195	0	2	1	0.447608214	6.85E-06	no
ZAP70	INSIDE	Hs.234569	7535	2	97716835	97719069	2235	4	12	11	-0.078492756	0.005098498	no
ZC3H3	INSIDE	Hs.521915	23144	8	144592651	144592915	265	1	0	2	0.180383973	0.00908114	no
ZCCHC14	INSIDE	Hs.156231	23174	16	85998727	85999227	501	1	1	4	-0.229976449	0.000851189	no
ZDHHC22	INSIDE	Hs.525485	283576	14	76676560	76676728	169	0	2	1	0.200945093	0.006092857	no
ZFHX1B	INSIDE		NA	2	144989591	144990228	638	8	5	3	0.245900421	2.11E-05	no
ZNF232	INSIDE	Hs.279914	7775	17	4959489	4960818	1330	5	17	11	0.133086792	0.000102647	no

ZNF415	INSIDE	Hs.147765	55786	19	58326877	58327667	791	2	3	1	0.18720298	0.002268245	no
ZNF418	INSIDE	Hs.660728	147686	19	63137962	63138543	582	5	6	4	0.11711928	0.004107393	no
ZNF447	INSIDE		NA	19	63287541	63289456	1916	9	18	14	-0.088817641	0.000127509	no
ZNF488	INSIDE	Hs.27788	118738	10	47990128	47991912	1785	0	6	6	-0.092214252	0.008402091	no
ZNF497	INSIDE	Hs.447840	162968	19	63558843	63559439	597	2	3	4	0.13247593	0.006188628	no
ZNF526	INSIDE	Hs.137282	116115	19	47420275	47423217	2943	4	13	15	-0.080436984	0.002221796	no
ZNF528	INSIDE	Hs.662043	84436	19	57593133	57593407	275	0	1	2	0.400713505	0.000818432	no
ZNF606	INSIDE	Hs.654967	80095	19	63205068	63205354	287	1	1	2	0.512913313	0.009489032	no
ZNF677	INSIDE	Hs.20506	342926	19	58449807	58450273	467	6	2	1	0.509981282	5.43E-08	no
ZNF75A	INSIDE	Hs.513292	7627	16	3295371	3296418	1048	3	9	8	0.124409811	0.001332893	no
ZNF790	INSIDE	Hs.282067	388536	19	41979933	41980548	616	3	5	3	0.4680619	2.52E-06	no
ZNF804B	INSIDE	Hs.684410	219578	7	88226918	88227285	368	4	3	4	0.08727918	0.003514193	no
BHLHB5	DOWNSTREAM	Hs.388788	27319	8	65662089	65662668	580	3	3	4	0.151789305	0.009643285	yes
BMI1	DOWNSTREAM	Hs.496613	648	10	22663261	22663819	559	8	4	5	0.194180386	0.000149061	yes
CBLN1	DOWNSTREAM	Hs.458423	869	16	47866619	47866917	299	3	4	2	0.266905787	0.000165128	yes
CBLN1	DOWNSTREAM	Hs.458423	869	16	47869338	47869963	626	3	5	7	0.213920389	0.000165128	yes
CES2	DOWNSTREAM	Hs.282975	8824	16	65538995	65539406	412	2	2	1	-0.206944947	0.003748493	yes
DIO3	DOWNSTREAM	Hs.49322	1735	14	101100495	101100844	350	5	1	3	0.170818091	0.000496453	yes
EMX2	DOWNSTREAM	Hs.202095	2018	10	119301163	119301556	394	4	1	4	0.349199608	0.008754223	yes
FEZF2	DOWNSTREAM	Hs.241523	55079	3	62330088	62330520	433	3	1	2	0.388395655	1.15E-06	yes
FEZF2	DOWNSTREAM	Hs.241523	55079	3	62329653	62330087	435	3	5	4	0.160893538	0.000131028	yes
FOXD1	DOWNSTREAM	Hs.519385	2297	5	72776384	72776681	298	2	3	3	0.244012138	7.60E-09	yes
FOXG1B	DOWNSTREAM		NA	14	28313266	28313651	386	2	4	4	0.336965822	9.32E-06	yes
FOXL2	DOWNSTREAM	Hs.289292	668	3	140140060	140140370	311	2	4	2	0.514004012	1.79E-05	yes
FOXL2	DOWNSTREAM	Hs.289292	668	3	140139833	140140059	227	1	2	1	0.308734327	0.000400223	yes
HOXC12	DOWNSTREAM	Hs.381267	3228	12	52640583	52640782	200	0	1	1	0.388892729	0.001215671	yes
IRX1	DOWNSTREAM	Hs.424156	79192	5	3654226	3654749	524	2	1	4	0.166009284	0.000294968	yes
IRX2	DOWNSTREAM	Hs.282089	153572	5	2793750	2794308	559	4	9	5	0.159205222	0.000183817	yes
ISL1	DOWNSTREAM	Hs.505	3670	5	50730928	50731657	730	3	3	2	0.146374746	0.005559586	yes
LBX1	DOWNSTREAM	Hs.37128	10660	10	102966853	102967043	191	1	2	2	0.121774268	0.003795506	yes
MSX2	DOWNSTREAM	Hs.89404	4488	5	174091685	174091853	169	0	7	1	0.640186675	5.13E-07	yes
MSX2	DOWNSTREAM	Hs.89404	4488	5	174091199	174091684	486	3	6	5	0.368902426	5.28E-07	yes
MSX2	DOWNSTREAM	Hs.89404	4488	5	174091854	174093282	1429	5	5	6	0.123103125	0.000152595	yes
NKX2-2	DOWNSTREAM	Hs.516922	4821	20	21437424	21438020	597	2	6	5	0.330359079	1.42E-05	yes
NKX2-2	DOWNSTREAM	Hs.516922	4821	20	21438021	21438302	282	2	1	3	0.29909143	0.000111827	yes
NKX2-2	DOWNSTREAM	Hs.516922	4821	20	21438303	21439144	842	4	8	9	0.128522854	0.002813217	yes
NKX2-2	DOWNSTREAM	Hs.516922	4821	20	21436009	21436297	289	2	0	2	0.375837895	0.003166562	yes
ONECUT3	DOWNSTREAM	Hs.654347	390874	19	1726847	1727110	264	0	2	1	0.431629597	0.000261451	yes
PDX1	DOWNSTREAM	Hs.32938	3651	13	27400400	27401718	1319	6	7	10	0.214377488	3.55E-06	yes
PDX1	DOWNSTREAM	Hs.32938	3651	13	27399541	27400265	725	4	5	4	0.222697622	5.59E-06	yes
PITX1	DOWNSTREAM	Hs.84136	5307	5	134390584	134391348	765	2	3	5	0.313940688	9.57E-06	yes
POU4F3	DOWNSTREAM	Hs.553499	5459	5	145700122	145701175	1054	2	3	2	0.205745447	0.000689509	yes
POU4F3	DOWNSTREAM	Hs.553499	5459	5	145702840	145703449	610	0	3	4	0.190429315	0.001200381	yes
POU4F3	DOWNSTREAM	Hs.553499	5459	5	145705259	145706183	925	13	11	10	0.072027464	0.003667958	yes
POU4F3	DOWNSTREAM	Hs.553499	5459	5	145702599	145702839	241	2	2	2	0.104525846	0.00687536	yes
PRDM13	DOWNSTREAM	Hs.287386	59336	6	100173115	100174049	935	10	4	8	0.275969538	4.22E-07	yes
PTF1A	DOWNSTREAM	Hs.351503	256297	10	23523475	23524311	837	7	3	5	0.101476913	0.000514422	yes
RAX	DOWNSTREAM	Hs.278957	30062	18	55083177	55083356	180	2	1	1	0.253122933	0.001102898	yes
SHOX2	DOWNSTREAM	Hs.55967	6474	3	159294640	159295401	762	8	6	8	0.100010742	0.004310195	yes
SIX1	DOWNSTREAM	Hs.633506	6495	14	60173681	60174315	635	5	5	6	0.138015535	0.000239206	yes
SIX2	DOWNSTREAM	Hs.101937	10736	2	45085346	45086092	747	7	5	6	0.216381743	0.000219858	yes
SIX2	DOWNSTREAM	Hs.101937	10736	2	45084935	45085345	411	1	5	2	0.360730646	0.000429421	yes
SIX6	DOWNSTREAM	Hs.194756	4990	14	60051205	60051995	791	10	7	7	0.087465121	0.006547861	yes
SOX14	DOWNSTREAM	Hs.248184	8403	3	138971777	138972638	862	2	8	8	0.183519692	0.000468338	yes
SOX21	DOWNSTREAM	Hs.187577	11166	13	94152589	94153082	494	4	3	5	0.339507507	6.62E-06	yes
SOX21	DOWNSTREAM	Hs.187577	11166	13	94152182	94152588	407	1	3	5	0.618687883	0.000718429	yes
SOX21	DOWNSTREAM	Hs.187577	11166	13	94153083	94153500	418	0	2	1	0.161892068	0.004350111	yes
TFAP2A	DOWNSTREAM	Hs.519880	7020	6	10498216	10498841	626	4	4	6	0.394503634	2.23E-08	yes
TFAP2A	DOWNSTREAM	Hs.519880	7020	6	10498842	10499140	299	1	1	2	0.197098258	0.00026587	yes
TFAP2A	DOWNSTREAM	Hs.519880	7020	6	10497670	10498215	546	1	0	3	0.204342494	0.007759064	yes
TLX3	DOWNSTREAM	Hs.249125	30012	5	170674568	170674763	196	2	0	2	0.547176632	0.003561431	yes
UNG2	DOWNSTREAM		NA	5	54554685	54555557	873	2	9	7	0.226356261	1.23E-07	yes
XRN2	DOWNSTREAM	Hs.255932	22803	20	21324297	21326662	2366	35	25	19	0.049643288	0.009972334	yes
ZIC1	DOWNSTREAM	Hs.598590	7545	3	148619400	148619879	480	5	2	5	0.170362129	2.62E-05	yes
ZIC2	DOWNSTREAM	Hs.653700	7546	13	99439581	99441758	2178	10	14	12	0.083596894	0.007735076	yes
ATP12A	DOWNSTREAM	Hs.147111	479	13	24190593	24191615	1023	5	9	5	-0.156496621	0.000837262	no
BAPX1	DOWNSTREAM		NA	4	13146973	13147225	253	0	1	1	0.433935723	0.00010723	no
BHLHB4	DOWNSTREAM	Hs.551230	128408	20	61106979	61108788	1810	31	17	18	0.066766353	0.000940394	no
C1orf89	DOWNSTREAM	Hs.546430	79363	1	16425466	16426701	1236	6	7	6	0.10388146	0.004049806	no
CEI	DOWNSTREAM		NA	5	2808844	2809298	455	3	3	4	0.340939831	3.01E-06	no
CEI	DOWNSTREAM		NA	5	2809818	2810084	267	5	1	2	0.469522526	7.98E-05	no
CLU	DOWNSTREAM	Hs.436657	1191	8	27505316	27506559	1244	4	4	5	-0.15180768	7.84E-06	no
CRIPAK	DOWNSTREAM	Hs.26410	285464	4	1388105	1389118	1014	12	17	11	0.076134878	2.32E-05	no
DBNDD1	DOWNSTREAM	Hs.301394	79007	16	88593446	88594678	1233	1	7	6	-0.097321725	0.002072682	no
DLK1	DOWNSTREAM	Hs.533717	8788	14	100271419	100272205	787	0	1	1	-0.239307377	0.000508564	no
DLX2	DOWNSTREAM	Hs.419	1746	2	172667809	172668927	1119	6	12	10	-0.054715071	0.009926024	no
DLX5	DOWNSTREAM	Hs.99348	1749	7	96483659	96483830	172	2	1	1	0.17591719	0.003957164	no
DUX4C	DOWNSTREAM		NA	4	191172895	191174542	1648	5	20	4	-0.124173477	0.000181813	no
EIF2AK3	DOWNSTREAM	Hs.591589	9451	2	88630361	88631132	772	3	4	4	0.123348232	0.008999135	no
ERAF	DOWNSTREAM	Hs.274309	51327	16	31456152	31456693	542	8	12	3	0.115729933	0.000976765	no
EVX1	DOWNSTREAM	Hs.369879	2128	7	27257560	27258886	1327	12	13	12	0.087737642	0.000106065	no
FEZF1	DOWNSTREAM	Hs.553970	389549	7	121726767	121727566	800	3	3	4	0.460999306	5.62E-06	no
FEZF1	DOWNSTREAM	Hs.553970	389549	7	121727567	121727971	405	3	6	4	0.182001312	0.001415911	no
FLJ40235	DOWNSTREAM	Hs.381087	284369	19	56466060	56466579	520	3	2	4	0.192089638	0.006092857	no
FLJ40448	DOWNSTREAM	Hs.449817	339059	16	87343202	87343406	205	0	1	1	-0.129811852	0.003166562	no
FLJ46347	DOWNSTREAM		NA	2	174899350	174899911	562	9	1	5	0.401544365	0.000453355	no
FOXD2	DOWNSTREAM	Hs.166188	2306	1	47682049	47682313	265	1	2	1	0.394748685	9.21E-07	no
FOXD2	DOWNSTREAM	Hs.166188	2306	1	47682352	47682524	173	1	1	2	0.401863542	1.23E-05	no
FOXD2	DOWNSTREAM	Hs.166188	2306	1	47683492	47683914	423	3	2	2	0.186498326	0.001075804	no
FOXD2	DOWNSTREAM	Hs.166188	2306	1	47682970	47683491	522	5	5	6	0.194446827	0.006738173	no

FOXD3	DOWNSTREAM	Hs.546573	27022	1	63568531	63569020	490	3	6	3	0.243042351	0.005563311	no
FOXD4L3	DOWNSTREAM		NA	9	70111021	70111420	400	3	5	1	0.362861476	0.008675924	no
FOXE1	DOWNSTREAM	Hs.159234	2304	9	99659587	99660309	723	6	7	3	0.191179868	0.002199654	no
GBX2	DOWNSTREAM	Hs.184945	2637	2	236738460	236738671	212	0	2	2	0.17469037	0.00333258	no
GSH2	DOWNSTREAM		NA	4	54670530	54671086	557	2	5	6	0.125984185	0.00782224	no
HAND2	DOWNSTREAM	Hs.388245	9464	4	174676136	174677135	1000	2	3	6	0.105303608	0.001362177	no
HELT	DOWNSTREAM	Hs.531242	391723	4	186178884	186179243	360	1	1	3	0.643347166	5.82E-07	no
HELT	DOWNSTREAM	Hs.531242	391723	4	186179550	186179832	283	0	2	4	0.245877697	0.000398305	no
HELT	DOWNSTREAM	Hs.531242	391723	4	186179280	186179549	270	5	3	3	0.154358974	0.00390837	no
HIST3H2BB	DOWNSTREAM	Hs.376691	128312	1	226718994	226719176	183	2	2	1	0.805155987	7.60E-09	no
HLX1	DOWNSTREAM		NA	1	219133872	219134222	351	0	1	2	0.527671582	4.58E-07	no
HLXB9	DOWNSTREAM		NA	7	156489708	156489948	241	3	3	2	0.473873576	0.000505078	no
hsa-mir-137	DOWNSTREAM		NA	1	98283259	98284247	989	8	7	7	0.088019598	0.001739529	no
IRX6	DOWNSTREAM	Hs.369907	79190	16	53923221	53923472	252	2	1	1	0.373850633	0.000854429	no
JAG2	DOWNSTREAM	Hs.433445	3714	14	104673715	104675461	1747	2	11	4	-0.1178654	0.003690477	no
KIF25	DOWNSTREAM	Hs.150013	3834	6	168191399	168192697	1299	3	5	7	-0.090666577	9.87E-05	no
LHX2	DOWNSTREAM	Hs.696425	9355	9	125842147	125844706	2560	7	16	6	-0.054926066	0.005813768	no
LOC283392	DOWNSTREAM	Hs.363603	283392	12	70951996	70952552	557	7	4	6	0.39371305	4.71E-06	no
LOC283392	DOWNSTREAM	Hs.363603	283392	12	70951434	70951995	562	4	3	1	0.601021176	0.000142604	no
LOC399706	DOWNSTREAM		NA	10	1209062	1210641	1580	1	3	4	-0.108103579	0.004274999	no
MEIS1	DOWNSTREAM	Hs.526754	4211	2	66662510	66662890	381	7	2	4	0.299730134	1.34E-05	no
MEIS1	DOWNSTREAM	Hs.526754	4211	2	66661781	66662509	729	7	3	6	0.093261253	0.003820351	no
MSX1	DOWNSTREAM	Hs.424414	4487	4	4919399	4919715	317	1	3	2	0.141180217	0.004917952	no
MSX1	DOWNSTREAM	Hs.424414	4487	4	4918035	4919227	1193	11	12	3	0.178865807	0.005619856	no
NEUROG2	DOWNSTREAM	Hs.567563	63973	4	113651874	113652416	543	5	4	2	0.132654831	0.002157771	no
NKX2-5	DOWNSTREAM	Hs.54473	1482	5	172588361	172588591	231	3	1	2	0.283662411	0.000399734	no
NKX6-1	DOWNSTREAM	Hs.546270	4825	4	85633298	85633772	475	5	6	4	0.1493351	0.006370553	no
NKX6-2	DOWNSTREAM	Hs.134013	84504	10	134447477	134448316	840	10	17	9	0.152626797	0.000115437	no
OPRL1	DOWNSTREAM	Hs.2859	4987	20	62204062	62204499	438	3	8	4	0.117923554	0.005732351	no
OR2C1	DOWNSTREAM	Hs.258574	4993	16	3354944	3355196	253	1	3	2	0.14579713	0.001790254	no
OTUB1	DOWNSTREAM	Hs.473788	55611	11	63524157	63525049	893	10	15	10	0.088645256	0.000753552	no
PLAG1	DOWNSTREAM	Hs.14968	5324	8	57231997	57232731	735	5	6	6	0.149352148	4.60E-05	no
PLAGL2	DOWNSTREAM	Hs.154104	5326	20	30240794	30242244	1451	11	13	6	0.073712535	0.007363527	no
PROP1	DOWNSTREAM	Hs.158301	5626	5	177344331	177344745	415	3	3	3	0.12592358	0.004556543	no
PSCD2	DOWNSTREAM	Hs.144011	9266	19	53675348	53676359	1012	7	7	7	0.127453792	0.000193318	no
RTN4RL1	DOWNSTREAM	Hs.22917	146760	17	1783930	1784763	834	0	8	4	-0.106369961	0.004333028	no
SAMD1	DOWNSTREAM	Hs.140309	90378	19	14056954	14057471	518	0	4	3	0.169753228	5.74E-05	no
SAMD1	DOWNSTREAM	Hs.140309	90378	19	14057472	14057890	419	8	5	4	0.188418579	0.003352024	no
SIX6	DOWNSTREAM	Hs.194756	4990	14	60051996	60052846	851	11	3	5	0.095472916	0.003077619	no
SLC1A3	DOWNSTREAM	Hs.481918	6507	5	36725745	36726530	786	5	5	7	0.127797423	0.004771909	no
SLC43A1	DOWNSTREAM	Hs.591952	8501	11	57005878	57006497	620	0	4	1	0.198573863	0.000247051	no
SOX1	DOWNSTREAM	Hs.202526	6656	13	111776154	111776729	576	3	3	4	0.209897699	1.39E-05	no
SOX21	DOWNSTREAM	Hs.187577	11166	13	94157629	94158129	501	7	3	3	0.387990477	0.000588215	no
SP8	DOWNSTREAM	Hs.195922	221833	7	20782661	20783126	466	3	6	5	0.219680383	0.00445448	no
SRXN1	DOWNSTREAM	Hs.355284	140809	20	569800	571518	1719	6	10	3	0.104216711	0.001144224	no
STAT3	DOWNSTREAM	Hs.463059	6774	17	37717716	37718736	1021	6	9	9	0.08218854	0.000426592	no
STX4	DOWNSTREAM	Hs.83734	6810	16	30960844	30961306	463	3	4	5	0.297025067	3.84E-06	no
TBR1	DOWNSTREAM	Hs.705400	10716	2	161992314	161992561	248	1	0	2	0.295056785	0.000254192	no
TWIST1	DOWNSTREAM	Hs.66744	7291	7	19113841	19114444	604	2	1	1	0.587177987	9.32E-06	no
TWIST1	DOWNSTREAM	Hs.66744	7291	7	19112296	19112853	558	8	2	5	0.324717882	2.93E-05	no
TWIST1	DOWNSTREAM	Hs.66744	7291	7	19112891	19113840	950	1	3	4	0.379026456	0.000135306	no
TWIST1	DOWNSTREAM	Hs.66744	7291	7	19118337	19119097	761	3	5	5	0.225786846	0.000447951	no
UNCX4.1	DOWNSTREAM		NA	7	1244120	1244530	411	2	1	5	0.153328057	4.08E-05	no
UNCX4.1	DOWNSTREAM		NA	7	1248724	1249431	708	11	12	6	0.072662484	0.001014062	no
UNCX4.1	DOWNSTREAM		NA	7	1245155	1245311	157	0	1	1	0.177060088	0.009432211	no
VIPR2	DOWNSTREAM	Hs.654505	7434	7	158508409	158509631	1223	1	5	1	-0.252606269	0.001834654	no
XKR5	DOWNSTREAM	Hs.558840	389610	8	6650884	6651958	1075	1	6	5	-0.166617331	0.000253635	no
ZFP14	DOWNSTREAM	Hs.35524	57677	19	41514192	41515203	1012	2	7	1	0.341218643	6.57E-06	no
ZNF136	DOWNSTREAM	Hs.479874	7695	19	12166516	12167603	1088	5	13	4	0.275398145	0.000428039	no
ZNF146	DOWNSTREAM	Hs.643436	7705	19	41427805	41428428	624	5	5	6	0.197620832	3.01E-06	no
ZNF266	DOWNSTREAM	Hs.656185	10781	19	9378225	9379278	1054	5	3	8	0.136151478	5.51E-06	no
ZNF329	DOWNSTREAM	Hs.458377	79673	19	63321772	63322043	272	2	1	2	0.39174409	0.002604442	no
ZNF383	DOWNSTREAM	Hs.590975	163087	19	42434520	42434919	400	1	5	5	0.311652201	5.77E-05	no
ZNF610	DOWNSTREAM	Hs.357663	162963	19	57565209	57565420	212	3	2	2	0.619241712	4.89E-05	no
ZNF613	DOWNSTREAM	Hs.183390	79898	19	57144111	57144537	427	2	2	3	0.210119738	0.001045681	no
chr1:038714560-038714604	Unknown		NA	1	38714434	38714723	290	2	5	2	0.147439946	0.005169637	yes
chr10:008118022-008118066	Unknown		NA	10	8117803	8118303	501	3	5	4	0.171701548	4.76E-06	yes
chr10:022582526-022582570	Unknown		NA	10	22581636	22582780	1145	13	20	12	0.176990922	0.000302337	yes
chr10:022804950-022804994	Unknown		NA	10	22804878	22805613	736	14	10	8	0.232633059	0.000668493	yes
chr10:022805801-022805845	Unknown		NA	10	22805671	22805962	292	2	7	2	0.197007555	0.001297954	yes
chr10:022806090-022806137	Unknown		NA	10	22806001	22806155	155	0	2	1	0.721443542	1.05E-07	yes
chr10:023502165-023502224	Unknown		NA	10	23502100	23502605	506	8	5	5	0.545603952	1.97E-06	yes
chr10:102409224-102409278	Unknown		NA	10	102408334	102409381	1048	7	5	3	0.122485099	0.006693916	yes
chr10:102463570-102463614	Unknown		NA	10	102463540	102463710	171	3	1	1	0.406875974	0.001135499	yes
chr10:110215935-110215979	Unknown		NA	10	110215591	110216758	1168	11	5	6	0.113745364	9.76E-05	yes
chr10:118917207-118917251	Unknown		NA	10	118916896	118917333	438	2	3	3	0.265007543	0.000183817	yes
chr10:124883217-124883261	Unknown		NA	10	124883140	124883932	793	2	1	3	0.156371159	0.001718281	yes
chr11:087881596-087881640	Unknown		NA	11	87881285	87882510	1226	10	10	10	0.085117233	0.000732582	yes
chr12:005410245-005410289	Unknown		NA	12	5410100	5410457	358	2	1	3	0.219417364	0.008037516	yes
chr12:052607742-052607786	Unknown		NA	12	52607511	52608058	548	3	2	6	0.190539485	2.88E-05	yes
chr13:049601801-049601845	Unknown		NA	13	49601340	49601892	553	4	0	3	0.441216729	0.000449789	yes
chr13:049602063-049602107	Unknown		NA	13	49601893	49602203	311	2	3	3	0.114813918	0.005885081	yes
chr13:111755698-111755755	Unknown		NA	13	111755549	111755911	363	2	1	2	0.676701421	0.001635405	yes
chr13:111756146-111756190	Unknown		NA	13	111755912	111756705	794	7	7	7	0.135133867	0.000751611	yes
chr13:111757810-111757854	Unknown		NA	13	111757754	111758235	482	5	1	5	0.220215516	0.001974822	yes
chr13:111806906-111806950	Unknown		NA	13	111806203	111807151	949	7	12	8	0.262846275	8.13E-05	yes
chr13:111807261-111807305	Unknown		NA	13	111807152	111807580	429	10	2	3	0.193073217	0.000458718	yes
chr13:111807777-111807821	Unknown		NA	13	111807678	111808188	511	6	5	4	0.282450749	5.63E-06	yes
chr13:111808371-111808415	Unknown		NA	13	111808189	111808472	284	3	1	2	0.404946394	8.51E-07	yes
chr13:111808525-111808584	Unknown		NA	13	111808473	111808637	165	0	2	1	0.370122291	0.000518806	yes

chr13:111809033-111809077	Unknown	NA	13	111808802	111809336	535	1	2	3	0.705616947	5.13E-07	yes
chr14:028324555-028324603	Unknown	NA	14	28324100	28324928	829	7	7	9	0.084404571	0.000251282	yes
chr14:036043472-036043516	Unknown	NA	14	36042927	36043553	627	6	6	7	0.166751183	0.002682438	yes
chr15:087750542-087750587	Unknown	NA	15	87750273	87750769	497	5	4	4	0.396072936	1.23E-05	yes
chr15:087750803-087750847	Unknown	NA	15	87750770	87751011	242	1	1	1	0.138963546	0.00113994	yes
chr15:087751301-087751352	Unknown	NA	15	87751227	87751879	653	5	4	6	0.101641024	0.006533074	yes
chr15:087752147-087752201	Unknown	NA	15	87752091	87752698	608	3	2	1	0.676677931	2.53E-06	yes
chr15:087754075-087754119	Unknown	NA	15	87753854	87754170	317	2	2	3	0.151918547	0.000487275	yes
chr15:094712512-094712556	Unknown	NA	15	94712042	94712656	615	3	3	2	0.305736255	0.000274139	yes
chr15:094760446-094760490	Unknown	NA	15	94760296	94760709	414	3	5	4	0.22939111	0.000245173	yes
chr16:085088191-085088235	Unknown	NA	16	85088064	85088355	292	4	1	3	0.163720788	0.002218378	yes
chr16:085089118-085089163	Unknown	NA	16	85088988	85089224	237	5	1	2	0.398480032	0.000109022	yes
chr17:032239607-032239651	Unknown	NA	17	32239413	32240438	1026	9	13	7	0.119779915	0.000181989	yes
chr17:044182457-044182501	Unknown	NA	17	44181930	44182945	1016	1	4	4	0.231267515	0.000718429	yes
chr17:060204970-060205014	Unknown	NA	17	60204386	60205975	1590	16	9	4	0.064989215	0.008669241	yes
chr18:005186349-005186393	Unknown	NA	18	5186182	5186521	340	0	2	3	0.313260535	0.001497559	yes
chr18:005187248-005187292	Unknown	NA	18	5187082	5187443	362	3	4	2	0.202957462	0.002829097	yes
chr19:035408275-035408319	Unknown	NA	19	35408078	35408413	336	2	3	3	0.366990192	0.004343683	yes
chr19:036535778-036535822	Unknown	NA	19	36535637	36536226	590	6	1	5	0.239414386	0.000377139	yes
chr19:038804459-038804503	Unknown	NA	19	38803723	38805258	1536	20	28	12	0.059827293	0.009643285	yes
chr20:021034956-021035000	Unknown	NA	20	21034718	21035064	347	2	2	3	0.181772632	0.006370553	yes
chr20:033653563-033653607	Unknown	NA	20	33653528	33654428	901	2	4	2	0.473199817	8.10E-06	yes
chr20:047368788-047368832	Unknown	NA	20	47368000	47369145	1146	13	2	10	0.100388829	0.005896681	yes
chr20:054934429-054934473	Unknown	NA	20	54934264	54934700	437	1	4	3	0.419040982	1.80E-05	yes
chr3:044013052-044013096	Unknown	NA	3	44012821	44013463	643	6	5	7	0.380787909	0.004251958	yes
chr3:148559927-148559971	Unknown	NA	3	148559743	148560081	339	2	2	3	0.210200103	0.00236759	yes
chr3:182896592-182896636	Unknown	NA	3	182896494	182896759	266	3	2	2	0.26741142	0.00017796	yes
chr3:187394340-187394384	Unknown	NA	3	187393994	187395285	1292	5	14	10	0.133383589	0.002268245	yes
chr5:043031220-043031279	Unknown	NA	5	43031076	43031385	310	0	2	1	0.919559562	0.000109295	yes
chr5:050301446-050301490	Unknown	NA	5	50301335	50301758	424	4	3	3	0.33504949	4.71E-06	yes
chr5:072562232-072562276	Unknown	NA	5	72561760	72562448	689	6	5	6	0.128991997	1.75E-05	yes
chr5:072565596-072565640	Unknown	NA	5	72565511	72565717	207	1	1	2	0.124451618	0.008766997	yes
chr5:072630151-072630195	Unknown	NA	5	72629833	72630203	371	6	6	4	0.245080593	4.88E-07	yes
chr5:072631482-072631526	Unknown	NA	5	72631398	72631595	198	3	2	1	0.244265545	0.001551343	yes
chr5:072751651-072751695	Unknown	NA	5	72751580	72752092	513	0	2	2	0.257476367	4.88E-07	yes
chr5:072767699-072767743	Unknown	NA	5	72767302	72767874	573	0	2	4	0.223878442	1.15E-05	yes
chr5:077183344-077183392	Unknown	NA	5	77183032	77183426	395	11	3	1	0.79587406	4.27E-05	yes
chr5:077289699-077289743	Unknown	NA	5	77289554	77289832	279	3	5	2	0.07909598	0.003166562	yes
chr5:077304544-077304589	Unknown	NA	5	77304081	77304589	509	7	9	5	0.140057071	0.001539077	yes
chr5:092931949-092931993	Unknown	NA	5	92931934	92932425	492	6	9	5	0.095256181	0.003803366	yes
chr5:092965549-092965593	Unknown	NA	5	92965138	92965656	519	2	2	2	0.299610745	0.000181485	yes
chr5:113419685-113419730	Unknown	NA	5	113419478	113420138	661	5	7	6	0.113372248	0.006455207	yes
chr5:134853140-134853189	Unknown	NA	5	134852961	134853233	273	1	4	2	0.45760334	0.000102586	yes
chr5:134854001-134854045	Unknown	NA	5	134853551	134854124	574	6	5	7	0.322925681	0.000751611	yes
chr5:139506423-139506482	Unknown	NA	5	139506140	139506701	562	2	3	3	0.194894166	2.80E-05	yes
chr6:010490029-010490073	Unknown	NA	6	10489406	10490421	1016	4	8	11	0.154562736	1.23E-05	yes
chr6:010493168-010493214	Unknown	NA	6	10492917	10493224	308	3	5	3	0.239070536	6.24E-05	yes
chr6:010493490-010493540	Unknown	NA	6	10493260	10493630	371	4	4	2	0.52386345	3.08E-07	yes
chr6:026721885-026721929	Unknown	NA	6	26721292	26721962	671	2	2	1	0.338459958	0.003613506	yes
chr6:027755865-027755909	Unknown	NA	6	27755743	27756298	556	2	1	2	0.391063369	9.31E-08	yes
chr6:028711294-028711338	Unknown	NA	6	28711026	28711713	688	2	2	3	0.260497162	0.001773953	yes
chr6:041450391-041450435	Unknown	NA	6	41450185	41450621	437	2	3	4	0.196847571	0.000219621	yes
chr6:045738857-045738901	Unknown	NA	6	45738735	45739896	1162	8	10	12	0.119306316	0.000466137	yes
chr6:106536153-106536197	Unknown	NA	6	106535954	106536656	703	6	6	7	0.173473157	0.007450458	yes
chr6:106540864-106540908	Unknown	NA	6	106540605	106540981	377	3	4	3	0.22887573	0.004026607	yes
chr6:106548758-106548802	Unknown	NA	6	106548673	106548938	266	4	3	3	0.176395331	0.004513148	yes
chr8:011575051-011575095	Unknown	NA	8	11574927	11575110	184	3	2	2	0.326610665	0.001281884	yes
chr8:023615861-023615905	Unknown	NA	8	23615740	23616833	1094	12	4	8	0.120725706	0.001758558	yes
chr8:023618698-023618745	Unknown	NA	8	23617887	23619160	1274	4	8	9	0.142239793	0.00182399	yes
chr8:023619983-023620027	Unknown	NA	8	23619161	23620835	1675	10	7	18	0.127224825	0.001297954	yes
chr8:055545537-055545581	Unknown	NA	8	55545070	55545867	798	11	6	8	0.137446137	0.000165128	yes
chr8:068036682-068036726	Unknown	NA	8	68036623	68036827	205	1	3	1	0.214529735	0.005878032	yes
chr8:068037134-068037178	Unknown	NA	8	68036828	68037390	563	6	6	5	0.336810983	0.000183181	yes
chr8:068037663-068037708	Unknown	NA	8	68037573	68037841	269	2	1	2	0.66881527	1.21E-07	yes
chr8:073080120-073080164	Unknown	NA	8	73079671	73080546	876	5	4	5	0.179168118	0.007685519	yes
chr8:073326627-073326671	Unknown	NA	8	73326246	73326766	521	4	2	2	0.146925901	0.004376648	yes
chr8:100055148-100055192	Unknown	NA	8	100054825	100055283	459	3	6	5	0.10434795	0.004513148	yes
chr8:100055994-100056038	Unknown	NA	8	100055284	100056064	781	10	6	9	0.498323416	6.85E-06	yes
chr8:145909778-145909822	Unknown	NA	8	145909702	145910114	413	5	2	4	0.384593639	2.82E-05	yes
chr8:145910420-145910464	Unknown	NA	8	145910255	145910860	606	4	4	3	0.203204	0.000780339	yes
chr9:003170959-003171003	Unknown	NA	9	3170843	3172073	1231	13	11	8	0.109150271	0.005569664	yes
chr9:029204991-029205036	Unknown	NA	9	29204949	29205302	354	1	3	1	0.342469655	0.000171847	yes
chrX:039916342-039916386	Unknown	NA	X	39916084	39917470	1387	14	19	14	0.078251483	0.001214318	yes
chr1:002866362-002866406	Unknown	NA	1	2865344	2866744	1401	6	1	3	-0.124787544	0.004333895	no
chr1:004092583-004092630	Unknown	NA	1	4092144	4092816	673	0	5	4	-0.104828943	0.006033374	no
chr1:005716071-005716115	Unknown	NA	1	5716047	5716983	937	0	2	2	-0.155220827	0.000159091	no
chr1:008194734-008194782	Unknown	NA	1	8194332	8195142	811	1	1	5	-0.288536295	0.004730551	no
chr1:012523033-012523077	Unknown	NA	1	12522897	12524665	1769	1	7	3	-0.122172537	0.002633426	no
chr1:016720487-016720533	Unknown	NA	1	16719703	16720852	1150	4	5	2	0.28601952	0.000533244	no
chr1:022484852-022484896	Unknown	NA	1	22483887	22485240	1354	4	9	3	-0.136014968	0.003016494	no
chr1:032703703-032703747	Unknown	NA	1	32702883	32703925	1043	8	4	9	0.111767068	0.006705347	no
chr1:033863403-033863447	Unknown	NA	1	33862314	33863831	1518	0	5	5	-0.127957377	0.007257418	no
chr1:038816746-038816790	Unknown	NA	1	38816636	38817492	857	9	7	5	0.233286885	0.000181946	no
chr1:040371044-040371090	Unknown	NA	1	40370958	40371418	461	1	6	4	0.26401515	1.94E-05	no
chr1:043125530-043125574	Unknown	NA	1	43124950	43126336	1387	2	10	6	-0.13836263	0.001067918	no
chr1:046729099-046729143	Unknown	NA	1	46728650	46729389	740	6	10	7	0.120160822	0.000935156	no
chr1:046729562-046729606	Unknown	NA	1	46729390	46731102	1713	0	4	4	0.131225502	0.007200436	no
chr1:050655540-050655584	Unknown	NA	1	50655004	50656086	1083	6	4	11	0.091660204	0.001170048	no
chr1:057663217-057663261	Unknown	NA	1	57663126	57663516	391	1	1	2	0.485799652	0.001949183	no
chr1:061281182-061281226	Unknown	NA	1	61281066	61281368	303	1	4	2	0.092027852	0.004135386	no

chr1:069807034-069807078	Unknown	NA	1	69806841	69807174	334	3	4	4	0.124094696	0.002544747	no
chr1:085854547-085854591	Unknown	NA	1	85854272	85854772	501	7	5	5	0.176325391	0.003064348	no
chr1:119344681-119344725	Unknown	NA	1	119344589	119345055	467	2	7	3	0.342784277	1.34E-05	no
chr1:119350852-119350896	Unknown	NA	1	119350664	119351056	393	3	2	4	0.395251273	0.000181485	no
chr1:119351447-119351491	Unknown	NA	1	119351057	119351710	654	7	7	7	0.347678081	8.10E-06	no
chr1:119352168-119352212	Unknown	NA	1	119351711	119352480	770	4	8	9	0.229916183	4.90E-07	no
chr1:144702242-144702285	Unknown	NA	1	144701972	144702548	577	3	2	2	0.709914698	7.60E-09	no
chr1:145018989-145019033	Unknown	NA	1	145018733	145019359	627	2	4	3	0.24290305	0.000129518	no
chr1:146248916-146248960	Unknown	NA	1	146248609	146249311	703	3	7	7	0.141704828	0.003650871	no
chr1:146256185-146256229	Unknown	NA	1	146256145	146256722	578	5	4	2	0.175836789	0.006743874	no
chr1:146256725-146256784	Unknown	NA	1	146256723	146257026	304	0	3	1	0.189811137	0.001246631	no
chr1:146292898-146292942	Unknown	NA	1	146292503	146293135	633	0	6	4	-0.14167111	0.00899833	no
chr1:147423166-147423210	Unknown	NA	1	147422213	147423307	1095	4	6	4	0.233164121	6.85E-06	no
chr1:156386168-156386212	Unknown	NA	1	156386105	156386378	274	2	3	2	0.526764984	0.000808759	no
chr1:215374320-215374368	Unknown	NA	1	215373982	215374440	459	3	2	1	0.357153708	1.62E-05	no
chr1:215377743-215377787	Unknown	NA	1	215377549	215378629	1081	6	6	8	0.10063532	0.002106721	no
chr1:226724189-226724244	Unknown	NA	1	226724021	226724526	506	0	6	2	0.284862175	0.007204541	no
chr1:227609494-227609543	Unknown	NA	1	227609078	227609614	537	0	2	1	0.343430017	2.32E-05	no
chr1:242147711-242147755	Unknown	NA	1	242147548	242147790	243	2	2	2	0.563515796	1.11E-08	no
chr10:000762248-000762292	Unknown	NA	10	761585	763164	1580	1	4	1	-0.184366613	0.000889648	no
chr10:001242113-001242165	Unknown	NA	10	1240714	1242208	1495	2	4	1	-0.222129027	0.003470617	no
chr10:001242249-001242293	Unknown	NA	10	1242209	1243001	793	2	4	2	-0.295362613	5.84E-05	no
chr10:005922360-005922404	Unknown	NA	10	5922224	5923308	1085	2	6	6	-0.115946416	0.000413409	no
chr10:029051092-029051136	Unknown	NA	10	29050983	29051173	191	2	1	2	0.243425171	0.002371094	no
chr10:043483111-043483155	Unknown	NA	10	43482606	43483261	656	5	6	6	0.086600802	0.001613161	no
chr10:050009786-050009831	Unknown	NA	10	50009223	50010438	1216	0	9	7	-0.093937564	0.006577191	no
chr10:094170417-094170461	Unknown	NA	10	94170387	94170984	598	4	2	6	0.524063122	2.53E-06	no
chr10:119484526-119484570	Unknown	NA	10	119484260	119484625	366	1	3	2	0.397039654	0.000152595	no
chr10:119484822-119484866	Unknown	NA	10	119484626	119485025	400	2	5	3	0.172590166	0.000237702	no
chr10:125722841-125722885	Unknown	NA	10	125722409	125723010	602	2	3	5	0.357401687	4.88E-07	no
chr10:126127506-126127550	Unknown	NA	10	126127257	126127742	486	1	1	6	0.176980663	0.005774309	no
chr10:126128076-126128120	Unknown	NA	10	126127975	126128196	222	1	1	1	0.354573955	6.02E-05	no
chr10:126907354-126907398	Unknown	NA	10	126906140	126907423	1284	9	3	1	-0.112276643	0.000739737	no
chr10:130188852-130188896	Unknown	NA	10	130188777	130189889	1113	0	3	5	-0.117771941	0.002808465	no
chr10:132763609-132763653	Unknown	NA	10	132763270	132764018	749	0	11	2	-0.255690583	0.000167207	no
chr10:133032415-133032459	Unknown	NA	10	133032015	133032927	913	8	2	2	-0.280292996	0.003019381	no
chr10:133699747-133699794	Unknown	NA	10	133698743	133699833	1091	2	3	2	0.15285415	0.000331153	no
chr10:133700656-133700700	Unknown	NA	10	133700611	133701066	456	2	1	3	0.27248378	2.21E-05	no
chr10:133787866-133787910	Unknown	NA	10	133786987	133788471	1485	6	10	7	-0.188220096	0.000680108	no
chr10:134532809-134532853	Unknown	NA	10	134532240	134533001	762	3	5	4	-0.095552438	0.008049269	no
chr10:134590764-134590808	Unknown	NA	10	134590315	134591157	843	0	9	5	-0.10983352	0.005206251	no
chr11:001185371-001185415	Unknown	NA	11	1182891	1185813	2923	0	20	5	-0.084409928	0.004485229	no
chr11:001969819-001969863	Unknown	NA	11	1969247	1969997	751	1	5	2	-0.126990349	0.002417629	no
chr11:022411119-022411178	Unknown	NA	11	22411010	22411206	197	1	1	2	0.289625285	0.001192268	no
chr11:023708572-023708617	Unknown	NA	11	23708375	23709493	1119	4	7	5	-0.096138051	0.00645997	no
chr11:032312040-032312084	Unknown	NA	11	32311463	32312284	822	6	11	6	0.133683712	0.000929576	no
chr11:050183675-050183719	Unknown	NA	11	50183504	50184040	537	1	6	4	-0.093329982	0.008688652	no
chr11:091597388-091597432	Unknown	NA	11	91597158	91599147	1990	18	19	22	0.094606411	0.000295001	no
chr11:091599747-091599798	Unknown	NA	11	91599148	91599946	799	7	9	9	0.248838306	0.000632141	no
chr11:122953374-122953418	Unknown	NA	11	122952948	122953668	721	0	7	5	-0.119583671	0.001161347	no
chr12:037825253-037825297	Unknown	NA	12	37825157	37825597	441	6	6	4	0.60600164	8.10E-06	no
chr12:051645789-051645833	Unknown	NA	12	51645549	51645979	431	1	4	3	0.143017358	0.001343836	no
chr12:064422328-064422372	Unknown	NA	12	64422128	64422796	669	8	15	5	0.208552263	0.007132015	no
chr12:077722289-077722333	Unknown	NA	12	77721231	77723034	1804	0	15	4	-0.298272958	0.009527551	no
chr12:095407623-095407667	Unknown	NA	12	95407267	95407978	712	5	5	8	0.140990218	0.000578795	no
chr12:097663578-097663622	Unknown	NA	12	97663308	97663676	369	3	1	2	0.283556824	2.54E-05	no
chr12:111497932-111497980	Unknown	NA	12	111497369	111498072	704	7	7	5	0.122649494	0.003166562	no
chr12:113366320-113366364	Unknown	NA	12	113365873	113366440	568	7	5	5	0.192856312	0.003578783	no
chr12:118517138-118517182	Unknown	NA	12	118516919	118517940	1022	2	9	8	0.147023325	0.005980027	no
chr12:127318964-127319008	Unknown	NA	12	127318762	127319043	282	4	1	2	0.189294535	2.70E-05	no
chr12:127465823-127465867	Unknown	NA	12	127465181	127466240	1060	0	7	5	-0.180000598	0.004170982	no
chr12:127755839-127755883	Unknown	NA	12	127755412	127756879	1468	2	6	5	-0.103249436	0.000732131	no
chr12:131369707-131369751	Unknown	NA	12	131369376	131370094	719	1	7	7	-0.091636915	0.003432863	no
chr12:131994721-131994780	Unknown	NA	12	131994180	131994833	654	2	3	1	0.183786743	0.00209318	no
chr12:131994991-131995035	Unknown	NA	12	131994847	131995690	844	7	11	8	0.184474978	0.000583046	no
chr12:131995719-131995763	Unknown	NA	12	131995691	131996039	349	0	3	3	0.189717798	0.008630449	no
chr13:022938884-022938928	Unknown	NA	13	22938136	22939073	938	4	4	7	0.103473171	0.002076474	no
chr13:028005004-028005048	Unknown	NA	13	28004261	28005598	1338	17	18	7	0.081063725	0.001009066	no
chr13:035769816-035769864	Unknown	NA	13	35769662	35769914	253	2	1	2	0.441429125	5.76E-06	no
chr13:035770063-035770107	Unknown	NA	13	35769915	35770383	469	1	4	2	0.399633955	0.000899648	no
chr13:094418881-094418925	Unknown	NA	13	94418779	94419168	390	1	3	2	0.167329062	0.003628608	no
chr13:099447362-099447406	Unknown	NA	13	99447256	99447872	617	9	7	6	0.101823394	0.000324028	no
chr13:110998752-110998796	Unknown	NA	13	110998604	110999102	499	1	4	4	-0.130402363	0.000886065	no
chr14:020509724-020509771	Unknown	NA	14	20509131	20510014	884	5	5	6	-0.136075283	0.001539314	no
chr14:069386999-069387045	Unknown	NA	14	69386268	69387197	930	4	6	6	-0.174072139	1.62E-05	no
chr14:074517462-074517506	Unknown	NA	14	74517406	74518720	1315	0	7	2	-0.090614138	0.004942311	no
chr14:096569838-096569882	Unknown	NA	14	96569165	96570033	869	9	8	9	0.093140517	0.009240927	no
chr14:096754940-096754984	Unknown	NA	14	96754776	96755206	431	2	8	4	0.116896703	0.003650871	no
chr14:096755499-096755558	Unknown	NA	14	96755239	96755824	586	4	3	5	0.240028471	7.56E-05	no
chr14:098654701-098654752	Unknown	NA	14	98654534	98654833	300	8	0	2	0.266787426	0.008690253	no
chr14:100245679-100245723	Unknown	NA	14	100245591	100245821	231	1	4	1	0.436971618	0.000428039	no
chr14:102725391-102725435	Unknown	NA	14	102725000	102725531	532	10	7	6	0.056378609	0.009242243	no
chr14:103691632-103691676	Unknown	NA	14	103690232	103692264	2033	4	16	6	-0.082129338	0.000543573	no
chr14:103872035-103872091	Unknown	NA	14	103871954	103873394	1441	0	6	1	-0.16968673	0.006937792	no
chr15:018824083-018824127	Unknown	NA	15	18822111	18824425	2315	3	11	2	-0.310877791	0.000531264	no
chr15:023393237-023393281	Unknown	NA	15	23392701	23393959	1259	3	7	6	-0.152212	0.006003464	no
chr15:025725091-025725140	Unknown	NA	15	25725040	25725548	509	0	5	5	-0.142319242	0.009033014	no
chr15:025821609-025821653	Unknown	NA	15	25821255	25822467	1213	4	4	8	-0.089317181	0.006218678	no
chr15:039700900-039700944	Unknown	NA	15	39700847	39701156	310	7	3	3	0.227163003	0.007115217	no
chr15:060303704-060303748	Unknown	NA	15	60303669	60304438	770	3	8	7	-0.134890951	0.001224462	no

chr16:001020750-001020794	Unknown	NA	16	1020658	1021275	618	0	8	1	-0.138491229	0.004574159	no
chr16:003182715-003182759	Unknown	NA	16	3182222	3182917	696	4	8	4	0.097751394	0.00556192	no
chr16:012903849-012903893	Unknown	NA	16	12903752	12904844	1093	3	5	10	0.211626822	0.000268461	no
chr16:012905157-012905201	Unknown	NA	16	12905065	12905530	466	3	2	2	0.277966356	0.000122213	no
chr16:034153041-034153085	Unknown	NA	16	34152139	34153243	1105	4	6	3	-0.137920553	0.007124931	no
chr16:049432503-049432557	Unknown	NA	16	49432414	49432734	321	4	1	2	0.283361793	0.004012557	no
chr16:049705215-049705262	Unknown	NA	16	49705095	49705564	470	3	4	4	0.077277244	0.005675719	no
chr16:058346514-058346558	Unknown	NA	16	58345930	58346660	731	2	3	3	-0.144742187	0.000935156	no
chr16:074826690-074826734	Unknown	NA	16	74826431	74827084	654	2	5	2	-0.1293756	5.74E-05	no
chr16:084539055-084539114	Unknown	NA	16	84538369	84539977	1609	3	6	6	-0.154265198	1.13E-05	no
chr16:087497795-087497845	Unknown	NA	16	87496892	87499131	2240	4	13	5	-0.084002372	0.001780803	no
chr17:000352920-000352964	Unknown	NA	17	352818	353331	514	0	9	1	-0.285102297	0.005966388	no
chr17:021877633-021877677	Unknown	NA	17	21876589	21877879	1291	3	8	1	-0.126451027	0.002302843	no
chr17:072763492-072763538	Unknown	NA	17	72763176	72763954	779	0	4	2	-0.291368383	4.88E-05	no
chr18:006919937-006919981	Unknown	NA	18	6919203	6920230	1028	0	5	9	-0.070335929	0.00107151	no
chr18:010404558-010404602	Unknown	NA	18	10404178	10404670	493	1	4	2	-0.183203248	0.000746793	no
chr18:010579338-010579382	Unknown	NA	18	10578897	10579454	558	2	6	5	-0.116960837	0.005351228	no
chr18:041609256-041609300	Unknown	NA	18	41609159	41609740	582	2	5	5	0.19564204	0.000661972	no
chr18:051598094-051598138	Unknown	NA	18	51597948	51598239	292	1	1	3	0.39204558	0.000305929	no
chr18:070928647-070928691	Unknown	NA	18	70926401	70928747	2347	5	4	2	-0.086607179	0.008314834	no
chr18:070928889-070928933	Unknown	NA	18	70928748	70929342	595	2	3	3	-0.105911351	0.000456588	no
chr18:072455015-072455059	Unknown	NA	18	72454321	72455743	1423	6	3	4	-0.123338355	0.003888632	no
chr18:073819389-073819436	Unknown	NA	18	73817774	73820042	2269	2	6	2	-0.144340769	0.000336915	no
chr18:074365275-074365321	Unknown	NA	18	74364159	74366258	2100	0	3	3	-0.209945064	0.001202144	no
chr18:074587783-074587827	Unknown	NA	18	74587612	74588965	1354	5	4	3	-0.171828916	0.008688652	no
chr18:074642492-074642536	Unknown	NA	18	74641867	74642669	803	2	6	7	-0.156403837	1.39E-05	no
chr18:074654002-074654046	Unknown	NA	18	74653629	74654552	924	0	3	5	-0.179355268	0.000796722	no
chr18:075414911-075414955	Unknown	NA	18	75414129	75415004	876	4	5	3	-0.20621175	0.001218079	no
chr18:075643869-075643921	Unknown	NA	18	75643342	75644875	1534	4	10	6	-0.144211148	0.002396764	no
chr18:075661163-075661207	Unknown	NA	18	75660654	75661471	818	3	3	4	-0.292700879	1.36E-06	no
chr18:075668344-075668390	Unknown	NA	18	75667996	75668581	586	3	2	3	-0.217540359	0.005777556	no
chr18:075670709-075670753	Unknown	NA	18	75668582	75670868	2287	35	35	5	-0.120202835	0.000780339	no
chr18:075687255-075687299	Unknown	NA	18	75687025	75687559	535	3	3	5	-0.217713037	0.000489647	no
chr19:000373606-000373650	Unknown	NA	19	371697	373729	2033	2	14	6	-0.089409535	0.000370428	no
chr19:000377936-000377980	Unknown	NA	19	376712	378311	1600	1	23	3	-0.145345605	0.002969754	no
chr19:002464707-002464758	Unknown	NA	19	2463746	2464907	1162	2	11	6	-0.144293566	0.000294968	no
chr19:003782752-003782796	Unknown	NA	19	3782179	3782978	800	2	3	7	-0.094424638	0.005324076	no
chr19:009707647-009707696	Unknown	NA	19	9707264	9708117	854	5	3	5	-0.154360188	1.58E-05	no
chr19:013208004-013208048	Unknown	NA	19	13207691	13208677	987	0	5	6	-0.139486572	6.87E-05	no
chr19:036539860-036539916	Unknown	NA	19	36539702	36540193	492	5	1	6	0.113991839	0.006370553	no
chr19:038805695-038805739	Unknown	NA	19	38805513	38805997	485	5	5	4	0.351508338	1.57E-07	no
chr19:038806186-038806230	Unknown	NA	19	38806116	38806448	333	2	1	2	0.30657666	4.86E-05	no
chr19:042156197-042156241	Unknown	NA	19	42156091	42156863	773	2	3	4	0.263944791	1.02E-06	no
chr19:042999762-042999806	Unknown	NA	19	42999463	42999873	411	1	1	3	0.376514125	8.64E-06	no
chr19:043000067-043000111	Unknown	NA	19	42999874	43000712	839	5	3	5	0.24539793	0.000658536	no
chr19:043037662-043037706	Unknown	NA	19	43036940	43038039	1100	12	9	6	0.132907376	0.001605783	no
chr19:057648425-057648469	Unknown	NA	19	57648341	57648751	411	3	3	4	0.407692248	5.34E-06	no
chr19:057648996-057649040	Unknown	NA	19	57648900	57649465	566	1	1	2	0.196894996	4.95E-05	no
chr19:057688406-057688450	Unknown	NA	19	57688355	57688671	317	1	4	1	0.174127772	0.003064348	no
chr19:058233087-058233138	Unknown	NA	19	58232887	58233221	335	4	1	3	0.150009327	0.009489032	no
chr19:058253135-058253179	Unknown	NA	19	58252494	58253267	774	4	4	1	0.287510247	0.000153319	no
chr19:058503712-058503756	Unknown	NA	19	58503304	58503981	678	3	4	6	0.164447447	5.76E-05	no
chr19:061571571-061571615	Unknown	NA	19	61571169	61571830	662	5	4	7	0.297547834	8.03E-06	no
chr19:062302747-062302794	Unknown	NA	19	62302373	62302866	494	3	2	5	0.195175513	0.004003749	no
chr19:062310141-062310185	Unknown	NA	19	62310068	62310413	346	4	2	3	0.315575806	9.77E-05	no
chr19:062912331-062912377	Unknown	NA	19	62911452	62912639	1188	3	7	6	0.16251566	0.001248997	no
chr2:000458225-000458269	Unknown	NA	2	457936	459066	1131	11	6	10	0.09691251	0.001327435	no
chr2:000611450-000611494	Unknown	NA	2	610489	612111	1623	12	13	17	-0.074086625	0.0092925	no
chr2:007824903-007824947	Unknown	NA	2	7824369	7825219	851	3	5	6	-0.244995199	0.008080187	no
chr2:008085422-008085466	Unknown	NA	2	8084579	8086451	1873	4	16	1	-0.231020693	0.004012444	no
chr2:011018944-011018988	Unknown	NA	2	11018076	11019463	1388	4	15	7	-0.094885211	0.000955293	no
chr2:040531948-040531992	Unknown	NA	2	40531761	40532622	862	5	2	5	0.151531579	0.004637933	no
chr2:044882571-044882615	Unknown	NA	2	44882451	44883028	578	2	6	5	0.092637991	0.004456383	no
chr2:044883187-044883239	Unknown	NA	2	44883113	44883323	211	4	1	1	0.307246433	0.000250949	no
chr2:045008748-045008792	Unknown	NA	2	45008625	45008903	279	2	3	2	0.543154868	1.45E-08	no
chr2:045251181-045251225	Unknown	NA	2	45251126	45251548	423	3	3	4	0.312737391	4.50E-06	no
chr2:045251675-045251726	Unknown	NA	2	45251600	45251833	234	0	1	1	0.439354625	1.65E-05	no
chr2:052652132-052652176	Unknown	NA	2	52651807	52652970	1164	3	6	12	-0.116563402	0.00067866	no
chr2:086890200-086890244	Unknown	NA	2	86890016	86890814	799	5	4	6	0.121587021	0.005830762	no
chr2:101456825-101456869	Unknown	NA	2	101456700	101458034	1335	13	15	11	0.047307351	0.004881534	no
chr2:104827401-104827445	Unknown	NA	2	104827228	104827680	453	6	5	4	0.16528215	0.008532748	no
chr2:104828267-104828313	Unknown	NA	2	104827681	104828386	706	5	7	6	0.155470011	0.001045681	no
chr2:107527205-107527249	Unknown	NA	2	107527039	107528381	1343	5	7	1	-0.220517397	0.001028072	no
chr2:130061596-130061640	Unknown	NA	2	130060822	130061986	1165	5	7	6	-0.112458221	0.00042654	no
chr2:154438156-154438207	Unknown	NA	2	154437758	154438371	614	2	1	5	0.189057897	0.000647586	no
chr2:210382261-210382305	Unknown	NA	2	210381895	210383146	1252	1	2	5	-0.118802493	0.001161347	no
chr2:226155455-226155499	Unknown	NA	2	226154696	226156184	1489	4	8	13	-0.082138054	0.000284943	no
chr2:236753045-236753089	Unknown	NA	2	236752881	236753453	573	7	5	4	0.076250446	0.005854334	no
chr2:240493519-240493563	Unknown	NA	2	240493118	240493747	630	3	3	4	-0.256583131	0.009404396	no
chr2:242576047-242576091	Unknown	NA	2	242575964	242576758	795	0	9	2	-0.148567689	0.001558127	no
chr2:242578563-242578607	Unknown	NA	2	242578017	242579443	1427	3	7	6	-0.107522087	0.007379696	no
chr20:001731832-001731876	Unknown	NA	20	1731740	1732450	711	5	10	6	0.104722348	0.00908114	no
chr20:001732612-001732658	Unknown	NA	20	1732451	1732806	356	4	3	2	0.343798769	0.000282555	no
chr20:020949092-020949136	Unknown	NA	20	20949061	20949293	233	2	1	2	0.222150452	0.006218678	no
chr20:021030517-021030561	Unknown	NA	20	21030341	21030745	405	4	2	4	0.095962869	0.002150401	no
chr20:033652088-033652132	Unknown	NA	20	33651826	33652251	426	2	5	2	0.243285753	2.85E-05	no
chr20:033652793-033652837	Unknown	NA	20	33652252	33653067	816	8	4	8	0.226624706	0.000183817	no
chr20:044215640-044215687	Unknown	NA	20	44215255	44215766	512	3	3	5	0.12480389	0.009125023	no
chr20:050155217-050155261	Unknown	NA	20	50154381	50155479	1099	11	15	7	0.117455937	0.000455933	no
chr20:051710382-051710436	Unknown	NA	20	51709799	51710476	678	4	1	6	0.103306183	0.003613506	no

chr20:061523306-061523350	Unknown	NA	20	61523128	61524499	1372	2	8	7	-0.083558822	0.002491725	no
chr20:061932055-061932099	Unknown	NA	20	61931986	61932269	284	6	3	1	0.376510914	0.006369468	no
chr21:014378462-014378506	Unknown	NA	21	14377901	14380060	2160	7	15	7	-0.09369725	0.003819289	no
chr21:044446690-044446736	Unknown	NA	21	44446468	44447846	1379	0	8	4	-0.093406715	0.003166562	no
chr21:044750976-044751020	Unknown	NA	21	44750926	44751600	675	3	5	5	-0.18271105	0.001906164	no
chr22:016229574-016229618	Unknown	NA	22	16229234	16229865	632	1	4	6	0.134633641	0.003166562	no
chr22:026340446-026340490	Unknown	NA	22	26340420	26340651	232	3	2	2	0.301714934	0.000153482	no
chr22:037123872-037123916	Unknown	NA	22	37123645	37125082	1438	23	21	2	0.102819695	0.007532109	no
chr22:044640770-044640814	Unknown	NA	22	44639582	44640844	1263	2	5	2	-0.0838221	0.009207819	no
chr22:046984090-046984134	Unknown	NA	22	46983997	46984708	712	1	3	2	-0.132214395	9.44E-05	no
chr22:047109101-047109145	Unknown	NA	22	47107189	47109279	2091	4	12	6	-0.069419487	0.008111962	no
chr3:044014514-044014559	Unknown	NA	3	44014113	44014596	484	0	2	5	0.406338108	5.07E-06	no
chr3:044014625-044014669	Unknown	NA	3	44014597	44014915	319	1	1	3	0.371873805	4.02E-05	no
chr3:044015118-044015162	Unknown	NA	3	44014975	44015305	331	1	3	3	0.129043929	0.008044182	no
chr3:044258500-044258544	Unknown	NA	3	44257925	44258972	1048	8	9	10	-0.116615928	0.003239258	no
chr3:047025406-047025450	Unknown	NA	3	47025384	47026135	752	6	7	7	0.091780845	0.003862159	no
chr3:087220842-087220886	Unknown	NA	3	87220476	87221455	980	6	5	6	0.107602275	0.001248997	no
chr3:106555127-106555171	Unknown	NA	3	106555107	106555818	712	3	5	7	0.201592446	0.000149308	no
chr3:119198844-119198888	Unknown	NA	3	119198207	119199124	918	7	8	4	0.109982649	0.004682425	no
chr3:131465602-131465646	Unknown	NA	3	131465300	131465970	671	6	7	5	-0.211412813	0.001577482	no
chr3:140130416-140130460	Unknown	NA	3	140129920	140131756	1837	3	7	6	-0.104291994	0.000818675	no
chr3:165204755-165204799	Unknown	NA	3	165204626	165205079	454	2	4	3	-0.242015877	0.009033014	no
chr3:182029622-182029666	Unknown	NA	3	182028431	182030790	2360	1	25	7	-0.097949049	0.002770604	no
chr3:182895767-182895823	Unknown	NA	3	182895330	182896322	993	8	6	6	0.101558996	0.001816711	no
chr3:182896362-182896406	Unknown	NA	3	182896323	182896493	171	3	0	2	0.34687745	0.000508564	no
chr3:182927142-182927186	Unknown	NA	3	182927099	182927435	337	2	3	3	0.261278575	0.001222358	no
chr3:195069993-195070045	Unknown	NA	3	195069881	195071221	1341	7	12	7	-0.088032685	0.005356	no
chr4:001039833-001039877	Unknown	NA	4	1039506	1039982	477	1	4	4	-0.111980217	0.003608335	no
chr4:001391116-001391160	Unknown	NA	4	1390734	1391230	497	4	5	5	0.315539305	1.03E-07	no
chr4:001391658-001391702	Unknown	NA	4	1391231	1391756	526	9	5	5	0.1715717	2.19E-06	no
chr4:003614074-003614118	Unknown	NA	4	3612780	3614307	1528	19	10	4	-0.178887955	0.002921211	no
chr4:003641332-003641376	Unknown	NA	4	3641030	3641500	471	1	5	4	-0.194974709	4.12E-05	no
chr4:003712180-003712236	Unknown	NA	4	3712165	3712634	470	0	1	1	-0.255654763	0.003950536	no
chr4:006165350-006165395	Unknown	NA	4	6165047	6165796	750	2	1	3	-0.221162004	0.007668816	no
chr4:008696800-008696844	Unknown	NA	4	8696519	8697356	838	0	4	5	-0.15275439	0.003748493	no
chr4:013133374-013133418	Unknown	NA	4	13132907	13133619	713	0	5	7	0.192430875	1.02E-05	no
chr4:013140029-013140073	Unknown	NA	4	13139170	13140233	1064	3	3	4	0.119738641	0.006772545	no
chr4:024699535-024699579	Unknown	NA	4	24699254	24699778	525	3	3	5	0.186832993	0.003228177	no
chr4:025115995-025116039	Unknown	NA	4	25115633	25117639	2007	18	14	14	-0.069021934	0.003783016	no
chr4:041570334-041570378	Unknown	NA	4	41570045	41570448	404	1	5	3	0.2478107	7.21E-05	no
chr4:041570496-041570540	Unknown	NA	4	41570449	41570627	179	1	3	2	0.301363978	0.001120827	no
chr4:041575987-041576031	Unknown	NA	4	41575895	41576185	291	4	3	2	0.130630906	0.004143249	no
chr4:041577424-041577468	Unknown	NA	4	41577308	41577816	509	3	2	3	0.290812845	4.08E-05	no
chr4:048858303-048858347	Unknown	NA	4	48857870	48858523	654	4	9	2	-0.352403612	0.002608093	no
chr4:061750281-061750325	Unknown	NA	4	61749641	61750408	768	2	8	7	0.1891032	0.003950536	no
chr4:100793601-100793652	Unknown	NA	4	100792828	100795111	2284	2	12	5	-0.165785669	0.002249551	no
chr4:111728519-111728565	Unknown	NA	4	111728210	111728891	682	1	3	5	-0.0998418	0.002041937	no
chr4:174664804-174664848	Unknown	NA	4	174664697	174664868	172	1	2	2	0.272717611	0.001680001	no
chr4:187658939-187658998	Unknown	NA	4	187657938	187659535	1598	2	2	4	-0.149757628	0.001819565	no
chr4:189317815-189317859	Unknown	NA	4	189317299	189318901	1603	2	11	1	-0.203827186	0.009643285	no
chr4:189789790-189789845	Unknown	NA	4	189789003	189789977	975	5	9	1	-0.255388115	0.001137131	no
chr5:001223919-001223974	Unknown	NA	5	1223234	1224025	792	0	1	1	-0.236556522	0.001105037	no
chr5:001798718-001798766	Unknown	NA	5	1797835	1799316	1482	1	5	7	-0.098948506	0.005565579	no
chr5:002002097-002002141	Unknown	NA	5	2001760	2002826	1067	0	9	5	-0.206983932	0.001368137	no
chr5:002060676-002060720	Unknown	NA	5	2059788	2061323	1536	3	14	10	-0.056099631	0.008027732	no
chr5:042987843-042987887	Unknown	NA	5	42987761	42988744	984	3	8	4	0.131919807	0.00130735	no
chr5:054215046-054215100	Unknown	NA	5	54215028	54215299	272	0	2	2	0.405503705	7.98E-05	no
chr5:064022306-064022350	Unknown	NA	5	64021739	64022799	1061	10	11	10	0.080252558	0.007614312	no
chr5:072714097-072714141	Unknown	NA	5	72713991	72714254	264	3	3	2	0.096392139	0.007959345	no
chr5:115724923-115724967	Unknown	NA	5	115724675	115725577	903	7	6	7	0.093109654	0.005653974	no
chr5:169592469-169592513	Unknown	NA	5	169592320	169593502	1183	3	6	6	-0.095495112	0.000197029	no
chr5:172108373-172108420	Unknown	NA	5	172107609	172108967	1359	6	11	6	-0.079927913	0.005151278	no
chr5:172605184-172605232	Unknown	NA	5	172604912	172605267	356	2	0	3	0.374393034	0.005356	no
chr5:172605299-172605343	Unknown	NA	5	172605268	172606116	849	5	8	4	0.148475073	0.005980027	no
chr5:178733995-178734039	Unknown	NA	5	178732642	178735074	2433	0	7	4	-0.279990469	0.000701558	no
chr5:178889943-178889991	Unknown	NA	5	178889604	178890623	1020	4	12	6	0.101033851	0.009124803	no
chr5:180033608-180033652	Unknown	NA	5	180033353	180033890	538	12	10	5	0.098433073	0.002813217	no
chr6:019799993-019800040	Unknown	NA	6	19799770	19800288	519	8	3	5	0.211892516	0.000245173	no
chr6:030539836-030539880	Unknown	NA	6	30539528	30540655	1128	7	10	5	0.100691336	0.006450637	no
chr6:031256542-031256586	Unknown	NA	6	31256333	31256624	292	0	5	3	0.225560871	0.002909979	no
chr6:040675288-040675336	Unknown	NA	6	40675233	40675462	230	2	2	1	0.812966603	0.000668493	no
chr6:040675546-040675593	Unknown	NA	6	40675463	40675920	458	2	1	1	0.396897712	3.44E-05	no
chr6:066861276-066861320	Unknown	NA	6	66861109	66861566	458	2	6	1	-0.175223074	0.003700594	no
chr6:072186165-072186219	Unknown	NA	6	72185999	72186410	412	4	1	2	0.301859959	9.32E-06	no
chr6:074076164-074076208	Unknown	NA	6	74075854	74076347	494	2	3	3	0.315137823	0.001718281	no
chr6:074076721-074076771	Unknown	NA	6	74076627	74077794	1168	0	3	2	0.136608992	0.005237495	no
chr6:074081375-074081424	Unknown	NA	6	74081036	74081713	678	8	6	7	0.160038214	0.002041937	no
chr6:099379024-099379079	Unknown	NA	6	99378435	99379833	1399	9	2	9	0.08314255	0.004272524	no
chr6:112790679-112790734	Unknown	NA	6	112789962	112790818	857	0	8	1	-0.310364515	0.000556029	no
chr6:117192985-117193029	Unknown	NA	6	117192810	117193716	907	8	11	8	0.140151554	0.000720308	no
chr6:134680493-134680539	Unknown	NA	6	134680385	134680768	384	3	3	4	0.33898927	0.000530942	no
chr6:139056576-139056621	Unknown	NA	6	139056520	139057447	928	1	1	1	-0.186390296	0.004435187	no
chr6:161108003-161108047	Unknown	NA	6	161107801	161108776	976	5	8	4	0.126315307	0.00315852	no
chr6:168133923-168133967	Unknown	NA	6	168132951	168134010	1060	2	18	9	-0.119176557	0.007656599	no
chr6:169309274-169309318	Unknown	NA	6	169307592	169309540	1949	5	9	5	-0.068940213	0.004827202	no
chr6:170317195-170317239	Unknown	NA	6	170317141	170318133	993	1	12	5	-0.115919102	0.002588836	no
chr6:170629964-170630008	Unknown	NA	6	170628551	170630452	1902	0	2	2	-0.136309537	0.002348991	no
chr7:001303563-001303607	Unknown	NA	7	1303448	1303707	260	0	2	2	0.097114324	0.009056296	no
chr7:012409593-012409651	Unknown	NA	7	12409339	12409700	362	0	2	1	0.455759441	0.001907818	no
chr7:016533141-016533185	Unknown	NA	7	16533069	16533337	269	0	1	2	0.166591635	0.003957164	no

chr7:025868932-025868976	Unknown	NA	7	25868874	25869052	179	0	2	1	-0.098161143	0.0092925	no
chr7:027226632-027226676	Unknown	NA	7	27226464	27227025	562	7	7	5	0.14453568	0.009595486	no
chr7:032304799-032304843	Unknown	NA	7	32304503	32306076	1574	11	15	9	0.084541255	0.00787062	no
chr7:035192993-035193037	Unknown	NA	7	35192903	35193483	581	2	4	1	0.974816098	4.88E-07	no
chr7:035461003-035461047	Unknown	NA	7	35460724	35461069	346	6	3	3	0.282580611	0.000839121	no
chr7:035461229-035461278	Unknown	NA	7	35461070	35461409	340	3	1	2	0.387548689	0.000775294	no
chr7:039615979-039616023	Unknown	NA	7	39615496	39616120	625	3	6	4	0.202430768	0.001867261	no
chr7:056265069-056265113	Unknown	NA	7	56264859	56265146	288	4	4	3	0.201812858	0.00875078	no
chr7:063290331-063290375	Unknown	NA	7	63289401	63291259	1859	0	5	2	-0.156103505	0.001130933	no
chr7:084652845-084652889	Unknown	NA	7	84652815	84653057	243	2	1	2	0.350249105	3.09E-05	no
chr7:089586937-089586981	Unknown	NA	7	89586127	89587187	1061	8	7	2	0.164472918	0.007253615	no
chr7:127698293-127698337	Unknown	NA	7	127698129	127698532	404	3	5	5	0.148743275	0.002329209	no
chr7:128338061-128338105	Unknown	NA	7	128337616	128338206	591	6	7	6	0.114760176	0.00034643	no
chr7:149020697-149020741	Unknown	NA	7	149020337	149021049	713	5	4	4	0.155865501	1.38E-05	no
chr7:154857394-154857438	Unknown	NA	7	154856845	154857593	749	2	1	4	0.14309357	0.004475093	no
chr7:154857850-154857894	Unknown	NA	7	154857594	154858512	919	11	9	9	0.082067744	0.002492567	no
chr7:154858707-154858754	Unknown	NA	7	154858513	154859075	563	3	2	4	0.200678819	0.003352399	no
chr7:154859762-154859806	Unknown	NA	7	154859386	154860219	834	9	14	9	0.069944201	0.00487057	no
chr7:154860269-154860313	Unknown	NA	7	154860220	154860699	480	12	3	5	0.155187212	0.008082271	no
chr7:154866854-154866905	Unknown	NA	7	154866478	154866980	503	2	1	1	0.387579267	0.001767134	no
chr7:156506700-156506744	Unknown	NA	7	156506649	156506846	198	2	1	2	0.096965543	0.00605735	no
chr7:157148490-157148534	Unknown	NA	7	157147672	157148743	1072	4	4	2	0.116498552	0.005356	no
chr7:157169557-157169612	Unknown	NA	7	157169250	157169626	377	2	4	1	1.332095737	1.70E-09	no
chr7:157172169-157172218	Unknown	NA	7	157172112	157172342	231	0	1	2	0.129691079	0.007950172	no
chr7:157173784-157173837	Unknown	NA	7	157173767	157173978	212	0	1	2	0.100062444	0.009969772	no
chr7:158491345-158491389	Unknown	NA	7	158491108	158492631	1524	21	20	3	-0.149319562	0.004870031	no
chr8:000949806-000949850	Unknown	NA	8	949711	950339	629	2	2	2	-0.201857413	0.001200381	no
chr8:001093105-001093149	Unknown	NA	8	1092508	1093477	970	6	9	10	-0.130486993	0.000691221	no
chr8:001126093-001126151	Unknown	NA	8	1124562	1126270	1709	1	29	2	-0.198479018	0.005771948	no
chr8:001233802-001233846	Unknown	NA	8	1233051	1234426	1376	4	1	2	-0.185425442	0.0050978	no
chr8:002117743-002117789	Unknown	NA	8	2117201	2118860	1660	0	14	3	-0.259689832	0.003802288	no
chr8:011508465-011508511	Unknown	NA	8	11508365	11509394	1030	2	10	4	-0.118461929	0.000729753	no
chr8:030004743-030004787	Unknown	NA	8	30004119	30004833	715	5	8	4	-0.148442215	0.006142807	no
chr8:038627502-038627546	Unknown	NA	8	38627086	38627552	467	3	2	3	0.264791742	0.000350219	no
chr8:049945458-049945502	Unknown	NA	8	49945042	49945923	882	11	8	6	0.146213335	0.003239258	no
chr8:058217647-058217691	Unknown	NA	8	58217215	58218329	1115	10	5	10	0.104502969	0.000488423	no
chr8:071109486-071109530	Unknown	NA	8	71109371	71109747	377	2	6	4	0.178503286	0.000367837	no
chr8:135559940-135559984	Unknown	NA	8	135559444	135560716	1273	0	3	5	-0.104216877	0.005272376	no
chr8:142699273-142699319	Unknown	NA	8	142698282	142699995	1714	0	6	1	-0.137165068	0.007417688	no
chr8:142840103-142840147	Unknown	NA	8	142839474	142840280	807	3	4	5	-0.172837424	0.002418163	no
chr8:143200513-143200559	Unknown	NA	8	143199871	143200618	748	0	2	1	-0.274507958	0.001386891	no
chr9:013268559-013268603	Unknown	NA	9	13267881	13268712	832	3	3	5	0.161861241	0.005565579	no
chr9:014336226-014336274	Unknown	NA	9	14335759	14336676	918	6	4	6	0.257277322	0.002910593	no
chr9:014968006-014968057	Unknown	NA	9	14967371	14968589	1219	6	5	3	-0.142602989	0.004917952	no
chr9:018767281-018767325	Unknown	NA	9	18767101	18768565	1465	1	7	8	-0.092177559	0.00162121	no
chr9:023841552-023841596	Unknown	NA	9	23841077	23841657	581	4	3	5	0.246332181	0.001126561	no
chr9:029204824-029204873	Unknown	NA	9	29204586	29204948	363	2	1	2	0.755670493	4.88E-07	no
chr9:036729816-036729861	Unknown	NA	9	36729736	36729892	157	1	3	1	0.554767453	2.31E-05	no
chr9:066197878-066197922	Unknown	NA	9	66196814	66198471	1658	5	19	6	-0.086101209	0.00506045	no
chr9:073251288-073251332	Unknown	NA	9	73251098	73251442	345	1	2	2	0.421884953	0.004182722	no
chr9:087327574-087327619	Unknown	NA	9	87327296	87327840	545	10	3	5	0.19446008	0.000379763	no
chr9:088816560-088816604	Unknown	NA	9	88816292	88818000	1709	1	6	1	-0.252452295	6.85E-06	no
chr9:089664165-089664209	Unknown	NA	9	89663629	89664864	1236	1	7	3	-0.258519206	0.000680108	no
chr9:089811596-089811640	Unknown	NA	9	89810898	89812034	1137	4	8	9	-0.088672455	0.006380228	no
chr9:098489003-098489047	Unknown	NA	9	98488823	98489262	440	5	3	2	0.463868361	5.53E-06	no
chr9:132402949-132402993	Unknown	NA	9	132402591	132403247	657	0	1	4	0.192874946	0.001029612	no
chrX:039753018-039753062	Unknown	NA	X	39752969	39753612	644	6	9	7	0.061060389	0.003904726	no
chrX:071009843-071009887	Unknown	NA	X	71008448	71010150	1703	2	9	6	-0.126848068	0.000501033	no
chrX:144520702-144520746	Unknown	NA	X	144520351	144521171	821	1	7	6	-0.125037366	0.001151251	no
chrY:000671620-000671677	Unknown	NA	Y	671546	671735	190	0	1	1	0.371345794	0.001067918	no
chrY:012617382-012617425	Unknown	NA	Y	12616162	12618175	2014	3	9	7	-0.140886167	0.00461865	no
