# Supplementary material for: BRAF mutation-specific promoter methylation of FOX genes in colorectal cancer
Source: Clin Epigenetics. 2013 Jan 16;5(1):2. doi: 10.1186/1868-7083-5-2 (PMC3599401; doi:10.1186/1868-7083-5-2)
Supplement: Additional file 2 — Regions with CIMP-specific methylation changes (UCSC assembly: March 2006, NCBI36/hg18). [file 1868-7083-5-2-S2.pdf]

GeneName	Description	UniGene	EntrezGene	Chrom	Start	End	Length	BstUI sites	HpaII sites	Probes	logFC	adj.p.val	H3K27me3 bound
BBS5	PROMOTER	Hs.233398	129880	2	170043599	170044144	546	0	1	1	0.237674112	0.048389028	yes
COCH	PROMOTER	Hs.21016	1690	14	30412172	30414210	2039	18	17	14	0.034805078	0.248330417	yes
COL12A1	PROMOTER	Hs.101302	1303	6	75971995	75972515	521	5	4	5	0.208788654	0.053056319	yes
CYP26A1	PROMOTER	Hs.150595	1592	10	94817946	94819100	1155	10	6	12	0.187750657	0.000732582	yes
DKFZP779L1853	PROMOTER	Hs.640467	643162	12	46877813	46878660	848	3	2	5	0.363244672	0.015343842	yes
DKK1	PROMOTER	Hs.40499	22943	10	53743801	53745035	1235	5	11	11	0.011717037	0.845945133	yes
EBF1	PROMOTER	Hs.657753	1879	5	158465569	158466334	766	2	1	3	0.097253682	0.203663611	yes
FAM62C	PROMOTER	Hs.477711	83850	3	139635427	139635930	504	2	4	1	0.016882312	0.954332417	yes
FLT3	PROMOTER	Hs.507590	2322	13	27572738	27573180	443	4	4	4	0.20874013	0.003163171	yes
FOXC1	PROMOTER	Hs.348883	2296	6	1553532	1553683	152	0	2	1	0.139032017	0.008080187	yes
FOXC1	PROMOTER	Hs.348883	2296	6	1551209	1551628	420	1	2	4	0.317743408	0.015510742	yes
FOXC1	PROMOTER	Hs.348883	2296	6	1553720	1554106	387	1	3	3	0.183559333	0.188642052	yes
FOXC1	PROMOTER	Hs.348883	2296	6	1552611	1553143	533	1	0	7	0.115244221	0.136008302	yes
FOXG1B	PROMOTER		NA	14	28305356	28305691	336	4	5	2	0.117733701	0.022736911	yes
FZD2	PROMOTER	Hs.142912	2535	17	39990104	39992360	2257	19	18	13	0.006179817	0.898940981	yes
GATA4	PROMOTER	Hs.243987	2626	8	11597912	11598094	183	1	1	2	0.068954372	0.351269896	yes
GJB2	PROMOTER	Hs.524894	2706	13	19665677	19666414	738	3	4	3	0.054291346	0.358461372	yes
GREM1	PROMOTER	Hs.40098	26585	15	30796920	30797469	550	5	9	4	0.153382896	0.031412098	yes
GSC	PROMOTER	Hs.440438	145258	14	94306162	94306526	365	2	1	3	0.144584231	0.019651253	yes
HCCS	PROMOTER	Hs.211571	3052	X	11039063	11039917	855	2	9	8	-0.025267225	0.7267916	yes
HHEX	PROMOTER	Hs.118651	3087	10	94438652	94439164	513	2	5	5	0.058705801	0.418253084	yes
HMX2	PROMOTER	Hs.444756	3167	10	124892259	124893350	1092	4	8	5	0.147130649	0.001250274	yes
HOXC11	PROMOTER	Hs.127562	3227	12	52645223	52646112	890	1	4	2	0.312470157	0.003571467	yes
HOXC8	PROMOTER	Hs.664500	3224	12	52685578	52685945	368	7	0	3	0.043230667	0.931791627	yes
hsa-mir-424	PROMOTER		NA	X	133512257	133512983	727	11	5	7	0.073807668	0.148608784	yes
ISLR2	PROMOTER	Hs.254775	57611	15	72209856	72210394	539	5	1	3	0.418557962	0.001950518	yes
KCNV1	PROMOTER	Hs.13285	27012	8	111056863	111057513	651	0	6	5	0.008713906	0.93208388	yes
LBXCOR1	PROMOTER	Hs.451224	390598	15	65900334	65900788	455	3	5	3	0.401740048	0.003754112	yes
LOC285016	PROMOTER		NA	2	279216	279586	371	4	4	2	0.033833965	0.674162614	yes
NFATC1	PROMOTER	Hs.534074	4772	18	75258657	75259295	639	1	6	6	0.166226298	0.154281613	yes
NFATC1	PROMOTER	Hs.534074	4772	18	75259296	75259759	464	1	0	4	0.135315784	0.353511498	yes
NFATC1	PROMOTER	Hs.534074	4772	18	75258301	75258568	268	1	1	2	0.045501688	0.574321967	yes
NPR3	PROMOTER	Hs.237028	4883	5	32745873	32746812	940	8	5	9	0.090069513	0.155774863	yes
NRXN3	PROMOTER	Hs.368307	9369	14	78814935	78815789	855	9	6	9	0.106324623	0.131371582	yes
ONECUT1	PROMOTER	Hs.658573	3175	15	50870652	50871543	892	2	8	4	0.071879081	0.504273877	yes
OTP	PROMOTER	Hs.202247	23440	5	76971703	76972791	1089	7	8	11	0.02384284	0.473198686	yes
PAX6	PROMOTER	Hs.591993	5080	11	31795501	31795809	309	3	1	3	0.235002812	0.051796355	yes
PAX6	PROMOTER	Hs.591993	5080	11	31793315	31793727	413	0	1	4	0.098575251	0.167781024	yes
PAX9	PROMOTER	Hs.132576	5083	14	36196266	36196495	230	2	1	1	0.326347909	0.008994031	yes
PLEC1	PROMOTER	Hs.434248	5339	8	145121092	145121609	518	4	3	4	0.013085713	0.783397247	yes
PMP22	PROMOTER	Hs.372031	5376	17	15106084	15106620	537	3	0	2	-0.064864998	0.621858722	yes
POU3F2	PROMOTER	Hs.182505	5454	6	99386844	99388638	1795	20	9	18	0.088357025	0.03013578	yes
POU4F1	PROMOTER	Hs.654522	5457	13	78081430	78081799	370	1	5	3	0.072566762	0.167781024	yes
PRDM13	PROMOTER	Hs.287386	59336	6	100158334	100158661	328	2	4	3	0.254165539	0.023371468	yes
PRICKLE1	PROMOTER	Hs.524348	144165	12	41270336	41270614	279	2	3	2	0.334491829	0.026390199	yes
RAE1	PROMOTER	Hs.371698	8480	20	55359067	55359754	688	4	2	2	-0.068531466	0.364316354	yes
RGMA	PROMOTER	Hs.271277	56963	15	91433940	91434218	279	4	0	2	0.028987948	0.787106911	yes
ROBO3	PROMOTER	Hs.435621	64221	11	124239823	124240410	588	3	1	2	0.119425615	0.119654196	yes
SH3GL2	PROMOTER	Hs.75149	6456	9	17569037	17569857	821	15	12	8	0.046151206	0.159186792	yes
SIX2	PROMOTER	Hs.101937	10736	2	45090095	45090516	422	3	7	2	0.064703289	0.470882468	yes
SLC26A10	PROMOTER	Hs.159481	65012	12	56299262	56299549	288	1	2	2	0.064981216	0.741583755	yes
SLC26A10	PROMOTER	Hs.159481	65012	12	56299642	56300252	611	3	7	3	-0.000335228	0.998626302	yes
SLC6A4	PROMOTER	Hs.591192	6532	17	25587172	25587490	319	1	1	3	0.048051466	0.655689667	yes
TBX2	PROMOTER	Hs.705451	6909	17	56828611	56828930	320	2	6	2	0.104121758	0.385708892	yes
TFAP2A	PROMOTER	Hs.519880	7020	6	10529692	10530144	453	1	0	1	0.159905964	0.344090956	yes
TITF1	PROMOTER		NA	14	36063789	36064191	403	2	0	4	0.055516322	0.671790509	yes
TITF1	PROMOTER		NA	14	36064192	36064939	748	2	3	1	0.074351117	0.550748377	yes
TITF1	PROMOTER		NA	14	36059857	36060200	344	4	3	3	0.412115294	0.003163171	yes
TITF1	PROMOTER		NA	14	36060201	36060432	232	2	3	2	0.103005551	0.064562708	yes
VASH1	PROMOTER	Hs.525479	22846	14	76297048	76297535	488	4	4	2	0.147987461	0.026637548	yes
ZNF287	PROMOTER	Hs.99724	57336	17	16412540	16413583	1044	8	11	10	0.032616647	0.231208446	yes
ABCA5	PROMOTER	Hs.421474	23461	17	64835314	64835687	374	3	0	2	0.010450952	0.9440286	no
ACA21	PROMOTER		NA	17	34263104	34263549	446	4	2	4	-0.038630809	0.644423526	no
AGPAT5	PROMOTER	Hs.624002	55326	8	6552535	6552897	363	1	0	2	-0.049295954	0.737951575	no
AGRIN	PROMOTER		NA	1	944375	944994	620	9	13	3	0.02174617	0.657696051	no
AGXT2L1	PROMOTER	Hs.106576	64850	4	109903684	109904117	434	2	5	2	0.23830612	0.0651431	no
AIM1L	PROMOTER	Hs.128738	55057	1	26559420	26559926	507	3	3	5	0.034973134	0.665189695	no
ANK1	PROMOTER	Hs.654438	286	8	41775011	41775393	383	1	0	2	-0.038637227	0.543396243	no
ANKDD1A	PROMOTER	Hs.207157	348094	15	62984565	62985137	573	4	2	3	0.15281645	0.249217994	no
ANPEP	PROMOTER	Hs.1239	290	15	88158538	88159114	577	7	3	5	0.030830372	0.511601881	no
ARFGAP1	PROMOTER	Hs.25584	55738	20	61367163	61368918	1756	6	10	3	-0.067335187	0.469897191	no
ARL4A	PROMOTER	Hs.245540	10124	7	12692448	12692655	208	1	1	1	-0.008709963	0.932182968	no
ATOH1	PROMOTER	Hs.532680	474	4	94968697	94968899	203	5	1	1	0.030221368	0.694908204	no
ATP11B	PROMOTER	Hs.478429	23200	3	183993544	183993706	163	1	1	1	0.004430178	0.969839049	no
ATP1B1	PROMOTER	Hs.291196	481	1	167340954	167343148	2195	19	26	22	0.001151437	0.976693349	no
ATRNL1	PROMOTER	Hs.501127	26033	10	116841743	116842468	726	5	0	4	0.104520841	0.178504349	no
AXIN1	PROMOTER	Hs.592082	8312	16	351056	351793	738	2	6	5	-0.072511909	0.356919827	no
BARX1	PROMOTER	Hs.164960	56033	9	95761255	95761562	308	6	3	3	0.116182283	0.009223622	no
BBC3	PROMOTER	Hs.467020	27113	19	52434188	52434530	343	0	2	1	0.423817912	0.001592171	no
BCOR	PROMOTER	Hs.659681	54880	X	39848176	39848483	308	2	0	3	0.180120901	0.342411023	no
BMP3	PROMOTER	Hs.387411	651	4	82170614	82172422	1809	18	13	11	0.033932145	0.33899182	no
BMPER	PROMOTER	Hs.660998	168667	7	33910103	33910655	553	2	3	5	0.188539622	0.087600744	no
BMPR2	PROMOTER	Hs.471119	659	2	202948533	202948923	391	0	4	4	-0.012339062	0.782129903	no
C14orf79	PROMOTER	Hs.27183	122616	14	104515963	104516681	719	3	2	4	0.024065099	0.862215667	no
C15orf17	PROMOTER	Hs.367690	57184	15	72986459	72986670	212	2	0	1	-0.076472308	0.398124825	no
C1orf92	PROMOTER	Hs.491734	149499	1	155156461	155157536	1076	9	7	8	0.020783554	0.736200645	no
C2orf52	PROMOTER	Hs.375211	151477	2	232087184	232087703	520	12	1	6	0.045183813	0.634714489	no
C9orf23	PROMOTER	Hs.15961	138716	9	34600455	34602720	2266	5	13	11	0.009378429	0.796045027	no
CALM2	PROMOTER	Hs.643483	805	2	47256229	47257375	1147	9	10	11	0.018210165	0.700838912	no
CAMK2N2	PROMOTER	Hs.585003	94032	3	185462726	185463338	613	10	1	6	0.256655356	0.011536334	no
CAPZA2	PROMOTER	Hs.695918	830	7	116289500	116290793	1294	16	11	6	0.016558974	0.80256383	no
CCND1	PROMOTER	Hs.523852	595	11	69160211	69160523	313	0	2	3	0.078122588	0.50301543	no
CCNDBP1	PROMOTER	Hs.36794	23582	15	41264054	41265340	1287	1	7	3	0.011461844	0.862879478	no

CDC42BPA	PROMOTER	Hs.35433	8476	1	225573269	225573846	578	2	2	2	0.046691447	0.65277031	no
CENPM	PROMOTER	Hs.208912	79019	22	40673163	40673415	253	1	0	1	0.157949672	0.349732761	no
CHCHD5	PROMOTER	Hs.375707	84269	2	113057990	113058420	431	2	0	5	0.21249145	0.053079423	no
CIRBP	PROMOTER	Hs.634522	1153	19	1218452	1218828	377	2	0	3	0.005074961	0.980203882	no
COL27A1	PROMOTER	Hs.494892	85301	9	115955867	115956123	257	3	1	1	0.060156046	0.365409229	no
CREBBP	PROMOTER	Hs.459759	1387	16	3871516	3871865	350	4	2	2	-0.306401231	0.00074159	no
CROCC	PROMOTER	Hs.309403	9696	1	17112185	17112842	658	3	2	1	-0.094503489	0.58889582	no
CUGBP2	PROMOTER	Hs.309288	10659	10	11099812	11100806	995	23	27	7	0.028464868	0.440707725	no
CUL7	PROMOTER	Hs.520136	9820	6	43128985	43129948	964	10	4	8	-0.055580206	0.110259176	no
DBNL	PROMOTER	Hs.436500	28988	7	44049988	44051153	1166	5	7	7	-0.005040969	0.940460405	no
DCAMKL2	PROMOTER		NA	4	151218561	151218749	189	3	0	2	-0.06115451	0.65798251	no
DDX19A	PROMOTER	Hs.656037	55308	16	68938134	68938649	516	7	4	4	-0.033718965	0.366567896	no
DLX1	PROMOTER	Hs.407015	1745	2	172655954	172656381	428	4	5	4	0.116851339	0.095486203	no
DSG2	PROMOTER	Hs.412597	1829	18	27331241	27332663	1423	14	13	12	-0.031796119	0.1355968	no
EID2B	PROMOTER	Hs.135181	126272	19	44715012	44715513	502	7	4	5	-0.050366174	0.39413825	no
EPC2	PROMOTER	Hs.23270	26122	2	149117873	149119564	1692	17	18	17	-0.007511443	0.849342173	no
ESR2	PROMOTER	Hs.660607	2100	14	63830871	63831060	190	1	0	2	0.010016977	0.945656454	no
EXOC3L2	PROMOTER	Hs.337557	90332	19	50433535	50434274	740	1	8	6	-0.061541901	0.208496192	no
FAM119A	PROMOTER	Hs.668241	151194	2	208198737	208198934	198	2	0	2	-0.087661772	0.559482844	no
FAM77D	PROMOTER		NA	8	63323138	63323712	575	5	10	5	0.177445227	0.034100432	no
FAM77D	PROMOTER		NA	8	63322963	63323137	175	0	2	1	0.144535875	0.027543221	no
FILIP1L	PROMOTER	Hs.104672	11259	3	101077525	101078364	840	7	12	4	0.0404368	0.267251537	no
FLJ32658	PROMOTER	Hs.662085	147872	19	54582639	54583220	582	2	3	1	0.112647702	0.475626507	no
FLJ45537	PROMOTER	Hs.657740	401535	9	88953160	88953856	697	5	7	5	0.056698347	0.375038666	no
FLJ45684	PROMOTER		NA	19	598367	598548	182	0	3	1	0.019530848	0.871423856	no
FNBP1	PROMOTER	Hs.189409	23048	9	131845650	131846291	642	4	3	3	0.451611029	0.017086002	no
FOXB2	PROMOTER	Hs.553843	442425	9	78820845	78821525	681	8	5	7	0.162721712	0.028652306	no
FOXB2	PROMOTER	Hs.553843	442425	9	78818581	78818826	246	3	1	2	0.18794095	0.176431539	no
FOXD3	PROMOTER	Hs.546573	27022	1	63560388	63560797	410	3	4	3	0.144424789	0.191087449	no
FOXD3	PROMOTER	Hs.546573	27022	1	63559266	63559697	432	2	2	2	0.762957783	0.001146248	no
FOXD3	PROMOTER	Hs.546573	27022	1	63560088	63560334	247	0	3	2	0.186682076	0.04461936	no
GBX2	PROMOTER	Hs.184945	2637	2	236741768	236742410	643	4	12	6	0.013490961	0.818792459	no
GBX2	PROMOTER	Hs.184945	2637	2	236744782	236745370	589	3	1	5	0.12347681	0.014316294	no
GBX2	PROMOTER	Hs.184945	2637	2	236742623	236742879	257	2	1	2	0.124798112	0.340211221	no
GNG4	PROMOTER	Hs.159711	2786	1	233880863	233881157	295	2	0	2	0.126948895	0.191807962	no
GPR160	PROMOTER	Hs.231320	26996	3	171238872	171239043	172	1	1	1	0.0049339	0.968470415	no
GPR45	PROMOTER	Hs.590903	11250	2	105219531	105220332	802	2	3	8	0.124484872	0.004442128	no
GSTM3	PROMOTER	Hs.2006	2947	1	110084728	110085243	516	2	2	2	0.372268783	0.016631366	no
HAND2	PROMOTER	Hs.388245	9464	4	174696352	174696909	558	5	2	5	0.109338943	0.227996615	no
HELT	PROMOTER	Hs.531242	391723	4	186174639	186175527	889	2	0	2	0.154843449	0.12396104	no
HEY2	PROMOTER	Hs.144287	23493	6	126111401	126111888	488	4	1	4	0.377224458	0.067285354	no
HEY2	PROMOTER	Hs.144287	23493	6	126110841	126111400	560	4	5	5	0.099239912	0.040354895	no
HHIP	PROMOTER	Hs.507991	64399	4	145785564	145785749	186	2	0	1	0.107057505	0.496734419	no
HOXA2	PROMOTER	Hs.445239	3199	7	27109358	27110393	1036	1	4	5	0.087788918	0.078848284	no
HPCAL4	PROMOTER	Hs.705422	51440	1	39930706	39931278	573	4	1	5	0.018860086	0.8709859	no
HS6ST1	PROMOTER	Hs.512841	9394	2	128795910	128797629	1720	14	20	6	-0.01145466	0.753979774	no
hsa-mir-183	PROMOTER		NA	7	129209327	129209732	406	1	4	4	0.488451453	0.010225365	no
hsa-mir-183	PROMOTER		NA	7	129209733	129210658	926	2	6	7	0.122607094	0.043568604	no
hsa-mir-200b	PROMOTER		NA	1	1089217	1089433	217	1	2	2	-0.093165696	0.143339939	no
hsa-mir-9-2	PROMOTER		NA	5	88007690	88008051	362	1	2	2	0.033360126	0.717781628	no
ID2	PROMOTER	Hs.180919	3398	2	8734051	8734473	423	3	6	4	-0.019440095	0.689805427	no
ID2	PROMOTER	Hs.180919	3398	2	8733652	8734050	399	4	1	5	-0.009175396	0.940460405	no
IGF2BP1	PROMOTER	Hs.144936	10642	17	44429039	44429240	202	1	4	2	0.372740451	0.009223622	no
IL13	PROMOTER	Hs.845	3596	5	132019293	132019580	288	0	1	2	0.082019759	0.102320017	no
INSM1	PROMOTER	Hs.89584	3642	20	20295756	20296719	964	16	13	9	0.018177784	0.691857765	no
ISL2	PROMOTER	Hs.444677	64843	15	74414614	74414918	305	3	1	3	0.185623804	0.116639726	no
ITCH	PROMOTER	Hs.632272	83737	20	32414606	32415546	941	8	16	10	0.013099265	0.758559977	no
KCTD8	PROMOTER	Hs.479644	386617	4	44145796	44146066	271	2	1	2	0.092022081	0.34041452	no
LBH	PROMOTER	Hs.567598	81606	2	30307336	30308069	734	7	9	8	0.109076363	0.010617524	no
LCMT1	PROMOTER	Hs.337730	51451	16	25030292	25030984	693	6	4	7	-0.008814404	0.819553992	no
LEF1	PROMOTER	Hs.555947	51176	4	109311627	109312151	525	0	2	2	0.205171657	0.027192974	no
LEF1	PROMOTER	Hs.555947	51176	4	109312152	109312356	205	0	1	2	0.179034008	0.067097181	no
LEF1	PROMOTER	Hs.555947	51176	4	109312357	109312717	361	1	1	3	0.079672247	0.307718244	no
LHX4	PROMOTER	Hs.658487	89884	1	178464542	178464872	331	2	1	2	0.086290714	0.402891566	no
LHX9	PROMOTER	Hs.442578	56956	1	196147214	196147626	413	2	7	4	0.259915214	0.023546759	no
LHX9	PROMOTER	Hs.442578	56956	1	196147888	196148437	550	2	8	6	0.100617936	0.308578401	no
LOC152573	PROMOTER	Hs.370904	152573	4	42093552	42094199	648	5	5	4	0.086840109	0.115991495	no
LOC286187	PROMOTER	Hs.632064	286187	8	68103182	68103842	661	4	7	5	0.589739453	0.00237766	no
LOC388931	PROMOTER		NA	2	24085970	24087368	1399	10	14	10	0.030968212	0.343984217	no
LOC400657	PROMOTER	Hs.61508	400657	18	70416799	70417026	228	2	0	2	-0.061555823	0.848664988	no
LOC441046	PROMOTER	Hs.135705	441046	4	144699971	144700791	821	9	2	4	0.051438129	0.39818327	no
LRBA	PROMOTER	Hs.480938	987	4	152156641	152157197	557	4	0	5	-0.030021002	0.858845521	no
LRRC32	PROMOTER	Hs.151641	2615	11	76059838	76060186	349	2	1	2	0.181025089	0.284566793	no
LRRTM1	PROMOTER	Hs.591580	347730	2	80384769	80385136	368	2	4	4	0.155567629	0.056535826	no
LY6G5C	PROMOTER	Hs.25738	80741	6	31758903	31759104	202	2	0	2	0.243776602	0.260102687	no
LY6G5C	PROMOTER	Hs.25738	80741	6	31759105	31759300	196	0	1	1	0.086999296	0.446786082	no
LYPD1	PROMOTER	Hs.694844	116372	2	133144875	133145523	649	1	8	6	0.291362199	0.005601251	no
MAF	PROMOTER	Hs.134859	4094	16	78192143	78192328	186	1	1	1	0.000569447	0.99879184	no
MANBAL	PROMOTER	Hs.6126	63905	20	35351286	35352043	758	4	9	4	-0.012609706	0.851880099	no
MCOLN2	PROMOTER	Hs.591446	255231	1	85236379	85236881	503	3	3	5	0.26801329	0.078264312	no
MEF2C	PROMOTER	Hs.699175	4208	5	88215503	88216206	704	7	2	7	0.11226951	0.176687838	no
MEF2D	PROMOTER	Hs.314327	4209	1	154736868	154737516	649	3	11	5	-0.018315905	0.816531324	no
MEGF10	PROMOTER	Hs.438709	84466	5	126653893	126654805	913	4	11	9	0.10636304	0.098952167	no
MEIS1	PROMOTER	Hs.526754	4211	2	66515620	66515851	232	2	1	2	0.444461076	0.018840662	no
MLF1	PROMOTER	Hs.85195	4291	3	159771048	159771729	682	3	3	3	0.091834058	0.378958749	no
MNS1	PROMOTER	Hs.444483	55329	15	54544414	54544982	569	5	0	4	-0.01011357	0.936605047	no
MOCS1	PROMOTER	Hs.357128	4337	6	40009662	40010488	827	4	11	6	-0.020945224	0.438607616	no
MRPS31	PROMOTER	Hs.154655	10240	13	40243025	40243622	598	2	7	5	0.024514721	0.648886001	no
MTHFSD	PROMOTER	Hs.343627	64779	16	85146740	85147268	529	1	1	4	0.125260035	0.527825585	no
MTMR15	PROMOTER	Hs.584863	22909	15	28983271	28983782	512	11	4	5	-0.007573471	0.896022688	no
MYCN	PROMOTER	Hs.25960	4613	2	15997774	15998094	321	2	3	2	-0.057458399	0.576591301	no
MYOCD	PROMOTER	Hs.567641	93649	17	12509786	12510029	244	1	4	2	0.459230949	0.043899206	no
MYOCD	PROMOTER	Hs.567641	93649	17	12508943	12509785	843	7	7	5	0.115518543	0.052198837	no
NFIL3	PROMOTER	Hs.79334	4783	9	93227058	93227308	251	1	0	1	0.125215837	0.617312832	no

NKD2	PROMOTER	Hs.240951	85409	5	1056657	1056985	329	0	1	1	-0.116076767	0.244030892	no
NMNAT2	PROMOTER	Hs.497123	23057	1	181654184	181654508	325	0	1	2	0.248511147	0.005964081	no
NR4A3	PROMOTER	Hs.279522	8013	9	101627226	101627606	381	1	1	3	0.319463321	0.069892655	no
NTNG2	PROMOTER	Hs.163642	84628	9	134028361	134028642	282	4	9	2	0.063366293	0.064035599	no
OR5BU1	PROMOTER	Hs.449973	401994	1	246921442	246922645	1204	9	10	7	-0.025323316	0.711354688	no
OSR1	PROMOTER	Hs.123933	130497	2	19424601	19425220	620	5	6	7	0.096843341	0.069627353	no
P2RX5	PROMOTER	Hs.408615	5026	17	3544799	3546490	1692	11	15	8	0.006793238	0.905648782	no
P2RY6	PROMOTER	Hs.16362	5031	11	72652878	72653579	702	5	5	4	0.012087465	0.862432322	no
PAX3	PROMOTER	Hs.42146	5077	2	222876016	222876986	971	3	2	1	0.286627547	0.08674201	no
PAX3	PROMOTER	Hs.42146	5077	2	222875363	222875684	322	0	5	3	0.193441842	0.02042941	no
PCDHB15	PROMOTER	Hs.130757	56121	5	140595215	140596821	1607	8	6	2	0.020229364	0.835298216	no
PCOLCE2	PROMOTER	Hs.8944	26577	3	144090641	144090974	334	0	5	2	0.131024969	0.03260398	no
PDE3A	PROMOTER	Hs.591150	5139	12	20412555	20413262	708	4	5	5	0.185889121	0.109774438	no
PDGFRA	PROMOTER	Hs.74615	5156	4	54788551	54789607	1057	5	1	4	-0.013918553	0.790415438	no
PLOD2	PROMOTER	Hs.477866	5352	3	147361838	147362130	293	0	2	2	0.136482688	0.220544346	no
POFUT2	PROMOTER	Hs.592164	23275	21	45533023	45533278	256	1	1	1	0.019719557	0.779153937	no
PPIL2	PROMOTER	Hs.438587	23759	22	20341004	20341659	656	0	2	1	-0.037228568	0.831708147	no
PRDM2	PROMOTER	Hs.371823	7799	1	13948160	13948842	683	11	18	3	-0.000450706	0.995701119	no
PRKG1	PROMOTER	Hs.654556	5592	10	52503241	52504092	852	7	4	7	0.033222406	0.700116579	no
PTCHD1	PROMOTER	Hs.319503	139411	X	23261044	23261624	581	7	3	6	0.070050237	0.312849792	no
QARS	PROMOTER	Hs.79322	5859	3	49117323	49117802	480	0	3	1	0.033943111	0.774558558	no
RAB3C	PROMOTER	Hs.482173	115827	5	57914150	57914516	367	1	0	1	0.120280266	0.35705435	no
RBM23	PROMOTER	Hs.4997	55147	14	22458441	22458618	178	1	0	1	0.000386996	0.99879184	no
RBP1	PROMOTER	Hs.529571	5947	3	140740790	140741449	660	5	9	6	0.232432625	0.01525796	no
RECK	PROMOTER	Hs.388918	8434	9	36026381	36027594	1214	8	9	9	0.006600079	0.922488705	no
RG9MTD1	PROMOTER	Hs.643184	54931	3	102762729	102763665	937	3	4	5	-0.023650199	0.582186764	no
RPS23	PROMOTER	Hs.527193	6228	5	81609873	81610089	217	4	1	2	-0.02845092	0.775300848	no
RRM2	PROMOTER	Hs.226390	6241	2	10177709	10178095	387	4	2	2	-0.041541369	0.586311001	no
RUNX1T1	PROMOTER	Hs.368431	862	8	93184958	93185358	401	1	5	3	0.17495174	0.055852607	no
SDK1	PROMOTER	Hs.655699	221935	7	3306223	3306872	650	0	4	1	0.056713271	0.719394955	no
SETD4	PROMOTER	Hs.606200	54093	21	36354669	36354909	241	3	3	1	-0.0224387	0.878011295	no
SIKE	PROMOTER		NA	1	115124923	115125419	497	3	2	1	-0.021642252	0.906285727	no
SLC26A5	PROMOTER	Hs.585146	375611	7	102874053	102874241	189	0	1	1	-0.088653138	0.242952246	no
SLC34A2	PROMOTER	Hs.479372	10568	4	25265707	25266435	729	7	1	3	-0.041393078	0.742672678	no
SLC35A3	PROMOTER	Hs.448979	23443	1	100207502	100208763	1262	6	10	9	-0.001534346	0.97064648	no
SLIT2	PROMOTER	Hs.699467	9353	4	19862221	19862705	485	5	3	5	0.143777652	0.020016522	no
SLIT2	PROMOTER	Hs.699467	9353	4	19862706	19863582	877	5	13	10	0.104439681	0.009410686	no
SMO	PROMOTER	Hs.437846	6608	7	128614941	128615896	956	8	9	4	0.083309365	0.06563989	no
SMPD3	PROMOTER	Hs.368421	55512	16	67040132	67040773	642	3	2	3	-0.009324417	0.954060361	no
SNPH	PROMOTER	Hs.323833	9751	20	1194507	1194835	329	0	1	1	-0.015234345	0.937859087	no
snR38C	PROMOTER		NA	17	72065662	72065883	222	2	1	2	0.063207006	0.143647329	no
SP8	PROMOTER	Hs.195922	221833	7	20797116	20797816	701	2	5	3	0.187754237	0.049214476	no
SPAG7	PROMOTER	Hs.90436	9552	17	4812152	4812725	574	3	1	3	-0.043976218	0.622254614	no
SPAG8	PROMOTER	Hs.256747	26206	9	35801250	35802349	1100	3	4	3	-0.001018878	0.991106331	no
ST8SIA1	PROMOTER	Hs.408614	6489	12	22379428	22379748	321	6	3	2	0.204317256	0.053386814	no
STCH	PROMOTER	Hs.352341	6782	21	14677817	14678101	285	1	1	2	0.016398528	0.925860271	no
SUNC1	PROMOTER	Hs.406741	256979	7	48041130	48042676	1547	12	13	13	0.068804943	0.043111078	no
SVOPL	PROMOTER	Hs.99414	136306	7	137998866	137999865	1000	7	0	5	0.009751803	0.880953545	no
SYPL2	PROMOTER	Hs.528366	284612	1	109810333	109810621	289	2	4	2	0.333242998	0.028312789	no
TCF4	PROMOTER	Hs.644653	6925	18	51407883	51408355	473	2	2	4	0.152076197	0.053107126	no
TMEM91	PROMOTER		NA	19	46573983	46575567	1585	5	8	4	0.032872124	0.500167741	no
TRAF1	PROMOTER	Hs.531251	7185	9	122730352	122731428	1077	7	19	10	-0.022008242	0.624136002	no
TRAM1L1	PROMOTER	Hs.570737	133022	4	118225682	118226395	714	1	6	6	0.036458771	0.551415158	no
TRIM8	PROMOTER	Hs.336810	81603	10	104391660	104391833	174	0	1	1	-0.058583187	0.314169043	no
TSTA3	PROMOTER	Hs.404119	7264	8	144771420	144771626	207	3	0	1	0.008118662	0.942356712	no
UBE2E2	PROMOTER	Hs.475688	7325	3	23218727	23219238	512	1	2	3	0.258747626	0.097926522	no
UGP2	PROMOTER	Hs.516217	7360	2	63922341	63922507	167	2	1	1	-0.051736858	0.5003006	no
WDR54	PROMOTER	Hs.643480	84058	2	74495441	74497050	1610	6	14	16	0.030800893	0.467602345	no
WNT2	PROMOTER	Hs.567356	7472	7	116751077	116751317	241	0	2	2	0.235632466	0.039882645	no
ZFP3	PROMOTER	Hs.48832	124961	17	4922101	4922964	864	7	10	7	0.107620625	0.13752716	no
ZNF225	PROMOTER	Hs.279567	7768	19	49308833	49309510	678	2	2	2	0.00082216	0.995701119	no
ZNF331	PROMOTER	Hs.185674	55422	19	58749084	58750957	1874	11	6	12	-0.013140805	0.826722701	no
ZNF345	PROMOTER	Hs.362324	25850	19	42032895	42033131	237	0	3	1	0.202758089	0.01301757	no
ZNF347	PROMOTER	Hs.467239	84671	19	58354050	58354331	282	2	4	3	0.244948291	0.021550811	no
ZNF382	PROMOTER	Hs.631591	84911	19	41787246	41788878	1633	14	13	14	0.039168192	0.28350953	no
ZNF470	PROMOTER	Hs.204449	388566	19	61769709	61770986	1278	8	8	10	0.04377629	0.335433933	no
ZNF470	PROMOTER	Hs.204449	388566	19	61770987	61771665	679	0	5	2	0.171129335	0.07050263	no
ZNF502	PROMOTER	Hs.224843	91392	3	44728933	44729161	229	3	2	1	0.181454686	0.016063282	no
ZNF577	PROMOTER	Hs.148322	84765	19	57082594	57083183	590	5	2	5	0.116303955	0.219760219	no
GCM2-NO145	DIVERGENT_PROMOTER		NA	6	10990480	10991342	863	6	4	4	0.030414008	0.558963017	yes
hsa-mir-212-HIC1	DIVERGENT_PROMOTER		NA	17	1902162	1903130	969	0	2	2	-0.068721385	0.4474862	yes
C1orf147-RASSF5	DIVERGENT_PROMOTER		NA	1	204746527	204747456	930	7	6	7	0.041451205	0.48057829	no
CCT6B	DIVERGENT_PROMOTER	Hs.73072	10693	17	30312124	30312768	645	4	4	7	0.051187715	0.494144826	no
CKB-C14orf172	DIVERGENT_PROMOTER		NA	14	103058949	103059558	610	7	5	7	0.113012313	0.040059873	no
CKLF	DIVERGENT_PROMOTER	Hs.15159	51192	16	65143776	65144563	788	8	2	6	0.007029557	0.940460405	no
EVX2-HOXD13	DIVERGENT_PROMOTER		NA	2	176658363	176658723	361	4	1	3	0.360700288	6.85E-06	no
FLJ46082	DIVERGENT_PROMOTER	Hs.201709	389799	9	134274734	134276471	1738	11	18	7	-0.069112973	0.092118767	no
GCN5L2-HSPB9	DIVERGENT_PROMOTER		NA	17	37527850	37528126	277	2	2	2	0.013825542	0.923284756	no
HIST1H2BB-HIST1H3C	DIVERGENT_PROMOTER		NA	6	26151974	26152986	1013	3	3	4	0.068673555	0.495521576	no
HIST1H3G-HIST1H2BI	DIVERGENT_PROMOTER		NA	6	26380364	26380773	410	4	3	3	0.052151637	0.553906172	no
LMOD1-TIMM17A	DIVERGENT_PROMOTER		NA	1	200190470	200191191	722	3	4	2	0.03073394	0.668256763	no
MGC20983	DIVERGENT_PROMOTER	Hs.124010	115948	19	11406439	11407281	843	3	1	4	0.018605962	0.79048002	no
MLH1	DIVERGENT_PROMOTER	Hs.195364	4292	3	37009074	37010186	1113	6	5	12	0.196364213	0.100285787	no
SNX1	DIVERGENT_PROMOTER	Hs.188634	6642	15	62175042	62176036	995	3	9	9	0.018384761	0.794395527	no
ALX4	INSIDE	Hs.436055	60529	11	44283799	44284349	551	4	4	5	0.210161965	0.025013309	yes
ARHGAP9	INSIDE	Hs.437126	64333	12	56155101	56156473	1373	8	12	15	0.019803915	0.671202085	yes
B4GALNT1	INSIDE	Hs.591019	2583	12	56312448	56313083	636	9	10	6	0.016771714	0.724462577	yes
B4GALNT1	INSIDE	Hs.591019	2583	12	56307743	56308507	765	5	5	7	0.262786857	0.001343836	yes
BMP6	INSIDE	Hs.285671	654	6	7673194	7674075	882	3	8	3	0.20808914	0.07425829	yes
BMP6	INSIDE	Hs.285671	654	6	7671226	7671603	378	8	4	3	0.130188011	0.077549775	yes
C21orf63	INSIDE	Hs.208358	59271	21	32707500	32707946	447	4	0	2	-0.075428366	0.72965197	yes
CALCA	INSIDE	Hs.37058	796	11	14949872	14950696	825	2	1	4	0.018735971	0.887836841	yes
CDH11	INSIDE	Hs.116471	1009	16	63712697	63713109	413	3	8	4	0.135460012	0.123860549	yes
COCH	INSIDE	Hs.21016	1690	14	30414211	30414601	391	1	2	3	0.376543178	0.000963469	yes

DKFZP779L1853	INSIDE	Hs.640467	643162	12	46878661	46879044	384	2	4	3	0.29088007	0.012347022	yes
DLX3	INSIDE	Hs.134194	1747	17	45424583	45426362	1780	13	11	8	-0.013707786	0.730309735	yes
DSC3	INSIDE	Hs.41690	1825	18	26875421	26876732	1312	14	11	13	0.155302709	0.067141791	yes
DUOX2	INSIDE	Hs.71377	50506	15	43192510	43193155	646	1	1	3	0.142592815	0.103446698	yes
DUOXA2	INSIDE	Hs.497987	405753	15	43193943	43194818	876	2	4	3	0.075803417	0.594364864	yes
EPHB3	INSIDE	Hs.2913	2049	3	185763281	185763779	499	1	3	2	-0.073346583	0.570380759	yes
EYA4	INSIDE	Hs.661127	2070	6	133605174	133605603	430	1	1	4	0.115794304	0.009177566	yes
FLJ44815	INSIDE	Hs.514090	400591	17	29929984	29930462	479	3	5	5	0.040464772	0.429130913	yes
FLJ45983	INSIDE	Hs.669736	399717	10	8133177	8134109	933	4	17	10	0.071822545	0.097777512	yes
FOXF2	INSIDE	Hs.484423	2295	6	1338964	1339624	661	1	14	3	0.06728969	0.10510142	yes
FRAT1	INSIDE	Hs.126057	10023	10	99070249	99070534	286	3	2	3	0.142240329	0.006192178	yes
GATA2	INSIDE	Hs.367725	2624	3	129691884	129692479	596	2	4	6	0.098829053	0.043111078	yes
HAND1	INSIDE	Hs.152531	9421	5	153836059	153836436	378	1	4	3	0.071408323	0.463590056	yes
HAND1	INSIDE	Hs.152531	9421	5	153837431	153837797	367	2	5	3	0.319172342	0.01236766	yes
HBA1	INSIDE	Hs.449630	3039	16	167000	167407	408	4	2	1	0.147928638	0.05729434	yes
ISL1	INSIDE	Hs.505	3670	5	50718406	50719378	973	1	3	4	0.185289778	0.004841373	yes
ISL2	INSIDE	Hs.444677	64843	15	74420926	74421736	811	4	8	7	-0.026481053	0.543396243	yes
JMJD3	INSIDE	Hs.223678	23135	17	7697264	7697706	443	5	5	4	0.04912297	0.510508728	yes
KCNAB3	INSIDE	Hs.435074	9196	17	7766400	7768661	2262	2	10	5	0.009436347	0.915367682	yes
LBXCOR1	INSIDE	Hs.451224	390598	15	65908227	65908844	618	7	2	6	0.016421794	0.850704781	yes
LBXCOR1	INSIDE	Hs.451224	390598	15	65905147	65906032	886	7	3	6	0.176228181	0.000712418	yes
LOC375449	INSIDE		NA	5	65928841	65929437	597	5	1	5	0.110860401	0.121834832	yes
LOC439985	INSIDE	Hs.704151	439985	10	76827314	76827568	255	1	2	2	-0.009672827	0.940889325	yes
MAPK4	INSIDE	Hs.433728	5596	18	46341077	46341624	548	2	7	6	0.093772654	0.212578359	yes
MEGF11	INSIDE	Hs.438250	84465	15	64331251	64331650	400	1	9	2	0.118024285	0.051045233	yes
MKX	INSIDE	Hs.128193	283078	10	28071999	28073071	1073	6	14	11	0.158403547	0.0382346	yes
MKX	INSIDE	Hs.128193	283078	10	28073072	28073988	917	17	4	8	0.183364404	0.005057507	yes
MKX	INSIDE	Hs.128193	283078	10	28071089	28071309	221	3	2	2	0.191179816	0.005423377	yes
NKX2-2	INSIDE	Hs.516922	4821	20	21439699	21440084	386	1	3	3	0.087374175	0.230189084	yes
NKX2-8	INSIDE	Hs.234763	26257	14	36120670	36120916	247	1	2	2	0.113606294	0.153453405	yes
NOL4	INSIDE	Hs.514795	8715	18	30057056	30057488	433	2	4	4	0.19218287	0.158750636	yes
NOL4	INSIDE	Hs.514795	8715	18	30056745	30057055	311	2	3	2	0.063151186	0.516411264	yes
NR3C1	INSIDE	Hs.122926	2908	5	142762338	142763139	802	9	11	8	0.077499627	0.026115631	yes
NRN1	INSIDE	Hs.103291	51299	6	5951340	5951971	632	4	5	3	0.062135153	0.282855005	yes
OTP	INSIDE	Hs.202247	23440	5	76961846	76962779	934	3	10	9	0.179505033	0.012503691	yes
PAX1	INSIDE	Hs.349082	5075	20	21642186	21642422	237	0	1	1	0.431375123	0.002587308	yes
PAX2	INSIDE	Hs.155644	5076	10	102498612	102498922	311	1	2	2	0.116212465	0.048739779	yes
PAX8	INSIDE	Hs.469728	7849	2	113751020	113752772	1753	17	24	17	0.010353352	0.808156439	yes
PAX8	INSIDE	Hs.469728	7849	2	113749927	113750381	455	2	4	3	0.166285842	0.103890155	yes
PCDHGC5	INSIDE		NA	5	140851626	140853100	1475	10	15	11	0.034636604	0.334460348	yes
RAB31	INSIDE	Hs.99528	11031	18	9698902	9699163	262	2	1	2	0.135247751	0.082358027	yes
SARM1	INSIDE	Hs.532781	23098	17	23731857	23733178	1322	11	3	8	-0.008845652	0.91308546	yes
SCARF2	INSIDE	Hs.474251	91179	22	19108894	19109353	460	1	6	1	0.036440363	0.88748256	yes
SHC4	INSIDE	Hs.642615	399694	15	47042094	47042663	570	1	5	5	0.328134955	0.030336447	yes
SIX1	INSIDE	Hs.633506	6495	14	60185144	60185889	746	7	4	6	0.111722188	0.035033567	yes
SLC12A5	INSIDE	Hs.21413	57468	20	44118075	44119361	1287	2	4	2	0.017330366	0.797099958	yes
SLC37A2	INSIDE	Hs.352661	219855	11	124438383	124439227	845	5	7	5	-0.007420042	0.912515915	yes
SLC5A5	INSIDE	Hs.584804	6528	19	17844606	17844972	367	3	4	2	0.389137094	0.013647012	yes
ST8SIA4	INSIDE	Hs.308628	7903	5	100264656	100265360	705	0	4	3	0.308959942	0.005181622	yes
SYT15	INSIDE	Hs.696346	83849	10	46389863	46390520	658	5	5	6	0.08752902	0.043102829	yes
TBX2	INSIDE	Hs.705451	6909	17	56834955	56835612	658	5	3	5	0.21339337	0.128011454	yes
TBX2	INSIDE	Hs.705451	6909	17	56833274	56833487	214	0	1	2	0.27263982	0.000493318	yes
TCF8	INSIDE		NA	10	31649152	31649545	394	3	7	4	0.386255467	0.006059859	yes
TFAP2B	INSIDE	Hs.33102	7021	6	50918445	50919072	628	1	4	6	0.060149864	0.234725632	yes
TFAP2D	INSIDE	Hs.434107	83741	6	50799946	50800517	572	4	3	6	0.183227839	2.99E-05	yes
TRIM36	INSIDE	Hs.519514	55521	5	114542612	114542992	381	3	3	3	0.078318671	0.327591566	yes
TRIM36	INSIDE	Hs.519514	55521	5	114542993	114543749	757	5	4	7	0.039234343	0.654806373	yes
VGLL2	INSIDE	Hs.99324	245806	6	117693596	117694217	622	2	4	4	0.144869541	0.185752465	yes
WNT9B	INSIDE	Hs.326420	7484	17	42283921	42285064	1144	7	10	10	0.040380612	0.173101182	yes
ZIC1	INSIDE	Hs.598590	7545	3	148612866	148613353	488	1	1	4	0.173209036	0.012581622	yes
ABHD4	INSIDE	Hs.445665	63874	14	22137085	22137639	555	4	3	5	-0.013905989	0.805978324	no
ADAMTS10	INSIDE	Hs.657508	81794	19	8563205	8563420	216	1	0	2	0.094571515	0.438402571	no
ADAMTS19	INSIDE	Hs.23751	171019	5	128824265	128824660	396	2	0	4	0.147068693	0.512584319	no
ADD2	INSIDE	Hs.188528	119	2	70848610	70848803	194	2	0	2	0.13726866	0.002348991	no
AKAP8	INSIDE	Hs.631640	10270	19	15341002	15341342	341	3	1	3	0.109562114	0.366589606	no
ALX3	INSIDE	Hs.669953	257	1	110411603	110412414	812	8	7	7	0.072513242	0.025563638	no
ANKRD38	INSIDE	Hs.283398	163782	1	62556369	62557203	835	3	2	5	0.07212376	0.297401975	no
ARHGEF19	INSIDE	Hs.591532	128272	1	16406381	16406606	226	1	0	1	-0.043868323	0.893731529	no
ARHGEF6	INSIDE	Hs.522795	9459	X	135676642	135677301	660	6	4	7	0.040139064	0.414917522	no
ARHGEF7	INSIDE	Hs.508738	8874	13	110704665	110706107	1443	0	17	1	0.066899003	0.690227025	no
ATP11A	INSIDE	Hs.29189	23250	13	112534677	112535659	983	4	3	7	0.06554102	0.438607616	no
ATP11A	INSIDE	Hs.29189	23250	13	112522017	112523049	1033	3	5	5	0.103288847	0.256556081	no
BAPX1	INSIDE		NA	4	13152424	13153040	617	4	6	4	0.283427392	0.002425543	no
BC37295_3	INSIDE	Hs.458438	90485	19	61873468	61875702	2235	6	8	8	-0.006276275	0.923084085	no
BCL9	INSIDE	Hs.415209	607	1	145537009	145538073	1065	3	10	2	0.032365936	0.44843478	no
BMP7	INSIDE	Hs.473163	655	20	55272632	55273192	561	8	7	6	-0.006514481	0.936911024	no
BNIP3	INSIDE	Hs.144873	664	10	133643267	133643868	602	4	6	4	0.017295483	0.795279975	no
C1orf164	INSIDE	Hs.456557	55182	1	44854835	44855693	859	7	8	4	0.089310278	0.358112051	no
C20orf160	INSIDE	Hs.382151	140706	20	30082330	30083304	975	2	10	4	0.032702727	0.49352378	no
C20orf166	INSIDE	Hs.86507	128826	20	60562224	60563244	1021	3	6	2	-0.004697105	0.948475013	no
C20orf54	INSIDE	Hs.283865	113278	20	688747	690661	1915	3	8	8	-0.031311718	0.506734028	no
C3orf21	INSIDE	Hs.478741	152002	3	196472109	196472320	212	1	0	1	0.046670029	0.703089944	no
C4orf31	INSIDE	Hs.90250	79625	4	122212340	122212599	260	1	0	2	0.088293656	0.412383181	no
C9orf138	INSIDE	Hs.98943	158297	9	19017809	19018522	714	2	4	2	-0.097262669	0.183231016	no
CAND2	INSIDE	Hs.343664	23066	3	12826396	12827181	786	3	5	5	0.014561572	0.753526877	no
CAV1	INSIDE	Hs.74034	857	7	115953622	115954375	754	4	1	5	0.106193162	0.25314239	no
CCDC102A	INSIDE	Hs.644611	92922	16	56125463	56127820	2358	7	9	6	0.00521779	0.948062104	no
CCL28	INSIDE	Hs.656904	56477	5	43432912	43433199	288	6	3	2	0.24946962	0.106826799	no
CCL28	INSIDE	Hs.656904	56477	5	43432537	43432911	375	4	1	2	0.359321992	0.003756626	no
CCNL2	INSIDE	Hs.515704	81669	1	1323417	1325123	1707	18	15	2	-0.072610539	0.173547706	no
CENTA1	INSIDE	Hs.644629	11033	7	942902	944008	1107	0	16	1	-0.088512029	0.246225065	no
CLDN1	INSIDE	Hs.439060	9076	3	191522799	191522953	155	2	1	1	0.063077898	0.469251543	no
CLDN4	INSIDE	Hs.647036	1364	7	72883378	72884400	1023	4	6	6	-0.05826221	0.555634668	no
COL12A1	INSIDE	Hs.101302	1303	6	75851394	75851902	509	6	3	4	0.138777368	0.399703164	no

COL18A1	INSIDE	Hs.517356	80781	21	45720114	45720607	494	6	2	4	-0.019196995	0.76747684	no
COL4A2	INSIDE	Hs.508716	1284	13	109758900	109759172	273	1	3	2	0.239721699	0.005351228	no
COL5A1	INSIDE	Hs.210283	1289	9	136873791	136874453	663	2	5	6	0.023711772	0.825218309	no
CPSF1	INSIDE	Hs.493202	29894	8	145591998	145592867	870	4	8	4	-0.023239325	0.767727861	no
CR2	INSIDE	Hs.445757	1380	1	205694271	205694997	727	8	7	8	0.10118793	0.130115707	no
CRABP1	INSIDE	Hs.346950	1381	15	76420341	76420862	522	2	2	5	0.300253127	0.019036515	no
CRABP1	INSIDE	Hs.346950	1381	15	76421132	76421321	190	0	1	1	0.210574591	0.036048649	no
CRAMP1L	INSIDE	Hs.603676	57585	16	1615591	1616750	1160	1	3	6	-0.011477768	0.890415843	no
CREBBP	INSIDE	Hs.459759	1387	16	3715771	3716613	843	2	3	6	-0.036462807	0.565711104	no
CSMD2	INSIDE	Hs.656915	114784	1	34401058	34401661	604	3	4	4	0.050391622	0.55980255	no
CTDP1	INSIDE	Hs.465490	9150	18	75554429	75556137	1709	4	8	2	-0.056490037	0.257655625	no
CTPS2	INSIDE	Hs.227049	56474	X	16639523	16639906	384	1	1	1	0.023726397	0.896361135	no
CUGBP2	INSIDE	Hs.309288	10659	10	11247148	11247520	373	0	2	4	0.012172196	0.925669638	no
CYP1B1	INSIDE	Hs.154654	1545	2	38156559	38156826	268	1	1	1	0.463505636	0.03542507	no
DDIT4L	INSIDE	Hs.480378	115265	4	101329831	101330988	1158	5	12	7	0.047142325	0.379827382	no
DENND1C	INSIDE	Hs.236449	79958	19	6426524	6427133	610	4	1	6	0.046935054	0.795279975	no
DENND2C	INSIDE	Hs.654928	163259	1	115013666	115014093	428	3	3	4	0.174126691	0.028346729	no
DLX1	INSIDE	Hs.407015	1745	2	172659363	172659594	232	0	1	1	0.153486455	0.20211396	no
DLX2	INSIDE	Hs.419	1746	2	172674353	172675068	716	2	5	8	0.110340745	0.027058172	no
DMBT1	INSIDE	Hs.279611	1755	10	124391467	124393320	1854	0	3	6	-0.056778489	0.032367529	no
DMPK	INSIDE	Hs.631596	1760	19	50974944	50975652	709	0	1	1	0.059372451	0.54153812	no
DNAJC16	INSIDE	Hs.655410	23341	1	15725894	15726441	548	4	10	5	0.008867224	0.914384114	no
DUSP4	INSIDE	Hs.417962	1846	8	29250716	29251058	343	2	1	1	0.069216085	0.734305422	no
EGFL6	INSIDE	Hs.12844	25975	X	13498112	13498612	501	0	3	1	0.067624407	0.611447161	no
EMX1	INSIDE	Hs.516090	2016	2	73004678	73005679	1002	7	7	10	0.039340066	0.265092457	no
ENTPD4	INSIDE	Hs.444389	9583	8	23370487	23370641	155	1	0	1	0.03554071	0.797889709	no
EPHA3	INSIDE	Hs.123642	2042	3	89246558	89246762	205	2	0	2	0.034610781	0.798190347	no
ERGIC1	INSIDE	Hs.509163	57222	5	172193800	172194506	707	5	11	4	-0.009428377	0.87385163	no
FAM102B	INSIDE	Hs.200230	284611	1	108904600	108904989	390	1	0	2	-0.015868631	0.90446689	no
FAM124A	INSIDE	Hs.71913	220108	13	50723549	50724673	1125	0	6	5	-0.021274547	0.707735426	no
FAM19A5	INSIDE	Hs.436854	25817	22	47532700	47533272	573	3	7	6	-0.136367839	0.020073838	no
FAM5B	INSIDE	Hs.495918	57795	1	175416959	175418066	1108	5	9	6	0.093238301	0.260281593	no
FBXL10	INSIDE	Hs.524800	84678	12	120375078	120375708	631	4	8	6	0.036040006	0.662297697	no
FBXL18	INSIDE	Hs.623974	80028	7	5501005	5502340	1336	1	10	5	-0.067585959	0.306386463	no
FIBP	INSIDE	Hs.7768	9158	11	65410457	65412231	1775	2	3	3	-0.034749198	0.661494223	no
FLJ10769	INSIDE	Hs.408324	55739	13	110088549	110089021	473	3	2	5	0.107622238	0.089004154	no
FLJ30058	INSIDE		NA	X	130034242	130034847	606	1	2	4	0.064826077	0.168610059	no
FLJ37440	INSIDE		NA	2	112655492	112656246	755	7	6	6	0.253003875	0.013651021	no
FLJ45530	INSIDE	Hs.120392	400555	16	87163385	87163912	528	5	4	2	-0.004198882	0.96335635	no
FOXD4L4	INSIDE	Hs.534644	349334	9	69666954	69668217	1264	6	8	1	0.316727799	0.00899833	no
FSTL1	INSIDE	Hs.269512	11167	3	121651785	121652042	258	1	0	2	0.112613851	0.381021068	no
FSTL1	INSIDE	Hs.269512	11167	3	121652051	121652555	505	9	6	6	0.071897132	0.165723711	no
GABBR1	INSIDE	Hs.167017	2550	6	29703026	29704008	983	9	6	5	0.046570624	0.317105878	no
GALR2	INSIDE	Hs.666366	8811	17	71582754	71583229	476	6	0	4	0.280962734	0.110220839	no
GATAD2A	INSIDE	Hs.696033	54815	19	19472638	19473316	679	1	4	6	0.057196883	0.684059409	no
GBX2	INSIDE	Hs.184945	2637	2	236739948	236740439	492	1	1	4	0.19819901	0.021594218	no
GBX2	INSIDE	Hs.184945	2637	2	236739143	236739947	805	3	4	8	0.186853783	0.004350111	no
GFI1	INSIDE	Hs.73172	2672	1	92719872	92720488	617	1	6	7	0.194400537	0.190785289	no
GFI1B	INSIDE	Hs.553160	8328	9	134854651	134855268	618	1	5	4	-4.48E-06	0.999964982	no
GNAS	INSIDE	Hs.125898	2778	20	56848751	56849946	1196	9	7	12	0.099570434	0.036969182	no
GNG8	INSIDE	Hs.283961	94235	19	51828916	51829799	884	2	5	3	0.046048468	0.519139417	no
GPR123	INSIDE	Hs.435183	84435	10	134746728	134747160	433	2	2	4	-0.046199799	0.766493893	no
GPR123	INSIDE	Hs.435183	84435	10	134763049	134763758	710	0	6	3	-0.035226527	0.620672991	no
GPR133	INSIDE	Hs.656754	283383	12	130079402	130079695	294	1	0	1	-0.09519914	0.484293036	no
GSH2	INSIDE		NA	4	54661583	54662079	497	7	1	3	0.221139291	0.002448761	no
GTF2IRD1	INSIDE	Hs.647056	9569	7	73532901	73533419	519	1	3	2	-0.061690864	0.297279625	no
HOXA1	INSIDE	Hs.67397	3198	7	27100395	27100983	589	2	2	5	0.117659934	0.202723324	no
HOXA3	INSIDE	Hs.659337	3200	7	27113669	27115144	1476	6	12	10	0.071831467	0.013188101	no
IGSF22	INSIDE	Hs.434152	283284	11	18683842	18684419	578	6	1	6	0.221063531	0.014256009	no
INHBB	INSIDE	Hs.1735	3625	2	120821026	120821409	384	1	3	2	0.045449501	0.60512508	no
INSR	INSIDE	Hs.465744	3643	19	7218659	7219140	482	0	7	1	-0.061396727	0.784817921	no
ISL1	INSIDE	Hs.505	3670	5	50715086	50715715	630	2	0	1	0.085244844	0.50187151	no
ITGB2	INSIDE	Hs.375957	3689	21	45147433	45148482	1050	2	6	4	-0.052268301	0.269189368	no
KATNAL2	INSIDE	Hs.404137	83473	18	42780750	42781032	283	1	2	3	0.739544769	0.001179834	no
KCTD16	INSIDE	Hs.693927	57528	5	143564694	143565085	392	3	1	3	0.107541791	0.085122498	no
KHSRP	INSIDE	Hs.699378	8570	19	6365285	6365695	411	4	4	4	0.042853235	0.601726505	no
KIAA0427	INSIDE	Hs.145230	9811	18	44541459	44542185	727	1	2	4	-0.042740181	0.730309735	no
KIAA0664	INSIDE	Hs.22616	23277	17	2539485	2539875	391	0	3	2	0.039523971	0.784845875	no
KIAA1529	INSIDE	Hs.435629	57653	9	99109233	99109770	538	2	5	1	0.243208717	0.446715323	no
KLF9	INSIDE	Hs.150557	687	9	72217215	72217379	165	2	1	1	-0.056278382	0.323787383	no
KRBA1	INSIDE	Hs.299560	84626	7	149047920	149048612	693	0	1	1	-0.074055507	0.556702454	no
LDLRAD3	INSIDE	Hs.700909	143458	11	35922893	35923425	533	2	3	2	0.109731527	0.211993283	no
LHX4	INSIDE	Hs.658487	89884	1	178467921	178468646	726	2	1	6	0.270699306	0.043451258	no
LHX4	INSIDE	Hs.658487	89884	1	178470713	178471227	515	5	7	5	-0.007928413	0.923574112	no
LHX6	INSIDE	Hs.103137	26468	9	124029808	124030013	206	1	0	1	0.119892161	0.65342468	no
LHX6	INSIDE	Hs.103137	26468	9	124029344	124029714	371	2	6	3	0.036483632	0.799479503	no
LHX9	INSIDE	Hs.442578	56956	1	196153095	196153801	707	3	3	2	0.144267293	0.097151588	no
LMX1A	INSIDE	Hs.667312	4009	1	163470933	163471402	470	2	1	3	0.08207943	0.287767935	no
LOC400657	INSIDE	Hs.61508	400657	18	70415406	70415892	487	2	4	5	-0.028755603	0.491167086	no
LOC401387	INSIDE		NA	7	91646009	91646731	723	6	7	5	0.06044983	0.1886299	no
LOX	INSIDE	Hs.102267	4015	5	121440320	121441878	1559	16	15	16	0.064332471	0.241980504	no
LPHN2	INSIDE	Hs.24212	23266	1	82040847	82041672	826	4	7	8	0.037355766	0.344338735	no
LPHN3	INSIDE	Hs.28391	23284	4	62065426	62065995	570	1	1	5	0.013891585	0.835027181	no
LRP1B	INSIDE	Hs.656461	53353	2	142604599	142605113	515	2	2	5	0.202253966	0.086800216	no
LTBP4	INSIDE	Hs.466766	8425	19	45821663	45822019	357	0	2	4	-0.028880265	0.759674035	no
MAFF	INSIDE	Hs.517617	23764	22	36939447	36942044	2598	12	12	7	-0.012193655	0.829625004	no
MARVELD2	INSIDE	Hs.657687	153562	5	68747300	68747534	235	2	2	1	-0.154784952	0.026285137	no
MATN4	INSIDE	Hs.278489	8785	20	43359702	43360307	606	2	3	4	-0.08150363	0.114386889	no
MBNL2	INSIDE	Hs.657347	10150	13	96796487	96797617	1131	4	4	6	0.047237965	0.679592744	no
MEGF10	INSIDE	Hs.438709	84466	5	126654806	126655234	429	0	1	1	0.167573206	0.246796879	no
METRNL	INSIDE	Hs.591142	284207	17	78635510	78636204	695	0	4	4	-0.113870851	0.03151096	no
MGC15523	INSIDE	Hs.352240	124565	17	76873545	76874259	715	2	5	4	0.086103483	0.145161499	no
MKX	INSIDE	Hs.128193	283078	10	28071310	28071982	673	5	10	6	0.115005744	0.013637583	no
MLH1	INSIDE	Hs.195364	4292	3	37010201	37010368	168	2	2	1	0.151352525	0.17686018	no

MMP21	INSIDE	Hs.314141	118856	10	127454030	127454442	413	1	2	4	-0.049154435	0.691763478	no
MN1	INSIDE	Hs.268515	4330	22	26525521	26526428	908	3	10	11	0.019944412	0.625036234	no
MN1	INSIDE	Hs.268515	4330	22	26524857	26525520	664	5	7	5	0.05737103	0.522844871	no
MRPL43	INSIDE	Hs.421848	84545	10	102736208	102736719	512	2	1	3	-0.022938325	0.778734678	no
NAPSA	INSIDE	Hs.512843	9476	19	55553504	55554108	605	5	3	5	0.277651819	0.027994183	no
NBPF1	INSIDE	Hs.445080	55672	1	16811741	16812715	975	15	4	1	0.146584663	0.312194059	no
NFATC1	INSIDE	Hs.534074	4772	18	75319942	75320476	535	1	2	4	-0.068146306	0.340619058	no
NFATC1	INSIDE	Hs.534074	4772	18	75348686	75349174	489	5	2	2	-0.088020323	0.578869759	no
NFATC1	INSIDE	Hs.534074	4772	18	75309373	75310098	726	1	5	6	-0.013769107	0.913433882	no
NFATC2	INSIDE	Hs.699441	4773	20	49542445	49542683	239	1	3	2	0.097202271	0.336113586	no
NGEF	INSIDE	Hs.97316	25791	2	233471968	233474137	2170	2	4	1	-0.000812026	0.994194169	no
NHEJ1	INSIDE	Hs.225988	79840	2	219732913	219733072	160	1	1	1	0.128908599	0.074025788	no
NKX6-1	INSIDE	Hs.546270	4825	4	85633773	85634023	251	1	0	2	0.0600891	0.440184078	no
NOB1	INSIDE	Hs.271695	28987	16	68333407	68333777	371	0	2	1	0.033157348	0.759245059	no
NOC3L	INSIDE	Hs.74899	64318	10	96111954	96112595	642	2	1	1	-0.293932344	0.04414719	no
NOTCH3	INSIDE	Hs.8546	4854	19	15145793	15146700	908	2	6	5	-0.009923295	0.879333575	no
NOX5	INSIDE	Hs.657932	79400	15	67110637	67111533	897	3	4	5	0.058957667	0.558200632	no
NPTX2	INSIDE	Hs.3281	4885	7	98085361	98085600	240	5	4	2	0.315209741	0.028071095	no
NPY1R	INSIDE	Hs.519057	4886	4	164472772	164473118	347	3	3	2	0.114698858	0.285683328	no
NPY5R	INSIDE	Hs.519058	4889	4	164484465	164485320	856	11	13	10	0.015538643	0.789156353	no
NTF3	INSIDE	Hs.99171	4908	12	5473806	5474449	644	3	3	6	-0.032656975	0.638314444	no
NTNG1	INSIDE	Hs.657434	22854	1	107485076	107485290	215	0	1	1	0.24242939	0.003690434	no
OCA2	INSIDE	Hs.654411	4948	15	26017457	26017999	543	3	2	3	0.058609339	0.40756542	no
OSBP2	INSIDE	Hs.517546	23762	22	29547883	29548276	394	1	1	1	0.023412093	0.800965355	no
PCMTD2	INSIDE	Hs.473317	55251	20	62371046	62373405	2360	2	2	4	-0.050388269	0.644412582	no
PCOLCE2	INSIDE	Hs.8944	26577	3	144089894	144090073	180	1	1	1	0.24849732	0.038016321	no
PDE3A	INSIDE	Hs.591150	5139	12	20414343	20414575	233	0	2	2	0.225734656	0.08818462	no
PDE4DIP	INSIDE	Hs.584841	9659	1	143786728	143787883	1156	7	10	6	0.113214901	0.019737428	no
PDLIM3	INSIDE	Hs.85862	27295	4	186692760	186693165	406	3	3	3	0.051862229	0.418832869	no
PDSS2	INSIDE	Hs.486095	57107	6	107887341	107887497	157	0	2	1	-0.000741	0.996274098	no
PFKP	INSIDE	Hs.26010	5214	10	3150867	3151126	260	3	4	2	-0.179090474	0.519444076	no
PKLR	INSIDE	Hs.95990	5313	1	153530205	153532707	2503	6	16	16	0.033384044	0.463393344	no
PLEKHC1	INSIDE	Hs.509343	10979	14	52486734	52486897	164	1	0	1	0.027278955	0.864554114	no
PODNL1	INSIDE	Hs.448497	79883	19	13904895	13905364	470	3	5	4	0.066929012	0.75329766	no
POU6F2	INSIDE	Hs.137106	11281	7	39420336	39420779	444	3	2	4	0.392032616	0.009456006	no
PRDM16	INSIDE	Hs.99500	63976	1	3147344	3148382	1039	4	10	9	-0.029657784	0.77915414	no
PRDM16	INSIDE	Hs.99500	63976	1	3296534	3297169	636	4	4	3	0.007759027	0.940778981	no
PRDM16	INSIDE	Hs.99500	63976	1	3027796	3028970	1175	5	12	6	-0.060590462	0.110650709	no
PSTPIP1	INSIDE	Hs.129758	9051	15	75111485	75113359	1875	1	8	3	-0.031765375	0.689527355	no
PTMS	INSIDE	Hs.504613	5763	12	6747113	6747853	741	1	3	1	-0.026140516	0.9567876	no
PTPRZ1	INSIDE	Hs.489824	5803	7	121300690	121301293	604	5	2	6	0.29485443	0.001146248	no
PTRF	INSIDE	Hs.437191	284119	17	37811410	37811705	296	2	1	2	0.061537937	0.679592744	no
PYY	INSIDE	Hs.169249	5697	17	39427228	39427510	283	1	0	2	0.138816172	0.586149607	no
RASA3	INSIDE	Hs.655219	22821	13	113856170	113857698	1529	6	1	1	-0.028444796	0.866801492	no
RASA3	INSIDE	Hs.655219	22821	13	113793033	113795829	2797	5	8	3	-0.009942099	0.918802666	no
RASIP1	INSIDE	Hs.233955	54922	19	53930012	53930793	782	1	6	6	0.065304531	0.599277962	no
RBM11	INSIDE	Hs.283828	54033	21	14510729	14511001	273	1	0	2	0.070688306	0.687160449	no
RCN3	INSIDE	Hs.567550	57333	19	54728458	54729780	1323	12	10	8	-0.008847527	0.891736341	no
RPH3AL	INSIDE	Hs.651925	9501	17	122549	123660	1112	1	3	1	-0.042725035	0.812125407	no
RTBDN	INSIDE	Hs.21162	83546	19	12796820	12797764	945	2	3	4	0.0236367	0.685331885	no
SERPINB9	INSIDE	Hs.104879	5272	6	2836823	2837175	353	1	1	3	0.105721149	0.504144288	no
SH2D3C	INSIDE	Hs.306412	10044	9	129556088	129556465	378	4	4	4	0.000975845	0.995701119	no
SH2D3C	INSIDE	Hs.306412	10044	9	129543650	129544434	785	4	4	5	0.018648427	0.805512971	no
SLC1A2	INSIDE	Hs.502338	6506	11	35397192	35397706	515	9	6	6	0.160705754	0.02288148	no
SLC26A4	INSIDE	Hs.571246	5172	7	107088619	107089828	1210	10	5	12	0.082164344	0.075908771	no
SLC5A1	INSIDE	Hs.1964	6523	22	30769205	30769490	286	1	1	2	-0.1955249	0.21772455	no
SLCO5A1	INSIDE	Hs.443609	81796	8	70906445	70907033	589	1	2	6	0.005753365	0.940652774	no
SMOC2	INSIDE	Hs.487200	64094	6	168660241	168662979	2739	6	10	5	-0.034803791	0.506084252	no
SMTNL2	INSIDE	Hs.441709	342527	17	4433627	4434407	781	3	4	3	0.012740711	0.87347824	no
SOCS2	INSIDE	Hs.485572	8835	12	92490469	92491797	1329	15	10	15	0.027620534	0.488763515	no
SOX8	INSIDE	Hs.243678	30812	16	976711	976958	248	1	1	2	-0.024540521	0.811537446	no
SPATA2	INSIDE	Hs.48513	9825	20	47955241	47957315	2075	4	6	10	-0.013951063	0.774747746	no
SPIB	INSIDE	Hs.437905	6689	19	55622868	55623780	913	6	5	6	0.039716901	0.584798273	no
SPOCK2	INSIDE	Hs.523009	9806	10	73518139	73518384	246	4	1	2	0.376588175	0.007295447	no
SRD5A2	INSIDE	Hs.458345	6716	2	31658452	31658844	393	3	0	1	0.296180266	0.216910122	no
STC2	INSIDE	Hs.233160	8614	5	172688371	172688586	216	1	0	2	0.149612443	0.404861147	no
STX2	INSIDE	Hs.437585	2054	12	129868979	129869478	500	3	2	5	-0.066934977	0.243419951	no
SUPV3L1	INSIDE	Hs.106469	6832	10	70609920	70610509	590	2	6	4	-0.02239606	0.690309696	no
TBR1	INSIDE	Hs.705400	10716	2	161983608	161983940	333	1	3	3	0.32566226	0.035185818	no
TFDP1	INSIDE	Hs.79353	7027	13	113339555	113340971	1417	7	4	4	-0.012488317	0.923447928	no
TGFB2	INSIDE	Hs.133379	7042	1	216586661	216587189	529	4	4	5	0.218554986	0.050132784	no
TMEM16C	INSIDE	Hs.91791	63982	11	26309962	26310430	469	4	5	5	0.299786454	0.040991646	no
TMEM16F	INSIDE	Hs.696326	196527	12	43896889	43897127	239	1	2	2	-0.001075119	0.9930544	no
TMEM22	INSIDE	Hs.655019	80723	3	138021207	138022368	1162	6	3	7	0.0437725	0.514884892	no
TPBG	INSIDE	Hs.82128	7162	6	83131631	83132118	488	3	4	5	0.121366043	0.299720548	no
TRIM54	INSIDE	Hs.516036	57159	2	27382336	27382910	575	1	4	4	0.180601889	0.018413586	no
TSHZ1	INSIDE	Hs.284217	10194	18	71128785	71129819	1035	1	3	4	-0.068971683	0.432911523	no
TSPAN3	INSIDE	Hs.5062	10099	15	75149799	75150640	842	16	15	6	0.007016421	0.866683477	no
UBE2W	INSIDE	Hs.696026	55284	8	74952880	74953030	151	1	0	1	-0.005543812	0.976166769	no
UBTD2	INSIDE	Hs.179852	92181	5	171642696	171642892	197	0	2	1	0.103750147	0.200634726	no
USP5	INSIDE	Hs.631661	8078	12	6831867	6832175	309	2	4	3	-0.024710984	0.585136572	no
VLDLR	INSIDE	Hs.370422	7436	9	2613032	2613419	388	3	1	4	0.1266073	0.018249217	no
WDR21B	INSIDE	Hs.213307	285429	4	41678788	41679059	272	1	1	3	0.03739746	0.806523384	no
WNK4	INSIDE	Hs.105448	65266	17	38188918	38191403	2486	14	10	14	0.003783965	0.947250708	no
WNT16	INSIDE	Hs.272375	51384	7	120757606	120758191	586	6	1	4	0.238290171	0.058478476	no
WNT6	INSIDE	Hs.29764	7475	2	219443677	219444895	1219	7	9	6	0.035741333	0.516484356	no
WNT7A	INSIDE	Hs.72290	7476	3	13870813	13871741	929	1	5	4	-0.012033686	0.876914731	no
ZC3H7B	INSIDE	Hs.592188	23264	22	40071239	40071959	721	0	1	2	0.018158218	0.838732716	no
ZDHHC19	INSIDE	Hs.111591	131540	3	197418528	197418781	254	7	0	2	0.371058386	0.345964306	no
ZNF274	INSIDE	Hs.83761	10782	19	63407058	63407772	715	6	4	7	0.012991163	0.829825583	no
ZNF569	INSIDE	Hs.511848	148266	19	42649046	42649861	816	4	5	4	0.163570875	0.107509734	no
ZNF701	INSIDE		NA	19	57765660	57766460	801	2	0	3	0.138200316	0.146258691	no
ZNF790	INSIDE	Hs.282067	388536	19	41979933	41980548	616	3	5	3	0.4680619	2.52E-06	no
ZNF790	INSIDE	Hs.282067	388536	19	42020793	42021125	333	5	4	2	0.172205898	0.060714202	no

ZNF813	INSIDE	Hs.705729	126017	19	58663023	58663270	248	4	4	1	0.285715302	0.011004003	no
C9orf102	DOWNSTREAM	Hs.632686	56959	9	97822935	97823411	477	6	5	4	0.088970149	0.083223086	yes
DIO3	DOWNSTREAM	Hs.49322	1735	14	101099597	101099901	305	4	1	3	0.230044537	0.013469917	yes
EOMES	DOWNSTREAM	Hs.591663	8320	3	27729229	27729644	416	2	5	3	0.282787117	0.023019001	yes
FEZF2	DOWNSTREAM	Hs.241523	55079	3	62329308	62329494	187	3	1	1	0.033947234	0.689087908	yes
HAND1	DOWNSTREAM	Hs.152531	9421	5	153833449	153834101	653	2	2	3	0.118213115	0.237881486	yes
ISL1	DOWNSTREAM	Hs.505	3670	5	50730630	50730927	298	1	3	3	0.507372692	0.010225365	yes
MIS12	DOWNSTREAM	Hs.267194	79003	17	5343660	5344265	606	4	10	6	0.050872506	0.185888549	yes
OTP	DOWNSTREAM	Hs.202247	23440	5	76959468	76960331	864	11	8	8	0.039302865	0.258572246	yes
RPRML	DOWNSTREAM	Hs.367999	388394	17	42410273	42410742	470	3	0	4	0.137493983	0.328582045	yes
TCF21	DOWNSTREAM	Hs.78061	6943	6	134258453	134259172	720	3	4	5	-0.022943811	0.686881048	yes
DLL1	DOWNSTREAM	Hs.379912	28514	6	170431172	170431687	516	1	3	5	-0.106142918	0.303081425	no
FLJ39743	DOWNSTREAM	Hs.668070	283777	15	96788601	96789124	524	2	1	2	-0.033174372	0.923284756	no
FLJ43339	DOWNSTREAM		NA	15	38402318	38402907	590	2	1	6	-0.016046934	0.870009377	no
FOXB2	DOWNSTREAM	Hs.553843	442425	9	78827671	78828159	489	5	5	4	0.14560509	0.035022531	no
HAND2	DOWNSTREAM	Hs.388245	9464	4	174679864	174680381	518	6	5	5	0.397970727	0.052089743	no
HLXB9	DOWNSTREAM		NA	7	156489949	156490698	750	5	2	8	0.050692873	0.167020598	no
MEIS1	DOWNSTREAM	Hs.526754	4211	2	66657397	66657632	236	1	2	2	0.165973331	0.089586118	no
MSX1	DOWNSTREAM	Hs.424414	4487	4	4918035	4919227	1193	11	12	3	0.178865807	0.005619856	no
NGRN	DOWNSTREAM	Hs.513145	51335	15	88619119	88620107	989	7	3	3	0.054184275	0.571870567	no
NKX6-1	DOWNSTREAM	Hs.546270	4825	4	85633298	85633772	475	5	6	4	0.1493351	0.006370553	no
NOC4L	DOWNSTREAM	Hs.558536	79050	12	131205116	131205669	554	2	7	2	0.007677283	0.974305972	no
ODZ3	DOWNSTREAM	Hs.130438	55714	4	183964869	183965098	230	2	1	1	0.036796509	0.786337144	no
SLC1A3	DOWNSTREAM	Hs.481918	6507	5	36725745	36726530	786	5	5	7	0.127797423	0.004771909	no
SPHK2	DOWNSTREAM		NA	19	53825022	53825661	640	4	6	5	-0.022964777	0.822905897	no
ZFYVE21	DOWNSTREAM	Hs.592322	79038	14	103274572	103275046	475	1	2	3	0.047129868	0.730928911	no
chr10:042568840-042568884	Unknown		NA	10	42568496	42568974	479	5	4	3	0.066834757	0.174988448	yes
chr10:101271888-101271932	Unknown		NA	10	101271803	101272056	254	3	3	2	0.071850267	0.700838912	yes
chr10:112392988-112393032	Unknown		NA	10	112392444	112393116	673	2	3	3	0.27607581	0.025126028	yes
chr11:043921791-043921843	Unknown		NA	11	43921641	43921932	292	3	0	2	0.0653407	0.616988704	yes
chr12:052430762-052430821	Unknown		NA	12	52430621	52431552	932	7	4	7	0.022163993	0.755204633	yes
chr13:049600069-049600117	Unknown		NA	13	49599878	49600295	418	5	5	5	0.230067038	0.032452193	yes
chr13:107946642-107946686	Unknown		NA	13	107945549	107946973	1425	24	17	12	0.044018335	0.278706424	yes
chr14:036044633-036044677	Unknown		NA	14	36044320	36044736	417	6	2	5	0.027019048	0.659152356	yes
chr14:036186039-036186083	Unknown		NA	14	36185611	36186264	654	3	9	5	0.132916247	0.121892558	yes
chr14:036187223-036187267	Unknown		NA	14	36186265	36187651	1387	11	12	15	0.040106748	0.437806878	yes
chr15:032594005-032594049	Unknown		NA	15	32593763	32595035	1273	13	10	6	0.046055502	0.412383181	yes
chr15:087743702-087743746	Unknown		NA	15	87743554	87743881	328	3	7	3	0.171412329	0.090003828	yes
chr15:094753880-094753935	Unknown		NA	15	94753776	94754391	616	5	6	2	0.061999943	0.501528453	yes
chr17:040454837-040454881	Unknown		NA	17	40452167	40455156	2990	16	19	10	0.012996348	0.765269525	yes
chr18:005186914-005186958	Unknown		NA	18	5186754	5187081	328	2	0	3	0.077822085	0.634234974	yes
chr18:022489587-022489632	Unknown		NA	18	22489365	22489862	498	2	0	3	0.168117624	0.110641573	yes
chr18:053622261-053622309	Unknown		NA	18	53622175	53622377	203	1	0	1	-0.047061481	0.90337791	yes
chr20:021034956-021035000	Unknown		NA	20	21034718	21035064	347	2	2	3	0.181772632	0.006370553	yes
chr5:050300996-050301040	Unknown		NA	5	50300825	50301334	510	4	7	4	0.093167152	0.137410809	yes
chr5:134852878-134852922	Unknown		NA	5	134852638	134852960	323	4	1	2	0.091821791	0.673602531	yes
chr6:017210372-017210416	Unknown		NA	6	17210305	17210670	366	9	3	4	0.148122915	0.370553539	yes
chr6:026721885-026721929	Unknown		NA	6	26721292	26721962	671	2	2	1	0.338459958	0.003613506	yes
chr6:037611967-037612024	Unknown		NA	6	37611477	37612328	852	5	7	8	0.09511397	0.045121361	yes
chr8:023640269-023640313	Unknown		NA	8	23640009	23640799	791	9	15	7	0.026075312	0.488307452	yes
chr8:030889223-030889267	Unknown		NA	8	30888710	30889759	1050	18	7	9	0.071629296	0.088352939	yes
chr9:014338970-014339026	Unknown		NA	9	14338903	14339130	228	0	1	1	0.240337293	0.025670146	yes
chr1:014091848-014091893	Unknown		NA	1	14091321	14092981	1661	11	14	5	-0.01517028	0.760590647	no
chr1:016720487-016720533	Unknown		NA	1	16719703	16720852	1150	4	5	2	0.28601952	0.000533244	no
chr1:022484852-022484896	Unknown		NA	1	22483887	22485240	1354	4	9	3	-0.136014968	0.003016494	no
chr1:046686821-046686865	Unknown		NA	1	46686615	46687623	1009	2	8	4	0.013886779	0.82570946	no
chr1:050666116-050666160	Unknown		NA	1	50665352	50666749	1398	5	12	9	0.059863097	0.144086402	no
chr1:087390563-087390613	Unknown		NA	1	87390235	87390709	475	1	3	3	0.137982153	0.417824881	no
chr1:088700757-088700816	Unknown		NA	1	88700684	88700847	164	1	1	1	0.173983958	0.209934397	no
chr1:110428880-110428924	Unknown		NA	1	110428020	110429189	1170	13	8	11	0.060551227	0.199154513	no
chr1:113088612-113088656	Unknown		NA	1	113088480	113088868	389	5	5	3	0.193338299	0.103919045	no
chr1:143751455-143751499	Unknown		NA	1	143750738	143751750	1013	6	4	7	0.089002029	0.220061858	no
chr1:150348046-150348090	Unknown		NA	1	150346486	150348136	1651	15	4	7	0.138202158	0.145841214	no
chr10:031114438-031114484	Unknown		NA	10	31114112	31114848	737	4	3	4	0.178065851	0.01004373	no
chr10:045039700-045039744	Unknown		NA	10	45039635	45039941	307	1	4	2	-0.09994707	0.274617494	no
chr10:075160143-075160187	Unknown		NA	10	75159526	75160737	1212	5	14	2	0.043614054	0.424377308	no
chr10:079733603-079733662	Unknown		NA	10	79733422	79733885	464	1	0	3	-0.004602537	0.987226522	no
chr10:121761403-121761447	Unknown		NA	10	121761262	121761496	235	0	3	2	0.072453953	0.65255068	no
chr10:129837631-129837675	Unknown		NA	10	129837617	129837901	285	2	3	3	-0.001277963	0.987502264	no
chr10:129838964-129839008	Unknown		NA	10	129838869	129839152	284	2	2	2	0.082565735	0.37086326	no
chr10:134657347-134657391	Unknown		NA	10	134657272	134658090	819	3	3	2	-0.053571074	0.674163809	no
chr11:001014727-001014771	Unknown		NA	11	1014647	1015468	822	1	8	7	0.047605063	0.591616534	no
chr11:001315593-001315637	Unknown		NA	11	1315431	1316484	1054	5	5	3	-0.047258306	0.410199045	no
chr11:001316868-001316913	Unknown		NA	11	1316485	1317157	673	1	6	6	-0.014999001	0.85316734	no
chr11:065003020-065003064	Unknown		NA	11	65002543	65003357	815	3	4	5	0.014051943	0.832770641	no
chr11:122806352-122806396	Unknown		NA	11	122805791	122807533	1743	10	14	12	0.02955888	0.298054365	no
chr11:122953374-122953418	Unknown		NA	11	122952948	122953668	721	0	7	5	-0.119583671	0.001161347	no
chr12:006036126-006036170	Unknown		NA	12	6035906	6036955	1050	8	5	5	-0.014790872	0.694822444	no
chr12:097375047-097375091	Unknown		NA	12	97374428	97375167	740	4	8	2	0.037163582	0.742004492	no
chr12:125241924-125241968	Unknown		NA	12	125241137	125242835	1699	8	10	9	-0.007415872	0.895083583	no
chr13:111010030-111010074	Unknown		NA	13	111009787	111010204	418	1	2	1	-0.13197147	0.05729941	no
chr13:111678587-111678631	Unknown		NA	13	111678527	111678817	291	5	4	2	0.017732632	0.8267616	no
chr13:113944864-113944908	Unknown		NA	13	113944829	113945843	1015	5	5	5	0.012727768	0.913922643	no
chr14:088564061-088564114	Unknown		NA	14	88563932	88564196	265	3	1	2	0.135844642	0.255926963	no
chr14:099714009-099714053	Unknown		NA	14	99713874	99714352	479	5	4	4	-0.003512942	0.954145741	no
chr15:029476816-029476860	Unknown		NA	15	29476473	29477105	633	1	2	2	-0.022794836	0.841454697	no
chr15:050883720-050883768	Unknown		NA	15	50883452	50884000	549	2	3	3	0.109158976	0.202534751	no
chr15:096908995-096909044	Unknown		NA	15	96908392	96909771	1380	9	9	8	0.019471208	0.708284962	no
chr16:085807639-085807683	Unknown		NA	16	85807533	85808184	652	3	7	6	-0.015372456	0.906706404	no
chr16:087485836-087485882	Unknown		NA	16	87485738	87486098	361	0	1	3	-0.019353368	0.908533573	no
chr16:087503305-087503349	Unknown		NA	16	87503220	87503516	297	1	4	1	-0.022858155	0.915507746	no
chr17:000655354-000655399	Unknown		NA	17	655088	655567	480	3	3	3	-0.094456422	0.288054842	no
chr17:052478530-052478574	Unknown		NA	17	52477740	52479114	1375	7	14	11	-0.017214203	0.734000785	no
chr18:005227613-005227672	Unknown		NA	18	5227337	5227693	357	0	2	1	0.047711785	0.719458315	no

chr18:035675460-035675504	Unknown	NA	18	35674858	35676043	1186	3	3	7	0.01491184	0.893247121	no
chr18:070134562-070134606	Unknown	NA	18	70134526	70134916	391	1	3	3	-0.113142747	0.054774067	no
chr18:074718149-074718193	Unknown	NA	18	74718048	74718258	211	2	2	2	-0.063842586	0.61132851	no
chr18:075414911-075414955	Unknown	NA	18	75414129	75415004	876	4	5	3	-0.20621175	0.001218079	no
chr18:075415121-075415171	Unknown	NA	18	75415005	75415501	497	0	2	1	-0.17055299	0.190993415	no
chr18:075437150-075437194	Unknown	NA	18	75436846	75437286	441	1	2	3	-0.010186777	0.962513718	no
chr18:075496257-075496301	Unknown	NA	18	75496225	75496665	441	2	1	5	-0.089369075	0.262727835	no
chr18:075668344-075668390	Unknown	NA	18	75667996	75668581	586	3	2	3	-0.217540359	0.005777556	no
chr19:001477512-001477556	Unknown	NA	19	1477126	1477715	590	3	3	3	-0.044928757	0.625450909	no
chr19:002462783-002462828	Unknown	NA	19	2462656	2463107	452	0	2	2	-0.130217372	0.261558617	no
chr19:002839832-002839876	Unknown	NA	19	2839620	2839894	275	1	1	1	0.00775765	0.929759623	no
chr19:010388063-010388107	Unknown	NA	19	10387882	10388157	276	1	1	2	0.077664548	0.415085135	no
chr2:007824903-007824947	Unknown	NA	2	7824369	7825219	851	3	5	6	-0.244995199	0.008080187	no
chr2:073282865-073282909	Unknown	NA	2	73282770	73283040	271	2	2	3	0.271944153	0.03066704	no
chr2:095556308-095556352	Unknown	NA	2	95555634	95556928	1295	12	9	2	0.100020831	0.070064633	no
chr2:106325502-106325546	Unknown	NA	2	106325285	106325990	706	2	3	6	-0.063252119	0.261280408	no
chr2:127251272-127251316	Unknown	NA	2	127251111	127251529	419	2	4	3	0.08624916	0.400598541	no
chr2:128149767-128149811	Unknown	NA	2	128149601	128149972	372	3	6	3	0.051309638	0.440681351	no
chr2:225614863-225614907	Unknown	NA	2	225614706	225614926	221	2	3	1	0.186312796	0.103392824	no
chr2:236752750-236752809	Unknown	NA	2	236752579	236752880	302	2	2	2	0.123616525	0.208550808	no
chr2:241235802-241235846	Unknown	NA	2	241235618	241236201	584	2	2	3	0.04608075	0.709450017	no
chr20:045301523-045301573	Unknown	NA	20	45300750	45301658	909	3	8	6	0.064271906	0.330479644	no
chr22:016229574-016229618	Unknown	NA	22	16229234	16229865	632	1	4	6	0.134633641	0.003166562	no
chr22:016229953-016230000	Unknown	NA	22	16229866	16230028	163	1	1	1	0.411181986	0.026354093	no
chr22:030388357-030388401	Unknown	NA	22	30388249	30388571	323	1	0	1	-0.119369182	0.611468158	no
chr22:044420612-044420666	Unknown	NA	22	44420549	44421005	457	1	2	1	-0.027005768	0.915367682	no
chr3:030911245-030911289	Unknown	NA	3	30911039	30911332	294	6	2	3	0.329645109	0.068793587	no
chr3:042996134-042996178	Unknown	NA	3	42995380	42996513	1134	12	11	10	0.004033727	0.961418783	no
chr3:044015118-044015162	Unknown	NA	3	44014975	44015305	331	1	3	3	0.129043929	0.008044182	no
chr3:128336746-128336790	Unknown	NA	3	128335307	128337062	1756	6	7	3	0.018562377	0.817731482	no
chr4:001595345-001595389	Unknown	NA	4	1594249	1595496	1248	3	11	4	-0.004493919	0.938643103	no
chr4:003712180-003712236	Unknown	NA	4	3712165	3712634	470	0	1	1	-0.255654763	0.003950536	no
chr4:008398657-008398701	Unknown	NA	4	8398528	8399355	828	3	4	2	-0.261246521	0.01998124	no
chr4:008696800-008696844	Unknown	NA	4	8696519	8697356	838	0	4	5	-0.15275439	0.003748493	no
chr4:014473804-014473848	Unknown	NA	4	14473494	14473913	420	5	4	4	0.018428469	0.782607186	no
chr4:015971628-015971672	Unknown	NA	4	15971334	15971923	590	3	2	2	-0.032675058	0.801570092	no
chr4:017391744-017391788	Unknown	NA	4	17391498	17391992	495	0	7	4	0.082492093	0.412120609	no
chr4:123090967-123091011	Unknown	NA	4	123090778	123091193	416	3	5	4	0.075218155	0.356918347	no
chr4:128763530-128763574	Unknown	NA	4	128763322	128764381	1060	10	12	10	0.062730798	0.161492927	no
chr4:141638636-141638680	Unknown	NA	4	141638070	141639039	970	12	4	8	0.133755357	0.028855222	no
chr4:183303382-183303426	Unknown	NA	4	183303306	183303711	406	0	2	1	0.181428704	0.199746139	no
chr4:184641504-184641548	Unknown	NA	4	184640183	184642493	2311	7	13	1	-0.052489487	0.572926462	no
chr4:186287064-186287108	Unknown	NA	4	186286993	186287190	198	3	0	2	0.083958685	0.404944187	no
chr4:186287218-186287268	Unknown	NA	4	186287191	186287428	238	1	0	1	0.128207534	0.441984509	no
chr4:187262662-187262706	Unknown	NA	4	187262425	187262781	357	1	1	1	-0.063997149	0.52757832	no
chr5:001698180-001698224	Unknown	NA	5	1697516	1698285	770	4	0	2	-0.03870884	0.861897627	no
chr5:087934449-087934496	Unknown	NA	5	87934320	87934537	218	0	1	1	-0.239846413	0.070077485	no
chr5:143958646-143958690	Unknown	NA	5	143958516	143958814	299	1	3	3	0.052131153	0.614630144	no
chr5:172001705-172001755	Unknown	NA	5	172001698	172002004	307	3	0	1	0.065772746	0.776887642	no
chr5:176102972-176103016	Unknown	NA	5	176102424	176103332	909	3	8	6	0.039575352	0.332899592	no
chr6:006491870-006491915	Unknown	NA	6	6491471	6492265	795	7	8	8	0.10208867	0.107950164	no
chr6:066861276-066861320	Unknown	NA	6	66861109	66861566	458	2	6	1	-0.175223074	0.003700594	no
chr6:072186165-072186219	Unknown	NA	6	72185999	72186410	412	4	1	2	0.301859959	9.32E-06	no
chr6:146961681-146961725	Unknown	NA	6	146961590	146962285	696	3	1	4	0.111454723	0.080133578	no
chr6:166587587-166587631	Unknown	NA	6	166587396	166587701	306	2	5	2	0.046034414	0.39818327	no
chr6:168373153-168373197	Unknown	NA	6	168372287	168373316	1030	0	12	3	0.027978774	0.766336505	no
chr7:001608369-001608413	Unknown	NA	7	1608185	1608550	366	0	5	4	-0.069334981	0.423314083	no
chr7:001610038-001610082	Unknown	NA	7	1608886	1610400	1515	2	11	4	0.012130543	0.86331032	no
chr7:005430482-005430526	Unknown	NA	7	5430442	5430643	202	1	5	1	-0.01937285	0.825578449	no
chr7:029655539-029655583	Unknown	NA	7	29655385	29657238	1854	8	12	4	0.015306621	0.849195455	no
chr7:054924140-054924199	Unknown	NA	7	54923952	54924222	271	1	0	3	0.181142694	0.136885305	no
chr7:065515926-065515970	Unknown	NA	7	65515702	65516505	804	6	3	5	0.059443412	0.325658304	no
chr7:084407178-084407222	Unknown	NA	7	84406769	84407535	767	4	6	3	-0.060161863	0.378844406	no
chr7:111155850-111155895	Unknown	NA	7	111155479	111156148	670	4	8	6	-0.072374306	0.345161873	no
chr7:113512986-113513033	Unknown	NA	7	113512868	113513251	384	1	0	4	0.02214255	0.933963497	no
chr7:127531799-127531843	Unknown	NA	7	127531753	127532175	423	2	5	1	0.122217844	0.060668971	no
chr7:151695026-151695070	Unknown	NA	7	151694746	151695564	819	3	7	3	0.005651829	0.966879801	no
chr7:154857394-154857438	Unknown	NA	7	154856845	154857593	749	2	1	4	0.14309357	0.004475093	no
chr7:157630344-157630388	Unknown	NA	7	157630258	157631228	971	5	1	1	0.027594044	0.742732706	no
chr8:001959623-001959671	Unknown	NA	8	1958608	1959870	1263	5	5	7	-0.046270152	0.260897308	no
chr8:024855808-024855852	Unknown	NA	8	24855659	24856134	476	3	3	5	0.137544879	0.105845829	no
chr8:027792950-027792994	Unknown	NA	8	27792644	27793386	743	3	3	4	-0.092675137	0.240844919	no
chr8:142840103-142840147	Unknown	NA	8	142839474	142840280	807	3	4	5	-0.172837424	0.002418163	no
chr8:143330884-143330928	Unknown	NA	8	143330360	143331468	1109	7	14	1	-0.090576116	0.481575808	no
chr9:008847976-008848020	Unknown	NA	9	8847754	8848226	473	6	2	2	0.14170227	0.356485568	no
chr9:008848430-008848474	Unknown	NA	9	8848227	8848497	271	1	2	4	0.110852762	0.278875364	no
chr9:014336965-014337011	Unknown	NA	9	14336715	14337149	435	3	2	4	0.388442763	0.036860924	no
chr9:044167268-044167316	Unknown	NA	9	44167109	44167618	510	3	5	1	0.491029759	0.038857513	no
chr9:073251288-073251332	Unknown	NA	9	73251098	73251442	345	1	2	2	0.421884953	0.004182722	no
chr9:092919954-092919998	Unknown	NA	9	92919465	92920297	833	4	7	9	0.020163677	0.797691977	no
chr9:093483951-093483995	Unknown	NA	9	93483907	93484278	372	1	2	2	0.049265003	0.516411264	no
chr9:099605605-099605649	Unknown	NA	9	99605421	99606253	833	9	6	4	0.00980726	0.887202365	no
chr9:106770451-106770495	Unknown	NA	9	106770385	106770586	202	1	2	2	0.127222872	0.298462295	no
chr9:106770713-106770757	Unknown	NA	9	106770587	106770787	201	2	1	2	0.262810632	0.026972148	no
chr9:123699042-123699088	Unknown	NA	9	123698489	123699112	624	2	1	6	-0.050662672	0.674820615	no
chr9:133707322-133707374	Unknown	NA	9	133707169	133707431	263	3	1	2	-0.177009988	0.149150531	no
chr9:139521172-139521218	Unknown	NA	9	139520569	139521485	917	1	2	5	-0.148066227	0.022033348	no
chrX:039759494-039759538	Unknown	NA	X	39759406	39759607	202	1	1	1	0.039558395	0.807258235	no
chrY:000294404-000294447	Unknown	NA	Y	294283	295336	1054	3	9	2	0.098659716	0.146715604	no
chrY:013159340-013159384	Unknown	NA	Y	13158389	13160259	1871	9	2	8	0.011376753	0.827737708	no
chrY:024760938-024760981	Unknown	NA	Y	24760692	24761456	765	7	3	8	0.076357885	0.481568559	no
chrY:026019522-026019565	Unknown	NA	Y	26019235	26019749	515	2	2	2	0.402196482	0.013413406	no
