# Supplementary material for: BRAF mutation-specific promoter methylation of FOX genes in colorectal cancer
Source: Clin Epigenetics. 2013 Jan 16;5(1):2. doi: 10.1186/1868-7083-5-2 (PMC3599401; doi:10.1186/1868-7083-5-2)
Supplement: Additional file 3 — Sample information. [file 1868-7083-5-2-S3.pdf]

Additional File 3 - Sample information

Patient ID	Gender	Age	Histology	*MLH1* methylation	*CIMP* status	*BRAF* V600E
117	M	69	Ad	U	-	wt
180	F	86	Ad	U	-	wt
118	F	69	Ad	U	-	wt
127	F	87	Ad	U	-	wt
577	M	32	Ad	U	-	wt
187	F	61	Ca	U	-	wt
503	F	63	Ca	U	-	wt
97	F	68	Ca	U	-	wt
131	F	71	Ca	U	+	mut
505	F	83	Ca	U	+	wt
63	F	57	Ca	U	+	mut
57	F	75	Ca	pM	+	mut
601	F	83	Ad	M	+	wt
56	M	75	Ca	M	+	mut
499	F	69	Ca	M	+	mut
119	F	76	Ca	M	+	wt
49	F	80	Ca	M	+	mut
179	M	90	Ca	M	+	mut
132	F	64	Ca	M	+	mut
