# Supplementary material for: BRAF mutation-specific promoter methylation of FOX genes in colorectal cancer
Source: Clin Epigenetics. 2013 Jan 16;5(1):2. doi: 10.1186/1868-7083-5-2 (PMC3599401; doi:10.1186/1868-7083-5-2)
Supplement: Additional file 4 — Regions with BRAF mutation-specific methylation changes (UCSC assembly: March 2006, NCBI36/hg18). [file 1868-7083-5-2-S4.pdf]

Gene Name	Description	UniGene	Entrez Gene	Chrom	Start	End	Length	BstUI sites	HpaII sites	Probes	logFC	adj.P.Val	H3K27me3 bound
FLJ45983	DIVERGENT_PROMOTER	Hs.669736	399717	10	8135262	8136154	893	5	7	10	0.117947035	0.007175996	yes
hsa-mir-212-HIC1	DIVERGENT_PROMOTER		NA	17	1902162	1903130	969	0	2	2	0.245324707	0.001130901	yes
C1orf147-RASSF5	DIVERGENT_PROMOTER		NA	1	204746527	204747456	930	7	6	7	0.154261983	0.001410244	no
DDIT3-MBD6	DIVERGENT_PROMOTER		NA	12	56200553	56201169	617	5	4	5	-0.093515673	0.008402427	no
EVX2-HOXD13	DIVERGENT_PROMOTER		NA	2	176658724	176658898	175	1	0	1	0.273733053	0.003787918	no
FLJ45983-GATA3	DIVERGENT_PROMOTER		NA	10	8136361	8136628	268	4	4	1	0.172599148	0.002725499	no
GCN5L2-HSPB9	DIVERGENT_PROMOTER		NA	17	37527850	37528126	277	2	2	2	0.23793362	0.004819314	no
HIST1H2BB-HIST1H3C	DIVERGENT_PROMOTER		NA	6	26151974	26152986	1013	3	3	4	0.258348193	0.001822638	no
LOC116143	DIVERGENT_PROMOTER		NA	2	68237734	68238556	823	2	7	8	-0.106301126	0.001148009	no
MLH1	DIVERGENT_PROMOTER	Hs.195364	4292	3	37009074	37010186	1113	6	5	12	0.383242205	0.001467322	no
RGAG4-LOC340527	DIVERGENT_PROMOTER		NA	X	71268524	71268920	397	2	1	4	0.134387235	0.009335554	no
SATB2-FLJ32063	DIVERGENT_PROMOTER		NA	2	200041873	200042163	291	2	3	2	0.203772408	0.005069892	no
TAF7	DIVERGENT_PROMOTER	Hs.438838	6879	5	140680375	140681143	769	4	8	4	0.234437188	0.008608134	no
ZNF2	DIVERGENT_PROMOTER	Hs.590916	7549	2	95194775	95195174	400	1	1	4	0.142077864	0.007495946	no
ZNF569	DIVERGENT_PROMOTER	Hs.511848	148266	19	42649862	42650417	556	6	5	5	0.486279505	0.006454321	no
ARPP-21	PROMOTER	Hs.475902	10777	3	35655639	35656170	532	4	11	4	0.204189863	0.001467322	yes
BBS5	PROMOTER	Hs.233398	129880	2	170043599	170044144	546	0	1	1	0.357246564	0.0073902	yes
BDNF	PROMOTER	Hs.502182	627	11	27700175	27700615	441	1	0	4	0.387391403	0.003880277	yes
BMP6	PROMOTER	Hs.285671	654	6	7671036	7671225	190	2	1	1	0.48894727	0.000953447	yes
C20orf103	PROMOTER	Hs.22920	24141	20	9443102	9443665	564	2	3	4	0.197712526	0.009218599	yes
CIDEA	PROMOTER	Hs.249129	1149	18	12244331	12245122	792	12	5	9	0.225508502	0.001257025	yes
COCH	PROMOTER	Hs.21016	1690	14	30412172	30414210	2039	18	17	14	0.088545656	0.001830022	yes
COL12A1	PROMOTER	Hs.101302	1303	6	75971995	75972515	521	5	4	5	0.339482751	0.003398448	yes
COL12A1	PROMOTER	Hs.101302	1303	6	75972516	75972803	288	1	2	2	0.456230175	0.008625148	yes
CYP26A1	PROMOTER	Hs.150595	1592	10	94817946	94819100	1155	10	6	12	0.190207537	0.001578885	yes
DBX2	PROMOTER	Hs.302764	440097	12	43731177	43731817	641	4	2	6	0.188207525	0.006875425	yes
DMRT3	PROMOTER	Hs.189174	58524	9	959793	960443	651	4	4	5	0.156615208	0.003721962	yes
EBF1	PROMOTER	Hs.657753	1879	5	158465569	158466334	766	2	1	3	0.241042437	0.000788399	yes
EBF1	PROMOTER	Hs.657753	1879	5	158464782	158465253	472	4	4	5	0.288455213	0.002400653	yes
EBF1	PROMOTER	Hs.657753	1879	5	158460071	158460759	689	5	5	6	0.148389118	0.007919239	yes
EBF2	PROMOTER	Hs.491292	64641	8	25964811	25965597	787	6	0	4	0.316134783	0.003495931	yes
EMX2	PROMOTER	Hs.202095	2018	10	119287464	119287753	290	3	1	2	0.20247192	0.005576168	yes
FAM62C	PROMOTER	Hs.477711	83850	3	139635427	139635930	504	2	4	1	0.480765238	0.003586334	yes
FLI1	PROMOTER	Hs.504281	2313	11	128068668	128069169	502	2	2	3	0.154769646	0.009446829	yes
FLT3	PROMOTER	Hs.507590	2322	13	27572738	27573180	443	4	4	4	0.268702417	0.000231399	yes
FOXA1	PROMOTER	Hs.163484	3169	14	37136950	37137252	303	1	1	1	0.53114072	0.006694417	yes
FOXC1	PROMOTER	Hs.348883	2296	6	1551209	1551628	420	1	2	4	0.447293876	0.001541679	yes
FOXC1	PROMOTER	Hs.348883	2296	6	1552611	1553143	533	1	0	7	0.237592782	0.00225147	yes
FOXC1	PROMOTER	Hs.348883	2296	6	1553720	1554106	387	1	3	3	0.399973683	0.004160655	yes
FOXC1	PROMOTER	Hs.348883	2296	6	1553532	1553683	152	0	2	1	0.166440795	0.005343603	yes
FOXF1	PROMOTER	Hs.155591	2294	16	85100331	85100986	656	10	12	6	0.175209414	0.000296803	yes
GAS2	PROMOTER		NA	11	22645148	22645365	218	1	1	1	0.457068661	0.005012074	yes
GRIK2	PROMOTER	Hs.98262	2898	6	101947561	101948205	645	2	1	1	0.387419301	0.004418351	yes
HHEX	PROMOTER	Hs.118651	3087	10	94438652	94439164	513	2	5	5	0.189411727	0.002661375	yes
HMX2	PROMOTER	Hs.444756	3167	10	124892259	124893350	1092	4	8	5	0.161586839	0.000969397	yes
HOXC11	PROMOTER	Hs.127562	3227	12	52645223	52646112	890	1	4	2	0.374564985	0.001231975	yes
HOXC8	PROMOTER	Hs.664500	3224	12	52685578	52685945	368	7	0	3	0.904588283	0.001210989	yes
HS3ST3B1	PROMOTER	Hs.48384	9953	17	14143173	14144047	875	2	9	8	0.136777944	0.000762312	yes
ISLR2	PROMOTER	Hs.254775	57611	15	72209856	72210394	539	5	1	3	0.588109669	5.03E-06	yes
LBXCOR1	PROMOTER	Hs.451224	390598	15	65900334	65900788	455	3	5	3	0.481555902	0.001354784	yes
LHX1	PROMOTER	Hs.443727	3975	17	32368397	32369050	654	7	2	7	0.165942618	0.008373072	yes
LOC643923	PROMOTER	Hs.647220	643923	11	106967578	106967989	412	2	4	5	0.218280528	0.005014718	yes
MKX	PROMOTER	Hs.128193	283078	10	28074434	28074945	512	9	9	5	0.303383815	0.005014718	yes
NFATC1	PROMOTER	Hs.534074	4772	18	75259296	75259759	464	1	0	4	0.426715885	0.000743027	yes
NFATC1	PROMOTER	Hs.534074	4772	18	75258657	75259295	639	1	6	6	0.375198889	0.000872582	yes
NKX2-3	PROMOTER	Hs.243272	159296	10	101282054	101282866	813	4	6	6	0.16583427	0.002700841	yes
NPR3	PROMOTER	Hs.237028	4883	5	32745873	32746812	940	8	5	9	0.197323469	0.001527366	yes
NR2E1	PROMOTER	Hs.157688	7101	6	108585659	108586260	602	7	7	5	0.338210409	0.003273772	yes
NR2F1	PROMOTER	Hs.519445	7025	5	92941349	92941573	225	4	5	2	0.428919471	0.000296467	yes
NR2F1	PROMOTER	Hs.519445	7025	5	92941574	92942225	652	3	3	5	0.129674544	0.005271963	yes
NRXN3	PROMOTER	Hs.368307	9369	14	78814935	78815789	855	9	6	9	0.220570228	0.001715396	yes
ONECUT1	PROMOTER	Hs.658573	3175	15	50870652	50871543	892	2	8	4	-0.299386793	0.000363682	yes
PAX6	PROMOTER	Hs.591993	5080	11	31795501	31795809	309	3	1	3	0.432781032	0.00033289	yes
PAX6	PROMOTER	Hs.591993	5080	11	31790734	31791451	718	5	12	8	0.110005814	0.005363106	yes
PAX9	PROMOTER	Hs.132576	5083	14	36196266	36196495	230	2	1	1	0.417215803	0.002549979	yes
PLEC1	PROMOTER	Hs.434248	5339	8	145121092	145121609	518	4	3	4	0.103127091	0.000755458	yes
POU3F2	PROMOTER	Hs.182505	5454	6	99386844	99388638	1795	20	9	18	0.156044854	9.41E-05	yes
POU4F1	PROMOTER	Hs.654522	5457	13	78081800	78082006	207	0	2	2	0.185191057	0.006339341	yes
PRDM13	PROMOTER	Hs.287386	59336	6	100158334	100158661	328	2	4	3	0.405084652	0.000446388	yes
PRICKLE1	PROMOTER	Hs.524348	144165	12	41270336	41270614	279	2	3	2	0.574795172	0.000111698	yes
PRICKLE1	PROMOTER	Hs.524348	144165	12	41270615	41270891	277	0	1	1	0.588527431	0.000909204	yes
RAB6B	PROMOTER		NA	3	135097652	135098306	655	2	1	3	0.277425003	0.000200764	yes
RGMA	PROMOTER	Hs.271277	56963	15	91433940	91434218	279	4	0	2	0.235553952	0.00054177	yes
SCN4B	PROMOTER	Hs.65239	6330	11	117529253	117529999	747	2	4	2	0.173665223	0.004805681	yes
SH3GL2	PROMOTER	Hs.75149	6456	9	17569037	17569857	821	15	12	8	0.09495963	0.004590488	yes
SHOX2	PROMOTER	Hs.55967	6474	3	159305290	159305717	428	0	2	1	0.172122877	0.001541679	yes
SIX1	PROMOTER	Hs.633506	6495	14	60185975	60186328	354	6	5	2	0.200940388	0.00173212	yes
SIX2	PROMOTER	Hs.101937	10736	2	45090095	45090516	422	3	7	2	0.217389136	0.005247877	yes
SLC1A2	PROMOTER	Hs.502338	6506	11	35397707	35398204	498	5	4	5	0.123550609	0.002980322	yes
SLC26A10	PROMOTER	Hs.159481	65012	12	56299262	56299549	288	1	2	2	0.450671342	0.000589411	yes
SLC26A10	PROMOTER	Hs.159481	65012	12	56299642	56300252	611	3	7	3	0.17178857	0.003340558	yes
SLC6A4	PROMOTER	Hs.591192	6532	17	25587172	25587490	319	1	1	3	0.260126297	0.000794408	yes
SOX1	PROMOTER	Hs.202526	6656	13	111759930	111760494	565	5	3	6	0.114820099	0.005346729	yes
TBX2	PROMOTER	Hs.705451	6909	17	56828611	56828930	320	2	6	2	0.325440325	0.001912032	yes
TITF1	PROMOTER		NA	14	36063789	36064191	403	2	0	4	0.28805712	0.002917629	yes
TITF1	PROMOTER		NA	14	36060201	36060432	232	2	3	2	0.174761784	0.003211258	yes
TITF1	PROMOTER		NA	14	36059857	36060200	344	4	3	3	0.454421864	0.003805037	yes
TLX3	PROMOTER	Hs.249125	30012	5	170668670	170669045	376	3	2	4	0.271028366	0.003844533	yes
TNFRSF19	PROMOTER	Hs.149168	55504	13	23050867	23051593	727	7	9	7	0.123519983	0.009335554	yes

TPH1	PROMOTER	Hs.591999	7166	11	18023916	18024558	643	4	4	3	0.161216391	0.007748335	yes
ZNF781	PROMOTER	Hs.631565	163115	19	42874452	42875284	833	7	4	7	0.166242619	0.003800041	yes
ABCA5	PROMOTER	Hs.421474	23461	17	64835314	64835687	374	3	0	2	0.243897477	0.005343603	no
ACADL	PROMOTER	Hs.471277	33	2	210797548	210798631	1084	14	12	10	0.09146752	0.002716778	no
ACP5	PROMOTER	Hs.1211	54	19	11547845	11550737	2893	7	12	3	0.138381331	0.00792501	no
AGXT2L1	PROMOTER	Hs.106576	64850	4	109903684	109904117	434	2	5	2	0.513353322	1.35E-05	no
ANK1	PROMOTER	Hs.654438	286	8	41775011	41775393	383	1	0	2	0.143889567	0.007063241	no
ANKDD1A	PROMOTER	Hs.207157	348094	15	62990747	62990991	245	2	3	2	0.235404043	0.004956232	no
ARL4A	PROMOTER	Hs.245540	10124	7	12692448	12692655	208	1	1	1	0.218264334	1.66E-05	no
ATRNL1	PROMOTER	Hs.501127	26033	10	116841743	116842468	726	5	0	4	0.239047492	0.001482741	no
AXIN1	PROMOTER	Hs.592082	8312	16	351056	351793	738	2	6	5	0.24174537	0.000265564	no
B4GALNT3	PROMOTER	Hs.504416	283358	12	432987	433255	269	0	2	2	0.27825649	0.004336924	no
BARHL2	PROMOTER	Hs.451956	343472	1	90963768	90964279	512	2	3	5	0.283580525	0.00358025	no
BARX1	PROMOTER	Hs.164960	56033	9	95761255	95761562	308	6	3	3	0.140494567	0.005747581	no
BCOR	PROMOTER	Hs.659681	54880	X	39848176	39848483	308	2	0	3	0.476473112	0.00807921	no
BMPER	PROMOTER	Hs.660998	168667	7	33910103	33910655	553	2	3	5	0.325734171	0.005734996	no
C1orf176	PROMOTER	Hs.59584	64789	1	40746546	40746807	262	1	4	3	0.269538664	0.009890391	no
C22orf27	PROMOTER	Hs.592202	150291	22	29647864	29648406	543	3	0	2	0.237130483	0.005747581	no
C2orf52	PROMOTER	Hs.375211	151477	2	232087184	232087703	520	12	1	6	0.197719373	0.009198677	no
CAMK2N2	PROMOTER	Hs.585003	94032	3	185462726	185463338	613	10	1	6	0.425468854	1.06E-05	no
CCND1	PROMOTER	Hs.523852	595	11	69160211	69160523	313	0	2	3	0.322886261	0.000408902	no
CD1D	PROMOTER	Hs.1799	912	1	156414173	156414588	416	2	0	1	0.331305716	0.004074142	no
CENPM	PROMOTER	Hs.208912	79019	22	40673163	40673415	253	1	0	1	0.495653137	0.000729383	no
CHCHD5	PROMOTER	Hs.375707	84269	2	113057990	113058420	431	2	0	5	0.329127528	0.00637525	no
CIRBP	PROMOTER	Hs.634522	1153	19	1218452	1218828	377	2	0	3	0.301333344	0.005747581	no
CNTD2	PROMOTER	Hs.631603	79935	19	45424464	45424795	332	1	2	1	0.400565717	0.0015404	no
COL24A1	PROMOTER	Hs.659516	255631	1	86394659	86395279	621	1	7	7	0.215801003	0.00637525	no
CPNE8	PROMOTER	Hs.40910	144402	12	37586447	37586931	485	1	3	3	0.186094835	0.007850636	no
CREBBP	PROMOTER	Hs.459759	1387	16	3871516	3871865	350	4	2	2	0.282465085	0.006390701	no
CROCC	PROMOTER	Hs.309403	9696	1	17112185	17112842	658	3	2	1	0.3894536	0.00597696	no
CYB5R2	PROMOTER	Hs.414362	51700	11	7651920	7652389	470	2	3	5	0.656716886	0.004121758	no
D21S2056E	PROMOTER		NA	21	44030586	44031811	1226	7	1	4	0.389426557	0.005527689	no
DCDC2	PROMOTER	Hs.659324	51473	6	24465443	24466471	1029	7	3	8	0.151653417	0.004322494	no
DDX4	PROMOTER	Hs.223581	54514	5	55068895	55069637	743	0	1	3	0.107497444	0.008013985	no
DGKI	PROMOTER	Hs.242947	9162	7	137181774	137182816	1043	12	14	8	-0.098998738	0.001354784	no
DIRAS2	PROMOTER	Hs.165636	54769	9	92444631	92445202	572	2	3	5	0.107694952	0.006665237	no
DLX1	PROMOTER	Hs.407015	1745	2	172655954	172656381	428	4	5	4	0.230878891	0.000987277	no
DLX6	PROMOTER	Hs.249196	1750	7	96472440	96472863	424	3	6	4	0.371733781	0.001261731	no
DMRTA1	PROMOTER	Hs.371976	63951	9	22436554	22436821	268	1	3	2	0.18653546	0.007090974	no
FAM127B	PROMOTER	Hs.460924	26071	X	134012843	134014349	1507	12	8	5	0.098053494	0.00489994	no
FAM24B	PROMOTER	Hs.114648	196792	10	124629320	124629892	573	3	7	4	0.176068763	0.004123255	no
FAM77D	PROMOTER		NA	8	63323138	63323712	575	5	10	5	0.324675572	5.87E-05	no
FAM77D	PROMOTER		NA	8	63322963	63323137	175	0	2	1	0.209923075	0.003574802	no
FEZF1	PROMOTER	Hs.553970	389549	7	121737367	121737769	403	1	5	4	0.245319521	0.002146598	no
FEZF1	PROMOTER	Hs.553970	389549	7	121737770	121738151	382	2	6	4	0.202570042	0.009126773	no
FLJ13305	PROMOTER	Hs.440466	84140	2	61934573	61935037	465	2	4	4	-0.105805822	0.008625148	no
FLJ36748	PROMOTER		NA	5	148631080	148632637	1558	16	19	9	0.089888982	0.004590488	no
FLJ45537	PROMOTER	Hs.657740	401535	9	88953160	88953856	697	5	7	5	0.169480902	0.003093037	no
FLJ46347	PROMOTER		NA	2	174914958	174915628	671	5	7	7	0.128952445	0.000975868	no
FNBP1	PROMOTER	Hs.189409	23048	9	131845650	131846291	642	4	3	3	0.724750112	0.000136259	no
FOXB1	PROMOTER	Hs.160375	27023	15	58076583	58077032	450	7	1	3	0.224135051	0.006216994	no
FOXB2	PROMOTER	Hs.553843	442425	9	78820845	78821525	681	8	5	7	0.255533748	0.001105907	no
FOXB2	PROMOTER	Hs.553843	442425	9	78818581	78818826	246	3	1	2	0.382709762	0.007441416	no
FOXD3	PROMOTER	Hs.546573	27022	1	63560388	63560797	410	3	4	3	0.359336963	0.000461399	no
FOXD3	PROMOTER	Hs.546573	27022	1	63559266	63559697	432	2	2	2	0.812445673	0.001392004	no
FOXD3	PROMOTER	Hs.546573	27022	1	63560088	63560334	247	0	3	2	0.309639668	0.001482741	no
GBX2	PROMOTER	Hs.184945	2637	2	236742623	236742879	257	2	1	2	0.40033932	0.000343362	no
GBX2	PROMOTER	Hs.184945	2637	2	236744782	236745370	589	3	1	5	0.171578512	0.001618035	no
Gcom1	PROMOTER	Hs.437256	145781	15	55670787	55671286	500	0	2	1	0.194175595	0.009764856	no
GIOT-1	PROMOTER		NA	19	41848369	41849711	1343	3	9	3	0.207816827	0.001663177	no
GIOT-1	PROMOTER		NA	19	41849712	41849982	271	4	2	2	0.180222123	0.009184469	no
GNG4	PROMOTER	Hs.159711	2786	1	233880863	233881157	295	2	0	2	0.2626796	0.008523264	no
GSK3A	PROMOTER	Hs.466828	2931	19	47441048	47441821	774	1	1	1	0.26795926	0.007748335	no
GSPT2	PROMOTER	Hs.59523	23708	X	51503099	51503386	288	1	1	1	0.275237122	0.004418351	no
GSTM3	PROMOTER	Hs.2006	2947	1	110084728	110085243	516	2	2	2	0.590768703	0.000166263	no
HELT	PROMOTER	Hs.531242	391723	4	186174639	186175527	889	2	0	2	0.366389171	6.65E-05	no
HELT	PROMOTER	Hs.531242	391723	4	186176506	186176785	280	3	2	4	0.228408243	0.002419601	no
HEY2	PROMOTER	Hs.144287	23493	6	126111401	126111888	488	4	1	4	0.718905845	0.000471486	no
HEY2	PROMOTER	Hs.144287	23493	6	126110841	126111400	560	4	5	5	0.149390198	0.005068387	no
HLX1	PROMOTER		NA	1	219118312	219119454	1143	4	11	11	0.116898836	0.001210989	no
HOXA3	PROMOTER	Hs.659337	3200	7	27121293	27122037	745	4	5	7	0.154904117	0.000863599	no
HRK	PROMOTER	Hs.87247	8739	12	115803763	115804390	628	9	3	4	0.137849081	0.001482741	no
HS6ST1	PROMOTER	Hs.512841	9394	2	128793643	128794144	502	2	5	5	0.136827181	0.006878481	no
hsa-mir-15b	PROMOTER		NA	3	161601199	161601874	676	7	8	5	-0.103064998	0.007179819	no
hsa-mir-183	PROMOTER		NA	7	129209327	129209732	406	1	4	4	0.725145907	0.000193688	no
hsa-mir-183	PROMOTER		NA	7	129209733	129210658	926	2	6	7	0.184448975	0.005941415	no
hsa-mir-200b	PROMOTER		NA	1	1089217	1089433	217	1	2	2	0.208892135	0.000664282	no
hsa-mir-200b	PROMOTER		NA	1	1090055	1090287	233	1	3	1	0.203904545	0.003844533	no
hsa-mir-548b	PROMOTER		NA	6	119440601	119440819	219	1	0	1	0.306554194	0.005704088	no
hsa-mir-561	PROMOTER		NA	2	188865720	188866305	586	3	2	3	0.320213671	0.001768986	no
hsa-mir-7-2	PROMOTER		NA	15	86948180	86948889	710	2	5	3	0.206665792	0.00043294	no
ID2	PROMOTER	Hs.180919	3398	2	8734051	8734473	423	3	6	4	0.099003095	0.006912265	no
IFNA8	PROMOTER	Hs.73890	3445	9	21392648	21393618	971	4	2	5	0.254362208	0.000793383	no
IGF2BP1	PROMOTER	Hs.144936	10642	17	44429039	44429240	202	1	4	2	0.473720678	0.002890578	no
INTS3	PROMOTER	Hs.516522	65123	1	151965818	151967910	2093	7	7	5	-0.137373956	0.006020137	no
KCTD8	PROMOTER	Hs.479644	386617	4	44145796	44146066	271	2	1	2	0.262095589	0.002979694	no
KLHL34	PROMOTER	Hs.448572	257240	X	21586875	21587478	604	3	2	2	0.166683932	0.001691162	no
Kua	PROMOTER		NA	20	48204012	48204328	317	1	2	2	-0.124353104	0.007381148	no
LEF1	PROMOTER	Hs.555947	51176	4	109311627	109312151	525	0	2	2	0.332597457	0.000504409	no
LEF1	PROMOTER	Hs.555947	51176	4	109312357	109312717	361	1	1	3	0.203857265	0.006390701	no

LHX4	PROMOTER	Hs.658487	89884	1	178464542	178464872	331	2	1	2	0.285372942	0.001148009	no
LHX9	PROMOTER	Hs.442578	56956	1	196147214	196147626	413	2	7	4	0.455501938	3.68E-05	no
LHX9	PROMOTER	Hs.442578	56956	1	196147888	196148437	550	2	8	6	0.26738041	0.004008308	no
LOC152573	PROMOTER	Hs.370904	152573	4	42093552	42094199	648	5	5	4	0.169847766	0.002630754	no
LOC152573	PROMOTER	Hs.370904	152573	4	42094234	42095613	1380	15	16	14	0.087979455	0.00732353	no
LOC286187	PROMOTER	Hs.632064	286187	8	68103182	68103842	661	4	7	5	0.726481434	0.000324328	no
LOC346157	PROMOTER		NA	6	27464325	27464703	379	1	0	3	0.353382381	0.005669947	no
LOC388931	PROMOTER		NA	2	24085970	24087368	1399	10	14	10	0.087228534	0.00383971	no
LOC389151	PROMOTER	Hs.531376	389151	3	140221195	140222808	1614	10	15	7	-0.102536209	0.006204594	no
LRAT	PROMOTER	Hs.658427	9227	4	155884372	155884859	488	5	8	6	0.358186938	0.001263186	no
LRGUK	PROMOTER	Hs.149774	136332	7	133462178	133463180	1003	4	0	4	0.175917445	0.001128576	no
LRRTM1	PROMOTER	Hs.591580	347730	2	80384769	80385136	368	2	4	4	0.307110257	8.85E-05	no
LY6G5C	PROMOTER	Hs.25738	80741	6	31758903	31759104	202	2	0	2	0.654536129	0.000988043	no
LY6G5C	PROMOTER	Hs.25738	80741	6	31759105	31759300	196	0	1	1	0.289807622	0.003454464	no
LYPD1	PROMOTER	Hs.694844	116372	2	133144875	133145523	649	1	8	6	0.453650381	5.61E-06	no
MANBAL	PROMOTER	Hs.6126	63905	20	35351286	35352043	758	4	9	4	-0.115769095	0.009264995	no
MCOLN2	PROMOTER	Hs.591446	255231	1	85236379	85236881	503	3	3	5	0.505966131	0.001028097	no
MED12L	PROMOTER	Hs.58561	116931	3	152286577	152287389	813	5	10	7	0.188706957	0.00567102	no
MEF2C	PROMOTER	Hs.699175	4208	5	88215503	88216206	704	7	2	7	0.270017686	0.000578172	no
MEGF10	PROMOTER	Hs.438709	84466	5	126653893	126654805	913	4	11	9	0.197838476	0.003054314	no
MEIS1	PROMOTER	Hs.526754	4211	2	66515620	66515851	232	2	1	2	0.889063822	2.79E-09	no
MEIS1	PROMOTER	Hs.526754	4211	2	66514500	66515619	1120	12	11	10	0.091472129	0.002716778	no
MLF1	PROMOTER	Hs.85195	4291	3	159771048	159771729	682	3	3	3	0.312229909	0.00033289	no
MOCS1	PROMOTER	Hs.357128	4337	6	40009662	40010488	827	4	11	6	0.064846389	0.0074411	no
MRPS31	PROMOTER	Hs.154655	10240	13	40243025	40243622	598	2	7	5	-0.115883619	0.005343603	no
MTERF	PROMOTER	Hs.532216	7978	7	91347860	91348424	565	4	3	5	0.417483927	0.002025207	no
MTHFSD	PROMOTER	Hs.343627	64779	16	85146740	85147268	529	1	1	4	0.582381578	6.20E-05	no
MYOCD	PROMOTER	Hs.567641	93649	17	12509786	12510029	244	1	4	2	0.8628087	9.38E-05	no
MYOCD	PROMOTER	Hs.567641	93649	17	12508943	12509785	843	7	7	5	0.179504143	0.00593266	no
NKD2	PROMOTER	Hs.240951	85409	5	1056657	1056985	329	0	1	1	0.319207866	0.000368969	no
NR4A3	PROMOTER	Hs.279522	8013	9	101627226	101627606	381	1	1	3	0.736333547	2.68E-06	no
OS9	PROMOTER	Hs.527861	10956	12	56373924	56375079	1156	0	3	5	-0.141550481	0.003613413	no
PAX3	PROMOTER	Hs.42146	5077	2	222876016	222876986	971	3	2	1	0.642392881	2.37E-05	no
PAX3	PROMOTER	Hs.42146	5077	2	222875363	222875684	322	0	5	3	0.263796818	0.004590488	no
PCOLCE2	PROMOTER	Hs.8944	26577	3	144090641	144090974	334	0	5	2	0.203724354	0.001806125	no
PDE3A	PROMOTER	Hs.591150	5139	12	20412555	20413262	708	4	5	5	0.361637409	0.002277235	no
POMC	PROMOTER	Hs.1897	5443	2	25245191	25245590	400	0	1	2	0.230046767	0.00632089	no
PPP2R2B	PROMOTER	Hs.655213	5521	5	146238541	146238920	380	4	1	4	0.410165577	0.003797662	no
PRDM16	PROMOTER	Hs.99500	63976	1	2972331	2974091	1761	3	9	4	0.139048794	0.001485121	no
PRKG1	PROMOTER	Hs.654556	5592	10	52504093	52504797	705	5	5	6	0.198095596	0.001284104	no
PTBP1	PROMOTER	Hs.172550	5725	19	746087	746615	529	1	6	3	0.197919569	0.009264995	no
QARS	PROMOTER	Hs.79322	5859	3	49117323	49117802	480	0	3	1	0.240485896	0.002630754	no
RAB3C	PROMOTER	Hs.482173	115827	5	57914150	57914516	367	1	0	1	0.370089034	0.001243008	no
RASL10B	PROMOTER	Hs.437035	91608	17	31082250	31082682	433	0	1	1	0.244598318	0.008342725	no
RBP1	PROMOTER	Hs.529571	5947	3	140740790	140741449	660	5	9	6	0.344586903	0.000597721	no
RERG	PROMOTER	Hs.199487	85004	12	15265180	15265760	581	5	3	7	0.18701555	0.00637613	no
RNF165	PROMOTER	Hs.501114	494470	18	42166806	42167456	651	2	0	2	0.457955834	0.000238612	no
RPRM	PROMOTER	Hs.100890	56475	2	154042584	154043826	1243	11	10	14	0.101809409	0.00861713	no
RRP15	PROMOTER	Hs.660109	51018	1	216524376	216524938	563	3	2	4	0.154113159	0.002069312	no
RUNX1T1	PROMOTER	Hs.368431	862	8	93184958	93185358	401	1	5	3	0.30486373	0.001286966	no
SDK1	PROMOTER	Hs.655699	221935	7	3306223	3306872	650	0	4	1	0.375001152	0.000380469	no
SIX3	PROMOTER	Hs.658847	6496	2	45016209	45016438	230	2	4	2	0.436526866	0.005669947	no
SLC26A5	PROMOTER	Hs.585146	375611	7	102874053	102874241	189	0	1	1	0.21598799	0.00312403	no
SLIT2	PROMOTER	Hs.699467	9353	4	19862706	19863582	877	5	13	10	0.145838909	0.000601049	no
SLIT2	PROMOTER	Hs.699467	9353	4	19862221	19862705	485	5	3	5	0.217439498	0.000831518	no
SMO	PROMOTER	Hs.437846	6608	7	128614941	128615896	956	8	9	4	0.161879045	0.000296467	no
SP8	PROMOTER	Hs.195922	221833	7	20797116	20797816	701	2	5	3	0.315611592	0.001543497	no
SPO11	PROMOTER	Hs.159737	23626	20	55338007	55338795	789	4	2	5	-0.141556126	0.006398142	no
ST8SIA1	PROMOTER	Hs.408614	6489	12	22379428	22379748	321	6	3	2	0.313751704	0.007203048	no
STCH	PROMOTER	Hs.352341	6782	21	14677817	14678101	285	1	1	2	0.286651935	0.006348036	no
SUNC1	PROMOTER	Hs.406741	256979	7	48041130	48042676	1547	12	13	13	0.104877449	0.005012074	no
SYPL2	PROMOTER	Hs.528366	284612	1	109810333	109810621	289	2	4	2	0.626975821	1.00E-05	no
SYT7	PROMOTER	Hs.502730	9066	11	61111242	61111718	477	3	0	2	0.20937016	0.002630754	no
TBC1D9	PROMOTER	Hs.480819	23158	4	141897206	141897500	295	4	2	3	0.491471267	0.004031106	no
TBC1D9	PROMOTER	Hs.480819	23158	4	141897501	141897709	209	0	1	2	0.163825001	0.007179819	no
TCF4	PROMOTER	Hs.644653	6925	18	51407883	51408355	473	2	2	4	0.264192474	0.001148009	no
TMEM22	PROMOTER	Hs.655019	80723	3	138020344	138021206	863	7	11	10	0.107313382	0.002648964	no
TNFRSF9	PROMOTER	Hs.654459	3604	1	7924442	7925553	1112	7	1	5	0.209758983	0.000622963	no
TOMM70A	PROMOTER	Hs.227253	9868	3	101603422	101603672	251	0	2	2	0.405533381	0.009126773	no
TRAM1L1	PROMOTER	Hs.570737	133022	4	118225682	118226395	714	1	6	6	0.143544795	0.004151773	no
TRIM71	PROMOTER	Hs.567678	131405	3	32833099	32833780	682	17	6	5	0.2912125	0.004799771	no
TSHR	PROMOTER	Hs.160411	7253	14	80490970	80491378	409	5	1	4	0.20162378	0.006914411	no
UBE2E2	PROMOTER	Hs.475688	7325	3	23218727	23219238	512	1	2	3	0.606463522	1.35E-05	no
WDR54	PROMOTER	Hs.643480	84058	2	74495441	74497050	1610	6	14	16	-0.111371143	0.001574342	no
WFDC2	PROMOTER	Hs.2719	10406	20	43531619	43531779	161	3	1	1	0.419183843	0.004084275	no
ZFP3	PROMOTER	Hs.48832	124961	17	4922101	4922964	864	7	10	7	0.233702721	0.000980559	no
ZNF302	PROMOTER	Hs.436350	55900	19	39859544	39860250	707	1	3	4	0.204796309	0.001210989	no
ZNF347	PROMOTER	Hs.467239	84671	19	58354050	58354331	282	2	4	3	0.33267127	0.005315293	no
ZNF470	PROMOTER	Hs.204449	388566	19	61770987	61771665	679	0	5	2	0.293193995	0.003636031	no
ZNF502	PROMOTER	Hs.224843	91392	3	44728933	44729161	229	3	2	1	0.232147648	0.006697532	no
ZNF533	PROMOTER	Hs.655005	151126	2	180434610	180435004	395	3	4	2	0.22057106	0.002854594	no
ZNF577	PROMOTER	Hs.148322	84765	19	57082594	57083183	590	5	2	5	0.276362751	0.002630754	no
ZNF678	PROMOTER	Hs.656372	339500	1	225813299	225815584	2286	9	13	9	-0.122498173	0.003797662	no
ZNRF4	PROMOTER	Hs.126496	148066	19	5406047	5406527	481	0	5	1	0.303263442	0.009264995	no
ZSCAN5	PROMOTER	Hs.177688	79149	19	61431293	61431643	351	1	3	2	0.213349606	0.006512357	no
ALX4	INSIDE	Hs.436055	60529	11	44283799	44284349	551	4	4	5	0.331989485	0.000668866	yes
ARHGAP9	INSIDE	Hs.437126	64333	12	56155101	56156473	1373	8	12	15	0.11099664	0.000787137	yes
B4GALNT1	INSIDE	Hs.591019	2583	12	56312448	56313083	636	9	10	6	0.109133302	0.000729383	yes
B4GALNT1	INSIDE	Hs.591019	2583	12	56307743	56308507	765	5	5	7	0.271318679	0.002988163	yes
BACH2	INSIDE	Hs.269764	60468	6	91061442	91062429	988	13	12	10	0.116417481	0.002707936	yes

BHLHB5	INSIDE	Hs.388788	27319	8	65656636	65656998	363	3	6	2	0.280204383	0.00637613	yes
BMP6	INSIDE	Hs.285671	654	6	7671226	7671603	378	8	4	3	0.24466334	0.001090425	yes
BMP6	INSIDE	Hs.285671	654	6	7673194	7674075	882	3	8	3	0.384850719	0.00121363	yes
C12orf39	INSIDE	Hs.130692	80763	12	21571871	21572435	565	5	8	5	0.181675108	0.001292103	yes
C4orf22	INSIDE	Hs.527104	255119	4	81475832	81476465	634	7	4	4	0.150260712	0.006216994	yes
CALCA	INSIDE	Hs.37058	796	11	14949872	14950696	825	2	1	4	0.240869151	0.002661375	yes
CDKN2A	INSIDE	Hs.512599	1029	9	21983369	21984414	1046	3	5	4	0.121562473	0.005478254	yes
CLDN11	INSIDE	Hs.31595	5010	3	171619320	171619939	620	2	2	5	0.151098114	0.0073902	yes
COMP	INSIDE	Hs.1584	1311	19	18759059	18759477	419	0	4	4	0.377919997	0.002665988	yes
DCC	INSIDE	Hs.579550	1630	18	48122354	48122874	521	7	5	5	0.160643906	0.00092553	yes
DSC3	INSIDE	Hs.41690	1825	18	26875421	26876732	1312	14	11	13	0.310516243	0.000162574	yes
DUOX2	INSIDE	Hs.71377	50506	15	43192510	43193155	646	1	1	3	0.258937849	0.005012074	yes
DUOXA2	INSIDE	Hs.497987	405753	15	43193943	43194818	876	2	4	3	0.317992682	0.005576168	yes
EPHB3	INSIDE	Hs.2913	2049	3	185763281	185763779	499	1	3	2	0.363175939	9.18E-05	yes
FAM123A	INSIDE	Hs.528335	219287	13	24641475	24642005	531	0	3	1	0.160170021	0.008806197	yes
FLJ44815	INSIDE	Hs.514090	400591	17	29929984	29930462	479	3	5	5	0.120901348	0.009214112	yes
FLJ45983	INSIDE	Hs.669736	399717	10	8133177	8134109	933	4	17	10	0.127072665	0.005841944	yes
FLJ46831	INSIDE		NA	10	129426531	129427122	592	2	2	7	0.307825044	0.009335554	yes
FOXF2	INSIDE	Hs.484423	2295	6	1338964	1339624	661	1	14	3	0.125677678	0.003636031	yes
HAND1	INSIDE	Hs.152531	9421	5	153837431	153837797	367	2	5	3	0.467191269	0.000438954	yes
HAND1	INSIDE	Hs.152531	9421	5	153836059	153836436	378	1	4	3	0.229112574	0.007748335	yes
HOXC4	INSIDE	Hs.549040	3221	12	52733972	52734774	803	2	5	4	0.204504393	0.009264995	yes
HTRA1	INSIDE	Hs.501280	5654	10	124212079	124213023	945	2	7	2	0.160738562	0.004848817	yes
JMJD3	INSIDE	Hs.223678	23135	17	7697264	7697706	443	5	5	4	0.182643325	0.003425946	yes
LBX1	INSIDE	Hs.37128	10660	10	102977666	102978273	608	2	4	6	0.299879234	0.003054314	yes
LOC375449	INSIDE		NA	5	65928841	65929437	597	5	1	5	0.239452414	0.000578172	yes
LOC439985	INSIDE	Hs.704151	439985	10	76827314	76827568	255	1	2	2	0.215725737	0.005154954	yes
MAB21L1	INSIDE	Hs.584776	4081	13	34947753	34948527	775	3	8	6	0.527548165	0.006204594	yes
MAPK4	INSIDE	Hs.433728	5596	18	46341077	46341624	548	2	7	6	0.248791526	0.00027303	yes
MEGF11	INSIDE	Hs.438250	84465	15	64331251	64331650	400	1	9	2	0.193405214	0.002696034	yes
MGC39900	INSIDE	Hs.675540	286527	X	103104232	103104897	666	1	3	3	0.257983123	0.005014718	yes
MKX	INSIDE	Hs.128193	283078	10	28073072	28073988	917	17	4	8	0.266184435	3.32E-05	yes
MKX	INSIDE	Hs.128193	283078	10	28071999	28073071	1073	6	14	11	0.277344391	0.000324328	yes
MKX	INSIDE	Hs.128193	283078	10	28071089	28071309	221	3	2	2	0.230345303	0.002604011	yes
MKX	INSIDE	Hs.128193	283078	10	28070263	28070967	705	4	5	9	0.164999883	0.005647265	yes
NOL4	INSIDE	Hs.514795	8715	18	30057056	30057488	433	2	4	4	0.436180878	0.00092553	yes
NOL4	INSIDE	Hs.514795	8715	18	30056745	30057055	311	2	3	2	0.240140504	0.002827751	yes
NR2E1	INSIDE	Hs.157688	7101	6	108603450	108603769	320	1	3	3	0.101609152	0.005055513	yes
OTP	INSIDE	Hs.202247	23440	5	76961846	76962779	934	3	10	9	0.273950701	0.000175846	yes
PAX2	INSIDE	Hs.155644	5076	10	102498612	102498922	311	1	2	2	0.192447333	0.001951032	yes
PCDHGC5	INSIDE		NA	5	140851626	140853100	1475	10	15	11	-0.092285179	0.006501146	yes
PHACTR1	INSIDE	Hs.436996	221692	6	13122708	13123493	786	3	3	5	0.175022518	0.000438954	yes
RAB31	INSIDE	Hs.99528	11031	18	9698902	9699163	262	2	1	2	0.295825341	3.68E-05	yes
RNF39	INSIDE	Hs.121178	80352	6	30146752	30148179	1428	6	3	9	-0.121376533	0.006692289	yes
SCARF2	INSIDE	Hs.474251	91179	22	19108894	19109353	460	1	6	1	0.531910454	0.000208174	yes
SHC4	INSIDE	Hs.642615	399694	15	47042094	47042663	570	1	5	5	0.629151761	9.38E-06	yes
SIM1	INSIDE	Hs.520293	6492	6	101010265	101010587	323	1	2	2	0.400332527	0.005941415	yes
SIX1	INSIDE	Hs.633506	6495	14	60185144	60185889	746	7	4	6	0.194184531	0.000286326	yes
SIX1	INSIDE	Hs.633506	6495	14	60184546	60185143	598	8	4	7	0.301935644	0.000450862	yes
SLC5A5	INSIDE	Hs.584804	6528	19	17844606	17844972	367	3	4	2	0.568689808	0.000597721	yes
ST8SIA4	INSIDE	Hs.308628	7903	5	100264656	100265360	705	0	4	3	0.401113855	0.000622963	yes
STMN2	INSIDE	Hs.521651	11075	8	80687259	80688418	1160	11	11	12	0.1078783	0.006398563	yes
TBX2	INSIDE	Hs.705451	6909	17	56834955	56835612	658	5	3	5	0.518803713	3.72E-05	yes
TBX2	INSIDE	Hs.705451	6909	17	56832907	56833273	367	3	3	2	0.436095579	0.002592306	yes
TCF8	INSIDE		NA	10	31649152	31649545	394	3	7	4	0.491581369	0.001243008	yes
TRIM36	INSIDE	Hs.519514	55521	5	114542612	114542992	381	3	3	3	0.24680307	0.000332888	yes
WNT1	INSIDE	Hs.248164	7471	12	47658584	47659400	817	5	3	9	0.151568459	0.000633333	yes
WNT5A	INSIDE	Hs.696364	7474	3	55495188	55495452	265	3	3	3	0.300933663	0.003182178	yes
ZFPM2	INSIDE	Hs.431009	23414	8	106400652	106401462	811	7	5	6	0.209347212	0.008287601	yes
ZIC1	INSIDE	Hs.598590	7545	3	148612866	148613353	488	1	1	4	0.229397727	0.002725499	yes
ABR	INSIDE	Hs.159306	29	17	879681	880221	541	0	3	3	0.15517472	0.008855542	no
ADAMTS19	INSIDE	Hs.23751	171019	5	128824265	128824660	396	2	0	4	0.574579197	0.001618035	no
ADAMTS3	INSIDE	Hs.590919	9508	4	73652608	73653426	819	0	7	6	0.13948964	0.004484579	no
ADD2	INSIDE	Hs.188528	119	2	70848610	70848803	194	2	0	2	0.142026044	0.005941415	no
ADRA1D	INSIDE	Hs.557	146	20	4149354	4150170	817	1	2	1	0.1744941	0.005792302	no
ANK1	INSIDE	Hs.654438	286	8	41744279	41744619	341	2	5	3	0.128891871	0.001354784	no
ANKRD38	INSIDE	Hs.283398	163782	1	62556369	62557203	835	3	2	5	0.21116771	0.000597721	no
ARHGEF6	INSIDE	Hs.522795	9459	X	135676642	135677301	660	6	4	7	0.133393035	0.00147928	no
ARID3A	INSIDE	Hs.501296	1820	19	892993	893528	536	2	7	6	0.180852792	0.004507646	no
ATCAY	INSIDE	Hs.418055	85300	19	3858477	3859012	536	1	6	5	0.234125407	0.002357812	no
BAPX1	INSIDE		NA	4	13152424	13153040	617	4	6	4	0.358635008	0.000176079	no
BC37295_3	INSIDE	Hs.458438	90485	19	61873468	61875702	2235	6	8	8	-0.10705879	0.005111304	no
BRD3	INSIDE	Hs.522472	8019	9	135904131	135904633	503	1	2	6	0.149997349	0.008300526	no
C15orf26	INSIDE	Hs.130979	161502	15	79213504	79214216	713	2	3	2	0.432594773	0.00102355	no
C1orf164	INSIDE	Hs.456557	55182	1	44854835	44855693	859	7	8	4	0.335120098	9.42E-06	no
C3orf21	INSIDE	Hs.478741	152002	3	196472109	196472320	212	1	0	1	0.258436868	0.003721962	no
C4orf31	INSIDE	Hs.90250	79625	4	122212340	122212599	260	1	0	2	0.261187	0.0073902	no
CACNB4	INSIDE	Hs.614033	785	2	152662830	152663409	580	6	9	5	0.211951739	0.000291544	no
CASKIN1	INSIDE		NA	16	2176282	2177434	1153	0	8	5	0.226992706	0.002069312	no
CAV1	INSIDE	Hs.74034	857	7	115953622	115954375	754	4	1	5	0.255940329	0.004697439	no
CCDC36	INSIDE	Hs.631931	339834	3	49210711	49211719	1009	2	3	2	0.195926457	0.008263145	no
CCL28	INSIDE	Hs.656904	56477	5	43432912	43433199	288	6	3	2	0.504846761	0.001029915	no
CCL28	INSIDE	Hs.656904	56477	5	43432537	43432911	375	4	1	2	0.378476611	0.008612085	no
CDH4	INSIDE	Hs.473231	1002	20	59927640	59929852	2213	4	2	4	-0.36789486	0.009254494	no
CDK2AP2	INSIDE	Hs.523835	10263	11	67031204	67031673	470	0	2	2	0.120568064	0.0082658	no
CDKN2A	INSIDE	Hs.512599	1029	9	21964346	21965045	700	4	4	6	0.304468477	0.001609727	no
CHST10	INSIDE	Hs.516370	9486	2	100399487	100400084	598	1	1	1	0.3009168	0.005518027	no
CHST6	INSIDE	Hs.655622	4166	16	74085777	74086498	722	1	3	2	0.432867003	0.001822638	no
CLDN4	INSIDE	Hs.647036	1364	7	72883378	72884400	1023	4	6	6	0.25654841	0.000774416	no
CLYBL	INSIDE	Hs.655642	171425	13	99345610	99345932	323	9	1	3	0.210862686	0.003797662	no

COL24A1	INSIDE	Hs.659516	255631	1	86392965	86394658	1694	10	3	10	0.162218829	0.00597696	no
COL4A2	INSIDE	Hs.508716	1284	13	109758900	109759172	273	1	3	2	0.367054906	9.38E-06	no
COL5A1	INSIDE	Hs.210283	1289	9	136873791	136874453	663	2	5	6	0.256610533	2.03E-05	no
CPSF1	INSIDE	Hs.493202	29894	8	145591998	145592867	870	4	8	4	0.160554049	0.002725499	no
CR2	INSIDE	Hs.445757	1380	1	205694271	205694997	727	8	7	8	0.217359885	0.000922325	no
CRABP1	INSIDE	Hs.346950	1381	15	76420341	76420862	522	2	2	5	0.532747546	1.00E-05	no
CRABP1	INSIDE	Hs.346950	1381	15	76420863	76421131	269	2	1	3	0.125233644	0.00732353	no
CREBBP	INSIDE	Hs.459759	1387	16	3715771	3716613	843	2	3	6	-0.139650323	0.00817337	no
CRMP1	INSIDE	Hs.135270	1400	4	5939785	5940244	460	1	4	2	0.339121859	0.001030731	no
CRTC1	INSIDE	Hs.371096	23373	19	18734212	18734650	439	1	2	2	0.265547597	0.009553442	no
CSMD2	INSIDE	Hs.656915	114784	1	34401058	34401661	604	3	4	4	0.231824685	0.000368969	no
CSPG2	INSIDE		NA	5	82805735	82806311	577	1	3	4	0.131993883	0.006073278	no
CUGBP2	INSIDE	Hs.309288	10659	10	11247148	11247520	373	0	2	4	-0.218312436	0.004420233	no
CUTL2	INSIDE		NA	12	109959453	109959677	225	0	2	1	0.553718914	0.000533593	no
CYP1B1	INSIDE	Hs.154654	1545	2	38156559	38156826	268	1	1	1	0.818663352	0.000194418	no
DAB2IP	INSIDE	Hs.522378	153090	9	123400179	123400372	194	0	1	2	0.205531383	0.005951267	no
DDIT4L	INSIDE	Hs.480378	115265	4	101329831	101330988	1158	5	12	7	0.130342914	0.009198677	no
DENND1C	INSIDE	Hs.236449	79958	19	6426524	6427133	610	4	1	6	0.445629667	2.13E-05	no
DENND2C	INSIDE	Hs.654928	163259	1	115013666	115014093	428	3	3	4	0.31334745	3.76E-05	no
DLX2	INSIDE	Hs.419	1746	2	172674353	172675068	716	2	5	8	0.148650495	0.008806197	no
DUSP4	INSIDE	Hs.417962	1846	8	29250716	29251058	343	2	1	1	0.399010328	0.0073902	no
EDG1	INSIDE	Hs.154210	1901	1	101474991	101475322	332	5	2	3	0.358319064	0.001647154	no
ELOVL5	INSIDE	Hs.520189	60481	6	53320555	53321007	453	1	3	4	0.175759511	0.009264995	no
EMX1	INSIDE	Hs.516090	2016	2	73001251	73002887	1637	3	7	6	0.125751852	0.003721962	no
ENTPD4	INSIDE	Hs.444389	9583	8	23370487	23370641	155	1	0	1	0.292081127	0.001097114	no
EPHA3	INSIDE	Hs.123642	2042	3	89246558	89246762	205	2	0	2	0.264409242	0.003688157	no
FAM19A5	INSIDE	Hs.436854	25817	22	47405256	47405926	671	11	1	3	0.429580418	0.001128576	no
FAM19A5	INSIDE	Hs.436854	25817	22	47532700	47533272	573	3	7	6	0.178789632	0.00722389	no
FBXL10	INSIDE	Hs.524800	84678	12	120366437	120366718	282	0	4	2	0.245140182	0.00637613	no
FBXL14	INSIDE	Hs.367956	144699	12	1571733	1572970	1238	4	3	2	0.155999003	0.004582759	no
FLJ30058	INSIDE		NA	X	130034242	130034847	606	1	2	4	0.14676178	0.001296472	no
FLJ37440	INSIDE		NA	2	112655492	112656246	755	7	6	6	0.331456402	0.003787918	no
FNBP1	INSIDE	Hs.189409	23048	9	131843862	131844333	472	0	4	3	0.148809363	0.005865743	no
FSTL1	INSIDE	Hs.269512	11167	3	121651785	121652042	258	1	0	2	0.411676851	5.06E-05	no
FSTL1	INSIDE	Hs.269512	11167	3	121652051	121652555	505	9	6	6	0.151927796	0.003721962	no
GABRA5	INSIDE	Hs.612087	2558	15	24679011	24679632	622	2	3	3	0.284496697	0.006059018	no
GAK	INSIDE	Hs.369607	2580	4	886725	889155	2431	8	10	2	0.225869756	5.28E-05	no
GALR2	INSIDE	Hs.666366	8811	17	71582754	71583229	476	6	0	4	0.702575266	5.61E-06	no
GATA4	INSIDE	Hs.243987	2626	8	11644563	11645255	693	1	4	6	0.274079595	0.001485483	no
GATAD2A	INSIDE	Hs.696033	54815	19	19472638	19473316	679	1	4	6	0.314541248	0.001822638	no
GBX2	INSIDE	Hs.184945	2637	2	236739948	236740439	492	1	1	4	0.350856706	1.66E-05	no
GBX2	INSIDE	Hs.184945	2637	2	236739143	236739947	805	3	4	8	0.208743785	0.005236312	no
GFI1	INSIDE	Hs.73172	2672	1	92719872	92720488	617	1	6	7	0.447856056	0.001845025	no
GFI1B	INSIDE	Hs.553160	8328	9	134854651	134855268	618	1	5	4	0.13694093	0.002357812	no
GNG7	INSIDE	Hs.515544	2788	19	2601554	2601967	414	1	2	4	0.255030613	0.005846864	no
GPR123	INSIDE	Hs.435183	84435	10	134746728	134747160	433	2	2	4	0.378496697	7.05E-05	no
GPR123	INSIDE	Hs.435183	84435	10	134763049	134763758	710	0	6	3	0.169037009	0.001642501	no
GPR133	INSIDE	Hs.656754	283383	12	130079402	130079695	294	1	0	1	0.382818508	0.000362526	no
GREB1	INSIDE	Hs.467733	9687	2	11691457	11692607	1151	0	5	4	0.235689516	0.004945192	no
GRIK5	INSIDE	Hs.367799	2901	19	47238252	47238977	726	0	5	5	0.145216863	0.004484579	no
GSH2	INSIDE		NA	4	54661583	54662079	497	7	1	3	0.256808922	0.001097114	no
GSTM3	INSIDE	Hs.2006	2947	1	110083813	110084167	355	2	4	3	0.447145302	0.002609825	no
GTF2IRD1	INSIDE	Hs.647056	9569	7	73532901	73533419	519	1	3	2	0.157849835	0.005320685	no
HAL	INSIDE	Hs.190783	3034	12	94913418	94914214	797	3	4	5	0.174495397	0.007072356	no
HDGF2	INSIDE	Hs.43071	84717	19	4433648	4434145	498	2	3	3	0.19726155	0.00728138	no
HHIP	INSIDE	Hs.507991	64399	4	145786728	145787297	570	2	1	2	0.394344369	0.001261731	no
HOP	INSIDE		NA	4	57216146	57217322	1177	12	14	11	0.09823356	0.007397257	no
IGSF22	INSIDE	Hs.434152	283284	11	18683842	18684419	578	6	1	6	0.36293077	3.42E-05	no
INHBB	INSIDE	Hs.1735	3625	2	120821026	120821409	384	1	3	2	0.205673992	0.002661375	no
INSR	INSIDE	Hs.465744	3643	19	7218659	7219140	482	0	7	1	0.431108354	0.005110676	no
IQSEC3	INSIDE	Hs.536319	440073	12	84329	84626	298	2	2	2	0.197188913	0.006216994	no
IRX2	INSIDE	Hs.282089	153572	5	2801242	2803304	2063	20	28	20	-0.077989351	0.000953447	no
KATNAL2	INSIDE	Hs.404137	83473	18	42780750	42781032	283	1	2	3	0.759207064	0.002694268	no
KBTBD7	INSIDE	Hs.63841	84078	13	40665046	40666273	1228	2	0	2	0.213601391	0.005080855	no
KCNG2	INSIDE	Hs.247905	26251	18	75743592	75744482	891	17	13	7	0.158450638	0.007396042	no
KCTD16	INSIDE	Hs.693927	57528	5	143564694	143565085	392	3	1	3	0.198617641	0.002025207	no
KHSRP	INSIDE	Hs.699378	8570	19	6365285	6365695	411	4	4	4	0.182446094	0.005647265	no
KIAA0427	INSIDE	Hs.145230	9811	18	44541459	44542185	727	1	2	4	0.29664937	0.00027303	no
KIAA0664	INSIDE	Hs.22616	23277	17	2539485	2539875	391	0	3	2	0.295967728	0.001974976	no
KIAA0738	INSIDE		NA	7	143212750	143213598	849	5	5	5	-0.110564973	0.002696034	no
LAPTM4B	INSIDE	Hs.492314	55353	8	98857878	98858110	233	2	0	2	0.421514996	0.009694492	no
LDLRAD3	INSIDE	Hs.700909	143458	11	35922893	35923425	533	2	3	2	0.285807495	0.000381174	no
LHX2	INSIDE	Hs.696425	9355	9	125818886	125819237	352	1	2	3	0.334698309	0.000175846	no
LHX3	INSIDE	Hs.148427	8022	9	138232086	138232575	490	1	1	5	0.252027412	0.001642501	no
LHX4	INSIDE	Hs.658487	89884	1	178467921	178468646	726	2	1	6	0.537337084	1.58E-05	no
LHX4	INSIDE	Hs.658487	89884	1	178470713	178471227	515	5	7	5	0.139495141	0.003805037	no
LHX6	INSIDE	Hs.103137	26468	9	124029808	124030013	206	1	0	1	0.730528882	3.67E-05	no
LHX6	INSIDE	Hs.103137	26468	9	124029344	124029714	371	2	6	3	0.31439457	0.000494033	no
LHX9	INSIDE	Hs.442578	56956	1	196153802	196154436	635	3	6	6	0.318152098	0.003805037	no
LHX9	INSIDE	Hs.442578	56956	1	196153095	196153801	707	3	3	2	0.260123734	0.004508421	no
LMNB2	INSIDE	Hs.538286	84823	19	2395177	2396050	874	0	5	4	0.140777638	0.008457193	no
LOX	INSIDE	Hs.102267	4015	5	121440320	121441878	1559	16	15	16	0.166392125	0.001148009	no
LRP1B	INSIDE	Hs.656461	53353	2	142604599	142605113	515	2	2	5	0.389097059	0.001091076	no
LTBP4	INSIDE	Hs.466766	8425	19	45821663	45822019	357	0	2	4	0.180602337	0.00722294	no
MAMDC2	INSIDE	Hs.547172	256691	9	71848555	71848940	386	2	3	4	0.377746735	0.001173236	no
MARVELD2	INSIDE	Hs.657687	153562	5	68747300	68747534	235	2	2	1	0.2508024	0.000450862	no
MATN4	INSIDE	Hs.278489	8785	20	43359702	43360307	606	2	3	4	0.184675849	0.000130634	no
MC5R	INSIDE	Hs.248145	4161	18	13815709	13816542	834	4	2	4	0.160959752	0.008307478	no
MCAM	INSIDE	Hs.599039	4162	11	118686343	118687097	755	0	4	6	0.176934098	0.008024183	no
MEGF10	INSIDE	Hs.438709	84466	5	126654806	126655234	429	0	1	1	0.4471925	0.000762312	no

MEGF11	INSIDE	Hs.438250	84465	15	64331651	64332092	442	4	6	3	0.224542173	0.00728138	no
METRNL	INSIDE	Hs.591142	284207	17	78635510	78636204	695	0	4	4	0.172705647	0.002592306	no
MKX	INSIDE	Hs.128193	283078	10	28071310	28071982	673	5	10	6	0.170873969	0.000424888	no
MLF1	INSIDE	Hs.85195	4291	3	159771894	159772121	228	1	0	1	0.282301319	0.005363106	no
MMP21	INSIDE	Hs.314141	118856	10	127454030	127454442	413	1	2	4	0.273713201	0.002118288	no
MN1	INSIDE	Hs.268515	4330	22	26524857	26525520	664	5	7	5	0.270463726	3.23E-05	no
NBPF1	INSIDE	Hs.445080	55672	1	16811741	16812715	975	15	4	1	0.415526539	0.001578565	no
NFATC1	INSIDE	Hs.534074	4772	18	75348686	75349174	489	5	2	2	0.361121578	0.004962391	no
NFATC1	INSIDE	Hs.534074	4772	18	75319942	75320476	535	1	2	4	0.186128379	0.005348083	no
NFATC1	INSIDE	Hs.534074	4772	18	75309373	75310098	726	1	5	6	0.202717123	0.008115906	no
NFIC	INSIDE	Hs.170131	4782	19	3348765	3349835	1071	0	7	3	0.125563783	0.009028653	no
NHEJ1	INSIDE	Hs.225988	79840	2	219732913	219733072	160	1	1	1	0.225601637	0.003009101	no
NKX6-1	INSIDE	Hs.546270	4825	4	85633773	85634023	251	1	0	2	0.198242802	0.003346777	no
NPHS1	INSIDE	Hs.590942	4868	19	41026462	41027238	777	6	2	5	0.30343827	0.001842205	no
NPR1	INSIDE	Hs.490330	4881	1	151928710	151929324	615	0	4	4	0.346522723	0.000725792	no
NPTX2	INSIDE	Hs.3281	4885	7	98085361	98085600	240	5	4	2	0.501629386	0.000809454	no
NPY1R	INSIDE	Hs.519057	4886	4	164472772	164473118	347	3	3	2	0.333689402	0.000450862	no
NPY1R	INSIDE	Hs.519057	4886	4	164472412	164472771	360	7	3	4	0.141962846	0.008443375	no
NPY5R	INSIDE	Hs.519058	4889	4	164484465	164485320	856	11	13	10	0.109767973	0.006020137	no
NR5A2	INSIDE	Hs.33446	2494	1	198270992	198271505	514	4	1	5	0.353862348	0.002160094	no
NTNG1	INSIDE	Hs.657434	22854	1	107485076	107485290	215	0	1	1	0.254950887	0.008592457	no
NTNG1	INSIDE	Hs.657434	22854	1	107484497	107484972	476	2	5	4	0.177877857	0.008641899	no
OCA2	INSIDE	Hs.654411	4948	15	26017457	26017999	543	3	2	3	0.197511214	0.000953447	no
OSBP2	INSIDE	Hs.517546	23762	22	29547883	29548276	394	1	1	1	0.200091387	0.000671111	no
OSBP2	INSIDE	Hs.517546	23762	22	29631543	29632380	838	3	11	8	0.111496852	0.009694492	no
PAX5	INSIDE	Hs.654464	5079	9	37015371	37015632	262	1	2	2	0.294044905	0.005518027	no
PAX5	INSIDE	Hs.654464	5079	9	36871454	36872766	1313	2	5	4	0.166218205	0.009123084	no
PAX7	INSIDE	Hs.113253	5081	1	18832187	18832464	278	1	2	2	0.132577118	0.002665988	no
PAX7	INSIDE	Hs.113253	5081	1	18835904	18836409	506	2	3	3	0.153998408	0.009734636	no
PCOLCE2	INSIDE	Hs.8944	26577	3	144089894	144090073	180	1	1	1	0.421281341	0.000615783	no
PCOLCE2	INSIDE	Hs.8944	26577	3	144090074	144090640	567	13	8	6	0.139156694	0.003766172	no
PCSK1N	INSIDE	Hs.522640	27344	X	48577947	48578464	518	3	1	2	0.652987906	0.000597721	no
PDE3A	INSIDE	Hs.591150	5139	12	20414343	20414575	233	0	2	2	0.479196179	0.000139924	no
PDE4DIP	INSIDE	Hs.584841	9659	1	143786728	143787883	1156	7	10	6	0.171549564	0.000774416	no
PDGFRA	INSIDE	Hs.74615	5156	4	54791779	54792732	954	5	9	11	0.119308285	0.009305304	no
PFKP	INSIDE	Hs.26010	5214	10	3150867	3151126	260	3	4	2	0.671534587	0.003797662	no
PLXNA4B	INSIDE		NA	7	131911028	131911331	304	1	3	3	0.27729041	0.000787137	no
PODNL1	INSIDE	Hs.448497	79883	19	13904895	13905364	470	3	5	4	0.421270868	0.005110676	no
POU6F2	INSIDE	Hs.137106	11281	7	39420336	39420779	444	3	2	4	0.67012699	1.92E-06	no
PRDM16	INSIDE	Hs.99500	63976	1	3100863	3101844	982	1	7	6	0.215635891	0.000762312	no
PRDM16	INSIDE	Hs.99500	63976	1	3147344	3148382	1039	4	10	9	0.227842337	0.000867683	no
PRDM16	INSIDE	Hs.99500	63976	1	3296534	3297169	636	4	4	3	0.190165016	0.001149441	no
PRDM16	INSIDE	Hs.99500	63976	1	3332330	3332897	568	4	3	4	0.172694065	0.002263896	no
PRDM16	INSIDE	Hs.99500	63976	1	3027796	3028970	1175	5	12	6	0.113832939	0.004003363	no
PRDM16	INSIDE	Hs.99500	63976	1	3137657	3138759	1103	0	4	4	0.172750069	0.004956232	no
PTEN	INSIDE	Hs.500466	5728	10	89613869	89614388	520	0	4	1	0.260307565	0.00807921	no
PTPN21	INSIDE	Hs.437040	11099	14	88087468	88087680	213	4	4	1	0.200363189	0.008608134	no
PTPRZ1	INSIDE	Hs.489824	5803	7	121300690	121301293	604	5	2	6	0.360902886	5.87E-05	no
PTRF	INSIDE	Hs.437191	284119	17	37811410	37811705	296	2	1	2	0.344196348	0.001149441	no
PXDN	INSIDE	Hs.332197	7837	2	1725343	1726044	702	3	4	3	0.393455782	0.006851644	no
PYY	INSIDE	Hs.169249	5697	17	39427228	39427510	283	1	0	2	0.674668897	0.000351863	no
RASGEF1C	INSIDE	Hs.190559	255426	5	179495397	179496018	622	2	1	3	0.20062261	0.005431667	no
RASIP1	INSIDE	Hs.233955	54922	19	53930012	53930793	782	1	6	6	0.34591194	7.62E-05	no
RIN3	INSIDE	Hs.326822	79890	14	92223802	92224833	1032	10	7	8	0.163559403	0.005846864	no
RUTBC1	INSIDE		NA	17	2221931	2222795	865	2	7	4	0.162716896	0.007610747	no
SEMA6C	INSIDE	Hs.516316	10500	1	149370986	149371297	312	2	3	2	0.169672472	0.000341753	no
SERPINB9	INSIDE	Hs.104879	5272	6	2836823	2837175	353	1	1	3	0.455014922	0.000166263	no
SH2D3C	INSIDE	Hs.306412	10044	9	129556088	129556465	378	4	4	4	0.302305406	3.13E-05	no
SIPA1L3	INSIDE	Hs.655502	23094	19	43375747	43376713	967	1	3	4	0.153361027	0.000867683	no
SKI	INSIDE	Hs.705384	6497	1	2228412	2229015	604	2	4	7	0.085252313	0.003332061	no
SLC1A2	INSIDE	Hs.502338	6506	11	35397192	35397706	515	9	6	6	0.28070599	3.67E-05	no
SLC26A4	INSIDE	Hs.571246	5172	7	107088619	107089828	1210	10	5	12	0.154452029	0.001008779	no
SLC5A1	INSIDE	Hs.1964	6523	22	30769205	30769490	286	1	1	2	0.44947548	0.00397046	no
SLC9A3	INSIDE	Hs.658120	6550	5	559228	559734	507	4	4	4	0.174165573	0.000555153	no
SOX15	INSIDE	Hs.95582	6665	17	7432372	7433734	1363	8	8	8	0.117982768	0.004819314	no
SOX8	INSIDE	Hs.243678	30812	16	976711	976958	248	1	1	2	0.204144499	0.002397291	no
SPATC1	INSIDE	Hs.97726	375686	8	145172262	145173900	1639	1	5	4	0.104002248	0.009164864	no
SPIB	INSIDE	Hs.437905	6689	19	55622868	55623780	913	6	5	6	0.216740855	1.18E-05	no
SPOCK2	INSIDE	Hs.523009	9806	10	73518139	73518384	246	4	1	2	0.460788755	0.003398448	no
SRD5A2	INSIDE	Hs.458345	6716	2	31658452	31658844	393	3	0	1	0.824822664	8.85E-05	no
STC2	INSIDE	Hs.233160	8614	5	172688371	172688586	216	1	0	2	0.568040525	4.55E-05	no
STC2	INSIDE	Hs.233160	8614	5	172687815	172688370	556	6	7	3	0.251449842	0.001467322	no
STK32C	INSIDE	Hs.469002	282974	10	133889975	133890878	904	1	3	5	0.163544796	0.005012074	no
TBCD	INSIDE	Hs.464391	6904	17	78365855	78366384	530	2	0	4	0.276612449	0.007072356	no
TBR1	INSIDE	Hs.705400	10716	2	161981125	161982369	1245	1	8	7	0.15955339	0.007495946	no
TGFB2	INSIDE	Hs.133379	7042	1	216586661	216587189	529	4	4	5	0.385464439	0.000729383	no
TMEM16C	INSIDE	Hs.91791	63982	11	26309962	26310430	469	4	5	5	0.559100412	8.61E-05	no
TMEM51	INSIDE	Hs.465305	55092	1	15413699	15414089	391	1	2	4	0.284106259	0.003806838	no
TPBG	INSIDE	Hs.82128	7162	6	83131631	83132118	488	3	4	5	0.303039822	0.0073902	no
TRAF2	INSIDE	Hs.522506	7186	9	138924003	138924223	221	1	1	2	0.174529302	0.005932381	no
TRIM54	INSIDE	Hs.516036	57159	2	27382336	27382910	575	1	4	4	0.269059285	0.000872582	no
TSHZ1	INSIDE	Hs.284217	10194	18	71128785	71129819	1035	1	3	4	0.218852482	0.004819314	no
TSPAN9	INSIDE	Hs.504517	10867	12	3178449	3179624	1176	9	4	7	0.145971786	0.003721962	no
TTC16	INSIDE	Hs.642748	158248	9	129525955	129526667	713	0	2	6	0.171863187	0.001737527	no
VLDLR	INSIDE	Hs.370422	7436	9	2613032	2613419	388	3	1	4	0.190739181	0.000697695	no
WNT16	INSIDE	Hs.272375	51384	7	120757606	120758191	586	6	1	4	0.37578483	0.006415768	no
WNT7A	INSIDE	Hs.72290	7476	3	13870813	13871741	929	1	5	4	0.179103897	1.18E-05	no
ZDHHC19	INSIDE	Hs.111591	131540	3	197418528	197418781	254	7	0	2	1.146948356	0.000872582	no
ZFYVE28	INSIDE	Hs.292056	57732	4	2371133	2371718	586	0	4	6	0.191215253	0.002121075	no
ZH2C2	INSIDE		NA	5	102483364	102483556	193	1	0	1	0.129916057	0.007723284	no

ZNF569	INSIDE	Hs.511848	148266	19	42649046	42649861	816	4	5	4	0.316630327	0.002271372	no
ZNF606	INSIDE	Hs.654967	80095	19	63205355	63206386	1032	13	16	10	0.108556748	0.003211258	no
ZNF701	INSIDE		NA	19	57765660	57766460	801	2	0	3	0.284488512	0.003110207	no
ZNF790	INSIDE	Hs.282067	388536	19	42020793	42021125	333	5	4	2	0.311030177	0.00092553	no
ZNF813	INSIDE	Hs.705729	126017	19	58663023	58663270	248	4	4	1	0.342918913	0.008068783	no
C9orf102	DOWNSTREAM	Hs.632686	56959	9	97822935	97823411	477	6	5	4	0.155707055	0.004301151	yes
CDKN2A	DOWNSTREAM	Hs.512599	1029	9	21955241	21955580	340	2	2	2	0.426524369	3.88E-05	yes
EOMES	DOWNSTREAM	Hs.591663	8320	3	27729229	27729644	416	2	5	3	0.464927573	0.00021076	yes
FLJ45983	DOWNSTREAM	Hs.669736	399717	10	8131489	8132185	697	11	13	7	0.124346215	0.007850636	yes
HAND1	DOWNSTREAM	Hs.152531	9421	5	153833449	153834101	653	2	2	3	0.261013239	0.00991594	yes
ISL1	DOWNSTREAM	Hs.505	3670	5	50730630	50730927	298	1	3	3	0.732442293	0.000363682	yes
RPRML	DOWNSTREAM	Hs.367999	388394	17	42410273	42410742	470	3	0	4	0.376285612	0.004028031	yes
ASB10	DOWNSTREAM	Hs.647081	136371	7	150500999	150501333	335	4	0	1	0.467257763	0.007044737	no
C19orf19	DOWNSTREAM	Hs.104777	284451	19	404720	405488	769	1	3	4	0.170414264	0.006991101	no
CTDP1	DOWNSTREAM	Hs.465490	9150	18	75616745	75617176	432	2	2	4	0.175323164	0.007831582	no
DLL1	DOWNSTREAM	Hs.379912	28514	6	170431172	170431687	516	1	3	5	0.317316565	0.000450862	no
DLX5	DOWNSTREAM	Hs.99348	1749	7	96483659	96483830	172	2	1	1	0.204016244	0.002661375	no
FLJ32447	DOWNSTREAM		NA	2	222879077	222880374	1298	9	9	4	0.119191644	0.001159579	no
FLJ39743	DOWNSTREAM	Hs.668070	283777	15	96788601	96789124	524	2	1	2	0.673041777	0.000275772	no
FLJ45187	DOWNSTREAM		NA	10	21837595	21837980	386	3	7	4	0.139612352	0.005271963	no
FLJ45187	DOWNSTREAM		NA	10	21838299	21839108	810	6	6	9	0.108259469	0.005792558	no
FLJ46347	DOWNSTREAM		NA	2	174899350	174899911	562	9	1	5	0.372153457	0.003418706	no
FOXB2	DOWNSTREAM	Hs.553843	442425	9	78827671	78828159	489	5	5	4	0.224966742	0.002528802	no
FOXD2	DOWNSTREAM	Hs.166188	2306	1	47682970	47683491	522	5	5	6	0.226827554	0.00567102	no
FOXD4L3	DOWNSTREAM		NA	9	70111021	70111420	400	3	5	1	0.488083849	0.000987277	no
HLXB9	DOWNSTREAM		NA	7	156489949	156490698	750	5	2	8	-0.09963906	0.009335554	no
LHPP	DOWNSTREAM	Hs.527748	64077	10	126293609	126294243	635	0	20	2	0.222979576	0.000424888	no
LRPAP1	DOWNSTREAM	Hs.533136	4043	4	3482971	3483941	971	0	8	4	0.168731827	0.006020137	no
MEIS1	DOWNSTREAM	Hs.526754	4211	2	66657397	66657632	236	1	2	2	0.368575795	3.76E-05	no
MGC33407	DOWNSTREAM	Hs.209206	284382	19	8668264	8669288	1025	3	7	6	0.197098396	0.001599197	no
MSX1	DOWNSTREAM	Hs.424414	4487	4	4918035	4919227	1193	11	12	3	0.207069308	0.004819314	no
NKX6-1	DOWNSTREAM	Hs.546270	4825	4	85633298	85633772	475	5	6	4	0.169273544	0.0073902	no
NOC4L	DOWNSTREAM	Hs.558536	79050	12	131205116	131205669	554	2	7	2	0.389538618	0.002161257	no
ODZ3	DOWNSTREAM	Hs.130438	55714	4	183964869	183965098	230	2	1	1	0.290218044	0.000909204	no
RAD51AP1	DOWNSTREAM	Hs.705500	10635	12	4541945	4542311	367	2	1	1	0.345792368	0.005458994	no
SAMD1	DOWNSTREAM	Hs.140309	90378	19	14057472	14057890	419	8	5	4	0.195133726	0.009123084	no
SIX3	DOWNSTREAM	Hs.658847	6496	2	45033228	45033900	673	4	7	5	0.202249269	0.000438954	no
SLC1A3	DOWNSTREAM	Hs.481918	6507	5	36725745	36726530	786	5	5	7	0.174713531	0.00016339	no
SP8	DOWNSTREAM	Hs.195922	221833	7	20782661	20783126	466	3	6	5	0.242385433	0.006204594	no
UNCX4.1	DOWNSTREAM		NA	7	1246886	1247082	197	0	1	2	0.304657449	0.001642501	no
chr10:042570626-042570670	Unknown		NA	10	42570108	42570939	832	9	21	8	0.132502746	0.002040298	yes
chr10:101271888-101271932	Unknown		NA	10	101271803	101272056	254	3	3	2	0.462389702	0.000202855	yes
chr10:112392988-112393032	Unknown		NA	10	112392444	112393116	673	2	3	3	0.420745377	0.001263186	yes
chr11:043920340-043920384	Unknown		NA	11	43919927	43920678	752	11	10	4	0.112754925	0.005792302	yes
chr11:043921791-043921843	Unknown		NA	11	43921641	43921932	292	3	0	2	0.341127127	0.00029262	yes
chr11:078826519-078826563	Unknown		NA	11	78825171	78826568	1398	12	15	7	0.097457712	0.002491971	yes
chr12:005411804-005411848	Unknown		NA	12	5411467	5412119	653	7	3	5	0.222176376	0.008165393	yes
chr12:052430762-052430821	Unknown		NA	12	52430621	52431552	932	7	4	7	0.134351535	0.008855542	yes
chr14:036044633-036044677	Unknown		NA	14	36044320	36044736	417	6	2	5	0.144770964	0.001126745	yes
chr14:036186039-036186083	Unknown		NA	14	36185611	36186264	654	3	9	5	0.270903396	0.001599197	yes
chr14:036187223-036187267	Unknown		NA	14	36186265	36187651	1387	11	12	15	0.13812342	0.0015404	yes
chr15:032594005-032594049	Unknown		NA	15	32593763	32595035	1273	13	10	6	0.139676816	0.005531526	yes
chr15:087722918-087722967	Unknown		NA	15	87722624	87723963	1340	17	17	12	0.085322601	0.009264995	yes
chr15:087743702-087743746	Unknown		NA	15	87743554	87743881	328	3	7	3	0.308981922	0.003586334	yes
chr15:094753880-094753935	Unknown		NA	15	94753776	94754391	616	5	6	2	0.211647011	0.008165393	yes
chr18:005186914-005186958	Unknown		NA	18	5186754	5187081	328	2	0	3	0.341891255	0.008608134	yes
chr18:005187248-005187292	Unknown		NA	18	5187082	5187443	362	3	4	2	0.212437642	0.006415768	yes
chr18:005620001-005620045	Unknown		NA	18	5619215	5620115	901	12	14	9	0.221483523	0.002725499	yes
chr18:022489587-022489632	Unknown		NA	18	22489365	22489862	498	2	0	3	0.376099406	0.000146287	yes
chr18:053622261-053622309	Unknown		NA	18	53622175	53622377	203	1	0	1	0.695956035	0.002110835	yes
chr3:087924771-087924815	Unknown		NA	3	87924294	87925259	966	6	6	9	0.114598194	0.005068387	yes
chr5:072631482-072631526	Unknown		NA	5	72631398	72631595	198	3	2	1	0.239954506	0.006910451	yes
chr5:077182795-077182839	Unknown		NA	5	77182619	77183031	413	7	1	4	0.204273144	0.004785914	yes
chr5:092965549-092965593	Unknown		NA	5	92965138	92965656	519	2	2	2	0.248917632	0.005684483	yes
chr5:134852878-134852922	Unknown		NA	5	134852638	134852960	323	4	1	2	0.437899907	0.009164864	yes
chr5:137605598-137605643	Unknown		NA	5	137605402	137605684	283	5	5	3	0.242226931	0.005687689	yes
chr5:155088915-155088959	Unknown		NA	5	155088837	155089030	194	2	0	1	0.31175033	0.006629406	yes
chr8:030889223-030889267	Unknown		NA	8	30888710	30889759	1050	18	7	9	0.121815333	0.007179819	yes
chr9:014338970-014339026	Unknown		NA	9	14338903	14339130	228	0	1	1	0.403207646	0.000194418	yes
chr9:017897421-017897465	Unknown		NA	9	17896392	17897667	1276	11	8	14	0.098920522	0.009764856	yes
chr1:002762248-002762292	Unknown		NA	1	2762175	2762364	190	1	2	2	0.255443138	0.00817337	no
chr1:002837042-002837086	Unknown		NA	1	2836144	2837206	1063	0	9	4	0.121970759	0.004805681	no
chr1:022484852-022484896	Unknown		NA	1	22483887	22485240	1354	4	9	3	0.138655335	0.009569657	no
chr1:046686821-046686865	Unknown		NA	1	46686615	46687623	1009	2	8	4	0.123850986	0.002161257	no
chr1:046729099-046729143	Unknown		NA	1	46728650	46729389	740	6	10	7	0.11179663	0.00741527	no
chr1:047963255-047963299	Unknown		NA	1	47962790	47963364	575	2	0	1	0.242825376	0.008124333	no
chr1:050666116-050666160	Unknown		NA	1	50665352	50666749	1398	5	12	9	0.112995432	0.009028653	no
chr1:065241419-065241463	Unknown		NA	1	65241305	65241493	189	1	1	2	0.162775894	0.007063241	no
chr1:084098896-084098940	Unknown		NA	1	84098640	84099682	1043	3	12	8	0.079659082	0.007461798	no
chr1:088700757-088700816	Unknown		NA	1	88700684	88700847	164	1	1	1	0.426111947	0.001171119	no
chr1:110428880-110428924	Unknown		NA	1	110428020	110429189	1170	13	8	11	0.152853008	0.000461399	no
chr1:143751455-143751499	Unknown		NA	1	143750738	143751750	1013	6	4	7	0.192324064	0.009109189	no
chr1:150348046-150348090	Unknown		NA	1	150346486	150348136	1651	15	4	7	0.282287119	0.003478873	no
chr1:225796676-225796720	Unknown		NA	1	225796218	225797148	931	12	19	9	0.098901229	0.008303066	no
chr10:045039700-045039744	Unknown		NA	10	45039635	45039941	307	1	4	2	0.325338831	1.18E-05	no
chr10:049184259-049184309	Unknown		NA	10	49184049	49184401	353	0	3	2	0.158057571	0.007526649	no
chr10:079733603-079733662	Unknown		NA	10	79733422	79733885	464	1	0	3	0.464511999	0.001091254	no
chr10:088382093-088382137	Unknown		NA	10	88381688	88382214	527	8	3	4	0.148110039	0.008034791	no
chr10:093637287-093637331	Unknown		NA	10	93637218	93637394	177	4	6	1	0.276637251	0.006995306	no
chr10:121761403-121761447	Unknown		NA	10	121761262	121761496	235	0	3	2	0.43941714	3.72E-05	no

chr10:126127506-126127550	Unknown	NA	10	126127257	126127742	486	1	1	6	0.205669825	0.004799771	no
chr10:129837631-129837675	Unknown	NA	10	129837617	129837901	285	2	3	3	0.116952099	0.006020137	no
chr11:001014727-001014771	Unknown	NA	11	1014647	1015468	822	1	8	7	0.232205739	0.000415619	no
chr11:001315593-001315637	Unknown	NA	11	1315431	1316484	1054	5	5	3	0.14141351	0.006174761	no
chr11:018167519-018167563	Unknown	NA	11	18166971	18167733	763	3	4	2	0.206899143	0.004309577	no
chr11:065003020-065003064	Unknown	NA	11	65002543	65003357	815	3	4	5	0.119598176	0.007120429	no
chr12:088625631-088625675	Unknown	NA	12	88625559	88625851	293	2	1	4	0.562658585	0.004384962	no
chr12:095407623-095407667	Unknown	NA	12	95407267	95407978	712	5	5	8	0.133400973	0.00357547	no
chr12:097375047-097375091	Unknown	NA	12	97374428	97375167	740	4	8	2	0.275566153	0.000130634	no
chr12:113620397-113620441	Unknown	NA	12	113620263	113620488	226	6	0	1	0.286032441	0.004785914	no
chr12:129754079-129754123	Unknown	NA	12	129753945	129754204	260	0	3	2	0.378375593	0.000302788	no
chr12:131229709-131229753	Unknown	NA	12	131229593	131229939	347	1	0	2	0.209884341	0.006702282	no
chr12:131432359-131432403	Unknown	NA	12	131432095	131432996	902	2	8	4	0.192510818	0.003323139	no
chr12:131466402-131466446	Unknown	NA	12	131466317	131466749	433	1	5	2	0.376605506	0.000324328	no
chr12:131466798-131466842	Unknown	NA	12	131466750	131467010	261	2	2	3	0.279686869	0.002867778	no
chr12:131530072-131530116	Unknown	NA	12	131529804	131530414	611	2	6	4	0.249347923	0.003698639	no
chr12:131994991-131995035	Unknown	NA	12	131994847	131995690	844	7	11	8	0.174617508	0.003574802	no
chr13:094418881-094418925	Unknown	NA	13	94418779	94419168	390	1	3	2	0.186578097	0.004031106	no
chr13:110860915-110860974	Unknown	NA	13	110860735	110861107	373	1	1	2	0.193147194	0.005459419	no
chr13:113944864-113944908	Unknown	NA	13	113944829	113945843	1015	5	5	5	-0.211862962	0.001527366	no
chr14:088564061-088564114	Unknown	NA	14	88563932	88564196	265	3	1	2	0.384724763	0.000299639	no
chr15:050883720-050883768	Unknown	NA	15	50883452	50884000	549	2	3	3	0.238057577	0.005669947	no
chr16:001009408-001009452	Unknown	NA	16	1009253	1009502	250	3	1	3	0.276140918	0.000309317	no
chr16:003160651-003160698	Unknown	NA	16	3160404	3160775	372	3	7	3	0.178046629	0.000494033	no
chr16:049432503-049432557	Unknown	NA	16	49432414	49432734	321	4	1	2	0.306116375	0.007017533	no
chr16:083754500-083754544	Unknown	NA	16	83754186	83754958	773	0	6	2	0.220855962	0.00597696	no
chr16:085807639-085807683	Unknown	NA	16	85807533	85808184	652	3	7	6	0.257944326	0.000334579	no
chr16:087485836-087485882	Unknown	NA	16	87485738	87486098	361	0	1	3	0.281742577	0.005271963	no
chr17:000655354-000655399	Unknown	NA	17	655088	655567	480	3	3	3	0.230328687	0.007850636	no
chr17:021355973-021356017	Unknown	NA	17	21355855	21356616	762	3	6	2	0.092661093	0.005728876	no
chr17:077844979-077845027	Unknown	NA	17	77844862	77845387	526	2	1	1	0.231991201	0.004173793	no
chr18:003057416-003057460	Unknown	NA	18	3057201	3057546	346	1	3	3	0.270518851	0.007495946	no
chr18:010404558-010404602	Unknown	NA	18	10404178	10404670	493	1	4	2	0.211264135	0.00010578	no
chr18:070134562-070134606	Unknown	NA	18	70134526	70134916	391	1	3	3	0.17589477	0.006665237	no
chr18:073987792-073987840	Unknown	NA	18	73987623	73987973	351	1	1	2	0.444101778	0.003081509	no
chr18:074498897-074498941	Unknown	NA	18	74498329	74499023	695	3	8	8	0.10114425	0.004775517	no
chr18:074717975-074718019	Unknown	NA	18	74717301	74718047	747	2	3	6	0.377794272	0.001354784	no
chr18:075414911-075414955	Unknown	NA	18	75414129	75415004	876	4	5	3	0.220763323	0.001410244	no
chr18:075437150-075437194	Unknown	NA	18	75436846	75437286	441	1	2	3	0.389024668	0.000368969	no
chr18:075496257-075496301	Unknown	NA	18	75496225	75496665	441	2	1	5	0.232303435	0.001828804	no
chr18:075511187-075511231	Unknown	NA	18	75510923	75511280	358	1	3	3	0.255436539	0.002630754	no
chr19:002462783-002462828	Unknown	NA	19	2462656	2463107	452	0	2	2	0.368578692	0.000364122	no
chr19:010388063-010388107	Unknown	NA	19	10387882	10388157	276	1	1	2	0.276124149	0.000415619	no
chr2:007824903-007824947	Unknown	NA	2	7824369	7825219	851	3	5	6	0.307078333	0.002779577	no
chr2:070969510-070969554	Unknown	NA	2	70969031	70970109	1079	13	6	8	0.104802788	0.001263307	no
chr2:071307810-071307866	Unknown	NA	2	71307622	71307917	296	0	1	2	-0.162615599	0.007381148	no
chr2:073282865-073282909	Unknown	NA	2	73282770	73283040	271	2	2	3	0.472407517	0.00016339	no
chr2:074835279-074835324	Unknown	NA	2	74835192	74835506	315	1	2	1	-0.189168048	0.008741769	no
chr2:095556308-095556352	Unknown	NA	2	95555634	95556928	1295	12	9	2	0.166610923	0.005235835	no
chr2:104828267-104828313	Unknown	NA	2	104827681	104828386	706	5	7	6	0.143675338	0.009159433	no
chr2:106325502-106325546	Unknown	NA	2	106325285	106325990	706	2	3	6	0.165095629	0.001642501	no
chr2:130762643-130762687	Unknown	NA	2	130762559	130763045	487	2	8	5	0.13617187	0.006109424	no
chr2:178986527-178986572	Unknown	NA	2	178986201	178986921	721	6	9	5	0.152040904	0.007345026	no
chr2:219481758-219481807	Unknown	NA	2	219481400	219481881	482	0	3	1	0.526612553	0.000238612	no
chr2:225614863-225614907	Unknown	NA	2	225614706	225614926	221	2	3	1	0.388307199	0.000494033	no
chr2:236752750-236752809	Unknown	NA	2	236752579	236752880	302	2	2	2	0.260475122	0.009694492	no
chr2:240517106-240517150	Unknown	NA	2	240516626	240517167	542	3	3	4	0.206419532	0.003721962	no
chr2:241235802-241235846	Unknown	NA	2	241235618	241236201	584	2	2	3	0.278636612	0.001231975	no
chr20:021029850-021029894	Unknown	NA	20	21029646	21030075	430	7	3	4	0.92257595	0.002823481	no
chr22:016229953-016230000	Unknown	NA	22	16229866	16230028	163	1	1	1	0.701435784	0.000139401	no
chr22:026340446-026340490	Unknown	NA	22	26340420	26340651	232	3	2	2	0.280376432	0.000787137	no
chr22:026364886-026364930	Unknown	NA	22	26364113	26365189	1077	0	1	4	0.219045673	0.002619244	no
chr22:029268009-029268053	Unknown	NA	22	29267666	29268272	607	3	3	3	0.197081332	0.006665237	no
chr22:044420612-044420666	Unknown	NA	22	44420549	44421005	457	1	2	1	0.443352981	0.002490237	no
chr22:048198087-048198132	Unknown	NA	22	48197915	48198528	614	3	5	7	0.130011695	0.007345026	no
chr3:005112885-005112929	Unknown	NA	3	5112461	5113240	780	7	1	6	-0.11853436	0.002845709	no
chr3:030911245-030911289	Unknown	NA	3	30911039	30911332	294	6	2	3	0.711772933	1.66E-05	no
chr3:043380339-043380383	Unknown	NA	3	43379853	43380574	722	2	1	2	0.212713657	0.004945192	no
chr3:113061063-113061107	Unknown	NA	3	113060775	113061854	1080	7	11	9	0.074863489	0.008263145	no
chr3:128336746-128336790	Unknown	NA	3	128335307	128337062	1756	6	7	3	0.150050699	0.005346729	no
chr3:128337133-128337181	Unknown	NA	3	128337088	128337253	166	3	0	1	0.322577864	0.003880277	no
chr3:131465602-131465646	Unknown	NA	3	131465300	131465970	671	6	7	5	0.23172034	0.001434959	no
chr3:158743483-158743532	Unknown	NA	3	158742882	158743546	665	9	2	3	0.429308213	9.18E-05	no
chr3:169450407-169450451	Unknown	NA	3	169450050	169450857	808	11	15	8	0.121291012	0.004586125	no
chr4:001512675-001512719	Unknown	NA	4	1512489	1513072	584	2	9	2	0.177121016	0.004586125	no
chr4:003641332-003641376	Unknown	NA	4	3641030	3641500	471	1	5	4	0.143423327	0.006719358	no
chr4:008398657-008398701	Unknown	NA	4	8398528	8399355	828	3	4	2	0.431952857	0.00010578	no
chr4:008696800-008696844	Unknown	NA	4	8696519	8697356	838	0	4	5	0.178091051	0.002189638	no
chr4:014473804-014473848	Unknown	NA	4	14473494	14473913	420	5	4	4	0.173588088	5.61E-06	no
chr4:017391744-017391788	Unknown	NA	4	17391498	17391992	495	0	7	4	0.285022382	0.000692731	no
chr4:041562167-041562211	Unknown	NA	4	41561879	41562348	470	3	2	3	0.23110007	0.007914338	no
chr4:093322701-093322745	Unknown	NA	4	93322082	93322981	900	3	5	3	0.20092616	0.006638187	no
chr4:117846404-117846448	Unknown	NA	4	117845909	117846604	696	3	8	7	0.236367885	0.005237948	no
chr4:141638636-141638680	Unknown	NA	4	141638070	141639039	970	12	4	8	0.199820865	0.002630754	no
chr4:175371953-175371998	Unknown	NA	4	175371783	175372244	462	5	2	3	0.282220117	0.009109189	no
chr4:183299478-183299526	Unknown	NA	4	183298806	183299663	858	2	3	4	0.12795107	0.002979694	no
chr4:186287064-186287108	Unknown	NA	4	186286993	186287190	198	3	0	2	0.239933609	0.009647458	no
chr5:001698180-001698224	Unknown	NA	5	1697516	1698285	770	4	0	2	0.405373402	0.003703259	no
chr5:172001705-172001755	Unknown	NA	5	172001698	172002004	307	3	0	1	0.504888277	0.000763971	no
chr5:180474779-180474823	Unknown	NA	5	180474661	180475015	355	3	1	3	0.359479449	0.00099867	no

chr6:006491870-006491915	Unknown	NA	6	6491471	6492265	795	7	8	8	0.248981643	9.42E-06	no
chr6:030539836-030539880	Unknown	NA	6	30539528	30540655	1128	7	10	5	0.113634183	0.008013985	no
chr6:066861276-066861320	Unknown	NA	6	66861109	66861566	458	2	6	1	0.208108925	0.001541679	no
chr6:088932964-088933008	Unknown	NA	6	88932836	88933155	320	4	2	3	0.192345945	0.004004905	no
chr6:146961681-146961725	Unknown	NA	6	146961590	146962285	696	3	1	4	0.21746018	0.000611634	no
chr7:001608369-001608413	Unknown	NA	7	1608185	1608550	366	0	5	4	0.23109984	0.001760746	no
chr7:001610038-001610082	Unknown	NA	7	1608886	1610400	1515	2	11	4	0.132737971	0.00232708	no
chr7:012409593-012409651	Unknown	NA	7	12409339	12409700	362	0	2	1	0.463456794	0.005734996	no
chr7:012410231-012410288	Unknown	NA	7	12409701	12411176	1476	7	2	7	0.132143933	0.003721962	no
chr7:018093373-018093417	Unknown	NA	7	18093302	18093473	172	3	2	2	0.218547883	0.003922167	no
chr7:035543718-035543762	Unknown	NA	7	35543253	35543850	598	6	3	3	0.133211701	0.003043166	no
chr7:054924140-054924199	Unknown	NA	7	54923952	54924222	271	1	0	3	0.416970966	0.000296467	no
chr7:084407178-084407222	Unknown	NA	7	84406769	84407535	767	4	6	3	0.192139846	0.001131271	no
chr7:113512986-113513033	Unknown	NA	7	113512868	113513251	384	1	0	4	0.45527738	0.002904014	no
chr7:129213048-129213092	Unknown	NA	7	129212487	129213114	628	6	4	5	0.204647841	0.003800041	no
chr7:154866854-154866905	Unknown	NA	7	154866478	154866980	503	2	1	1	0.473065806	0.000202855	no
chr7:157146800-157146844	Unknown	NA	7	157146691	157147671	981	2	6	3	0.128024793	0.009544182	no
chr8:024855808-024855852	Unknown	NA	8	24855659	24856134	476	3	3	5	0.253979872	0.004322494	no
chr8:027792950-027792994	Unknown	NA	8	27792644	27793386	743	3	3	4	0.225365737	0.003054314	no
chr8:142840103-142840147	Unknown	NA	8	142839474	142840280	807	3	4	5	0.197029439	0.001467322	no
chr8:144383265-144383309	Unknown	NA	8	144382718	144383420	703	2	6	2	0.191028116	0.000762312	no
chr9:002923758-002923802	Unknown	NA	9	2923599	2924222	624	1	2	2	0.227549627	0.003043166	no
chr9:014336226-014336274	Unknown	NA	9	14335759	14336676	918	6	4	6	0.283232296	0.003495931	no
chr9:014336965-014337011	Unknown	NA	9	14336715	14337149	435	3	2	4	0.76507312	9.42E-06	no
chr9:016716900-016716944	Unknown	NA	9	16716638	16716977	340	2	3	2	0.447206189	1.58E-05	no
chr9:029202717-029202761	Unknown	NA	9	29202599	29202841	243	1	2	3	0.237593907	0.001745764	no
chr9:044167268-044167316	Unknown	NA	9	44167109	44167618	510	3	5	1	0.830087342	0.000710481	no
chr9:073251288-073251332	Unknown	NA	9	73251098	73251442	345	1	2	2	0.577729161	9.85E-05	no
chr9:092919954-092919998	Unknown	NA	9	92919465	92920297	833	4	7	9	0.167324322	0.000909204	no
chr9:093483951-093483995	Unknown	NA	9	93483907	93484278	372	1	2	2	0.1740491	0.007461798	no
chr9:106770451-106770495	Unknown	NA	9	106770385	106770586	202	1	2	2	0.419186016	2.89E-05	no
chr9:106770713-106770757	Unknown	NA	9	106770587	106770787	201	2	1	2	0.386660011	0.002725499	no
chr9:123699042-123699088	Unknown	NA	9	123698489	123699112	624	2	1	6	0.31420761	8.85E-05	no
chr9:127817844-127817888	Unknown	NA	9	127817520	127817921	402	0	2	4	0.16729368	0.005363106	no
chr9:128038017-128038070	Unknown	NA	9	128037870	128038093	224	0	2	2	0.343454803	0.001482741	no
chr9:128038266-128038325	Unknown	NA	9	128038094	128038349	256	0	2	3	0.4274606	0.005260226	no
chr9:133707322-133707374	Unknown	NA	9	133707169	133707431	263	3	1	2	0.462501812	1.44E-05	no
chr9:139521172-139521218	Unknown	NA	9	139520569	139521485	917	1	2	5	0.224714925	0.000992507	no
chrY:000112906-000112949	Unknown	NA	Y	112585	113331	747	0	5	4	0.182989304	0.003383714	no
