# Supplementary material for: BRAF mutation-specific promoter methylation of FOX genes in colorectal cancer
Source: Clin Epigenetics. 2013 Jan 16;5(1):2. doi: 10.1186/1868-7083-5-2 (PMC3599401; doi:10.1186/1868-7083-5-2)
Supplement: Additional file 5 — Primers used in this study. [file 1868-7083-5-2-S5.pdf]

Additional File 5 - Primers used

Primer name	Sequence
FW *CPSF6* .qPCR	AAGATTGCCTTCATGGAATTGAG
RV *CPSF6* .qPCR	TCGTGATCTACTATGGTCCCTCTCT
HNRNPM FW	GAGGCCATGCTCCTGGG
HNRNPM -RV	TTTAGCATCTTCCATGTGAAATCG
FOXB2 _BSA_Fw	M13 GGTGGTGGGAAGTATTTTTTATTTT
FOXB2 _BSA_Rev	M13 AAACCAAACCCTCACTAAATAAACTC
FOXD3 _BSA_Fw	M13 TTTTGTTTAAGGAGTGTGATAAAGTG
FOXD3 _BSA_Rev	M13 AATTCAAAAAATTATCAACAAAAAAAC
FOXF1 _BSA_Fw	M13 TTTTTGTTAGGGTTTTTGTTAT
FOXF1 _BSA_Rev	M13 AACCCACAAAAACTTAAATTC
